# Discovery of an MLLT1/3 YEATS Domain Chemical Probe

**DOI:** 10.1002/anie.201810617

**Published:** 2018-11-16

**Authors:** Moses Moustakim, Thomas Christott, Octovia P. Monteiro, James Bennett, Charline Giroud, Jennifer Ward, Catherine M. Rogers, Paul Smith, Ioanna Panagakou, Laura Díaz‐Sáez, Suet Ling Felce, Vicki Gamble, Carina Gileadi, Nadia Halidi, David Heidenreich, Apirat Chaikuad, Stefan Knapp, Kilian V. M. Huber, Gillian Farnie, Jag Heer, Nenad Manevski, Gennady Poda, Rima Al‐awar, Darren J. Dixon, Paul E. Brennan, Oleg Fedorov

**Affiliations:** ^1^ Structural Genomics Consortium & Target Discovery Institute University of Oxford, NDMRB Old Road Campus Oxford, OX3 7DQ & OX3 7FZ UK; ^2^ Department of Chemistry University of Oxford Chemistry Research Laboratory Mansfield Road Oxford OX1 3TA UK; ^3^ Structural Genomics Consortium & Botnar Research Centre University of Oxford Windmill Road Oxford OX3 7LD UK; ^4^ Institute for Pharmaceutical Chemistry and Buchmann Institute for Life Sciences Johann Wolfgang Goethe-University 60438 Frankfurt am Main Germany; ^5^ UCB Pharma Ltd Slough SL1 3WE UK; ^6^ Drug Discovery Program Ontario Institute for Cancer Research Toronto ON Canada; ^7^ Leslie Dan Faculty of Pharmacy University of Toronto Toronto ON Canada; ^8^ Department of Pharmacology and Toxicology University of Toronto Toronto ON Canada; ^9^ Alzheimer's Research (UK) Oxford Drug Discovery Institute Nuffield Department of Medicine University of Oxford NDM Research Building Roosevelt Drive Oxford OX3 7FZ UK

**Keywords:** chemical probes, epigenetics, MLLT1, MLLT3, YEATS

## Abstract

YEATS domain (YD) containing proteins are an emerging class of epigenetic targets in drug discovery. Dysregulation of these modified lysine‐binding proteins has been linked to the onset and progression of cancers. We herein report the discovery and characterisation of the first small‐molecule chemical probe, **SGC**‐**iMLLT**, for the YD of MLLT1 (ENL/YEATS1) and MLLT3 (AF9/YEATS3). **SGC‐iMLLT** is a potent and selective inhibitor of MLLT1/3–histone interactions. Excellent selectivity over other human YD proteins (YEATS2/4) and bromodomains was observed. Furthermore, our probe displays cellular target engagement of MLLT1 and MLLT3. The first small‐molecule X‐ray co‐crystal structures with the MLLT1 YD are also reported. This first‐in‐class probe molecule can be used to understand MLLT1/3‐associated biology and the therapeutic potential of small‐molecule YD inhibitors.

Lysine residues bearing acetyl (Kac) or crotonyl (Kcr) marks are fundamental components of the epigenetic code.^1^, ^2^ In addition to the well‐studied binders of acyl lysine, so‐called bromodomains,^3^, ^4^ YEATS (YAF9, ENL, AF9, TAF14, SAS5) domain containing proteins bind acetyl and crotonyl marks on histone tails. Recent reports have suggested that the dysregulation of YEATS domain (YD) containing proteins correlates with the onset and progression of cancers.^5^, ^6^, ^7^ There are four YD‐containing genes in humans (MLLT1, YEATS2, MLLT3, and YEATS4). Despite high sequence homology (88 % YD alignment) and similar roles in complex formation, both MLLT1 and MLLT3 appear to have independent roles in cancers such as acute myeloid leukaemia (AML).^5^, ^6^ MLLT1, which associates with DOT1L,^8^, ^9^, ^10^ has been implicated in Wilms’ tumour progression when mutations occur in the YD^11^ and associates with the AF4 subcomponent in certain rearranged leukaemia types.^12^ MLLT3 is a component of the super elongation complex (SEC),^7^ and unlike MLLT1, it is the most common fusion partner with MLL (mixed lineage leukaemia protein) in AML (ca. 30 % of cases).^7^ Recent reports suggested that MLLT1/3 share a “KILK” motif interaction, also present in the extra‐terminal (ET) domain of BRD3, which is responsible for the recruitment of chromatin‐remodelling complexes, for example, NuRD, BAF, and INO80.^13^ Despite the YD‐containing proteins being correlated with other diseases,^7^ no small‐molecule inhibitors have been reported to further the understanding of YD‐associated biology.

Building on our own and others understanding of developing acetyllysine reading domain inhibitors (bromodomains) exemplified by collaborative efforts targeting the BET bromodomains,^14^ p300/CBP,^15^ PCAF/GCN5,^16^ and others,^17^, ^18^, ^19^, ^20^ we sought to apply synthetic and medicinal chemistry efforts towards the identification of the first YD‐containing protein chemical probe.^21^ Owing to the high sequence homology of MLLT1 and MLLT3, particularly in the YD, achieving selective inhibitors would pose a challenge and indeed may not in fact be desired as dual inhibition may mitigate any functional redundancy in MLLT1/3.

A medium‐throughput screen of the Ontario Institute of Cancer Research (OICR) library (40 000 compounds)^22^ revealed compound **1** as a micromolar inhibitor of the MLLT1 YD in an AlphaScreen (AS) assay (MLLT1: IC_50_=2.1 μm; Figure 1). Compound **1** posed as an attractive chemical starting point for analogue generation owing to intuitive retrosynthetic disconnections and a lack of structural alerts after PAINS^23^ filtering. Guided by flexible docking with ICM^24^ using a model extracted from a previously reported co‐crystal structure of MLLT1:H3Kac27 peptide (Figure 2 A, PDB ID 5J9S), docked poses of compound **1** overlay with the amide bond present in the Kac residue in a flipped conformation (Figure 2 B). YDs demonstrate higher affinities for crotonylated lysine peptides over acetylated ones,^25^, ^26^ which is thought to be related to the presence of a π‐π‐π network between the crotonyl double bond and residues F28, Y78, and F59 in MLLT1.^7^ Docking studies revealed a potential interaction when compound **1** bound to MLLT1 YD between the amide bond CO and backbone NH of Y78, in addition to the amide N‐H interacting with the side chain of S58. The piperidine ring in compound **1** was likely protonated in its bound form (predicted p*K*_a_ 8.1 using ACD/Percepta p*K*_a_),^27^ and because of a number of polar residues close to the entrances of the YD binding channel such as E75, any additional substituents or modifications that decrease the piperidine ring basicity would likely cause a drop off in binding affinity. If analogues did indeed overlay with Kac/cr hinged on the amide bond depicted in Figure 2 B, substituents capable of improving π‐π‐π stacking would be favoured. Although the Kac/Kcr channel in MLLT1 is linear and narrow, both ends contain adequate space for ligand elaboration (Figure 2 B).

**Figure 1:**
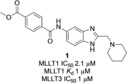
Identified hit MLLT1/3 inhibitor compound **1**.

**Figure 2:**
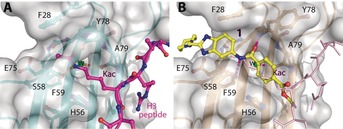
A) Co‐crystal structure of MLLT1 YD with H3Kac27 peptide bound (PDB ID 5J9S). B) Docking studies of compound **1** bound to MLLT1. YD from the co‐crystal structure with H3Kac27 forms similar hydrogen bonds with the backbone NH moieties of Y78 and A79, structural water HOH312, the S58 side chain, and π‐π‐π stacking interactions between the side chains of F28, F59, Y78, and H56.

Ligand development efforts focussed on the conversion of the potentially labile methyl ester of compound **1** into another substituent that would be tolerated and offer a suitable vector to identify new binding interactions. Utilising a “poised” approach,^28^ compound **1** was disconnected into synthons for rapid diversification (Figure 3). Structure–activity relationship (SAR) studies were carried out on compound **1**, culminating in the synthesis of >200 analogues (selected example compounds shown Table 1, others in the Supporting Information).

**Figure 3:**
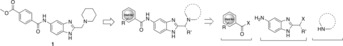
Synthetic disconnection of hit compound **1** into simple building blocks for the rapid construction of potential MLLT1/3 YD inhibitors.

**Table 1:** Selected binding affinity characterisation data of the benzimidazole series for MLLT1 YD.^[a]^

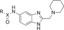	R	MLLT1 IC_50_ [μm]		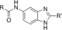	R	R′	MLLT1 IC_50_ [μm]
**1**	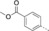	2.1±0.78		**80**	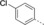	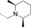	>20
**68**	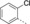	>20		**81**	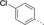	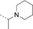	>20
**69**	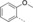	>20		**82**	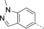	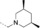	5.7±2.4
**70**	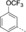	>20		**83**	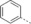	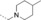	>20
**71**	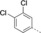	4.5±0.96		**84**	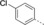	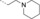	>20
**72**	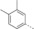	5.5±0.06		**85**	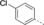	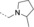	0.33±0.11
**73**	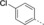	4.7±1.1		**86**	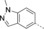	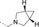	1.4±0.86
**74**	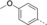	6.4±0.65		**87**	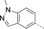	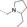	0.3±0.06
**75**	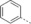	>20^[b]^		**88**	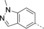	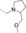	0.97±0.47
**76**	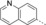	1.2±0.62		**89**	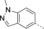	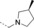	0.6±0.46
**77**	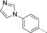	1.0±0.59		**90**	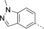	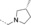	0.73±0.59
**78**	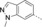	2.1±1.0		**91**	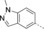	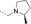	2.0±1.1
**79**	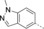	1.6±0.54		**92**	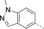	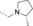	**0.26±0.09**

[a] X=C unless otherwise stated. [b] X=SO.

Derivatives of compound **1** were synthesised from 4‐nitrobenzene‐1,2‐diamine **2**, which was treated with chloroalkyl esters under acidic conditions to form condensed chloromethyl and 2‐chloroethyl benzimidazoles **3**–**5**. Benzimidazoles **3**–**5** were then substituted with amines, providing compounds **6**–**36**. Reduction of nitro compounds **6**–**36** furnished the corresponding anilines **37**–**67**, which were converted into amides and sulfonamides, namely compounds **68**–**183**. Compounds **68**–**183** were screened by AS, and selected examples were further validated by isothermal titration calorimetry (ITC; Scheme 1).

**Scheme 1:**
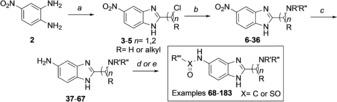
Synthesis of MLLT1/3 benzimidazole based inhibitors. Reagents and conditions: a) ethyl 2‐chloroacetate, ethyl 2‐chloropropanoate (1.2 equiv), 4 n HCl (0.6 m), 16 h, 100 °C, quant.; b) amine (1.2–1.5 equiv), Na_2_CO_3_ (1.5 equiv), 23 °C, 3–82 %; c) H_2_ Pd/C (10 %), MeOH, RT, 16 h, 17–88 %; d) sulfonyl/acid chloride (1.2 equiv), PS‐DIPEA (2 equiv), CH_2_Cl_2_ (0.2 m), 16 h, 11–100 %; e) acid (1.2 equiv), PS‐DIPEA (2 equiv), 16 h, 6–100 %.

Structural modifications of the 2‐ or 3‐ positions in the benzoyl motif of compound **1** were unfavoured (compounds **68**–**70**, Table 1). The introduction of *para* substitutions or 3,4‐disubstitions was better tolerated although the binding activity still decreased relative to compound **1** (compounds **71**–**74**). Replacement of the amide bond of ester **1** with a sulfonamide also ablated activity (compound **75**). The addition of electron‐poor heteroaromatic moieties at the benzoyl position to mimic the methyl ester of compound **1** (compounds **76**–**79**) improved binding activity. With suitable methyl ester replacements identified in compounds **77**–**79**, we focused our attention on further improving potency by modification of the basic amine. Introduction of a chiral centre on the piperidine ring of ester **1** would potentially increase preference for a particular conformation for a salt bridge (e.g., E75). Modifications to the basic amine involving substituted piperidine rings or homologation of the benzylic centre ablated or had no effect (compounds **80**–**84**) on binding affinity compared to unsubstituted compound **73**. Rearrangement of the piperidyl motif to a 2‐methylpyrrolidine or a fused cyclopropyl‐pyrrolidine in combination with the 1‐methyl‐1*H*‐indazol‐5‐yl motif to give compounds **85** and **86** resulted in a net increase in potency (MLLT1 YD: IC_50_ 0.33 μm and 1.4 μm). Elaboration of nanomolar inhibitor **85** into 2‐ethyl‐ and 2‐methoxymethylpyrrolidines **87** and **88** gave similarly or less active compounds.

In efforts to optimise substitution of the pyrrolidine core of compound **85**, all stereoisomers of both 2‐methyl‐ and 3‐methylpyrrolidine derivatives were synthesised (**89**–**92**, Table 1). Interestingly, the 2‐methyl‐substituted pyrrolidines **91** and **92** display a larger difference in binding activity between both enantiomers, with compound **92** displaying the most potent activity ((*S*)‐**92** MLLT1 YD IC_50_ 0.26 μm, (*R*)‐**91** MLLT1 YD IC_50_ 2.0 μm). Binding of compound **92** to MLLT1 YD was validated by ITC (MLLT1 YD *K*_d_ 0.129 μm). Weaker activity was observed for the *R* enantiomer **91** (MLLT1 YD *K*_d_ 0.83 μm), which allows it to be used as a chemically similar, but less active control compound. Compound **92** is predicted to be slightly more conformationally restricted about the aliphatic pyrrolidine ring due to substitution compared with original hit piperidine **1**, which may confer stabilised electrostatic interactions with charged side chain residues in MLLT1 YD. More potent binding observed from the introduction of heteroaromatics in place of the methyl benzoyl motif as with compound **92** may be attributed to a more complementary π‐π‐π “sandwich” stack in the binding site. Compounds **91** and **92** were found to be approximately equipotent against highly homologous MLLT3 YD by ITC (compound **91** *K*_d_ 0.54 μm; compound **92** *K*_d_ 0.077 μm).

Profiling of compound **92** against YEATS2 and YEATS4 revealed excellent selectivity for MLLT1/3 with no activity observed (YEATS2/4 IC_50_>10 μm). Profiling of compound **92** against a selection of bromodomains showed complete selectivity: There was no inhibition of BRD4 (I), CBP, TAF1, CECR2, and FALZ (10 μm using AS). This was validated in a thermal shift assay where both the original hit compound **1** and compound **92** showed no activity against 48 bromodomains (50 μm compound concentrations; see the Supporting Information).

After extensive crystal soaking experiments, an X‐ray co‐crystal structure of compound **92** in complex with MLLT1 YD was obtained (Figure 4 A). Compound **92** occupies the Kac/Kcr binding site of MLLT1 YD, making a number of interactions with loop 1, loop 4, and loop 6 (Figure 4 A; cyan, magenta, and yellow, respectively) adjacent to a structural water molecule. Interestingly, the binding mode of compound **92** matches docked predictions for compound **1**. Y78 adopts two conformations, namely an “in” pose where a π stacking interaction with the amide of **92** can take place, similar to the Kcr:MLLT3 YD crystal structure (PDB ID 5HJB), along with an “out” pose in which the Y78 side chain is now displayed edge‐to‐face with the adjacent side chain of F28.

**Figure 4:**
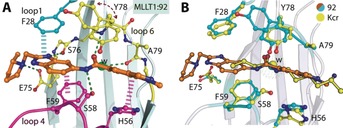
A) Detailed interactions of compound **92** (orange sticks) with MLLT1 YD (loop 1 in cyan, loop 4 in magenta, loop 6 in yellow; PDB ID 6HT1). B) Detailed interactions of compound **92** (orange sticks) with MLLT1 YD (cyan sticks) overlaid with a co‐crystal structure of Kcr (yellow sticks):MLLT3 YD (yellow sticks) (PDB ID 5HJB).

As demonstration of target engagement is a key criteria in chemical probe qualification,^29^ we sought to demonstrate cell activity of compound **92** through multiple methods. We demonstrated MLLT1 target engagement using a cellular thermal shift assay (CETSA)^30^ with endogenous MLLT1 in MV4;11 cells. Compound **92** showed stabilisation of MLLT1 (Figure 5 A–B) whereas the less active control compound **91** elicited no thermal stabilisation up to 10 μm. Next, we performed fluorescence recovery after photobleaching (FRAP) measurements using green fluorescent protein (GFP) tagged MLLT1 wild‐type, MLLT1 mutant, and MLLT3 wild‐type plasmids. Photobleaching of GFP‐tagged wild‐type MLLT1 and MLLT3 had a half recovery time (*t*_1/2_) of 0.46±0.06 s and 0.6±0.09 s, respectively (Figure 5 C,D). As *t*_1/2_ was relatively short, meaning that the majority of the protein is mobile, it was difficult to measure shorter recovery times to study the effect of compound **92**. Therefore, we preincubated the cells with HDAC inhibitor suberoylanilide hydroxamic acid (SAHA, 2.5 μm), preserving global histone acetylation and thus increasing binding of the wild‐type MLLT1 and MLLT3 but not mutant MLLT1 (Figure 5 C, D). Preincubation with SAHA increased *t*_1/2_ to 0.7767±0.09 s and 1.32±0.45 s for wild‐type MLLT1 and MLLT3, respectively. Incubation of cells with compound **92** in presence of SAHA significantly decreased *t*_1/2_ to 0.46±0.05 s and 0.56±0.02 s for MLLT1 and MLLT3, respectively (MLLT1: *P*<0.0001, MLLT3: *P*=0.0185). On the other hand, *t*_1/2_ of cells incubated in compound **91** was not significantly different from that of wild‐type MLLT1 or MLLT3 in the presence of SAHA (Figure 5 C, D). We also developed a full length MLLT1:Histone 3.3 (H3.3) NanoBRET assay to test compound **92**.^31^ Although the MLLT1 NanoBRET assay was responsive to SAHA treatment, showing a significant increase in BRET activity (mBU) relative to the DMSO control, there was no reduction in mBU in response to MLLT1/3 inhibitor treatment. For MLLT3, the NanoBRET assay showed clear dose‐dependent displacement of full‐length MLLT3‐NanoLuc from histone H3.3‐HaloTag (average IC_50_ 0.4±0.08 μm) in HEK293 cells (Figure 5 E and Figure S5).

**Figure 5:**
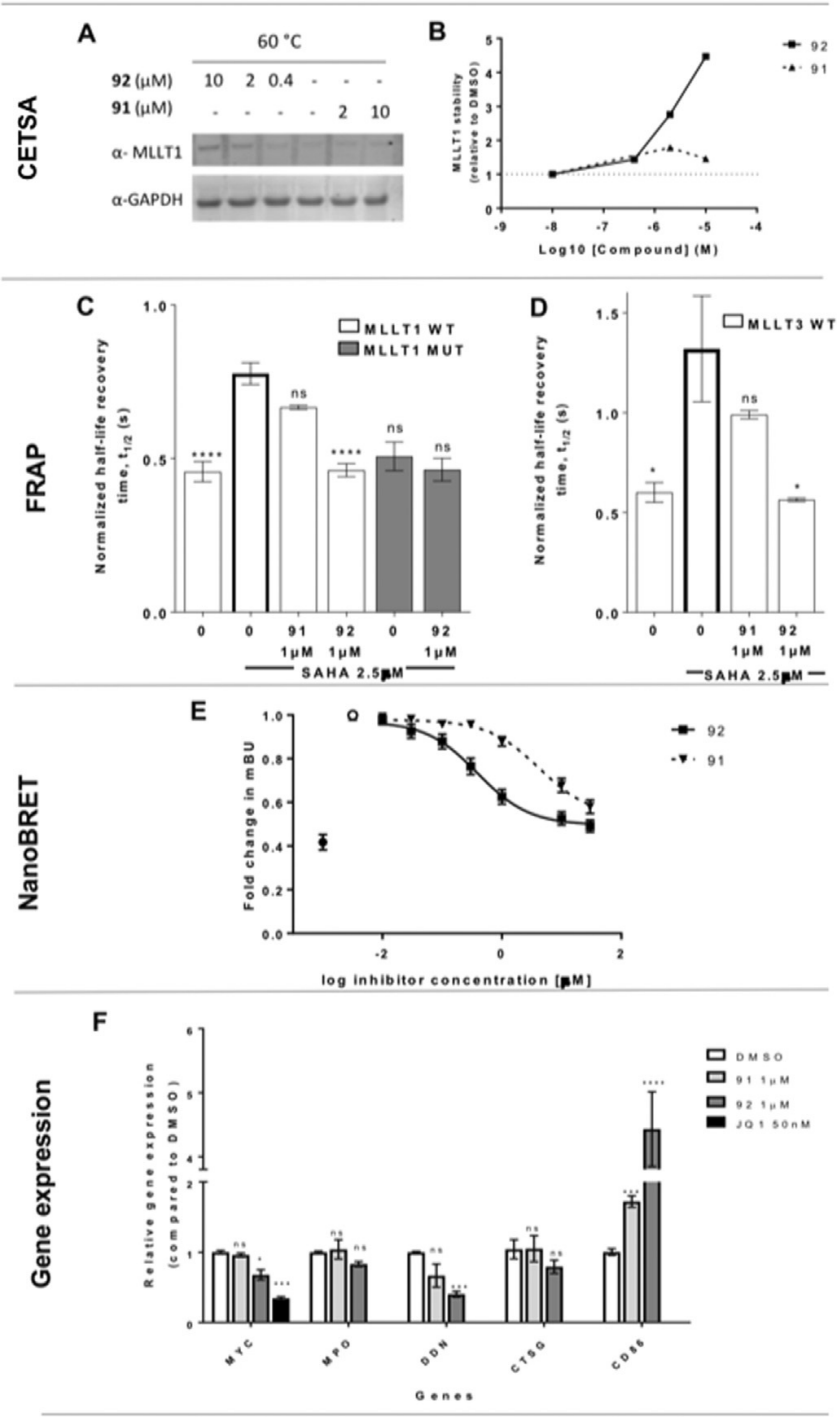
A) Western blot analysis of dose‐dependent thermal stabilization of endogenous MLLT1 in MV4;11 cells with compounds **92** and **91**. B) Stabilisation of MLLT1 induced by **92** and **91** normalised to DMSO. C) FRAP assay showing the half‐life recovery time in U2OS cells transfected with GFP‐tagged MLLT1 wild‐type (WT) and mutant (MUT) and D) MLLT3 WT after treatment with compound **92** or **91** (24 h) in the presence of 2.5 μm SAHA. Graphs represent *n*=3 biological replicates, with *n*≥10 cells per treatment group. Mean±SD, one‐way ANOVA with Tukey–Kramer correction for multiple comparisons **** *P*<0.0001, ** *P*=0.0025. E) NanoBRET dose response for **91** and **92** after 24 h treatment using N‐terminal nanoLuc‐MLLT3 and C‐terminal HaloTag‐H3.3 in HEK293 cells, in the presence of 2.5 μm SAHA. ▪ compound **92**, ▾ compound **91**, • DMSO, ○ DMSO+SAHA. Graphs represent *n*=6 biological replicates, with *n*>4 technical replicates. Mean±SEM, mBU: BRET units. F) qPCR for MYC, MPO, DDN, CTSG, and CD86 expression in MV4;11 cell line after 72 h incubation with compound **91** (1 μm), **92** (1 μm), or **JQ1** (50 nm, positive control for MYC downregulation). Graph represents *n*=3 biological replicates with *n*=2 technical replicates. Mean±SEM; a two‐way ANOVA with post‐hoc Dunnett's multiple comparisons test was used for statistical analyses compared to DMSO (ns not significant, * *P*<0.05, ** *P*<0.005, *** *P*<0.001, **** *P*<0.0001).

Following recent links of MLLT1 knockdown and CRISPR knockout reducing AML tumorigenesis,^5^, ^6^ we profiled genes that were shown to be down‐ (MYC, myeloperoxidase (MPO), dendrin (DDN), cathepsin G (CTSG)) and up (CD86)‐regulated in MV4;11 AML cells after MLLT1 knockdown/out.^5^ Compound **92** changed gene expression of 3/5 genes, showing a reduction in MYC, DDN and an increase in CD86 compared to DMSO. The weaker enantiomer, compound **91**, displayed diminished cell activity in the MLLT1 CETSA (Figure 5 A, B), FRAP (Figure 5 C, D), NanoBRET (Figure 5 E, average IC_50_ 5.8±0.07 μm), and gene expression (Figure 5 F).

We also investigated the metabolic stability of compound **92** in primary human hepatocytes. Compound **92** shows moderate metabolic resistance (*t*_1/2_ 53 min, 48 % remaining after 60 min), with a primary process for metabolism being N demethylation. An *N*‐cyclopropyl indazole analogue, compound **94**, designed to improve pharmacokinetics, largely mitigates N dealkylation observed and retains potent binding activity (MLLT1 *K*_d_ 0.058 μm), which was also rationalised in a co‐crystal structure with MLLT1 YD (see the Supporting Information, PDB ID 6HT0), but the overall half‐life of **94** was inferior to **92**, with pyrrolidine oxidation occurring more rapidly (*t*_1/2_≈30 min). These analogues provide information on how to develop MLLT1/3 probes with good PK properties.

We have reported the discovery of the first small‐molecule inhibitors and a cell‐potent, selective chemical probe for MLLT1 and MLLT3 YDs. Compound **1** was discovered as an initial hit in a medium‐throughput biochemical screen. Simple synthesis allowed rapid generation of >200 analogues and the chemical probe **92** (**SGC‐iMLLT**) with its less active control **91** (**SGC‐iMLLT‐N**). Selectivity was demonstrated over acyllysine binding modules, YEATS2/4, and 48 bromodomains. Using orthogonal combinations of cell target engagement studies (NanoBRET, FRAP, and CETSA), submicromolar cellular activity was confirmed. **SGC‐iMLLT** will enable researchers to design the first biological experiments exploring MLLT1/3 YD inhibition.

## Conflict of interest

N. Manevski and J. Heer are employees and hold shares in UCB Pharma Ltd.

## Supporting information

As a service to our authors and readers, this journal provides supporting information supplied by the authors. Such materials are peer reviewed and may be re‐organized for online delivery, but are not copy‐edited or typeset. Technical support issues arising from supporting information (other than missing files) should be addressed to the authors.

Supplementary

## References

[1] P. Filippakopoulos , S. Knapp , Nat. Rev. Drug Discovery 2014, 13, 337–356. 24751816 10.1038/nrd4286

[2] M. Lawrence , S. Daujat , R. Schneider , Trends Genet. 2016, 32, 42–56. 26704082 10.1016/j.tig.2015.10.007

[3] M. Moustakim , P. G. K. Clark , D. A. Hay , D. J. Dixon , P. E. Brennan , MedChemComm 2016, 7, 2246–2264. 29170712 10.1039/c6md00373g PMC5644722

[4] S. G. Smith , M. M. Zhou , ACS Chem. Biol. 2016, 11, 598–608. 26596782 10.1021/acschembio.5b00831 PMC7295032

[5] L. Wan et al., Nature 2017, 543, 265–269. 28241141 10.1038/nature21687 PMC5372383

[6] M. A. Erb et al., Nature 2017, 543, 270–274. 28241139 10.1038/nature21688 PMC5497220

[7] D. Zhao , Y. Li , X. Xiong , Z. Chen , H. Li , J. Mol. Biol. 2017, 429, 1994–2002. 28300602 10.1016/j.jmb.2017.03.010

[8] A. Yokoyama , M. Lin , A. Naresh , I. Kitabayashi , M. L. Cleary , Cancer Cell 2010, 17, 198–212. 20153263 10.1016/j.ccr.2009.12.040 PMC2824033

[9] D. Mueller et al., Blood 2007, 110, 4445–4454. 17855633 10.1182/blood-2007-05-090514 PMC2234781

[10] N. He , C. K. Chan , B. Sobhian , S. Chou , Y. Xue , M. Liu , T. Alber , M. Benkirane , Q. Zhou , Proc. Natl. Acad. Sci. USA 2011, 108, E636–E645, SE636/1-SE636/2. 10.1073/pnas.1107107108 PMC3169135 21873227

[11] E. J. Perlman et al., Nat. Commun. 2015, 6, 10013. 26635203 10.1038/ncomms10013 PMC4686660

[12] D. T. Zeisig , C. B. Bittner , B. B. Zeisig , M. P. García-Cuéllar , J. L. Hess , R. K. Slany , Oncogene 2005, 24, 5525–5532. 15856011 10.1038/sj.onc.1208699

[13] D. C. Wai et al., J. Biol. Chem. 2018, 293, 7160–7175. 29567837 10.1074/jbc.RA117.000678 PMC5949996

[14] P. Filippakopoulos et al., Nature 2010, 468, 1067–1073. 20871596 10.1038/nature09504 PMC3010259

[15] D. A. Hay et al., J. Am. Chem. Soc. 2014, 136, 9308–9319. 24946055 10.1021/ja412434f PMC4183655

[16] M. Moustakim et al., Angew. Chem. Int. Ed. 2017, 56, 827–831; 10.1002/anie.201610816 PMC5412877 27966810

[17] P. Chen et al., J. Med. Chem. 2016, 59, 1410–1424. 25799074 10.1021/acs.jmedchem.5b00209 PMC4770311

[18] L. Drouin et al., J. Med. Chem. 2015, 58, 2553–2559. 25719566 10.1021/jm501963e PMC4441536

[19] A. E. Fernández-Montalván et al., ACS Chem. Biol. 2017, 12, 2730–2736. 29043777 10.1021/acschembio.7b00708 PMC6218015

[20] D. A. Hay , C. M. Rogers , O. Fedorov , C. Tallant , S. Martin , O. P. Monteiro , S. Muller , S. Knapp , C. J. Schofield , P. E. Brennan , MedChemComm 2015, 6, 1381–1386.

[21] “SGC Chemical Probes” can be found under http://www.thesgc.org/chemical-probes, **2018**.

[22] The “Ontario Institute of Cancer Research” can be found under https://oicr.on.ca.

[23] D. A. Erlanson , J. Med. Chem. 2015, 58, 2088–2090. 25710486 10.1021/acs.jmedchem.5b00294

[24] M. A. C. Neves , M. Totrov , R. Abagyan , J. Comput.-Aided Mol. Des. 2012, 26, 675–686. 22569591 10.1007/s10822-012-9547-0 PMC3398187

[25] Y. Li et al., Cell 2014, 159, 558–571. 25417107 10.1016/j.cell.2014.09.049 PMC4344132

[26] Y. Buganim et al., PLoS One 2010, 5, e9657.

[27] ACD/Labs, *ACD/Labs Percepta Predictor*, ACD/Labs, Toronto, ON, Canada, **2015**.

[28] O. B. Cox et al., Chem. Sci. 2016, 7, 2322–2330. 29910922 10.1039/c5sc03115j PMC5977933

[29] M. E. Bunnage , E. L. P. Chekler , L. H. Jones , Nat. Chem. Biol. 2013, 9, 195–199. 23508172 10.1038/nchembio.1197

[30] R. Jafari , H. Almqvist , H. Axelsson , M. Ignatushchenko , T. Lundbäck , P. Nordlund , D. M. Molina , Nat. Protoc. 2014, 9, 2100–2122. 25101824 10.1038/nprot.2014.138

[31] T. Machleidt , C. C. Woodroofe , M. K. Schwinn , J. Méndez , M. B. Robers , K. Zimmerman , P. Otto , D. L. Daniels , T. A. Kirkland , K. V. Wood , ACS Chem. Biol. 2015, 10, 1797–1804. 26006698 10.1021/acschembio.5b00143

