# Supplementary material for: Discovery of an MLLT1/3 YEATS Domain Chemical Probe
Source: Angew Chem Int Ed Engl. 2018 Nov 16;57(50):16302–7. doi: 10.1002/anie.201810617 (PMC6348381; doi:10.1002/anie.201810617)
Supplement: Supplementary file 1 — Supplementary [file ANIE-57-16302-s001.pdf]

CHEMISTRY
A European Journal

Supporting Information

# From Acenaphthenes to (+)-Delavatine A: Visible-Light-Induced Ring Closure of Methyl (α-Naphthyl) Acrylates

Theodor Peez, Jan-Niclas Luy, Klaus Harms, Ralf Tonner, and Ulrich Koert*[a]

chem_201804735_sm_miscellaneous_information.pdf

## Table of Contents


**I General Experimental**

I.I Solvents and reagents

I.II Purification

I.III Characterisation

**II Practical Experimental**

II.I Synthetic Procedures

II.II AlphaScreen

Supplementary Table 1. AlphaScreen selectivity screen of compound **92** over other acyllysine reading domains.

II.III Isothermal Titration Calorimetry (ITC)

Methods

Supplementary Figure 1. ITC traces of compound **92** and MLLT1 YD.

Supplementary Figure 2. ITC trace of compound **91** and MLLT1 YD.

Supplementary Figure 3. ITC trace of compound **92** and MLLT3 YD.

Supplementary Figure 4. ITC trace of compound **91** and MLLT3 YD.

II.IV Protein Expression and Purification

II.V NanoLuciferase Bioluminescent Resonance Energy Transfer (NanoBRET™) Assay

Methods

Supplementary Figure 5. NanoBRET assay using compound **92** and **91** with MLLT3-NanoLuc and Halotagged-Histone H3.3.

Supplementary Figure 6. NanoBRET dose response for compound **94** with MLLT3-NanoLuc and Halotagged-Histone H3.3.

II.VI Fluorescence Recovery After Photobleaching (FRAP) assay

Methods

II.VII Thermal Shift Assay

Methods

Supplementary Table 2. Thermal stabilisation of compound **1**, **92** and Bromosporine with recombinant human bromodomain proteins.

II.VIII Cellular Thermal Shift Assay (CETSA)

Methods

Supplementary Figure 7. Western blot showing dose dependent heat shock stabilisation of endogenous MLLT1 in HEK293 cells by compound **92**.

II.IX *in vitro* metabolism studies

Supplementary Figure 8. *in vitro* metabolic stability of compound **92**.

II.X Cell Proliferation Studies

Supplementary Figure 9. NCI-60 panel data showing effects of compound **92** on cancer cell proliferation.

II.XI X-ray crystallography

Methods

Supplementary Table 3. Data collection and refinement statistics of MLLT1-ligand complexes.

Supplementary Figure 10. Co-crystal structures of compound **92** and compound **94** with MLLT1 YD with contour maps.

II.XII Analysis of gene expression by quantitative polymerase chain reaction (qPCR)

Methods

Supplementary Table 4. Taqman Gene Expression Assays.

**III Supplementary References**

# I General Experimental

## I.I Solvents and reagents

All solvents were purchased from commercial sources and used without purification (HPLC or analytical grade). Anhydrous solvents were purchased from Acros Organics stored under a nitrogen atmosphere with activated molecular sieves. Standard vacuum line techniques were used, and glassware was flame dried prior to use. Deionised water was sourced using an Elga DV 25 system. Organic solvents were dried during workup using anhydrous $Na_2SO_4$.

## I.II Purification and chromatography

Thin Layer Chromatography (TLC) was carried out using aluminium plates coated with 60 $F_{254}$ silica gel. Plates were visualised using UV light (254 or 365 nm) or staining with Ninhydrin (1 M, EtOH) or 1% aq. $KMnO_4$. Normal-phase silica gel chromatography was carried out using Biotage Isolera One flash column chromatography system (LPLC). Reverse-phase high pressure liquid chromatography (RP-HPLC) was performed using a Waters system equipped with a Waters 2545 Binary Gradient Module, a SecurityGuard™ ULTRA cartridges for EVO-C18 UHPLC HPLC, Kinetex 5 µM EVO C18 100 Å 100 x 3.0 mm column and a Waters SQ Detector 2 using the stated eluent system.

## I.II Characterisation

Infrared spectroscopy was carried out with a Thermo Scientific Nicolet iS5 FT-IR spectrometer fitted with an iD7-ATR accessory, selected absorption maxima ($\nu_{max}$) recorded in wavenumbers ($cm^{-1}$). NMR spectra were recorded using a Bruker Avance 400 MHz spectrometer using the deuterated solvent stated. Chemical shifts (δ) quoted in parts per million (ppm) and referenced to the residual solvent peak. Multiplicities are denoted as s-singlet, d- doublet, t- triplet, q- quartet and quin- quintet and derivatives thereof (br denotes a broad resonance peak). Coupling constants recorded as Hz and round to the nearest 0.1 Hz. Two-dimensional NMR experiments (COSY, HSQC, HMBC) were used to aid the assignment of $^{1}H$ and $^{13}C$ spectra. Low Resolution mass spectra were recorded on a Waters SQ Detector 2 (LC-MS). High Resolution Mass Spectrometry (HRMS) was recorded using an Agilent 6530 QTOF. Melting points were obtained using a Stuart SMP40 apparatus and are reported uncorrected in °C. Optical rotations were recorded using a Perkin Elmer 341 polarimeter; absolute optical rotations are quoted as $[\alpha]_D^T$ at 23 °C, concentration (c) is reported as g/100 mL. Compound names were generated using ChemBioDraw Ultra v14 systematic naming. Atom numbering in structures is purely for the purposes of assignment and does not reflect IUPAC numbering conventions.

**II.** **Practical Experimental**

II.I Synthetic Procedures

2-(chloromethyl)-5-nitro-1H-benzo[d]imidazole **3**

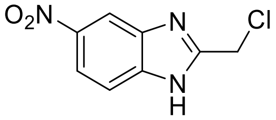

4-nitrobenzene-1,2-diamine **2** (10 g, 65.3 mmol, 1 eq) was dissolved in 4 *N* HCl solution (43.5 mL, 1.5 M) and had ethyl 2-chloroacetate (9.1 mL, 85 mmol, 1.3 eq) added dropwise before being heated to reflux for 6 hours. Upon reaction completion the mixture was cooled to 0°C with an ice bath and had ammonium hydroxide (19.3 mL, 174 mmol, 9 M) dropwise causing a red precipitate to form. The precipitate was filtered off and washed with $H_2O$ (x 3) and dried in an oven vacuum (60°C) to give a dark red solid **3** (13.15 g, 62.1 mmol,95%) which was used without further purification.

**LR-ESI-MS**: $C_8H_7ClN_3O_2$ $[M+H]^+$ *m/z* found 212.20, cald 212.02.

2-(2-chloroethyl)-5-nitro-1H-benzo[d]imidazole **4**

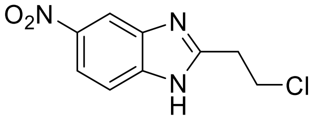

4-nitrobenzene-1,2-diamine **2** (5 g, 32.6 mmol, 1 eq) was dissolved in 4 N HCl solution (46.6 mL, 0.7 M) and had 3-chloropropanoyl chloride (6.22 g, 49.0 mmol, 1.5 eq) added dropwise before being heated to reflux for 16 hours. Upon reaction completion the mixture was cooled to 0°C with an ice bath and had ammonium hydroxide (50 mL, 450 mmol, 9 M) dropwise causing a red precipitate to form. The precipitate was filtered off and dissolved in DCM before being dried over $Na_2SO_4$ which was then filtered off to give a filtrate which was concentrated to  red oil. The oil was then purified using a KP-Sil SNAP 25 g column to give the product **4** as an orange unstable solid (0.282 g, 1.25 mmol, 4%) which was submitted directly into the following step.

**LR-ESI-MS**: $C_9H_9ClN_3O_2$ $[M+H]^+$ *m/z* found 226.29, cald 226.04.

2-(1-chloroethyl)-5-nitro-1H-benzo[d]imidazole **5**

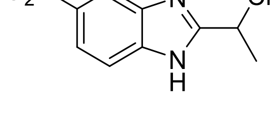

4-nitrobenzene-1,2-diamine **2** (10 g, 65.3 mmol, 1 eq) was dissolved in 4 N HCl solution (131 mL, 0.5 M) and had 2-chloropropanoyl chloride (6.86 mL, 71.8 mmol, 1.1 eq) added dropwise before being heated to reflux for 16 hours. Upon reaction completion the mixture was cooled to 0°C with an ice bath and had ammonium hydroxide (58 mL, 524 mmol, 9 M) dropwise causing a red precipitate to form. The precipitate was filtered off and washed with

$H_2O$ (x 3) and dried in an oven vacuum (60°C) to give a dark red solid **5** (4.92 g, 21.8 mmol, 33%) which was used without further purification.

**LR-ESI-MS**: $C_9H_9ClN_3O_2$ $[M+H]^+$ *m/z* found 226.29, cald 226.04.

2-((2-methylpiperidin-1-yl)methyl)-5-nitro-1H-benzo[d]imidazole **6**

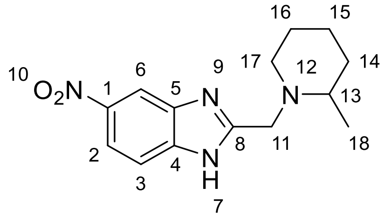


Initially a suspension of 2-(chloromethyl)-5-nitro-1H-benzo[d]imidazole **3** (3 g, 14.2 mmol, 1 eq) and $Na_2CO_3$ (2.25 g, 21.3 mmol, 1.5 eq) in anhydrous MeCN (43 mL, 0.33 M) had 2-methylpiperidine (3.36 mL, 28.4 mmol, 2 eq) added dropwise at room temperature. The reaction was allowed to stir at room temperature overnight. Upon reaction completion the suspension was filtered through a sintered frit and washed with acetone. The filtrate was concentrated to a residue which was purified using a Biotage MPLC KP Sil SNAP 25 g DCM/DCM (20% MeOH) 1:0 to 0:1 to give a red oil which was triturated with acetone to form a red foam **6** (1.884 g, 6.87 mmol, 48%).

**Mpt:** 167.8-169.8 °C; $\mathbf{v_{max}}$ **(cm$^{-1}$)** 2925, 2785, 1442, 1405, 1222, 1093, 887, 572, 541; **$^1$H NMR (400 MHz, DMSO-*d*$_6$) δ** 12.8 (s, 1H, N*H*), 8.4 (d, *J* = 2.3 Hz, 1H, 6), 8.1 (dd, *J* = 2.3, 8.9 Hz, 1H, 2), 7.7 (d, *J* = 8.9 Hz, 1H, 3), 4.1 (d, *J* = 15.1 Hz, 1H, 11'), 3.7 (d, *J* = 15.1 Hz, 1H, 11''), 2.7 (dt, *J* = 4.0, 11.7 Hz, 1H, 17''), 2.4 (ddd, *J* = 2.2, 6.1, 8.8 Hz, 1H, 13), 2.2 (ddd, *J* = 3.6, 10.0, 11.5 Hz, 1H, 17'), 1.7 – 1.6 (m, 2H, 14', 16'), 1.6 – 1.4 (m, 2H, 14'', 16''), 1.4 – 1.2 (m, 2H, 15), 1.1 (d, *J* = 6.2 Hz, 3H, 18); **$^{13}$C NMR (101 MHz, DMSO-*d*$_6$) δ** 158.3 (8), 142.2 (2, 6), 117.3 (3), 55.9 (11), 52.9 (13), 52.0 (17), 34.0 (14), 25.5 (16), 23.4 (15), 19.0 (18); **LR-ESI-MS**: $C_{14}H_{19}N_4O_2$ $[M+H]^+$ *m/z* found 275.23, cald 275.15; **HR-ESI-MS**: $C_{14}H_{19}N_4O_2$ $[M+H]^+$ *m/z* found 275.1508, cald 275.1508.

2-((3-methylpiperidin-1-yl)methyl)-5-nitro-1H-benzo[d]imidazole **7**

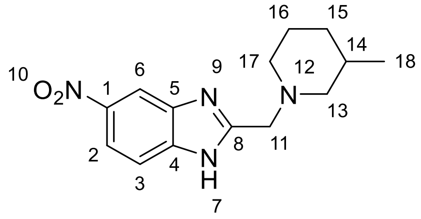


Initially a suspension of 2-(chloromethyl)-5-nitro-1H-benzo[d]imidazole **3** (3 g, 14.2 mmol, 1 eq) and $Na_2CO_3$ (2.25 g, 21.3 mmol, 1.5 eq) in anhydrous MeCN (43 mL, 0.33 M) had 3-methylpiperidine (3.36 mL, 28.4 mmol, 2 eq) added dropwise at room temperature. The reaction was allowed to stir at room temperature overnight. Upon reaction completion the suspension was filtered through a sintered frit and washed with acetone. The filtrate was concentrated to a residue which was purified using a Biotage MPLC KP Sil SNAP 25 g DCM/DCM (20% MeOH) 1:0 to 0:1 to give a red oil which was triturated with acetone to form a red foam **7** (3.08 g, 11.23 mmol, 79%).

**Mpt:** 80.3-82.3 °C; **$v_{max}$ (cm$^{-1}$)** 2925, 2796, 1466, 1449, 1332, 1309, 1117, 1064, 885, 826, 735, 688, 539; **$^1$H NMR (400 MHz, DMSO-*d*$_6$) δ** 12.9 (s, 1H, 7), 8.4 (s, 1H, 6), 8.1 (dd, *J* = 2.2, 8.9 Hz, 1H, 2), 7.7 (d, *J* = 8.8 Hz, 1H, 3), 3.7 (d, *J* = 2.9 Hz, 2H, 11), 2.9 – 2.6 (m, 2H, 17), 2.1 – 1.8 (m, 1H, 13'), 1.8 – 1.4 (m, 5H, 13'', 14, 15', 16), 1.0 – 0.7 (m, 4H, 14'', 18); **$^{13}$C NMR (101 MHz, DMSO-*d*$_6$) δ** 142.3 (2, 6), 61.5 (11), 56.2 (17), 53.6 (13), 32.3 (14), 30.6 (16), 26.3, 24.9 (15), 19.5 (18); **LR-ESI-MS**: $C_{14}H_{19}N_4O_2$ [M+H]$^+$ *m/z* found 275.23, cald 275.15; **HR-ESI-MS**: $C_{14}H_{19}N_4O_2$ [M+H]$^+$ *m/z* found 275.1508, cald 275.1508.

2-((4-methylpiperidin-1-yl)methyl)-5-nitro-1H-benzo[d]imidazole **8**

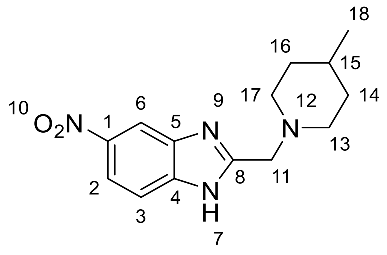


Initially a suspension of 2-(chloromethyl)-5-nitro-1H-benzo[d]imidazole **3** (3 g, 14.2 mmol, 1 eq) and $Na_2CO_3$ (2.25 g, 21.3 mmol, 1.5 eq) in anhydrous MeCN (43 mL, 0.33 M) had 4-methylpiperidine (3.36 mL, 28.4 mmol, 2 eq) added dropwise at room temperature. The reaction was allowed to stir at room temperature overnight. Upon reaction completion the suspension was filtered through a sintered frit and washed with acetone. The filtrate was concentrated to a residue which was purified using a Biotage MPLC KP Sil SNAP 25 g DCM/DCM (20% MeOH) 1:0 to 0:1 to give a red oil which was triturated with acetone to form a red foam **8** (2.43 g, 8.87 mmol, 63%).

**Mpt:** 165.6-167.6 °C; **$v_{max}$ (cm$^{-1}$)** 2941, 1624, 1517, 1465, 1364, 888, 818; **$^1$H NMR (400 MHz, DMSO-*d*$_6$) δ** 12.9 (s, 1H, 7), 8.4 (d, *J* = 2.3 Hz, 1H, 6), 8.1 (dd, *J* = 2.3, 8.9 Hz, 1H, 2), 7.7 (d, *J* = 8.8 Hz, 1H, 3), 3.8 (s, 2H, 11), 2.9 – 2.7 (m, 2H, 13'', 17''), 2.1 (td, *J* = 2.5, 11.6 Hz, 2H, 13', 17'), 1.7 – 1.5 (m, 2H, 14'', 16''), 1.4 – 1.3 (m, 1H, 15), 1.2 (td, *J* = 3.7, 12.2 Hz, 2H, 14', 16'), 0.9 (d, *J* = 6.4 Hz, 3H, 18); **$^{13}$C NMR (101 MHz, DMSO-*d*$_6$) δ** 157.5 (8), 142.3 (2, 6), 117.4 (3), 56.1 (11), 53.6 (13, 17), 33.8 (14, 16), 29.9 (15), 26.3, 21.8 (18); **LR-ESI-MS**: $C_{14}H_{19}N_4O_2$ [M+H]$^+$ *m/z* found 275.24, cald 275.15; **HR-ESI-MS**: $C_{14}H_{19}N_4O_2$ [M+H]$^+$ *m/z* found 275.1505, cald 275.1508.

5-nitro-2-(piperidin-1-ylmethyl)-1H-benzo[d]imidazole **9**

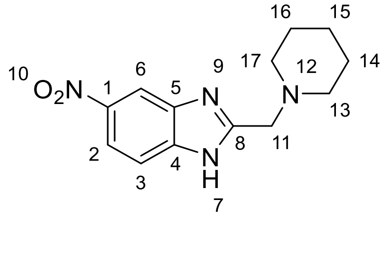


Initially a suspension of 2-(chloromethyl)-5-nitro-1H-benzo[d]imidazole **3** (5 g, 23.6 mmol, 1 eq) and $Na_2CO_3$ (3.76 g, 35.4 mmol, 1.5 eq) in anhydrous MeCN (72 mL, 0.33 M) had piperidine (4.67 mL, 47.3 mmol, 2 eq) added dropwise at room temperature. The reaction was allowed to stir at room temperature overnight. Upon reaction completion the suspension

was filtered through a sintered frit and washed with acetone. The filtrate was concentrated to a residue which was purified using a Biotage MPLC KP Sil SNAP 25 g DCM/DCM (20% MeOH) 1:0 to 0:1 to give a red oil which was triturated with acetone to form an orange solid **9** (2.245 g, 8.62 mmol, 37%).

**Mpt:** 149.3-151.3 °C; **$v_{max}$ ($cm^{-1}$)** 2935, 2801, 1518, 1468, 1413, 1107, 884; **$^1$H NMR (400 MHz, DMSO-*d*$_6$) δ** 12.9 (s, 1H, 7), 8.4 (d, *J* = 2.3 Hz, 1H, 6), 8.1 (dd, *J* = 2.3, 8.9 Hz, 1H, 2), 7.6 (d, *J* = 8.9 Hz, 1H, 3), 3.7 (s, 2H, 11), 2.4 (t, *J* = 5.4 Hz, 4H, 13, 17), 1.5 (p, *J* = 5.6 Hz, 4H, 14, 16), 1.4 (q, *J* = 6.0 Hz, 2H, 15); **$^{13}$C NMR (101 MHz, DMSO-*d*$_6$) δ** 206.5 (1), 157.4 (8), 142.3 (2, 6), 117.4 (3), 56.5 (11), 54.2 (13, 17), 25.4 (14, 16), 23.6 (15); **LR-ESI-MS**: $C_{13}H_{17}N_4O_2$ $[M+H]^+$ *m/z* found 261.23, cald 261.14; **HR-ESI-MS**: $C_{13}H_{17}N_4O_2$ $[M+H]^+$ *m/z* found 261.1349, cald 261.1352.

3-((5-nitro-1H-benzo[d]imidazol-2-yl)methyl)-2,3,4,5-tetrahydro-1H-benzo[d]azepine **10**

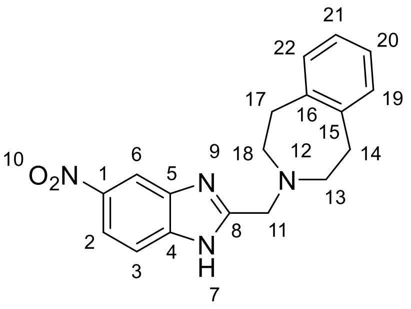


Initially a suspension of 2-(chloromethyl)-5-nitro-1H-benzo[d]imidazole **3** (0.869 g, 4.11 mmol, 1 eq) and $Na_2CO_3$ (0.653 g, 6.16 mmol, 1.5 eq) in anhydrous MeCN (12 mL, 0.33 M) had 2,3,4,5-tetrahydro-1H-benzo[d]azepine (907 mg, 6.16 mmol, 1.5 eq) added at room temperature. The reaction was allowed to stir at room temperature overnight. Upon reaction completion the suspension was filtered through a sintered frit and washed with acetone. The filtrate was concentrated to a residue which was purified using a Biotage MPLC KP Sil SNAP 25 g DCM/DCM (20% MeOH) 1:0 to 0:1 to give a red oil which was triturated with acetone to form a red crystalline solid **10** (1.080 g, 3.35 mmol, 82%).

**Mpt:** 96.5-98.5 °C; **$v_{max}$ ($cm^{-1}$)** 1513, 1330, 827, 735; **$^1$H NMR (400 MHz, DMSO-*d*$_6$) δ** 13.0 (s, 1H, 7), 8.4 (s, 1H, 6), 8.1 (dd, *J* = 2.3, 8.8 Hz, 1H, 2), 7.7 (d, *J* = 8.9 Hz, 1H, 3), 7.2 – 6.8 (m, 4H, 19, 20, 21, 22), 4.0 (s, 2H, 11), 2.9 (dd, *J* = 3.4, 6.6 Hz, 4H, 14, 17), 2.7 – 2.6 (m, 4H, 13, 18); **$^{13}$C NMR (101 MHz, DMSO-*d*$_6$) δ** 142.3 (1), 141.7 (2, 6), 128.7 (20, 21), 126.1 (19, 22), 56.1 (11), 55.2 (13, 18), 35.6 (14, 17); **LR-ESI-MS**: $C_{18}H_{19}N_4O_2$ $[M+H]^+$ *m/z* found 323.26, cald 323.15; **HR-ESI-MS**: $C_{18}H_{19}N_4O_2$ $[M+H]^+$ *m/z* found 323.1502, cald 323.1508.

5-nitro-2-((4-phenylpiperazin-1-yl)methyl)-1H-benzo[d]imidazole **11**

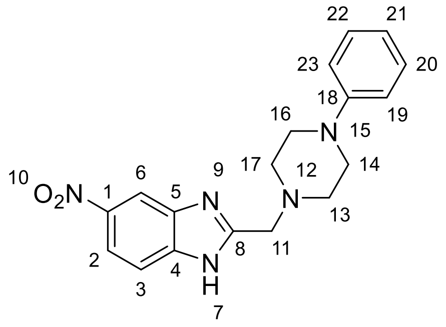


Initially a suspension of 2-(chloromethyl)-5-nitro-1H-benzo[d]imidazole **3** (0.901 g, 4.26 mmol, 1 eq) and $Na_2CO_3$ (0.677 g, 6.39 mmol, 1.5 eq) in anhydrous MeCN (13 mL, 0.33 M) had 1-phenylpiperazine (1.04 g, 6.39 mmol, 1.5 eq) added at room temperature. The reaction was allowed to stir at room temperature overnight. Upon reaction completion the suspension was filtered through a sintered frit and washed with acetone. The filtrate was concentrated to a residue which was purified using a Biotage MPLC KP Sil SNAP 25 g DCM/DCM (20% MeOH) 1:0 to 0:1 to give a red oil which was triturated with acetone to form a red solid **11** (0.713 g, 2.11 mmol, 50%).

**Mpt:** >195 °C; **$\nu_{max}$ (cm$^{-1}$)** 2818, 1598, 1518, 1370, 1462, 1133, 1009, 737, 692, 527; **$^1$H NMR (400 MHz, DMSO-*d*$_6$) δ** 13.0 (s, 1H, 7), 8.4 (s, 1H, 6), 8.1 (dd, *J* = 2.3, 8.8 Hz, 1H, 2), 7.7 (d, *J* = 8.9 Hz, 1H, 3), 7.3 – 7.1 (m, 2H, 20, 22), 7.0 – 6.9 (m, 2H, 19, 23), 6.8 (tt, *J* = 1.1, 7.3 Hz, 1H, 21), 3.9 (s, 2H, 11), 3.2 – 3.1 (m, 4H, 13, 17), 2.6 (dd, *J* = 3.7, 6.1 Hz, 4H, 14, 16); **$^{13}$C NMR (101 MHz, DMSO-*d*$_6$) δ** 150.9 (2, 6), 142.4 (3), 128.9 (20, 22), 118.9 (21), 115.4 (19, 23), 55.6 (11), 52.8 (13, 17), 48.1 (14, 16); **LR-ESI-MS**: $C_{18}H_{20}N_5O_5$ $[M+H]^+$ *m/z* found 338.46, cald 338.16; **HR-ESI-MS**: $C_{18}H_{20}N_5O_5$ $[M+H]^+$ *m/z* found 338.1609, cald 338.1617.

5-nitro-2-((4-(2-(pyrrolidin-1-yl)ethyl)piperidin-1-yl)methyl)-1H-benzo[d]imidazole **12**

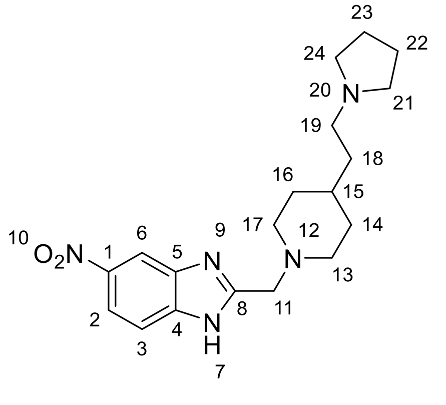


Initially a suspension of 2-(chloromethyl)-5-nitro-1H-benzo[d]imidazole **3** (0.48 g, 2.27 mmol, 1 eq) and $Na_2CO_3$ (0.36 g, 3.40 mmol, 1.5 eq) in anhydrous MeCN (6.8 mL, 0.33 M) had 4-(2-(pyrrolidin-1-yl)ethyl)piperidine (0.62 g, 3.40 mmol, 1.5 eq) added dropwise at room temperature. The reaction was allowed to stir at room temperature overnight. Upon reaction completion the suspension was filtered through a sintered frit and washed with acetone. The filtrate was concentrated to a residue which was purified using a Biotage MPLC KP Sil SNAP 25 g DCM/DCM (20% MeOH) 1:0 to 0:1 to give a red oil which was triturated with acetone to form an orange solid **12** (0.241 g, 0.674 mmol, 30%).

**Mpt:** 88.7-90.7 °C; $\nu_{max}$ **(cm$^{-1}$)** 2918, 2793, 1514, 1331, 1122, 1004, 828, 736; **$^1$H NMR (400 MHz, DMSO-*d*$_6$) δ** 8.4 (d, *J* = 2.3 Hz, 1H, 6), 8.1 (dd, *J* = 2.3, 8.8 Hz, 1H, 2), 7.7 (d, *J* = 8.8 Hz, 1H, 3), 3.7 (s, 2H, 11), 2.9 – 2.7 (m, 2H, 19), 2.4 – 2.3 (m, 6H, 13'', 17'', 21, 24), 2.1 – 2.0 (m, 2H, 13', 17'), 1.6 (dq, *J* = 4.3, 5.6, 8.9 Hz, 6H, 14', 16', 22, 23), 1.4 – 1.1 (m, 5H, 14'', 15, 16'', 18); **$^{13}$C NMR (101 MHz, DMSO-*d*$_6$) δ** 157.6 (8), 142.3 (2, 6), 117.4 (3), 73.8 (11), 56.1 (19), 53.6 (13, 17), 53.2 (21, 24), 35.2 (14, 16), 33.1 (22, 23), 31.9 (15), 23.0 (18); **LR-ESI-MS**: $C_{19}H_{28}N_5O_2$ $[M+H]^+$ *m/z* found 358.56, cald 358.22; **HR-ESI-MS**: $C_{19}H_{28}N_5O_2$ $[M+H]^+$ *m/z* found 358.2232, cald 358.2243.

5-nitro-2-((4-(2-(piperidin-1-yl)ethyl)piperidin-1-yl)methyl)-1H-benzo[d]imidazole **13**

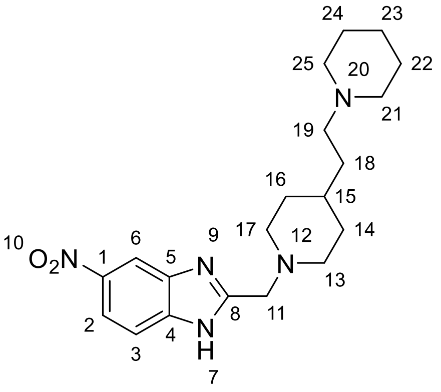


Initially a suspension of 2-(chloromethyl)-5-nitro-1H-benzo[d]imidazole **3** (0.77 g, 3.64 mmol, 1 eq) and $Na_2CO_3$ (0.58 g, 5.45 mmol, 1.5 eq) in anhydrous MeCN (11 mL, 0.33 M) had 1-(2-(piperidin-4-yl)ethyl)piperidine (1.07 g, 5.45 mmol, 1.5 eq) added dropwise at room temperature. The reaction was allowed to stir at room temperature overnight. Upon reaction completion the suspension was filtered through a sintered frit and washed with acetone. The filtrate was concentrated to a residue which was submitted to the next step without further purification **13** (0.592 g, 1.60 mmol, 44%).

2-((2-methylpyrrolidin-1-yl)methyl)-5-nitro-1H-benzo[d]imidazole **14**

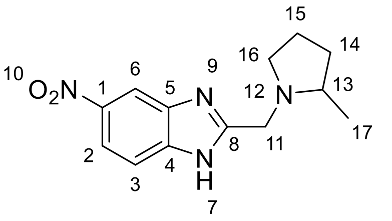


Initially a suspension of 2-(chloromethyl)-5-nitro-1H-benzo[d]imidazole **3** (1.46 g, 6.91 mmol, 1 eq) and $Na_2CO_3$ (1.09 g, 10.36 mmol, 1.5 eq) in anhydrous MeCN (21 mL, 0.33 M) had 2-methylpyrrolidine (0.88 g, 10.36 mmol, 1.5 eq) added dropwise at room temperature. The reaction was allowed to stir at room temperature overnight. Upon reaction completion the suspension was filtered through a sintered frit and washed with acetone. The filtrate was concentrated to a residue which was purified using a Biotage MPLC KP Sil SNAP 25 g DCM/DCM (20% MeOH) 1:0 to 0:1 to give a red oil which was triturated with acetone to form a dark red solid **14** (1.336 g, 5.13 mmol, 74%).

**Mpt:** 69.0-71.0 °C; $\nu_{max}$ **(cm$^{-1}$)** 2960, 2795, 1513, 1332, 1307, 827, 735; **$^1$H NMR (400 MHz, DMSO-*d*$_6$) δ** 12.9 (s, 1H, 7), 8.4 (d, *J* = 2.3 Hz, 1H, 6), 8.1 (dd, *J* = 2.3, 8.9 Hz, 1H, 2), 7.6 (d,

*J* = 8.7 Hz, 1H, 3), 4.1 (d, *J* = 14.7 Hz, 1H, 11''), 3.6 (d, *J* = 14.7 Hz, 1H, 11'), 2.9 (ddd, *J* = 3.4, 7.6, 9.0 Hz, 1H, 13), 2.6 – 2.4 (m, 1H, 16'), 2.3 (q, *J* = 8.7 Hz, 1H, 16''), 1.9 (dddd, *J* = 5.5, 7.2, 9.4, 12.4 Hz, 1H, 14''), 1.7 (dddd, *J* = 4.2, 6.8, 8.9, 17.7 Hz, 2H, 14', 15''), 1.4 (dddd, *J* = 6.3, 8.4, 10.2, 12.3 Hz, 1H, 15'), 1.0 (dd, *J* = 6.1, 15.4 Hz, 3H, 17); **$^{13}$C NMR (101 MHz, DMSO-*d*$_6$) δ** 142.2 (2, 6), 117.3 (3), 59.2 (11), 54.1 (13), 51.0 (16), 32.5 (14), 21.4 (15), 18.8 (17); **LR-ESI-MS**: $C_{13}H_{17}N_4O_2$ [M+H]$^+$ *m/z* found 261.36, cald 261.14; **HR-ESI-MS**: $C_{13}H_{17}N_4O_2$ [M+H]$^+$ *m/z* found 261.1289, cald 261.1352.

2-((cis-2,6-dimethylpiperidin-1-yl)methyl)-5-nitro-1H-benzo[d]imidazole **15**

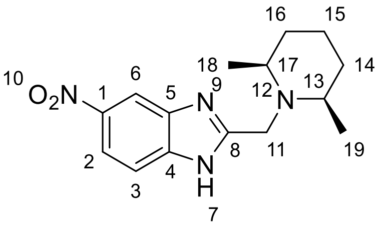


Initially a suspension of 2-(chloromethyl)-5-nitro-1H-benzo[d]imidazole **3** (1 g, 4.73 mmol, 1 eq) and $Na_2CO_3$ (0.751 g, 7.09 mmol, 1.5 eq) in anhydrous MeCN (14 mL, 0.33 M) had cis-2,6-dimethylpiperidine (0.802 g, 7.09 mmol, 1.5 eq) added dropwise at room temperature. The reaction was allowed to stir at room temperature overnight. Upon reaction completion the suspension was filtered through a sintered frit and washed with acetone. The filtrate was concentrated to a residue which was purified using a Biotage MPLC KP Sil SNAP 25 g DCM/DCM (20% MeOH) 1:0 to 0:1 to give a red oil which was triturated with acetone to form red solid **15** (0.506 g, 1.76 mmol, 37%).

**Mpt:** 150.9-152.9 °C; **$ν_{max}$ (cm$^{-1}$)** 2967, 2934, 1515, 1322, 1302, 1062, 818, 738; **$^1$H NMR (400 MHz, DMSO-*d*$_6$) δ** 12.6 (s, 1H, 7), 8.4 (d, *J* = 2.3 Hz, 1H, 6), 8.1 (dd, *J* = 2.3, 8.8 Hz, 1H, 2), 7.7 (d, *J* = 8.8 Hz, 1H, 3), 4.0 (s, 2H, 10''), 2.6 – 2.5 (m, 2H, 12, 16), 1.7 – 1.5 (m, 3H, 13'', 15''), 1.4 – 1.2 (m, 3H, 13', 14, 15'), 1.0 (d, *J* = 6.2 Hz, 6H, 17, 18); **$^{13}$C NMR (101 MHz, DMSO-*d*$_6$) δ** 206.5 (8), 142.1 (2, 6), 117.1 (3), 56.9 (11), 48.6 (13), 46.9 (17), 33.6 (14, 16), 24.0 (15); **LR-ESI-MS**: $C_{15}H_{21}N_4O_2$ [M+H]$^+$ *m/z* found 289.44, cald 289.17; **HR-ESI-MS**: $C_{15}H_{21}N_4O_2$ [M+H]$^+$ *m/z* found 289.1641, cald 289.1665.

2-((4-methylpiperazin-1-yl)methyl)-5-nitro-1H-benzo[d]imidazole **16**

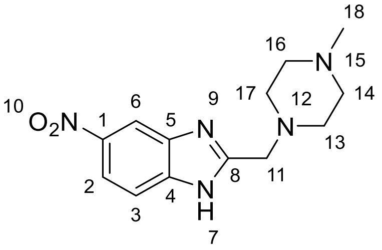


Initially a suspension of 2-(chloromethyl)-5-nitro-1H-benzo[d]imidazole **3** (1 g, 4.73 mmol, 1 eq) and $Na_2CO_3$ (0.751 g, 7.09 mmol, 1.5 eq) in anhydrous MeCN (14 mL, 0.33 M) had 1-methylpiperazine (0.710 g, 7.09 mmol, 1.5 eq) added dropwise at room temperature. The reaction was allowed to stir at room temperature overnight. Upon reaction completion the suspension was filtered through a sintered frit and washed with acetone. The filtrate was concentrated to a residue which was purified using a Biotage MPLC KP Sil SNAP 25 g

DCM/DCM (20% MeOH) 1:0 to 0:1 to give a red oil which was triturated with acetone to form a beige solid **16** (0.424 g, 1.54 mmol, 33%).

**Mpt:** 140.6-142.6 °C; **$\nu_{max}$ (cm$^{-1}$)** 3376, 2805, 1513, 1471, 1456, 1334, 1280, 821, 737; **$^1$H NMR (400 MHz, DMSO-*d*$_6$) δ** 12.9 (s, 1H, 7), 8.4 (d, *J* = 2.2 Hz, 1H, 6), 8.1 (dd, *J* = 2.3, 8.9 Hz, 1H, 2), 7.7 (d, *J* = 8.9 Hz, 1H, 3), 3.8 (s, 2H, 11), 2.5 (s, 4H, 13, 17), 2.4 (s, 4H, 14, 16), 2.2 (s, 3H, 18); **$^{13}$C NMR (101 MHz, DMSO-*d*$_6$) δ** 206.4 (8), 157.0 (1), 142.3 (2, 6), 117.4 (3), 55.6 (11), 54.5, 52.8 (14, 16), 48.6 (18), 45.7, 30.7; **LR-ESI-MS**: $C_{13}H_{18}N_5O_2$ [M+H]$^+$ *m/z* found 276.40, cald 276.15; **HR-ESI-MS**: $C_{13}H_{18}N_5O_2$ [M+H]$^+$ *m/z* found 276.1446, cald 276.1460.

*N*, *N*-dimethyl-1-(5-nitro-1H-benzo[d]imidazol-2-yl)methanamine **17**

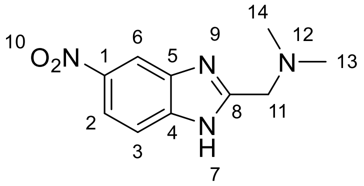


Initially a suspension of 2-(chloromethyl)-5-nitro-1H-benzo[d]imidazole **3** (1 g, 4.73 mmol, 1 eq) and $Na_2CO_3$ (0.751 g, 7.09 mmol, 1.5 eq) in anhydrous MeCN (14 mL, 0.33 M) had dimethylamine (3.54 mL, 7.09 mmol, 2 M in THF, 1.5 eq) added dropwise at room temperature. The reaction was allowed to stir at room temperature overnight. Upon reaction completion the suspension was filtered through a sintered frit and washed with acetone. The filtrate was concentrated to a residue which was purified using a Biotage MPLC KP Sil SNAP 25 g DCM/DCM (20% MeOH) 1:0 to 0:1 to give a red oil which was triturated with acetone to form brown solid **17** (0.761 g, 3.46 mmol, 73%).

**Mpt:** 148.1-150.1 °C; **$\nu_{max}$ (cm$^{-1}$)** 2778, 1453, 1335, 1310, 1036, 823, 736; **$^1$H NMR (400 MHz, DMSO-*d*$_6$) δ** 13.0 (s, 1H, 7), 8.4 (s, 1H, 6), 8.1 (dd, *J* = 2.3, 8.9 Hz, 1H, 2), 7.6 (d, *J* = 8.9 Hz, 1H, 3), 3.7 (s, 2H, 11), 2.3 (s, 6H, 13, 14); **$^{13}$C NMR (101 MHz, DMSO-*d*$_6$) δ** 142.3 (2, 6), 117.4 (3), 56.9 (11), 45.3 (13, 14); **LR-ESI-MS**: $C_{10}H_{13}N_4O_2$ [M+H]$^+$ *m/z* found 221.31, cald 221.10; **HR-ESI-MS**: $C_{10}H_{13}N_4O_2$ [M+H]$^+$ *m/z* found 221.1029, cald 221.1039.

5-nitro-2-(2-(piperidin-1-yl)ethyl)-1H-benzo[d]imidazole **18**

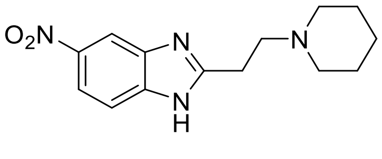


Initially a suspension of 2-(2-chloroethyl)-5-nitro-1H-benzo[d]imidazole **4** (1 g, 4.43 mmol, 1 eq) and $Na_2CO_3$ (0.705 g, 6.65 mmol, 1.5 eq) in anhydrous MeCN (13.4 mL, 0.33 M) had piperidine (0.657 mL, 6.65 mmol, 1.5 eq) added dropwise at room temperature. The reaction was allowed to stir at room temperature overnight. Upon reaction completion the suspension was filtered through a sintered frit and washed with acetone. The filtrate was concentrated to a residue which was purified using a Biotage MPLC KP Sil SNAP 25 g DCM/DCM (20% MeOH) 1:0 to 0:1 to give a red oil which was triturated with acetone to form an unstable orange foam **18** (0.106 g, 0.386 mmol, 9%) which was immediately submitted to the following step.

**LR-ESI-MS**: $C_{14}H_{19}N_4O_2$ $[M+H]^+$ *m/z* found 275.43, cald 275.15;

2-((cis-3,5-dimethylpiperidin-1-yl)methyl)-5-nitro-1H-benzo[d]imidazole **19**

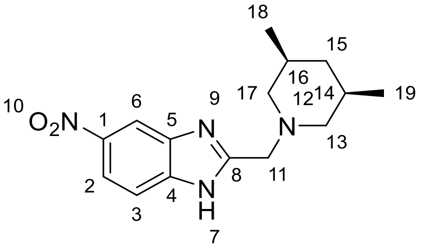


Initially a suspension of 2-(chloromethyl)-5-nitro-1H-benzo[d]imidazole **3** (1 g, 4.73 mmol, 1 eq) and $Na_2CO_3$ (0.751 g, 7.09 mmol, 1.5 eq) in anhydrous MeCN (13.4 mL, 0.33 M) had cis-3,5-dimethylpiperidine (0.802 g, 7.09 mmol, 1.5 eq) added dropwise at room temperature. The reaction was allowed to stir at room temperature overnight. Upon reaction completion the suspension was filtered through a sintered frit and washed with acetone. The filtrate was concentrated to a residue which was purified using a Biotage MPLC KP Sil SNAP 25 g DCM/DCM (20% MeOH) 1:0 to 0:1 to give a red oil which was triturated with acetone to form an orange foam **19** (0.979 g, 3.40 mmol, 72%).

**Mpt:** 108.9-110.9 °C; **$v_{max}$ ($cm^{-1}$)** 2949, 1515, 1332, 1065, 880, 826, 736; **$^1H$ NMR (400 MHz, DMSO-*d*$_6$) δ** 12.9 (s, 1H, 7), 8.6 – 8.3 (m, 1H, 6), 8.1 (dd, *J* = 2.3, 8.8 Hz, 1H, 2), 7.7 (d, *J* = 8.9 Hz, 1H, 3), 3.8 (s, 2H, 11), 2.8 – 2.7 (m, 2H, 13", 17"), 1.8 – 1.5 (m, 5H, 13', 14, 15, 16, 17'), 0.8 (d, *J* = 6.2 Hz, 6H, 18, 19), 0.6 – 0.4 (m, 1H, 15''); **$^{13}C$ NMR (101 MHz, DMSO-*d*$_6$) δ** 142.3 (2, 6), 61.1 (11), 55.9 (13, 17), 41.5 (14, 16), 30.6 (15), 19.4 (18, 19); **LR-ESI-MS**: $C_{15}H_{21}N_4O_2$ $[M+H]^+$ *m/z* found 289.26, cald 289.17; **HR-ESI-MS**: $C_{15}H_{21}N_4O_2$ $[M+H]^+$ *m/z* found 289.1654, cald 289.1665.

5-nitro-2-(1-(piperidin-1-yl)ethyl)-1H-benzo[d]imidazole **20**

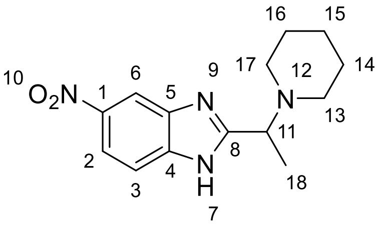


Initially a suspension of 2-(1-chloroethyl)-5-nitro-1H-benzo[d]imidazole **5** (1 g, 4.43 mmol, 1 eq) and $Na_2CO_3$ (0.705 g, 6.65 mmol, 1.5 eq) in anhydrous MeCN (11 mL, 0.4 M) had piperidine (0.657 mL, 6.65 mmol, 1.5 eq) added dropwise at room temperature. The reaction was allowed to stir at room temperature overnight. Upon reaction completion the suspension was filtered through a sintered frit and washed with acetone. The filtrate was concentrated to a residue which was purified using a Biotage MPLC KP Sil SNAP 25 g DCM/DCM (20% MeOH) 1:0 to 0:1 to give a red oil which was triturated with acetone to form an orange foam **20** (0.695 g, 2.53 mmol, 57%) which was used immediately in the following step.

**$^1H$ NMR (400 MHz, DMSO-*d*$_6$) δ** 12.9 (s, 1H, 7), 8.4 (s, 1H, 6), 8.1 (dd, *J* = 2.3, 8.9 Hz, 1H, 2), 7.7 (d, *J* = 8.9 Hz, 1H, 3), 3.4 – 3.3 (m, 1H, 11), 2.4 (t, *J* = 5.2 Hz, 4H, 13, 17), 1.5 (p, *J* = 5.5 Hz, 4H, 14, 16), 1.4 (d, *J* = 6.9 Hz, 3H, 18), 1.4 (q, *J* = 6.0, 6.9 Hz, 2H, 15); **$^{13}C$ NMR**

**(101 MHz, DMSO-*d*$_6$) δ** 142.2 (2, 6), 117.3 (3), 64.9 (11), 58.5 (13), 50.2 (17), 25.8 (14, 16), 24.1 (15), 13.8 (18); **LR-ESI-MS**: $C_{14}H_{19}N_4O_2$ $[M+H]^+$ *m/z* found 275.27, cald 275.15.

2-((3-methylpyrrolidin-1-yl)methyl)-5-nitro-1H-benzo[d]imidazole **21**

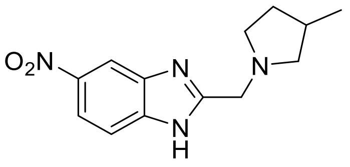


Initially a suspension of 2-(chloromethyl)-5-nitro-1H-benzo[d]imidazole **3** (1.1 g, 5.22 mmol, 1 eq) and $Na_2CO_3$ (1.38 g, 13.05 mmol, 2.5 eq) in anhydrous MeCN (15 mL, 0.4 M) had 3-methylpyrrolidine, HCl (0.952 g, 7.83 mmol, 1.5 eq) added at room temperature. The reaction was allowed to stir at room temperature overnight. Upon reaction completion the suspension was filtered through a sintered frit and washed with acetone. The filtrate was concentrated to a residue which was purified using a Biotage MPLC KP Sil SNAP 25 g DCM/DCM (20% MeOH) 1:0 to 0:1 to give a red oil which was triturated with acetone to form an orange foam **21** (0.04 g, 0.158 mmol, 3%) which was used immediately in the following step.

**LR-ESI-MS**: $C_{13}H_{17}N_4O_2$ $[M+H]^+$ *m/z* found 261.36, cald 261.14.

2-((trans-3,4-dimethylpyrrolidin-1-yl)methyl)-5-nitro-1H-benzo[d]imidazole **22**

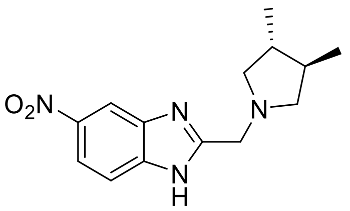


Initially a suspension of 2-(chloromethyl)-5-nitro-1H-benzo[d]imidazole **3** (0.886 g, 4.19 mmol, 1 eq) and $Na_2CO_3$ (1.11 g, 10.47 mmol, 2.5 eq) in anhydrous MeCN (13 mL, 0.4 M) had trans-3,4-dimethylpyrrolidine, HCl (0.852 g, 6.28 mmol, 1.5 eq) added at room temperature. The reaction was allowed to stir at room temperature overnight. Upon reaction completion the suspension was filtered through a sintered frit and washed with acetone. The filtrate was concentrated to a residue which was purified using a Biotage MPLC KP Sil SNAP 25 g DCM/DCM (20% MeOH) 1:0 to 0:1 to give a red oil which was triturated with acetone to form an unstable orange foam **22** (0.39 g, 1.42 mmol, 34%) which was used immediately in the following step.

(*R*)-2-((2-methylpyrrolidin-1-yl)methyl)-5-nitro-1H-benzo[d]imidazole **23**

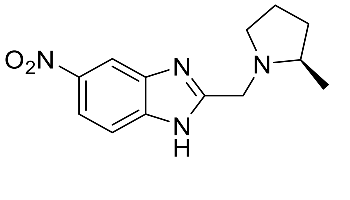


Initially a suspension of 2-(chloromethyl)-5-nitro-1H-benzo[d]imidazole **3** (1.25 g, 5.92 mmol, 1 eq) and $Na_2CO_3$ (1.57 g, 14.8 mmol, 2.5 eq) in anhydrous MeCN (18 mL, 0.4 M) had (*R*)-2-methylpyrrolidine, HCl (1.08 g, 8.87 mmol, 1.5 eq) added at room temperature. The reaction

was allowed to stir at room temperature overnight. Upon reaction completion the suspension was filtered through a sintered frit and washed with acetone. The filtrate was concentrated to a residue which was submitted to the next step without further purification **23** (0.741 g, 2.85 mmol, 48%).

**LR-ESI-MS**: $C_{13}H_{17}N_4O_2$ $[M+H]^+$ *m/z* found 261.37, cald 261.14.

(*R*)-2-((3-methylpyrrolidin-1-yl)methyl)-5-nitro-1H-benzo[d]imidazole **24**

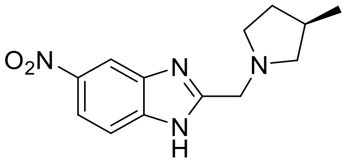

Initially a suspension of 2-(chloromethyl)-5-nitro-1H-benzo[d]imidazole **3** (0.925 g, 4.37 mmol, 1 eq) and $Na_2CO_3$ (1.16 g, 10.92 mmol, 2.5 eq) in anhydrous MeCN (13 mL, 0.33 M) had (*R*)-3-methylpyrrolidine, HCl (0.797 g, 6.55 mmol, 1.5 eq) added at room temperature. The reaction was allowed to stir at room temperature overnight. Upon reaction completion the suspension was filtered through a sintered frit and washed with acetone. The filtrate was concentrated to a residue which was submitted to the next step without further purification **24** (0.399 g, 1.53 mmol, 35%).

**LR-ESI-MS**: $C_{13}H_{17}N_4O_2$ $[M+H]^+$ *m/z* found 261.23, cald 261.14.

(*S*)-2-((3-methylpyrrolidin-1-yl)methyl)-5-nitro-1H-benzo[d]imidazole **25**

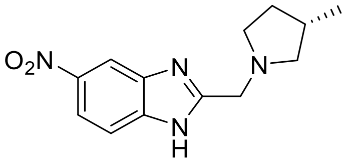

Initially a suspension of 2-(chloromethyl)-5-nitro-1H-benzo[d]imidazole **3** (0.363 g, 1.71 mmol, 1 eq) and $Na_2CO_3$ (0.454 g, 4.28 mmol, 2.5 eq) in anhydrous MeCN (5 mL, 0.33 M) had (*S*)-3-methylpyrrolidine, HCl (0.250 g, 2.06 mmol, 1.2 eq) added at room temperature. The reaction was allowed to stir at room temperature overnight. Upon reaction completion the suspension was filtered through a sintered frit and washed with acetone. The filtrate was concentrated to a residue which was submitted to the next step without further purification **25** (*quant*).

**LR-ESI-MS**: $C_{13}H_{17}N_4O_2$ $[M+H]^+$ *m/z* found 261.22, cald 261.14.

2-((3-azabicyclo[3.1.0]hexan-3-yl)methyl)-5-nitro-1H-benzo[d]imidazole **26**

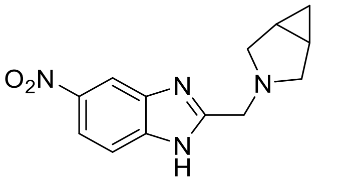

Initially a suspension of 2-(chloromethyl)-5-nitro-1H-benzo[d]imidazole **3** (1.47 g, 6.97 mmol, 1 eq) and $Na_2CO_3$ (1.85 g, 17.42 mmol, 2.5 eq) in anhydrous MeCN (21 mL, 0.33 M) had 3-

azabicyclo[3.1.0]hexane, HCl (1 g, 8.36 mmol, 1.2 eq) added at room temperature. The reaction was allowed to stir at room temperature overnight. Upon reaction completion the suspension was filtered through a sintered frit and washed with acetone. The filtrate was concentrated to a residue which was submitted to the next step without further purification **26** (*quant*).

**LR-ESI-MS**: $C_{13}H_{15}N_4O_2$ $[M+H]^+$ *m/z* found 259.21, cald 259.12.

2-((3,3-difluoropyrrolidin-1-yl)methyl)-5-nitro-H-benzo[d]imidazole **27**

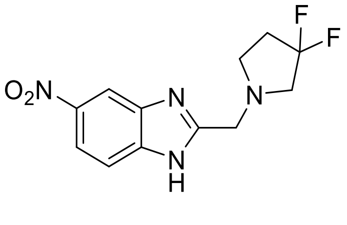


Initially a suspension of 2-(chloromethyl)-5-nitro-1H-benzo[d]imidazole **3** (0.614 g, 2.90 mmol, 1 eq) and $Na_2CO_3$ (0.769 g, 7.26 mmol, 2.5 eq) in anhydrous MeCN (8.8 mL, 0.33 M) had 3,3-difluoropyrrolidine, HCl (0.5 g, 3.48 mmol, 1.2 eq) added at room temperature. The reaction was allowed to stir at room temperature overnight. Upon reaction completion the suspension was filtered through a sintered frit and washed with acetone. The filtrate was concentrated to a residue which was submitted to the next step without further purification **27** (*quant*).

**LR-ESI-MS**: $C_{12}H_{13}F_2N_4O_2$ $[M+H]^+$ *m/z* found 283.17, cald 283.10.

(*S*)-2-((2-methylpyrrolidin-1-yl)methyl)-5-nitro-1H-benzo[d]imidazole **28**

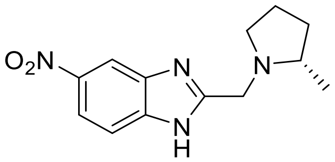


Initially a suspension of 2-(chloromethyl)-5-nitro-1H-benzo[d]imidazole **3** (1.45 g, 6.85 mmol, 1 eq) and $Na_2CO_3$ (1.82 g, 17.1 mmol, 2.5 eq) in anhydrous MeCN (20 mL, 0.33 M) had (*S*)-2-methylpyrrolidine, HCl (1 g, 8.22 mmol, 1.2 eq) added at room temperature. The reaction was allowed to stir at room temperature overnight. Upon reaction completion the suspension was filtered through a sintered frit and washed with acetone. The filtrate was concentrated to a residue which was submitted to the next step without further purification **28** (*quant*).

**LR-ESI-MS**: $C_{13}H_{17}N_4O_2$ $[M+H]^+$ *m/z* found 261.23, cald 261.14.

2-((3-methylpyrrolidin-1-yl)methyl)-5-nitro-1H-benzo[d]imidazole **29**

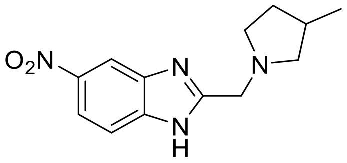

Initially a suspension of 2-(chloromethyl)-5-nitro-1H-benzo[d]imidazole **3** (1.45 g, 6.85 mmol, 1 eq) and $Na_2CO_3$ (1.82 g, 17.1 mmol, 2.5 eq) in anhydrous MeCN (20 mL, 0.33 M) had 3-methylpyrrolidine, HCl (1 g, 8.22 mmol, 1.2 eq) added at room temperature. The reaction was allowed to stir at room temperature overnight. Upon reaction completion the suspension was filtered through a sintered frit and washed with acetone. The filtrate was concentrated to a residue which was submitted to the next step without further purification **29** (*quant*).

**LR-ESI-MS**: $C_{13}H_{17}N_4O_2$ $[M+H]^+$ *m/z* found 261.23, cald 261.14.

2-((2,2-dimethylpyrrolidin-1-yl)methyl)-5-nitro-1H-benzo[d]imidazole **30**

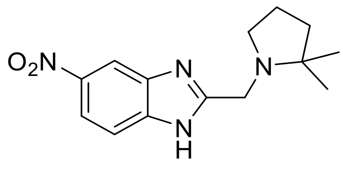

Initially a suspension of 2-(chloromethyl)-5-nitro-1H-benzo[d]imidazole **3** (1.3 g, 6.14 mmol, 1 eq) and $Na_2CO_3$ (1.63 g, 15.4 mmol, 2.5 eq) in anhydrous MeCN (18 mL, 0.33 M) had 2,2-dimethylpyrrolidine, HCl (1 g, 7.37 mmol, 1.2 eq) added at room temperature. The reaction was allowed to stir at room temperature overnight. Upon reaction completion the suspension was filtered through a sintered frit and washed with acetone. The filtrate was concentrated to a residue which was submitted to the next step without further purification **30** (*quant*).

**LR-ESI-MS**: $C_{14}H_{19}N_4O_2$ $[M+H]^+$ *m/z* found 275.35, cald 275.15.

2-((2-ethylpyrrolidin-1-yl)methyl)-5-nitro-1H-benzo[d]imidazole **31**

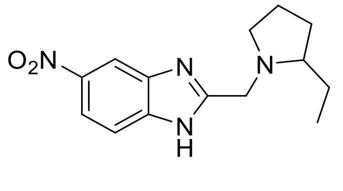

Initially a suspension of 2-(chloromethyl)-5-nitro-1H-benzo[d]imidazole **3** (1.3 g, 6.14 mmol, 1 eq) and $Na_2CO_3$ (1.63 g, 15.4 mmol, 2.5 eq) in anhydrous MeCN (18 mL, 0.33 M) had 2-ethylpyrrolidine, HCl (1 g, 7.37 mmol, 1.2 eq) added at room temperature. The reaction was allowed to stir at room temperature overnight. Upon reaction completion the suspension was filtered through a sintered frit and washed with acetone. The filtrate was concentrated to a residue which was submitted to the next step without further purification **31** (*quant*).

**LR-ESI-MS**: $C_{14}H_{19}N_4O_2$ $[M+H]^+$ *m/z* found 275.35, cald 275.15.

(1*S*,4*S*)-5-((5-nitro-1H-benzo[d]imidazol-2-yl)methyl)-2-oxa-5-azabicyclo[2.2.1]heptane **32**

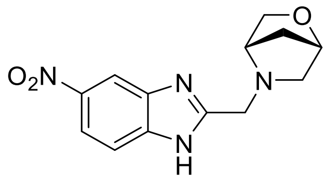

Initially a suspension of 2-(chloromethyl)-5-nitro-1H-benzo[d]imidazole **3** (1.3 g, 6.14 mmol, 1 eq) and $Na_2CO_3$ (1.63 g, 15.4 mmol, 2.5 eq) in anhydrous MeCN (18 mL, 0.33 M) had

(1*S*,4*S*)-2-oxa-5-azabicyclo[2.2.1]heptane, HCl (1 g, 7.37 mmol, 1.2 eq) added at room temperature. The reaction was allowed to stir at room temperature overnight. Upon reaction completion the suspension was filtered through a sintered frit and washed with acetone. The filtrate was concentrated to a residue which was submitted to the next step without further purification **32** (*quant*).

**LR-ESI-MS**: $C_{13}H_{15}N_4O_3$ $[M+H]^+$ *m/z* found 275.31, cald 275.11.

tert-butyl (2-(methyl((5-nitro-1H-benzo[d]imidazol-2-yl)methyl)amino)ethyl)carbamate **33**

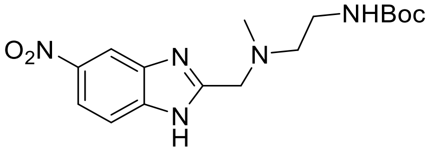


Initially a suspension of 2-(chloromethyl)-5-nitro-1H-benzo[d]imidazole **3** (0.5 g, 2.36 mmol, 1 eq) and $Na_2CO_3$ (0.301 g, 2.84 mmol, 2.5 eq) in anhydrous MeCN (7.2 mL, 0.33 M) had tert-butyl (2-(methylamino)ethyl)carbamate (0.494 g, 2.84 mmol, 1.2 eq) added at room temperature. The reaction was allowed to stir at room temperature overnight. Upon reaction completion the suspension was filtered through a sintered frit and washed with acetone. The filtrate was concentrated to a residue which was submitted to the next step without further purification **33** (*quant*).

**LR-ESI-MS**: $C_{16}H_{24}N_5O_4$ $[M+H]^+$ *m/z* found 350.28, cald 350.18.

2-((2-(methoxymethyl)pyrrolidin-1-yl)methyl)-5-nitro-1H-benzo[d]imidazole **34**

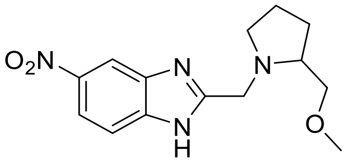


Initially a suspension of 2-(chloromethyl)-5-nitro-1H-benzo[d]imidazole **3** (0.5 g, 2.36 mmol, 1 eq) and $Na_2CO_3$ (0.301 g, 2.84 mmol, 2.5 eq) in anhydrous MeCN (7.2 mL, 0.33 M) had 2-(methoxymethyl)pyrrolidine (0.299 g, 2.60 mmol, 1.1 eq) added at room temperature. The reaction was allowed to stir at room temperature overnight. Upon reaction completion the suspension was filtered through a sintered frit and washed with acetone. The filtrate was concentrated to a residue which was submitted to the next step without further purification **34** (*quant*).

**LR-ESI-MS**: $C_{14}H_{19}N_4O_3$ $[M+H]^+$ *m/z* found 291.20, cald 291.15.

2-((2-isopropylpyrrolidin-1-yl)methyl)-5-nitro-1H-benzo[d]imidazole **35**

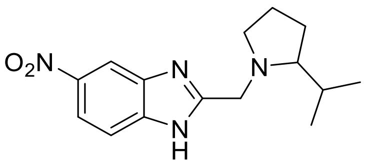

Initially a suspension of 2-(chloromethyl)-5-nitro-1H-benzo[d]imidazole **3** (0.5 g, 2.36 mmol, 1 eq) and $Na_2CO_3$ (0.301 g, 2.84 mmol, 2.5 eq) in anhydrous MeCN (7.2 mL, 0.33 M) had 2-isopropylpyrrolidine (0.321 g, 2.84 mmol, 1.2 eq) added at room temperature. The reaction was allowed to stir at room temperature overnight. Upon reaction completion the suspension was filtered through a sintered frit and washed with acetone. The filtrate was concentrated to a residue which was submitted to the next step without further purification **35** (*quant*).

**LR-ESI-MS**: $C_{15}H_{21}N_4O_2$ $[M+H]^+$ *m/z* found 289.23, cald 289.17.

5-nitro-2-((2-phenylpyrrolidin-1-yl)methyl)-1H-benzo[d]imidazole **36**

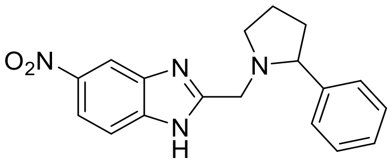


Initially a suspension of 2-(chloromethyl)-5-nitro-1H-benzo[d]imidazole **3** (1 g, 4.73 mmol, 1 eq) and $Na_2CO_3$ (0.601 g, 5.67 mmol, 2.5 eq) in anhydrous MeCN (14 mL, 0.33 M) had 2-phenylpyrrolidine (0.835 g, 5.67 mmol, 1.2 eq) added at room temperature. The reaction was allowed to stir at room temperature overnight. Upon reaction completion the suspension was filtered through a sintered frit and washed with acetone. The filtrate was concentrated to a residue which was submitted to the next step without further purification **36** (*quant*).

**LR-ESI-MS**: $C_{18}H_{19}N_4O_2$ $[M+H]^+$ *m/z* found 323.46, cald 323.15.

**General Procedure A: Reduction of nitro-benzimidazoles to anilino-benzimidazoles under hydrogenative conditions**

A stirred degassed solution of nitro-benzimidazole (1 eq) and Pd (10% on Carbon, 10% mass) in MeOH (0.15 M) was carefully evacuated and backfilled with $H_2$ atmosphere (1 bar) and finally allowed to stir at room temperature overnight with a $H_2$ balloon attached. Upon reaction completion the suspension was filtered through celite with MeOH washings. The filtrate was then concentrated to an oil before being purified via Biotage LPLC using a KP-Sil-NH column eluting with mixtures of CH:EA (10% MeOH) to give the corresponding anilines or purified using an SC-X ion exchange column eluting with mixtures of MeOH and $NH_3$ in MeOH to give anilines which were submitted to the next step without further purification.

2-(piperidin-1-ylmethyl)-1H-benzo[d]imidazol-5-amine **37**

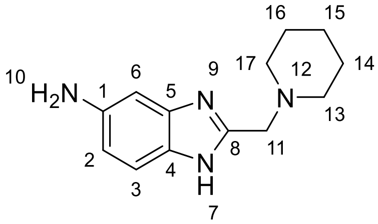


Synthesised according to general procedure **A** to give **37** (1.511 g, 6.56 mmol, 83 %) as a brown crystalline solid.

**Mpt:** 74.1-76.1 °C; **$v_{max}$ (cm$^{-1}$)** 2931, 1632, 1428, 1336, 1105, 805, 624, 432; **$^{1}$H NMR (400 MHz, DMSO-*d*$_6$) δ** 7.2 (d, *J* = 8.5 Hz, 1H, 3), 6.6 (d, *J* = 2.1 Hz, 1H, 6), 6.5 (dd, *J* = 2.1, 8.5 Hz, 1H, 2), 3.6 (s, 2H, 11), 2.4 (d, *J* = 5.5 Hz, 4H, 13, 17), 1.5 (q, *J* = 5.6 Hz, 4H, 14, 16), 1.4 – 1.3 (m, 2H, 15); **$^{13}$C NMR (101 MHz, DMSO-*d*$_6$) δ** 148.9 (1), 144.2 (3), 110.9 (2, 6), 56.4 (11), 54.0 (13, 17), 25.3 (14, 16), 23.6 (15); **LR-ESI-MS**: $C_{13}H_{19}N_4$ [M+H]$^+$ *m/z* found 231.21, cald 231.16; **HR-ESI-MS**: $C_{13}H_{19}N_4$ [M+H]$^+$ *m/z* found 231.1602, cald 231.1610.

2-((2-methylpiperidin-1-yl)methyl)-1H-benzo[d]imidazol-5-amine **38**

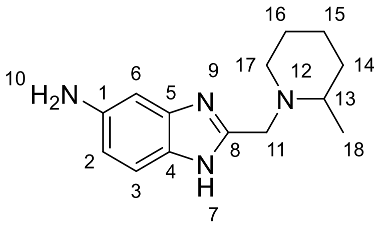


Synthesised according to general procedure **A** to give **38** (0.120 g, 0.492 mmol, 72 %) as an off white solid.

**Mpt:** 94.0-96.0 °C; **$v_{max}$ (cm$^{-1}$)** 2926, 2852, 1590, 1426, 1328, 1133, 1054, 804, 729; **$^{1}$H NMR (400 MHz, DMSO-*d*$_6$) δ** 7.1 (d, *J* = 8.4 Hz, 1H, 3), 6.6 (d, *J* = 2.0 Hz, 1H, 6), 6.4 (dd, *J* = 2.1, 8.5 Hz, 1H, 2), 3.9 (d, *J* = 14.3 Hz, 1H, 11''), 3.5 (d, *J* = 14.3 Hz, 1H, 11'), 2.7 (dt, *J* = 4.0, 11.8 Hz, 1H, 13), 2.4 – 2.3 (m, 1H, 17'), 2.1 (td, *J* = 3.2, 11.1 Hz, 1H, 17''), 1.6 (dd, *J* = 2.7, 9.1 Hz, 2H, 14', 16'), 1.5 – 1.3 (m, 2H, 14'', 16''), 1.2 (dtd, *J* = 1.9, 6.3, 7.4, 17.1 Hz, 2H, 15), 1.1 (d, *J* = 6.2 Hz, 3H, 18); **$^{13}$C NMR (101 MHz, DMSO-*d*$_6$) δ** 149.6 (30), 144.1 (2), 110.7 (6), 55.7 (11), 52.5 (13), 51.8 (17), 34.1 (14), 25.5 (16), 23.5 (15), 19.0 (18); **LR-ESI-MS**: $C_{14}H_{21}N_4$ [M+H]$^+$ *m/z* found 245.35, cald 245.18; **HR-ESI-MS**: $C_{14}H_{20}N_4Na$ [M+Na]$^+$ *m/z* found 267.1571, cald 267.1586.

2-((3-methylpiperidin-1-yl)methyl)-1H-benzo[d]imidazol-5-amine **39**

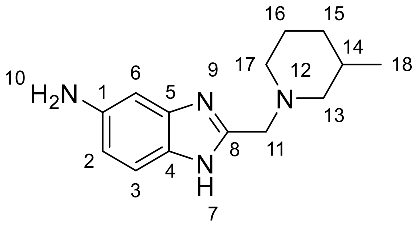


Synthesised according to general procedure **A** to give **39** (0.11 g, 0.450 mmol, 75 %) as an off white solid.

**Mpt:** 75.5-77.5 °C; **$v_{max}$ (cm$^{-1}$)** 2924, 1633, 1339, 1168, 804, 622; **$^{1}$H NMR (400 MHz, DMSO-*d*$_6$) δ** 7.1 (d, *J* = 8.4 Hz, 1H), 6.6 (d, *J* = 2.0 Hz, 1H), 6.4 (dd, *J* = 2.1, 8.5 Hz, 1H), 3.6 – 3.5 (m, 2H, 11), 2.8 – 2.7 (m, 2H, 13'', 17'), 1.9 (td, *J* = 2.9, 11.4 Hz, 1H, 13'), 1.7 – 1.4 (m, 5H, 15, 16, 17''), 0.8 (d, *J* = 5.8 Hz, 4H, 14, 18); **$^{13}$C NMR (101 MHz, DMSO-*d*$_6$) δ** 144.1 (3), 110.7 (2, 6), 61.4 (11), 56.4 (13), 53.6 (17), 32.4 (16), 30.6 (15), 25.0 (14), 19.6 (18); **LR-ESI-MS**: $C_{14}H_{21}N_4$ [M+H]$^+$ *m/z* found 245.34, cald 245.18; **HR-ESI-MS**: $C_{14}H_{20}N_4Na$ [M+Na]$^+$ *m/z* found 267.1574, cald 267.1586.

2-((4-methylpiperidin-1-yl)methyl)-1H-benzo[d]imidazol-5-amine **40**

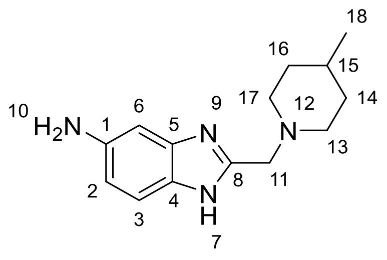


Synthesised according to general procedure **A** to give **40** (0.0492 g, 0.201 mmol, 58 %) as an off white solid.

**Mpt:** 74.6-76.6 °C; **$\nu_{max}$ (cm$^{-1}$)** 2916, 1632, 1332, 1254, 1112, 804, 624; **$^{1}$H NMR (400 MHz, DMSO-*d*$_6$) δ** 7.1 (d, *J* = 8.4 Hz, 1H), 6.6 (d, *J* = 2.2 Hz, 1H), 6.4 (dd, *J* = 2.1, 8.5 Hz, 1H), 3.6 (s, 2H, 11), 2.8 (dt, *J* = 3.3, 11.8 Hz, 2H, 13'', 17''), 2.0 (td, *J* = 2.4, 11.6 Hz, 2H, 13', 17'), 1.6 – 1.5 (m, 2H, 14'', 16''), 1.3 (tdd, *J* = 4.0, 7.0, 9.9 Hz, 1H, 15), 1.2 – 1.1 (m, 2H, 14', 16'), 0.9 (d, *J* = 6.5 Hz, 3H, 18); **$^{13}$C NMR (101 MHz, DMSO-*d*$_6$) δ** 144.1 (3), 110.7 (2, 6), 56.3 (11), 53.5 (13, 17), 33.8 (14, 16), 30.1 (15), 21.9 (18); **LR-ESI-MS**: $C_{14}H_{21}N_4$ [M+H]$^+$ *m/z* found 245.36, cald 245.18; **HR-ESI-MS**: $C_{14}H_{21}N_4$ [M+H]$^+$ *m/z* found 245.1756, cald 245.1766.

2-((1,2,4,5-tetrahydro-3H-benzo[d]azepin-3-yl)methyl)-1H-benzo[d]imidazol-5-amine **41**

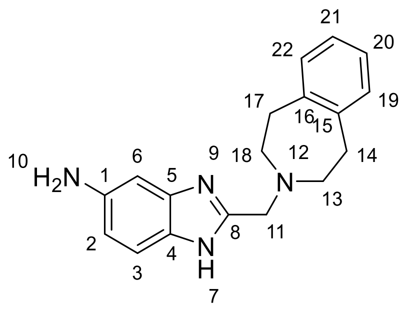


Synthesised according to general procedure **A** to give **41** (0.413 g, 1.413 mmol, 42 %) as an orange foam.

**Mpt:** 111.8-113.8 °C; **$\nu_{max}$ (cm$^{-1}$)** 2905, 2810, 1632, 1426, 749; **$^{1}$H NMR (400 MHz, DMSO-*d*$_6$) δ** 11.8 (d, *J* = 44.5 Hz, 1H, 7), 7.2 (d, *J* = 8.5 Hz, 1H, 3), 7.1 (d, *J* = 1.7 Hz, 5H, 19, 20, 21, 22), 6.8 – 6.6 (m, 1H, 6), 6.4 (dd, *J* = 2.1, 8.5 Hz, 1H, 2), 4.7 (d, *J* = 76.3 Hz, 2H, 10), 3.8 (d, *J* = 36.9 Hz, 2H, 11), 2.9 (dd, *J* = 3.5, 6.6 Hz, 4H, 13, 18), 2.7 – 2.5 (m, 5H, 14, 17); **$^{13}$C NMR (101 MHz, DMSO-*d*$_6$) δ** 149.0 (8), 144.4 (1), 141.9 (15, 16), 135.7 (5), 135.3 (4), 128.7 (20, 21), 126.1 (19, 22), 118.4 (3), 110.5 (2), 94.7 (6), 56.4 (11), 55.1 (13, 18), 35.7 (14, 17); **LR-ESI-MS**: $C_{18}H_{21}N_4$ [M+H]$^+$ *m/z* found 293.47, cald 293.18; **HR-ESI-MS**: $C_{18}H_{20}N_4Na$ [M+Na]$^+$ *m/z* found 315.1573, cald 315.1586.

2-((4-phenylpiperazin-1-yl)methyl)-1H-benzo[d]imidazol-5-amine **42**

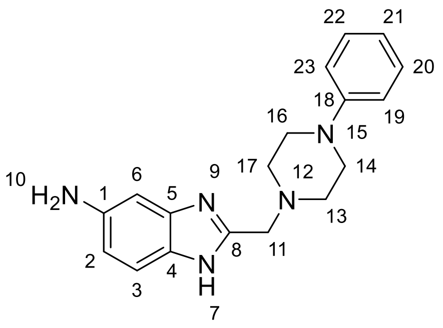


Synthesised according to general procedure **A** to give **42** (0.411 g, 1.337 mmol, 63 %) as an orange solid.

**Mpt:** 117.2-119.2 °C; **$\nu_{max}$ (cm$^{-1}$)** 2815, 1632, 1597, 1493, 1449, 1427, 1339, 1221, 1002, 807, 756, 690; **$^{1}$H NMR (400 MHz, DMSO-*d*$_6$) δ** 11.7 (s, 1H, 7), 7.3 – 7.1 (m, 3H, 3, 20, 22), 7.0 – 6.8 (m, 2H, 19, 23), 6.8 (td, *J* = 1.1, 7.2 Hz, 1H, 21), 6.6 (d, *J* = 2.1 Hz, 1H, 6), 6.4 (dd, *J* = 2.1, 8.5 Hz, 1H, 2), 4.7 (s, 2H), 3.6 (s, 2H, 11), 3.1 (dd, *J* = 3.6, 6.4 Hz, 4H, 13, 17), 2.6 (t, *J* = 4.9 Hz, 4H, 14, 16); **$^{13}$C NMR (101 MHz, DMSO-*d*$_6$) δ** 151.0 (8), 148.3 (1), 144.5 (18), 135.8 (5), 135.3 (4), 128.9 (20, 22), 118.8 (21), 118.4 (3), 115.4, 110.6 (6), 94.7 (2), 55.9 (11), 52.8 (13, 17), 48.1 (14, 16); **LR-ESI-MS**: $C_{18}H_{22}N_5$ $[M+H]^+$ *m/z* found 308.32, cald 308.19; **HR-ESI-MS**: $C_{18}H_{22}N_5$ $[M+H]^+$ *m/z* found 308.1860, cald 308.1875.

2-((4-(2-(pyrrolidin-1-yl)ethyl)piperidin-1-yl)methyl)-1H-benzo[d]imidazol-5-amine **43**

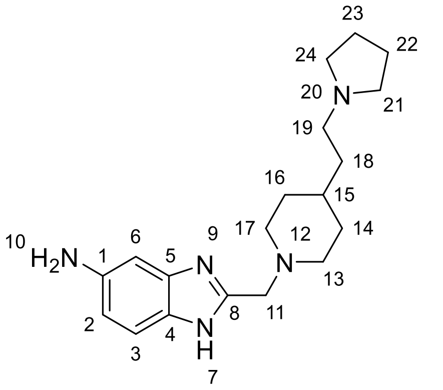


Synthesised according to general procedure **A** to give **43** (0.158 g, 0.482 mmol, 71 %) as an orange solid which was purified using an SC-X ion exchange column and submitted to the next step without further purification.

**LR-ESI-MS**: $C_{19}H_{30}N_5$ $[M+H]^+$ *m/z* found 328.61, cald 328.25.

2-((4-(2-(piperidin-1-yl)ethyl)piperidin-1-yl)methyl)-1H-benzo[d]imidazol-5-amine **44**

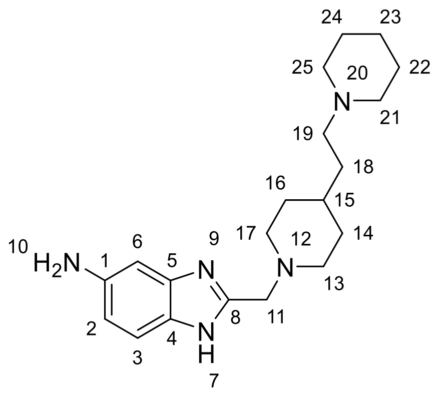

Synthesised according to general procedure **A** to give **44** (0.194 g, 0.568 mmol, 36 %) which was purified using an SC-X ion exchange column and submitted to the next step without further purification.

**LR-ESI-MS**: $C_{20}H_{32}N_5$ $[M+H]^+$ *m/z* found 343.63, cald 342.27.

2-((cis-2,6-dimethylpiperidin-1-yl)methyl)-1H-benzo[d]imidazol-5-amine **45**

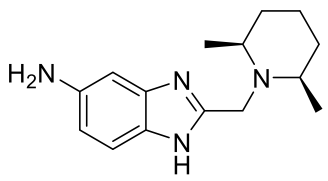

Synthesised according to general procedure **A** to give **45** (0.149 g, 0.578 mmol, 33 %) as an orange solid which was purified using an SC-X ion exchange column and submitted to the next step without further purification.

**LR-ESI-MS**: $C_{15}H_{23}N_4$ $[M+H]^+$ *m/z* found 259.47, cald 259.19.

2-((4-methylpiperazin-1-yl)methyl)-1H-benzo[d]imidazol-5-amine **46**

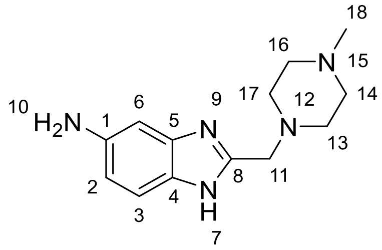

Synthesised according to general procedure **A** to give **46** (0.243 g, 0.991 mmol, 64 %) as an off white solid.

**Mpt:** 64.8-66.8 °C; **$\nu_{max}$ (cm$^{-1}$)** 2798, 1631, 1452, 1429, 1343, 1161, 1135, 816, 622; **$^1$H NMR (400 MHz, DMSO-*d*$_6$) δ** 11.7 (d, *J* = 44.0 Hz, 1H, 7), 7.1 (dd, *J* = 8.4, 35.7 Hz, 1H, 3), 6.7 – 6.5 (m, 1H, 6), 6.5 – 6.4 (m, 1H, 2), 4.7 (d, *J* = 78.0 Hz, 2H, 10), 3.6 (d, *J* = 8.2 Hz, 2H, 11), 2.5 – 2.2 (m, 8H, 13, 14, 16, 17), 2.1 (d, *J* = 2.5 Hz, 3H, 18); **$^{13}$C NMR (101 MHz, DMSO-*d*$_6$) δ** 148.5 (8), 144.4 (1), 135.7 (5), 135.2 (4), 118.4 (3), 110.5 (2), 94.7 (6), 55.9 (d, *J* = 9.1 Hz, 11), 54.6 (13, 17), 52.8 (14, 16), 45.8 (18); **LR-ESI-MS**: $C_{13}H_{20}N_5$ $[M+H]^+$ *m/z*

found 246.44, cald 246.17; **HR-ESI-MS**: $C_{13}H_{19}N_5Na$ $[M+Na]^+$ *m/z* found 268.1528, cald 268.1538.

2-((dimethylamino)methyl)-1H-benzo[d]imidazol-5-amine **47**

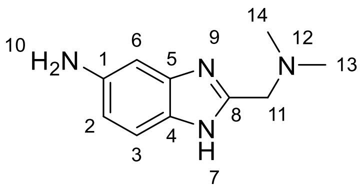


Synthesised according to general procedure **A** to give **47** (0.129 g, 0.678 mmol, 20 %) as a red oil.

**$v_{max}$ (cm$^{-1}$)** 2773, 1632, 1435, 1345, 1170, 805, 622; **$^1$H NMR (400 MHz, DMSO-*d*$_6$) δ** 11.9 – 11.6 (m, 1H, 7), 7.3 – 7.0 (m, 1H, 3), 6.8 – 6.5 (m, 1H, 6), 6.5 – 6.3 (m, 1H, 2), 5.0 – 4.5 (m, 2H, 10), 3.6 – 3.4 (m, 2H, 11), 2.2 (s, 6H, 13, 14); **$^{13}$C NMR (101 MHz, DMSO-*d*$_6$) δ** 149.6 (8), 144.8 (1), 136.2 (5), 135.7 (4), 118.8 (3), 110.9 (2), 95.2 (6), 57.6 (11), 45.6 (13, 14); **LR-ESI-MS**: $C_{10}H_{15}N_4$ $[M+H]^+$ *m/z* found 191.35, cald 191.13; **HR-ESI-MS**: $C_{10}H_{14}N_4Na$ $[M+Na]^+$ *m/z* found 213.1108, cald 213.1116.

2-((cis-3,5-dimethylpiperidin-1-yl)methyl)-1H-benzo[d]imidazol-5-amine **48**

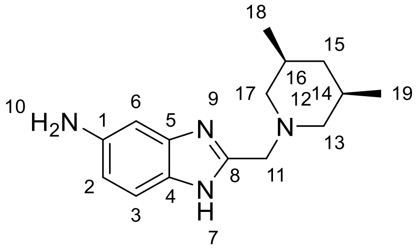


Synthesised according to general procedure **A** to give **48** (0.553 g, 2.14 mmol, 62 %) as an off white solid.

**Mpt:** 92.5-94.5 °C; **$v_{max}$ (cm$^{-1}$)** 2794, 1631, 1426, 1342, 1162, 804; **$^1$H NMR (400 MHz, DMSO-*d*$_6$) δ** 7.1 (d, *J* = 8.5 Hz, 1H, 3), 6.6 (d, *J* = 2.1 Hz, 1H, 6), 6.4 (dd, *J* = 2.1, 8.4 Hz, 1H, 2), 3.6 (s, 2H, 11'), 2.8 – 2.7 (m, 2H, 13'', 17''), 1.6 (tdq, *J* = 2.7, 3.4, 6.7, 10.1 Hz, 3H, 13', 15'', 17'), 1.5 (t, *J* = 10.8 Hz, 2H, 14, 16), 0.8 (d, *J* = 6.3 Hz, 6H, 18, 19), 0.5 (td, *J* = 11.4, 13.1 Hz, 1H, 15'); **$^{13}$C NMR (101 MHz, DMSO-*d*$_6$) δ** 149.2 (3), 144.2 (6), 110.8 (2), 61.0 (13, 17), 56.1 (11), 41.6 (15), 30.5 (14, 16), 19.5 (18, 19); **LR-ESI-MS**: $C_{15}H_{23}N_4$ $[M+H]^+$ *m/z* found 259.47, cald 259.19; **HR-ESI-MS**: $C_{15}H_{22}N_4Na$ $[M+Na]^+$ *m/z* found 281.1732, cald 281.1742.

2-(1-(piperidin-1-yl)ethyl)-1H-benzo[d]imidazol-5-amine **49**

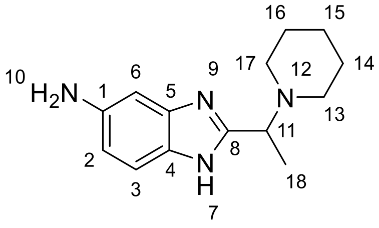

Synthesised according to general procedure **A** to give **49** (0.314 g, 1.29 mmol, 88 %) as a white solid.

**Mpt:** 108.8-110.8 °C; **$v_{max}$ ($cm^{-1}$)** 2923, 1633, 1452, 1308, 1220, 1166, 1056, 623; **$^{1}H$ NMR (400 MHz, DMSO-*d*$_6$) δ** 11.5 (d, *J* = 43.0 Hz, 1H, 7), 7.1 (dd, *J* = 8.4, 35.2 Hz, 1H, 6), 6.8 – 6.6 (m, 1H, 2), 6.5 – 6.3 (m, 1H, 3), 4.9 – 4.5 (m, 2H, 10), 3.7 (q, *J* = 6.8 Hz, 1H, 11), 2.4 (t, *J* = 5.4 Hz, 4H, 13, 17), 1.5 (p, *J* = 5.5 Hz, 4H, 14, 16), 1.3 (t, *J* = 7.4 Hz, 5H, 15, 18); **$^{13}C$ NMR (101 MHz, DMSO-*d*$_6$) δ** 152.5 (8), 144.2 (1), 135.5 (5), 135.1 (4), 118.4 (3), 110.3 (2), 94.8 (6), 64.9 (11), 58.5 (13), 50.2 (17), 25.8 (14, 16), 24.2 (15), 14.6 (18); **LR-ESI-MS**: $C_{14}H_{21}N_4$ $[M+H]^+$ *m/z* found 245.39, cald 245.18; **HR-ESI-MS**: $C_{14}H_{20}N_4Na$ $[M+Na]^+$ *m/z* found 267.1578, cald 267.1586.

2-(2-(piperidin-1-yl)ethyl)-1H-benzo[d]imidazol-5-amine **50**

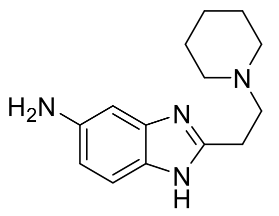

Synthesised according to general procedure **A** to give **50** (0.018 g, 0.074 mmol, 20 %) as an unstable solid which was immediately used in the following step.

**LR-ESI-MS**: $C_{14}H_{21}N_4$ $[M+H]^+$ *m/z* found 245.43, cald 245.18.

(*R*)-2-((2-methylpyrrolidin-1-yl)methyl)-1H-benzo[d]imidazol-5-amine **51**

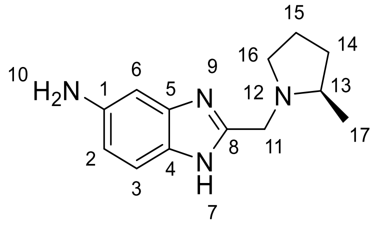

Synthesised according to general procedure **A** to give **51** (0.298 g, 1.297 mmol, 46 %) as a dark green solid.

**Mpt:** 92.4-94.4 °C; **$v_{max}$ ($cm^{-1}$)** 2958, 2794, 1632, 1426, 1165, 803, 622; **$^{1}H$ NMR (400 MHz, DMSO-*d*$_6$) δ** 11.7 (d, *J* = 44.8 Hz, 1H), 7.1 (dd, *J* = 8.5, 34.7 Hz, 1H, 3), 6.8 – 6.5 (m, 1H, 6), 6.5 – 6.4 (m, 1H, 2), 4.8 (s, 2H, 10), 3.9 (d, *J* = 13.9 Hz, 1H, 11''), 3.4 (d, *J* = 13.8 Hz, 1H, 11'), 2.9 (td, *J* = 3.2, 7.8, 8.6 Hz, 1H, 16''), 2.4 (q, *J* = 7.0 Hz, 1H, 16'), 2.2 (q, *J* = 8.8 Hz, 1H, 13), 1.9 (dddd, *J* = 5.7, 7.3, 9.4, 12.5 Hz, 1H, 14''), 1.7 – 1.5 (m, 2H, 14', 15''), 1.4 – 1.2 (m, 1H, 15'), 1.1 (d, *J* = 6.0 Hz, 3H, 17); **$^{13}C$ NMR (101 MHz, DMSO-*d*$_6$) δ** 149.7 (1), 144.3 (8), 135.7 (5), 135.3 (4), 118.3 (3), 110.4 (2), 94.8 (6), 58.8 (11), 54.0 (16), 50.9 (13), 32.5 (14), 21.3 (15), 18.9 (17); **LR-ESI-MS**: $C_{13}H_{19}N_4$ $[M+H]^+$ *m/z* found 231.30, cald 231.16; **HR-ESI-MS**: $C_{13}H_{18}N_4Na$ $[M+Na]^+$ *m/z* found 253.1429, cald 253.1418.

2-((3-methylpyrrolidin-1-yl)methyl)-1H-benzo[d]imidazol-5-amine **52**

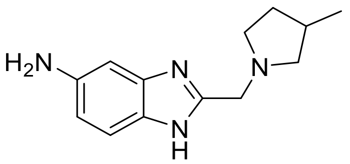

Synthesised according to general procedure **A** to give **52** (0.027 g, 0.117 mmol, 74 %) as a yellow oil which was purified using an SC-X ion exchange column and submitted to the next step without further purification.

**LR-ESI-MS**: $C_{13}H_{19}N_4$ $[M+H]^+$ *m/z* found 231.39, cald 231.16.

2-(((3*R*, 4*R*)-3,4-dimethylpyrrolidin-1-yl)methyl)-1H-benzo[d]imidazol-5-amine **53**

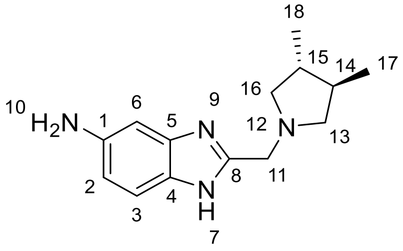

Synthesised according to general procedure **A** to give **53** (0.186 g, 0.762 mmol, 54 %) as a yellow oil.

**$v_{max}$ (cm$^{-1}$)** 2951, 1633, 1450, 1427, 1169, 804, 623; **$^1$H NMR (400 MHz, DMSO-*d*$_6$) δ** 11.9 – 11.5 (m, 1H, 7), 7.2 – 7.0 (m, 1H, 6), 6.8 – 6.5 (m, 1H, 3), 6.5 – 6.4 (m, 1H, 2), 4.8 (s, 2H, 10), 3.8 – 3.6 (m, 2H, 11), 2.8 (dd, *J* = 6.9, 8.8 Hz, 2H, 13'', 16'), 2.2 (dd, *J* = 6.6, 8.9 Hz, 2H, 13', 16''), 1.7 – 1.6 (m, 2H, 14, 15), 1.0 – 0.9 (m, 6H, 17, 18); **$^{13}$C NMR (101 MHz, DMSO-*d*$_6$) δ** 149.5 (8), 144.3 (1), 135.7 (5), 135.2 (4), 118.4 (3), 110.5 (2), 94.8 (6), 61.7 (13, 16), 53.6 (11), 40.4 (14, 15), 18.4 (17, 18); **LR-ESI-MS**: $C_{14}H_{21}N_4$ $[M+H]^+$ *m/z* found 245.39, cald 245.18; **HR-ESI-MS**: $C_{14}H_{20}N_4Na$ $[M+Na]^+$ *m/z* found 267.1575, cald 267.1586.

2-((2-methylpyrrolidin-1-yl)methyl)-1H-benzo[d]imidazol-5-amine **54**

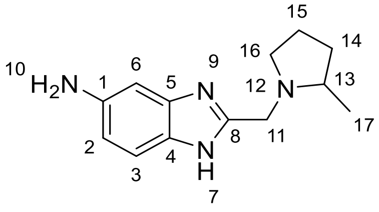

Synthesised according to general procedure **A** to give **54** (0.264 g, 1.15 mmol, 78 %) as a brown oil.

**$v_{max}$ (cm$^{-1}$)** 2958, 2793, 1632, 1426, 1165, 804, 622; **$^1$H NMR (400 MHz, DMSO-*d*$_6$) δ** 11.7 (s, 1H, 7), 7.2 (d, *J* = 8.4 Hz, 1H, 6), 6.6 (s, 1H, 3), 6.5 (d, *J* = 8.6 Hz, 1H, 2), 5.2 – 4.4 (m, 2H, 10), 3.9 (d, *J* = 13.9 Hz, 1H, 11''), 3.4 – 3.3 (m, 1H, 11'), 2.9 (ddd, *J* = 3.1, 7.7, 9.9 Hz, 1H, 13), 2.5 – 2.3 (m, 1H, 16'), 2.2 (q, *J* = 8.8 Hz, 1H, 16''), 1.9 (dddd, *J* = 3.5, 5.2, 9.4, 12.5 Hz, 1H, 14''), 1.7 – 1.5 (m, 2H, 14', 15''), 1.3 (dddd, *J* = 6.2, 8.4, 10.1, 12.2 Hz, 1H, 15'), 1.1 (d, *J* = 6.0 Hz, 3H, 17); **$^{13}$C NMR (101 MHz, DMSO-*d*$_6$) δ** 149.8 (8), 144.3 (1), 135.7 (5), 135.3 (4), 118.3 (3), 110.5 (2), 94.9 (6), 58.9 (11), 54.0 (13), 50.9 (16), 32.6 (14), 21.4 (15),

18.9 (17); **LR-ESI-MS**: $C_{13}H_{19}N_4$ $[M+H]^+$ *m/z* found 231.40, calds 231.16; **HR-ESI-MS**: $C_{13}H_{18}N_4Na$ $[M+Na]^+$ *m/z* found 253.1419, cald 253.1429.

(*R*)-2-((3-methylpyrrolidin-1-yl)methyl)-1H-benzo[d]imidazol-5-amine **55**

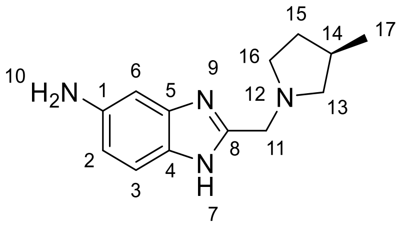


Synthesised according to general procedure **A** to give **55** (0.233 g, 1.01 mmol, 66 %) as an off white solid.

**Mpt:** 143.2-145.2 °C; $\mathbf{v_{max}}$ **(cm$^{-1}$)** 2784, 1629, 1427, 1024, 798, 622; **$^1$H NMR (400 MHz, DMSO-*d*$_6$) δ** 11.7 (s, 1H, 7), 7.2 (d, *J* = 8.4 Hz, 1H, 3), 6.7 – 6.5 (m, 1H, 6), 6.4 (dd, *J* = 2.1, 8.5 Hz, 1H, 2), 4.8 (s, 2H, 10), 3.9 – 3.5 (m, 2H, 11''), 2.9 – 2.7 (m, 1H, 13''), 2.6 (td, *J* = 5.7, 8.4 Hz, 1H, 16'), 2.5 (s, 1H, 16''), 2.3 – 2.1 (m, 1H, 13'), 2.1 – 1.9 (m, 2H, 15), 1.4 – 1.2 (m, 1H, 14), 1.0 (d, *J* = 6.7 Hz, 3H, 17); **$^{13}$C NMR (101 MHz, DMSO-*d*$_6$) δ** 149.5 (8), 144.3 (1), 135.7 (5), 135.2 (4), 118.3 (3), 110.4 (2), 94.7 (6), 61.7 (11), 53.6 (13), 53.4 (16), 32.4 (15), 31.5 (14), 20.4 (17); **LR-ESI-MS**: $C_{13}H_{19}N_4$ $[M+H]^+$ *m/z* found 231.40, cald 231.16; **HR-ESI-MS**: $C_{13}H_{18}N_4Na$ $[M+Na]^+$ *m/z* found 253.1421, cald 253.1429.

(*S*)-2-((3-methylpyrrolidin-1-yl)methyl)-1H-benzo[d]imidazol-5-amine **56**

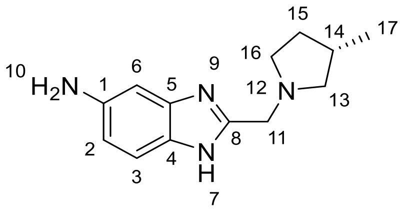


Synthesised according to general procedure **A** to give **56** (0.061 g, 0.263 mmol, 23 %) as a tan oil.

$\mathbf{v_{max}}$ **(cm$^{-1}$)** 2952, 1632, 1428, 805, 625; **$^1$H NMR (400 MHz, DMSO-*d*$_6$) δ** 11.9 – 11.4 (m, 1H), 7.3 – 6.9 (m, 1H, 6), 6.8 – 6.5 (m, 1H, 3), 6.5 – 6.4 (m, 1H, 2), 4.8 (s, 2H, 10), 3.7 (s, 2H, 11'), 2.8 (dd, *J* = 7.3, 8.8 Hz, 1H, 13'), 2.6 (td, *J* = 5.8, 8.4 Hz, 1H, 16'), 2.5 – 2.4 (m, 1H, 13''), 2.2 (ddt, *J* = 6.7, 9.1, 13.5 Hz, 1H, 16''), 2.0 (dd, *J* = 6.9, 8.8 Hz, 1H, 15'), 2.0 – 1.9 (m, 1H, 14), 1.3 (ddt, *J* = 6.0, 8.4, 12.2 Hz, 1H, 15''), 1.0 (d, *J* = 6.7 Hz, 3H, 17); **$^{13}$C NMR (101 MHz, DMSO-*d*$_6$) δ** 149.5 (8), 144.3 (1), 135.7 (5), 135.2 (4), 118.3 (3), 110.5 (2), 94.8 (6), 61.7 (13), 53.6 (16), 53.4 (11), 32.4 (14), 31.5 (15), 20.4 (17); **LR-ESI-MS**: $C_{13}H_{19}N_4$ $[M+H]^+$ *m/z* found 231.27, cald 231.16; **HR-ESI-MS**: $C_{13}H_{19}N_4$ $[M+H]^+$ *m/z* found 231.1602, cald 231.1610.

2-((3-azabicyclo[3.1.0]hexan-3-yl)methyl)-1H-benzo[d]imidazol-5-amine **57**

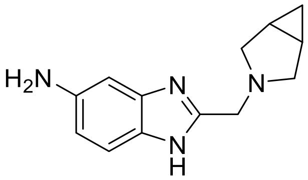

Synthesised according to general procedure **A** to give **57** (0.464 g, 2.03 mmol, 29 %) as a tan solid which was purified using an SC-X ion exchange column and submitted to the next step without further purification.

**LR-ESI-MS**: $C_{13}H_{17}N_4$ $[M+H]^+$ *m/z* found 229.35, cald 229.15.

2-((3,3-difluoropyrrolidin-1-yl)methyl)-1H-benzo[d]imidazol-5-amine **58**

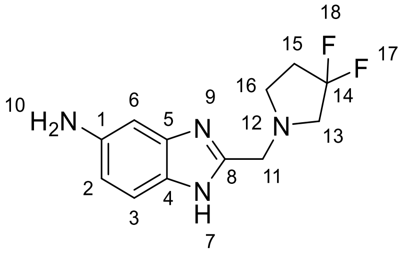

Synthesised according to general procedure **A** to give **58** (0.257 g, 1.02 mmol, 35 %) as a brown solid which was purified using an SC-X ion exchange column and submitted to the next step without further purification.

**LR-ESI-MS**: $C_{12}H_{15}F_2N_4$ $[M+H]^+$ *m/z* found 253.32, cald 253.13.

(*S*)-2-((2-methylpyrrolidin-1-yl)methyl)-1H-benzo[d]imidazol-5-amine **59**

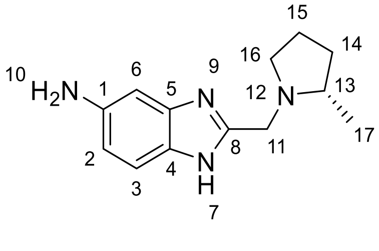

Synthesised according to general procedure **A** to give **59** (0.320 g, 1.39 mmol, 20 %) as a beige solid which was purified using an SC-X ion exchange column and submitted to the next step without further purification.

**LR-ESI-MS**: $C_{13}H_{19}N_4$ $[M+H]^+$ *m/z* found 231.35, cald 231.16.

2-((3-methylpyrrolidin-1-yl)methyl)-1H-benzo[d]imidazol-5-amine **60**

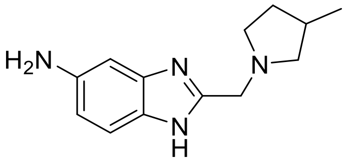

Synthesised according to general procedure **A** to give **60** (0.270 g, 1.17 mmol, 17 %) as a tan solid which was purified using an SC-X ion exchange column and submitted to the next step without further purification.

**LR-ESI-MS**: $C_{13}H_{19}N_4$ $[M+H]^+$ *m/z* found 231.34, cald 231.16.

2-((2,2-dimethylpyrrolidin-1-yl)methyl)-1H-benzo[d]imidazol-5-amine **61**

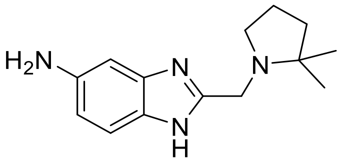


Synthesised according to general procedure **A** to give **61** (0.654 g, 2.68 mmol, 44 %) as a beige solid which was purified using an SC-X ion exchange column and submitted to the next step without further purification.

**LR-ESI-MS**: $C_{14}H_{21}N_4$ $[M+H]^+$ *m/z* found 245.43, cald 245.18.

2-((2-ethylpyrrolidin-1-yl)methyl)-1H-benzo[d]imidazol-5-amine **62**

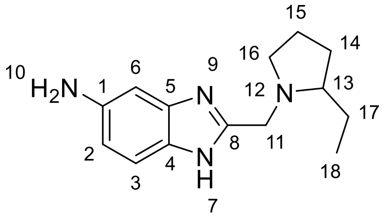


Synthesised according to general procedure **A** to give **62** (0.507 g, 2.08 mmol, 34 %) as a tan solid which was purified using an SC-X ion exchange column and submitted to the next step without further purification.

**LR-ESI-MS**: $C_{14}H_{21}N_4$ $[M+H]^+$ *m/z* found 245.43, cald 245.18.

2-(((1*S*, 4*S*)-2-oxa-5-azabicyclo[2.2.1]heptan-5-yl)methyl)-1H-benzo[d]imidazol-5-amine **63**

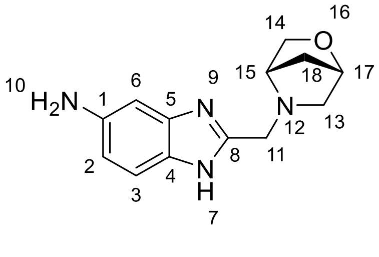


Synthesised according to general procedure **A** to give **63** (0.264 g, 1.08 mmol, 18 %) as a yellow solid which was purified using an SC-X ion exchange column and submitted to the next step without further purification.

**LR-ESI-MS**: $C_{13}H_{17}N_4O$ $[M+H]^+$ *m/z* found 245.39, cald 245.14.

tert-butyl (2-(((5-amino-1H-benzo[d]imidazol-2-yl)methyl)(methyl)amino)ethyl)carbamate **64**

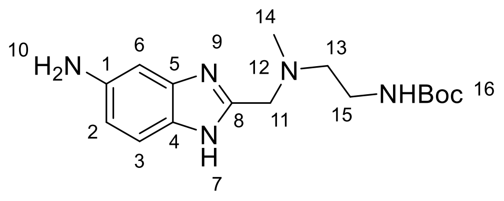


Synthesised according to general procedure **A** to give **64** (0.199 g, 0.622 mmol, 26 %) as a dark brown solid.

**Mpt:** 88.1-90.1 °C; **$v_{max}$ ($cm^{-1}$)** 3204, 2973, 1687, 1496, 1163, 806, 623; **$^1$H NMR (400 MHz, DMSO-*d*$_6$) δ** 11.7 (m, 1H, 7), 7.1 (m, 1H, 3), 6.8 (t, *J* = 5.7 Hz, 1H, N<u>H</u>Boc), 6.6 (m, 1H, 2), 6.4 (m, 1H, 6), 4.7 (m, 2H, 10), 3.6 (m, 2H, 11), 3.1 (q, *J* = 6.4 Hz, 2H, 13), 2.4 (t, *J* = 6.7 Hz, 2H, 15), 2.2 (s, 3H, 14), 1.4 (s, 9H, 16); **$^{13}$C NMR (101 MHz, DMSO-*d*$_6$) δ** 155.6 (-NH<u>C</u>O-$_2$tBu), 149.3 (8), 144.4 (1), 135.6 (5), 135.3 (4), 118.4 (3), 110.4 (2), 94.7 (6), 77.6 (-O-<u>C</u>(CH-$_3$)$_3$), 56.4 (11), 55.2 (13), 42.3 (14), 38.0 (15), 28.3 (-O-C(<u>C</u>H$_3$)$_3$); **LR-ESI-MS**: $C_{16}H_{26}N_5O$ $[M+H]^+$ *m/z* found 320.49, cald 320.21; **HR-ESI-MS**: $C_{16}H_{26}N_5O$ $[M+H]^+$ *m/z* found unstable Boc group failed to give identifiable [M], cald 320.2087.

2-((2-(methoxymethyl)pyrrolidin-1-yl)methyl)-1H-benzo[d]imidazol-5-amine **65**

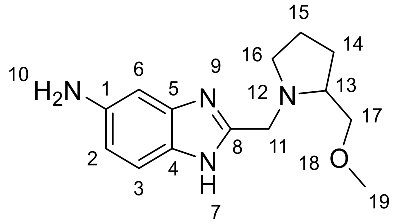


Synthesised according to general procedure **A** to give **65** (0.207 g, 0.795 mmol, 34 %) as a black waxy solid.

**$v_{max}$ ($cm^{-1}$)** 3195, 2871, 1632, 1426, 1093, 804, 624; **$^1$H NMR (400 MHz, DMSO-*d*$_6$) δ** 11.6 (s, 1H, 7), 7.2 – 7.0 (m, 1H, 3), 6.8 – 6.5 (m, 1H, 6), 6.5 – 6.3 (m, 1H, 2), 5.0 – 4.5 (m, 2H, 10), 4.1 – 4.0 (m, 1H, 11''), 3.6 – 3.5 (m, 1H, 11'), 3.4 – 3.3 (m, 1H, 17''), 3.2 (s, 4H, 17', 19), 2.9 (ddd, *J* = 3.6, 5.9, 9.4 Hz, 1H, 13), 2.8 (dt, *J* = 5.8, 8.3 Hz, 1H, 16''), 2.3 (td, *J* = 7.7, 9.1 Hz, 1H, 16'), 1.8 (dq, *J* = 8.2, 12.2 Hz, 1H, 14''), 1.6 (tt, *J* = 5.9, 8.4 Hz, 2H, 14', 15''), 1.6 – 1.4 (m, 1H, 15'); **$^{13}$C NMR (101 MHz, DMSO-*d*$_6$) δ** 149.7 (8), 144.3 (1), 135.6 (5), 135.3 (4), 118.3 (3), 110.5 (2), 94.8 (6), 75.8 (17), 62.0 (19), 58.4, 54.3 (13), 52.2 (16), 28.4 (14), 22.6 (15); **LR-ESI-MS**: $C_{14}H_{21}N_4O$ $[M+H]^+$ *m/z* found 261.45, cald 261.17; **HR-ESI-MS**: $C_{14}H_{20}N_4NaO$ $[M+Na]^+$ *m/z* found 283.1528, cald 283.1535.

2-((2-isopropylpyrrolidin-1-yl)methyl)-1H-benzo[d]imidazol-5-amine **66**

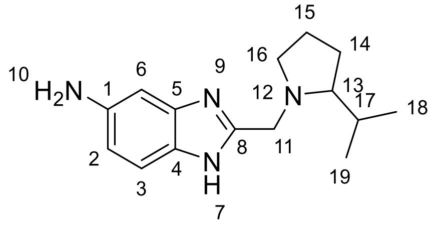

Synthesised according to general procedure **A** to give **66** (0.148 g, 0.576 mmol, 24 %) as a tan solid which was purified using an SC-X ion exchange column and submitted to the next step without further purification.

**LR-ESI-MS**: $C_{15}H_{23}N_4$ $[M+H]^+$ *m/z* found 259.43, cald 259.19.

2-((2-isopropylpyrrolidin-1-yl)methyl)-1H-benzo[d]imidazol-5-amine **67**

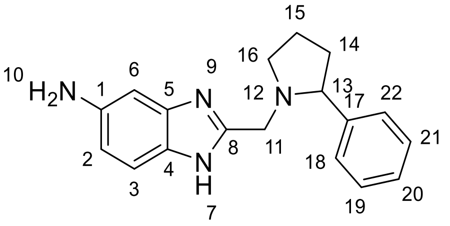

Synthesised according to general procedure **A** to give **67** which was used without further purification (0.148 g, 0.576 mmol, 24 %) as a tan foam.

**LR-ESI-MS**: $C_{18}H_{21}N_4$ $[M+H]^+$ *m/z* found 293.42, cald 293.18.

**General Procedure B: Amide Synthesis**

To a stirred solution of the corresponding anilines (1 eq) in $CH_2Cl_2$ (0.1 M, anhydrous) was added DIPEA-PS (200-400 mesh, 40% loading) (2 eq), the corresponding acid (1.2 eq) and HATU (1.2 eq). The suspension was stirred vigorously for 16 h before being concentrated onto silica gel and purified via Biotage LPLC using a mixture of DCM:DCM (20% MeOH) or CH:EA (10% MeOH). To give the products which were triturated to form solids with $Et_2O$ or CH.

**General Procedure C: Sulfonamide/Amide Synthesis**

To a stirred solution of the corresponding anilines (1 eq) in $CH_2Cl_2$ (0.1 M, anhydrous) was added DIPEA-PS (200-400 mesh, 40% loading) (3 eq) and the corresponding acid/sulfonyl chloride (1.5 eq). The suspension was stirred vigorously for 16 h before being concentrated onto silica gel and purified via Biotage LPLC using a mixture of DCM:DCM (20% MeOH) or CH:EA (10% MeOH). To give the products which were triturated to form solids with $Et_2O$ or CH.

2-chloro-*N*-(2-(piperidin-1-ylmethyl)-1H-benzo[d]imidazol-5-yl)benzamide **68**

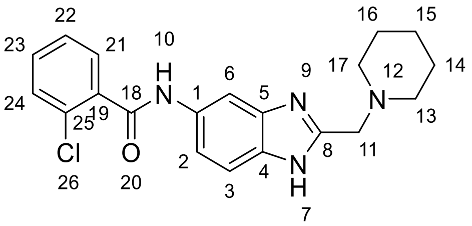

Synthesised according to general procedure **C** to give **68** (0.058 g, 0.156 mmol, 72 %) as a white solid.

**Mpt:** 128.9-130.9 °C; **$\nu_{max}$ (cm$^{-1}$)** 2932, 1531, 1450, 1251, 1109,1037, 859, 807, 652; **$^1$H NMR (400 MHz, DMSO-*d*$_6$) δ** 12.2 (s, 1H, 10), 10.5 (s, 1H, 7), 8.0 (d, *J* = 42.8 Hz, 1H, 21),

7.7 – 7.1 (m, 6H, 2, 3, 6, 22, 23, 24), 3.6 (s, 2H, 11), 2.4 (d, *J* = 5.5 Hz, 4H, 13, 17), 1.5 (p, *J* = 5.5 Hz, 4H, 14, 16), 1.4 (q, *J* = 5.8 Hz, 2H, 15); **$^{13}$C NMR (101 MHz, DMSO-*d*$_6$) δ** 164.6 (18), 137.3 (25), 130.9 (19), 130.0 (24), 129.6 (21), 129.0 (22, 23), 127.2 (2, 6), 56.6 (11), 54.1 (13, 17), 25.5 (14, 16), 23.8 (15); **LR-ESI-MS**: $C_{20}H_{22}ClN_4O$ $[M+H]^+$ *m/z* found 369.34, cald 369.15; **HR-ESI-MS**: $C_{20}H_{22}ClN_4O$ $[M+H]^+$ *m/z* found 369.1463, cald 369.1482.

2-methoxy-*N*-(2-(piperidin-1-ylmethyl)-1H-benzo[d]imidazol-5-yl)benzamide **69**

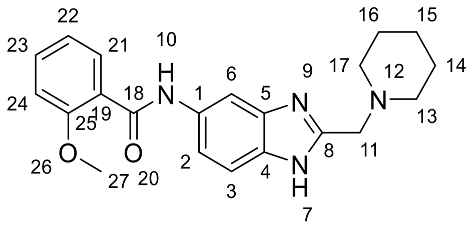


Synthesised according to general procedure **C** to give **69** (0.029 g, 0.080 mmol, 37 %) as a yellow solid.

**Mpt:** 111.7-113.7 °C; **$v_{max}$ (cm$^{-1}$)** 3356, 2936, 2359, 1661, 1599, 1249, 1020, 755; **$^1$H NMR (400 MHz, DMSO-*d*$_6$) δ** 12.2 (s, 1H, 10), 10.1 (s, 1H, 7), 8.1 (s, 1H, 21), 7.7 (dd, *J* = 1.9, 7.6 Hz, 1H, 3), 7.6 – 7.1 (m, 4H, 2, 6, 23, 24), 7.1 – 6.9 (m, 1H, 22), 3.9 (s, 3H, 27), 3.6 (s, 2H, 11), 2.4 (t, *J* = 5.4 Hz, 4H, 13, 17), 1.5 (p, *J* = 5.5 Hz, 5H, 14, 16), 1.4 (q, *J* = 5.3, 6.0 Hz, 2H, 15); **$^{13}$C NMR (101 MHz, DMSO-*d*$_6$) δ** 156.5 (18), 131.9 (21, 23), 129.7 (5), 120.5 (22), 120.0 (3), 112.3, 112.0 (24), 56.6 (11), 55.9 (27), 54.1 (13, 17), 25.5 (14, 16), 23.8 (15); **LR-ESI-MS**: $C_{21}H_{25}N_4O_2$ $[M+H]^+$ *m/z* found 365.33, cald 365.20; **HR-ESI-MS**: $C_{21}H_{25}N_4O_2$ $[M+H]^+$ *m/z* found 365.1965, cald 365.1978.

*N*-(2-(piperidin-1-ylmethyl)-1*H*-benzo[*d*]imidazol-5-yl)-3-(trifluoromethoxy)benzamide **70**

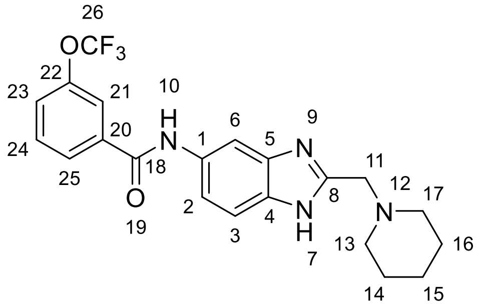


Synthesised according to general procedure **B** to give **70** (0.033 g, 0.079 mmol, 36 %) as an off white solid.

**Mpt:** 111.6-113.6 °C; **$v_{max}$ (cm$^{-1}$)** 2934, 1647, 1531, 1451, 804, 705; **$^{19}$F NMR (376 MHz, DMSO-*d*$_6$) δ** -56.7; **$^1$H NMR (400 MHz, DMSO-*d*$_6$) δ** 12.3 (s, 1H, 7), 10.4 (s, 1H, 10), 8.1 – 8.0 (m, 2H, 21, 25), 7.9 (p, *J* = 1.2 Hz, 1H, 6), 7.7 (t, *J* = 8.0 Hz, 1H, 24), 7.6 (ddt, *J* = 1.1, 2.4, 8.1 Hz, 1H, 23), 7.5 (d, *J* = 4.7 Hz, 2H, 2, 3), 3.7 (s, 2H, 11), 2.4 (t, *J* = 5.3 Hz, 4H, 13, 17), 1.5 (p, *J* = 5.5 Hz, 4H, 14, 16), 1.4 (q, *J* = 5.8 Hz, 2H, 15); **$^{13}$C NMR (101 MHz, DMSO-*d*$_6$) δ** 163.5 (18), 148.8 – 148.0 (m, 22), 137.4 (20), 130.6 (23), 126.8 (24), 123.9 (21), 121.4 (2), 120.2 (25), 118.8 (3), 56.6 (11), 54.1 (13, 17), 25.4 (14, 16), 23.7 (15); **LR-ESI-MS**: $C_{21}H_{22}F_3N_4O_2$ $[M+H]^+$ *m/z* found 419.62, cald 419.17; **HR-ESI-MS**: $C_{21}H_{22}F_3N_4O_2$ $[M+H]^+$ *m/z* found 419.1670, cald 419.1695.

3,4-dichloro-*N*-(2-(piperidin-1-ylmethyl)-1H-benzo[d]imidazol-5-yl)benzamide **71**

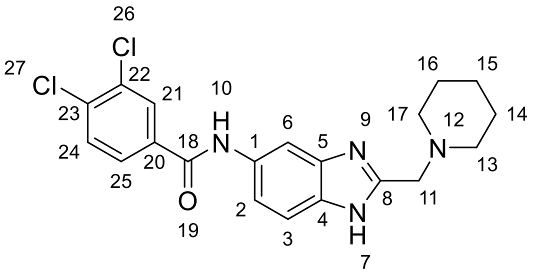


Synthesised according to general procedure **C** to give **71** (0.031 g, 0.076 mmol, 35 %) as an off white solid.

**Mpt:** 142.2-144.2 °C; **$v_{max}$ (cm$^{-1}$)** 2932, 1645, 1451, 1239, 1134, 1030, 746; **$^1$H NMR (400 MHz, DMSO-*d*$_6$) δ** 12.2 (s, 1H, 10), 10.4 (d, *J* = 19.7 Hz, 1H, 7), 8.2 (d, *J* = 2.1 Hz, 1H, 21), 8.1 – 7.9 (m, 2H, 6, 24), 7.8 (d, *J* = 8.4 Hz, 1H, 25), 7.5 – 7.3 (m, 2H, 2, 3), 3.6 (s, 2H, 11), 2.4 (t, *J* = 5.1 Hz, 4H, 13, 17), 1.5 (p, *J* = 5.5 Hz, 4H, 14, 16), 1.4 (d, *J* = 7.7 Hz, 2H, 15); **$^{13}$C NMR (101 MHz, DMSO-*d*$_6$) δ** 162.8 (18), 152.3 (8), 131.3 (21), 130.7 (25), 129.6 (23), 128.0 (22), 118.1 (3), 115.0 (2), 110.8 (6), 56.7 (11), 54.2 (13, 17), 25.4 (14, 16), 23.8 (15); **LR-ESI-MS**: $C_{20}H_{21}Cl_2N_4O$ [M+H]$^+$ *m/z* found 403.32, cald 403.11; **HR-ESI-MS**: $C_{20}H_{21}Cl_2N_4O$ [M+H]$^+$ *m/z* found 403.1072, cald 403.1092.

3,4-dimethyl-*N*-(2-(piperidin-1-ylmethyl)-1H-benzo[d]imidazol-5-yl)benzamide **72**

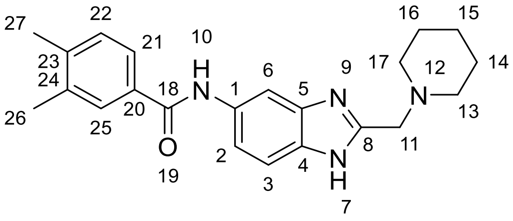


Synthesised according to general procedure **C** to give **72** (0.031 g, 0.085 mmol, 39 %) as an off white solid.

**Mpt:** 124.0-126.0 °C; **$v_{max}$ (cm$^{-1}$)** 2932, 1529, 1449, 806, 753; **$^1$H NMR (400 MHz, DMSO-*d*$_6$) δ** 12.9 – 11.5 (m, 0H, 10), 10.1 (s, 1H, 7), 8.1 (s, 1H, 6), 7.8 (d, *J* = 1.9 Hz, 1H, 25), 7.7 (dd, *J* = 2.1, 7.7 Hz, 1H, 21), 7.4 (s, 2H, 2, 3), 7.2 (dd, *J* = 7.8, 21.2 Hz, 1H, 22), 3.6 (s, 2H, 11), 2.4 (t, *J* = 5.3 Hz, 4H, 13, 17), 2.3 – 2.2 (m, 7H, 26, 27), 1.5 (p, *J* = 5.5 Hz, 4H, 14, 16), 1.4 – 1.3 (m, 2H, 15); **$^{13}$C NMR (101 MHz, DMSO-*d*$_6$) δ** 165.2 (18), 140.0 (24), 136.2 (23), 132.7 (20), 130.3 (2), 129.5 (3), 129.4 (21), 128.6 (25), 126.9 (6), 125.1 (22), 56.7 (11), 54.2 (13, 17), 25.5 (14, 16), 23.8 (15), 19.5 (27), 19.4 (26); **LR-ESI-MS**: $C_{22}H_{27}N_4O$ [M+H]$^+$ *m/z* found 363.42, cald 363.22; **HR-ESI-MS**: $C_{22}H_{27}N_4O$ [M+H]$^+$ *m/z* found 363.2169, cald 363.2185.

4-chloro-*N*-(2-(piperidin-1-ylmethyl)-1H-benzo[d]imidazol-5-yl)benzamide **73**

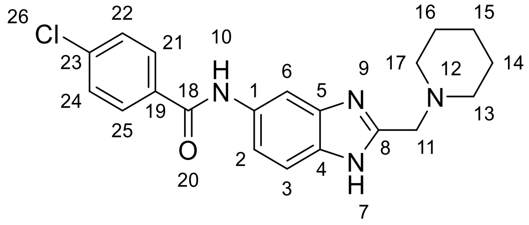


Synthesised according to general procedure **C** to give **73** (0.061 g, 0.166 mmol, 77 %) as a white solid.

**Mpt:** 122.3-124.3 °C; **$v_{max}$ (cm$^{-1}$)** 2932, 1645, 1593, 1335, 1274, 843, 749; **$^{1}$H NMR (400 MHz, DMSO-*d*$_6$) δ** 13.2 (s, 1H, 10), 11.3 (s, 1H, 7), 9.1 – 8.8 (m, 3H, 3, 21, 25), 8.7 – 8.3 (m, 4H, 2, 6, 22, 24), 4.6 (s, 2H, 11), 3.5 (p, *J* = 1.8 Hz, 4H, 13, 17), 3.4 (t, *J* = 5.1 Hz, 4H, 14, 16), 2.4 (q, *J* = 6.0 Hz, 2H, 15); **$^{13}$C NMR (101 MHz, DMSO-*d*$_6$) δ** 164.1 (18), 136.2 (23), 133.9 (19), 131.1 (3), 129.6 (21, 25), 128.4 (22, 24), 128.4 (2, 6), 56.6 (11), 54.1 (13, 17), 25.4 (14, 16), 23.8 (15); **LR-ESI-MS**: $C_{20}H_{22}ClN_4O$ [M+H]$^+$ *m/z* found 369.27, cald 369.15; **HR-ESI-MS**: $C_{20}H_{22}ClN_4O$ [M+H]$^+$ *m/z* found 367.1467, cald 369.1482.

4-methoxy-*N*-(2-(piperidin-1-ylmethyl)-1H-benzo[d]imidazol-5-yl)benzamide **74**

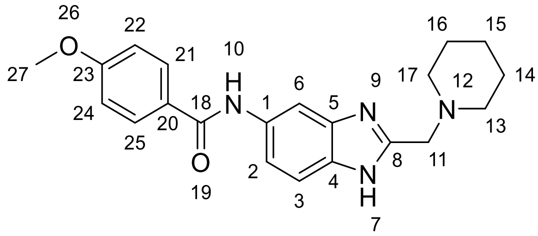


Synthesised according to general procedure **C** to give **74** (0.009 g, 0.025 mmol, 11 %) as a white solid.

**Mpt:** 117.6-119.6 °C; **$v_{max}$ (cm$^{-1}$)** 2932, 1604, 1451, 1177, 1026, 842, 760, 611; **$^{1}$H NMR (400 MHz, DMSO-*d*$_6$) δ** 12.2 (s, 1H, 7), 10.1 (s, 1H, 10), 8.0 (s, 1H, 6), 8.0 – 7.8 (m, 2H, 21, 25), 7.4 (s, 2H, 2, 3), 7.1 – 6.7 (m, 2H, 22, 24), 3.8 (s, 3H, 27), 3.6 (s, 2H, 11), 2.4 (t, *J* = 5.3 Hz, 4H, 13, 17), 1.5 (q, *J* = 5.6 Hz, 4H, 14, 16), 1.4 – 1.3 (m, 2H, 15); **$^{13}$C NMR (101 MHz, DMSO-*d*$_6$) δ** 164.6 (18), 161.7 (23), 129.5 (21, 25), 127.3 (20), 113.5 (22, 24), 56.7 (11), 55.4 (27), 54.2 (13, 17), 25.5 (14, 16), 23.8 (15); **LR-ESI-MS**: $C_{21}H_{25}N_4O_2$ [M+H]$^+$ *m/z* found 365.37, cald 365.19; **HR-ESI-MS**: $C_{21}H_{25}N_4O_2$ [M+H]$^+$ *m/z* found 365.1964, cald 365.1978.

*N*-(2-(piperidin-1-ylmethyl)-1H-benzo[d]imidazol-5-yl)benzenesulfonamide **75**

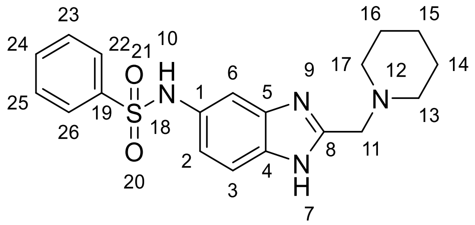


Synthesised according to general procedure **C** to give **75** (0.017 g, 0.045 mmol, 21 %) as an off white solid.

**Mpt:** 154.5-156.5 °C; **$v_{max}$ ($cm^{-1}$)** 2935, 1625, 1447, 1168, 724, 574; **$^1H$ NMR (400 MHz, DMSO-$d_6$) δ** 8.4 – 8.3 (m, 2H, 22, 26), 7.8 – 7.7 (m, 1H, 24), 7.7 – 7.6 (m, 2H, 23, 25), 7.6 (d, *J* = 8.8 Hz, 1H, 3), 6.7 (d, *J* = 2.1 Hz, 1H, 6), 6.7 (dd, *J* = 2.2, 8.8 Hz, 1H, 2), 5.1 (s, 2H, 7, 10), 3.8 (s, 2H, 11), 2.4 (s, 4H, 13, 17), 1.5 – 1.3 (m, 6H, 14, 15, 16); **$^{13}C$ NMR (101 MHz, DMSO-$d_6$) δ** 150.3 (8), 146.7 (5), 142.3 (1), 138.0 (24), 134.8 (19), 129.5 (22, 26), 127.3 (23, 25), 123.8 (3), 113.5 (2), 113.5 (4), 102.8 (6), 55.8 (11), 54.0 (13, 17), 25.2 (14, 16), 23.7 (15); **LR-ESI-MS**: $C_{19}H_{23}N_4O_2S$ $[M+H]^+$ *m/z* found 371.31, cald 371.15; **HR-ESI-MS**: $C_{19}H_{23}N_4O_2S$ $[M+H]^+$ *m/z* found 371.1528, cald 371.1542.

*N*-(2-(piperidin-1-ylmethyl)-1H-benzo[d]imidazol-5-yl)quinoline-6-carboxamide **76**

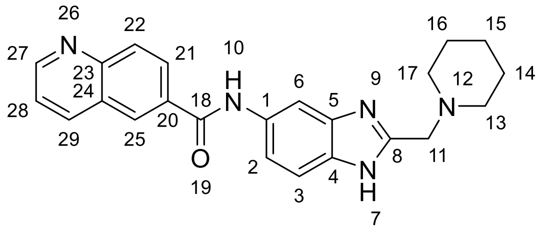

Synthesised according to general procedure **B** to give **76** (0.057 g, 0.148 mmol, 68 %) as an off white solid.

**Mpt:** 166.5-168.5 °C; **$v_{max}$ ($cm^{-1}$)** 2931, 1646, 1530, 1485, 1417, 1335, 781, 556; **$^1H$ NMR (400 MHz, DMSO-$d_6$) δ** 12.3 (s, 1H, 10), 10.5 (s, 1H, 7), 9.0 (dd, *J* = 1.7, 4.2 Hz, 1H, 29), 8.7 (d, *J* = 2.0 Hz, 1H, 25), 8.6 – 8.5 (m, 1H, 21), 8.3 (dd, *J* = 2.0, 8.8 Hz, 1H, 27), 8.1 (d, *J* = 8.8 Hz, 2H, 6, 22), 7.6 (dd, *J* = 4.2, 8.3 Hz, 1H, 28), 7.5 (s, 2H, 2, 3), 3.7 (s, 2H, 11), 2.4 (t, *J* = 5.3 Hz, 4H, 13, 17), 1.5 (q, *J* = 5.6 Hz, 4H, 14, 16), 1.4 (t, *J* = 5.5 Hz, 2H, 15); **$^{13}C$ NMR (101 MHz, DMSO-$d_6$) δ** 165.3 (18), 152.6 (27), 149.2 (29), 137.6 (25), 133.5 (21), 129.5 (22), 128.8 (20), 128.6 (24), 127.6 (28), 122.7 (23), 57.1 (11), 54.6 (13, 17), 25.9 (14, 16), 24.2 (15); **LR-ESI-MS**: $C_{23}H_{24}N_5O$ $[M+H]^+$ *m/z* found 386.52, cald 386.20; **HR-ESI-MS**: $C_{23}H_{24}N_5O$ $[M+H]^+$ *m/z* found 386.1948, cald 386.1981.

4-(1H-imidazol-1-yl)-*N*-(2-(piperidin-1-ylmethyl)-1H-benzo[d]imidazol-5-yl)benzamide **77**

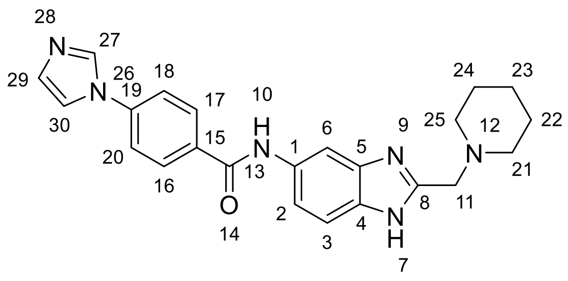

Synthesised according to general procedure **B** to give **77** (0.013 g, 0.032 mmol, 15 %) as a clear yellow oil.

**$v_{max}$ ($cm^{-1}$)** 3116, 2932, 1646, 1552, 1484, 1301, 1248, 1055, 809; **$^1H$ NMR (400 MHz, DMSO-$d_6$) δ** 12.3 (s, 1H, 10), 10.3 (s, 1H, 7), 8.4 (d, *J* = 1.2 Hz, 1H, 27), 8.2 – 8.1 (m, 2H, 16, 17), 8.1 (s, 1H, 6), 7.9 (t, *J* = 1.5 Hz, 1H, 29), 7.9 – 7.8 (m, 2H, 18, 20), 7.5 (s, 2H, 2, 3), 7.3 – 7.1 (m, 1H, 30), 3.7 (s, 2H, 11), 2.5 – 2.3 (m, 4H, 21, 25), 1.5 (q, *J* = 5.6 Hz, 4H, 22, 24), 1.4 (q, *J* = 6.0 Hz, 2H, 23); **$^{13}C$ NMR (101 MHz, DMSO-$d_6$) δ** 164.2 (13), 139.0 (19), 135.7 (27), 133.1 (15), 130.3 (29), 129.4 (16, 17), 119.5 (18, 20), 117.9 (30), 56.6 (11), 54.1

(21, 25), 25.4 (22, 24), 23.8 (23); **LR-ESI-MS**: $C_{23}H_{25}N_6O$ $[M+H]^+$ *m/z* found 401.63, cald 401.21; **HR-ESI-MS**: $C_{23}H_{25}N_6O$ $[M+H]^+$ *m/z* found 401.2056, cald 401.2090.

1-methyl-*N*-(2-(piperidin-1-ylmethyl)-1H-benzo[d]imidazol-5-yl)-1H-indazole-6-carboxamide **78**

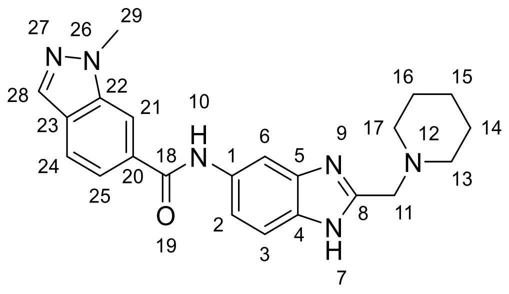

Synthesised according to general procedure **B** to give **78** (0.058 g, 0.151 mmol, 69 %) as a white solid.

**Mpt:** 160.5-162.5 °C; **$v_{max}$ ($cm^{-1}$)** 2932, 1644, 1527, 1450, 1250, 1108, 556; **$^1$H NMR (400 MHz, DMSO-*d*$_6$) δ** 12.3 (s, 1H, 10), 10.3 (s, 1H, 7), 8.3 (q, *J* = 1.1 Hz, 1H, 28), 8.1 (d, *J* = 0.9 Hz, 1H, 21), 8.1 (s, 1H, 6), 7.9 (dd, *J* = 0.8, 8.4 Hz, 1H, 25), 7.7 (dd, *J* = 1.4, 8.5 Hz, 1H, 24), 7.5 (s, 2H, 2, 3), 4.1 (s, 3H, 29), 3.7 (s, 2H, 11), 2.4 (d, *J* = 5.5 Hz, 4H, 13, 17), 1.5 (q, *J* = 5.6 Hz, 4H, 14, 16), 1.4 (t, *J* = 5.9 Hz, 2H, 15); **$^{13}$C NMR (101 MHz, DMSO-*d*$_6$) δ** 166.0 (18), 139.6 (28), 133.4 (25), 132.9 (21), 125.4 (24), 121.1 (20), 120.2 (23), 110.1 (22), 57.1 (11), 54.6 (13, 17), 36.1 (29), 25.9 (14, 16), 24.2 (15); **LR-ESI-MS**: $C_{22}H_{25}N_6O$ $[M+H]^+$ *m/z* found 389.58, cald 389.21; **HR-ESI-MS**: $C_{22}H_{25}N_6O$ $[M+H]^+$ *m/z* found 389.2063, cald 389.2090.

1-methyl-*N*-(2-(piperidin-1-ylmethyl)-1H-benzo[d]imidazol-5-yl)-1H-indazole-5-carboxamide **79**

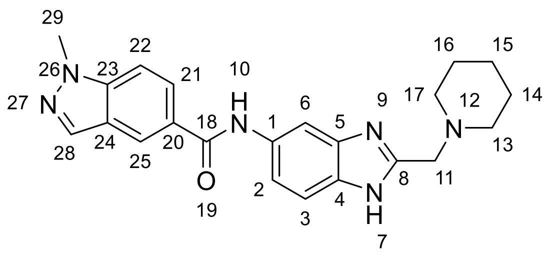

Synthesised according to general procedure **B** to give **79** (0.073 g, 0.188 mmol, 87 %) as an off white solid.

**Mpt:** 162.2-164.2 °C; **$v_{max}$ ($cm^{-1}$)** 2932, 1641, 1529, 1484, 1450, 1108, 838, 666; **$^1$H NMR (400 MHz, DMSO-*d*$_6$) δ** 12.2 (s, 1H, 10), 10.3 (s, 1H, 7), 8.5 (dd, *J* = 0.8, 1.6 Hz, 1H, 25), 8.2 (d, *J* = 0.9 Hz, 1H, 28), 8.1 (s, 1H, 6), 8.0 (dd, *J* = 1.7, 8.8 Hz, 1H, 21), 7.8 (dt, *J* = 0.9, 8.9 Hz, 1H, 22), 7.5 (s, 2H, 2, 3), 4.1 (s, 3H, 29), 3.7 (s, 2H, 11), 2.5 – 2.3 (m, 4H, 13, 17), 1.5 (q, *J* = 5.6 Hz, 4H, 14, 16), 1.4 – 1.3 (m, 2H, 15); **$^{13}$C NMR (101 MHz, DMSO-*d*$_6$) δ** 165.4 (18), 140.6 (28), 133.9 (23), 127.6 (20), 125.6 (25), 123.0 (21), 121.3 (24), 109.5 (22), 56.6 (11), 54.2 (13, 17), 35.6 (29), 25.4 (14, 16), 23.8 (15); **LR-ESI-MS**: $C_{22}H_{25}N_6O$ $[M+H]^+$ *m/z* found 389.51, cald 389.21; **HR-ESI-MS**: $C_{22}H_{25}N_6O$ $[M+H]^+$ *m/z* found 389.2065, cald 389.2090.

4-chloro-*N*-(2-(((2R,6S)-2,6-dimethylpiperidin-1-yl)methyl)-1H-benzo[d]imidazol-5-yl)benzamide **80**

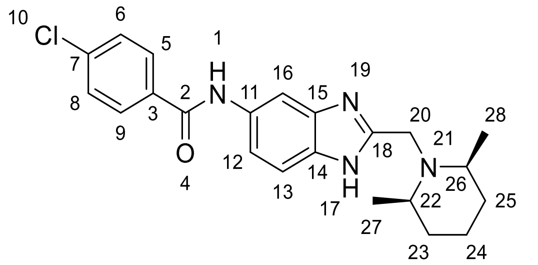

Synthesised according to general procedure **C** to give **80** (0.046 g, 0.116 mmol, 45 %) as a yellow solid.

**Mpt:** >220 °C; **$v_{max}$ ($cm^{-1}$)** 2925, 1643, 1595, 1483, 1291, 1262, 1090, 1013, 842, 807, 749; **$^1$H NMR (400 MHz, DMSO-*$d_6$*) δ** 11.9 (s, 1H, 1), 10.3 (s, 1H, 17), 8.2 – 7.8 (m, 3H, 5, 9, 16), 7.7 – 7.5 (m, 2H, 6, 8), 7.5 – 7.3 (m, 2H, 12, 13), 4.0 (s, 2H, 20''), 2.6 – 2.5 (m, 2H, 22, 26), 1.7 – 1.5 (m, 3H, 23', 24', 25'), 1.4 – 1.2 (m, 3H, 23'', 24'', 25''), 1.1 (d, *J* = 6.1 Hz, 6H, 27, 28); **$^{13}$C NMR (101 MHz, DMSO-*$d_6$*) δ** 164.2 (2), 136.1 (7), 134.0 (16), 131.1 (12, 13), 129.6 (5, 9), 128.6 (14), 128.4 (6, 8), 56.3 (20), 46.4 (22, 26), 34.1 (23, 25), 24.1 (24), 21.4 (27, 28); **LR-ESI-MS**: $C_{22}H_{26}ClN_4O$ $[M+H]^+$ *m/z* found 397.35, cald 397.18; **HR-ESI-MS**: $C_{22}H_{26}ClN_4O$ $[M+H]^+$ *m/z* found 397.1769, cald 397.1795.

4-chloro-*N*-(2-(1-(piperidin-1-yl)ethyl)-1H-benzo[d]imidazol-5-yl)benzamide **81**

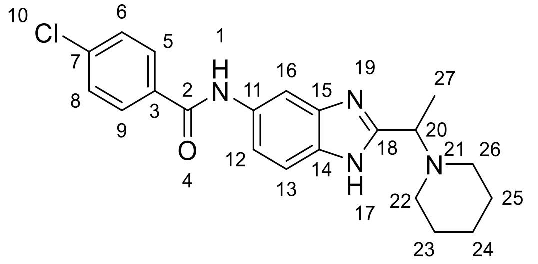

Synthesised according to general procedure **C** to give **81** (0.063 g, 0.165 mmol, 80 %) as a white solid.

**Mpt:** 144.8-146.8 °C; **$v_{max}$ ($cm^{-1}$)** 2932, 2851, 1595, 1526, 1449 1413, 1303, 1090, 843, 808, 750, 530; **$^1$H NMR (400 MHz, DMSO-*$d_6$*) δ** 12.2 (s, 1H, 1), 10.3 (s, 1H, 17), 8.1 (s, 1H, 16), 8.0 (d, *J* = 8.6 Hz, 2H, 5, 9), 7.7 – 7.5 (m, 2H, 6, 8), 7.5 (d, *J* = 8.8 Hz, 2H, 12, 13), 3.9 (q, *J* = 6.8 Hz, 1H, 20), 2.5 (t, *J* = 5.2 Hz, 4H, 22, 26), 1.5 (t, *J* = 5.9 Hz, 4H, 23, 25), 1.4 (d, *J* = 6.9 Hz, 3H, 27), 1.4 – 1.3 (m, 2H, 24); **$^{13}$C NMR (101 MHz, DMSO-*$d_6$*) δ** 164.2 (2), 136.2 (7), 133.9 (3), 131.2 (14), 129.6 (5, 9), 128.7 (15), 128.4 (6, 8), 58.5 (20), 50.3 (22, 26), 25.6 (23, 25), 24.0 (24), 14.5 (27); **LR-ESI-MS**: $C_{21}H_{24}ClN_4O$ $[M+H]^+$ *m/z* found 383.49, cald 383.16; **HR-ESI-MS**: $C_{21}H_{24}ClN_4O$ $[M+H]^+$ *m/z* found 383.1610, cald 383.1639.

N-(2-(((cis)-3,5-dimethylpiperidin-1-yl)methyl)-1H-benzo[d]imidazol-5-yl)-1-methyl-1H-indazole-5-carboxamide **82**

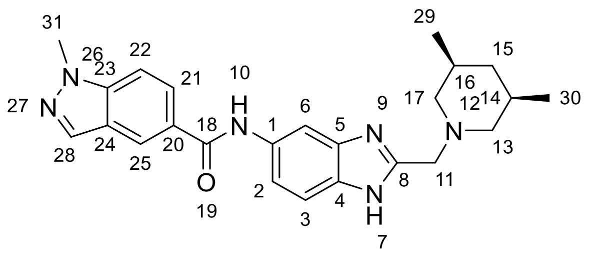

Synthesised according to general procedure **B** to give **82** (0.039 g, 0.094 mmol, 48 %) as a white solid.

**Mpt:** 156.6-158.6 °C; **$\nu_{max}$ (cm$^{-1}$)** 2948, 2923, 1599, 1484, 1339, 1250, 1191, 843, 622; **$^{1}$H NMR (400 MHz, DMSO-*d*$_6$) δ** 12.2 (s, 1H, 10), 10.3 (s, 1H, 7), 8.5 (dd, *J* = 0.8, 1.7 Hz, 1H, 28), 8.2 (d, *J* = 0.9 Hz, 1H, 25), 8.1 (s, 1H, 6), 8.0 (dd, *J* = 1.6, 8.9 Hz, 1H, 21), 7.8 (dt, *J* = 0.9, 8.9 Hz, 1H, 22), 7.5 (s, 2H, 2, 3), 4.1 (s, 3H, 31), 3.7 (s, 2H, 11), 2.8 (dt, *J* = 2.3, 10.6 Hz, 2H, 13'', 17''), 1.8 – 1.6 (m, 3H, 13', 15'', 17'), 1.6 (t, *J* = 10.7 Hz, 2H, 14, 16), 0.8 (d, *J* = 6.3 Hz, 6H, 29, 30), 0.6 – 0.4 (m, 1H, 15'); **$^{13}$C NMR (101 MHz, DMSO-*d*$_6$) δ** 165.4 (18), 140.6 (23), 133.9 (21), 127.6 (28), 125.6 (25), 123.0 (24), 121.3 (20), 109.5 (22), 61.1 (13, 17), 56.1 (11), 41.6 (31), 35.6 (15), 30.6 (14, 16), 19.5 (29, 30); **LR-ESI-MS**: $C_{24}H_{29}N_6O$ [M+H]$^+$ *m/z* found 417.42, cald 417.24; **HR-ESI-MS**: $C_{24}H_{29}N_6O$ [M+H]$^+$ *m/z* found 417.2367, cald 417.2403.

*N*-(2-((4-methylpiperidin-1-yl)methyl)-1H-benzo[d]imidazol-5-yl)benzamide **83**

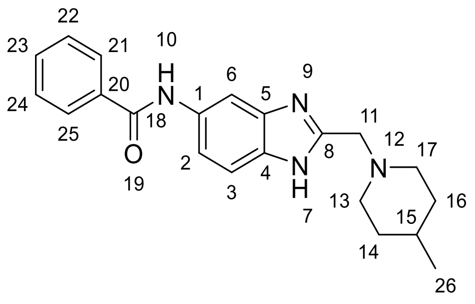

Synthesised according to general procedure **C** to give **83** (0.015 g, 0.044 mmol, 22 %) as an off white solid.

**Mpt:** 135.5-137.5 °C; **$\nu_{max}$ (cm$^{-1}$)** 2920, 1528, 1331, 1280, 1080, 806, 692, 626; **$^{1}$H NMR (400 MHz, DMSO-*d*$_6$) δ** 12.2 (s, 1H, 10), 10.2 (d, *J* = 20.9 Hz, 1H, 7), 8.1 (s, 1H, 6), 8.1 – 7.9 (m, 2H, 21, 25), 7.6 – 7.3 (m, 5H, 2, 3, 22, 23, 24), 3.7 (s, 2H, 11), 2.9 – 2.7 (m, 2H, 13'', 17''), 2.0 (td, *J* = 2.4, 11.6 Hz, 2H, 13', 17'), 1.6 – 1.5 (m, 2H, 14'', 16''), 1.4 – 1.3 (m, 1H, 15), 1.2 – 1.1 (m, 2H, 14', 16'), 0.9 (d, *J* = 6.4 Hz, 3H, 26); **$^{13}$C NMR (101 MHz, DMSO-*d*$_6$) δ** 165.3 (18), 135.3 (20), 131.4 (23), 128.4 (21, 25), 127.6 (22, 24), 56.3 (11), 53.6 (13, 17), 33.9 (14, 16), 30.1 (15), 21.9 (26); **LR-ESI-MS**: $C_{21}H_{25}N_4O$ [M+H]$^+$ *m/z* found 349.60, cald 349.20; **HR-ESI-MS**: $C_{21}H_{25}N_4O$ [M+H]$^+$ *m/z* found 349.2008, cald 349.2028.

4-chloro-*N*-(2-(2-(piperidin-1-yl)ethyl)-1H-benzo[d]imidazol-5-yl)benzamide **84**

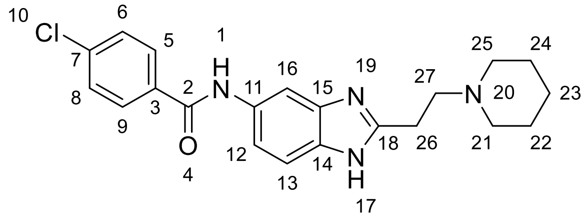

Synthesised according to general procedure **C** to give **84** (0.015 g, 0.04 mmol, 49 %) as an off white solid.

**Mpt:** 124.7-126.7 °C; **$\nu_{max}$ (cm$^{-1}$)** 3192, 2932, 1651, 1597, 1526, 1288, 1014, 810, 761, 656; **$^{1}$H NMR (400 MHz, DMSO-*d*$_6$) δ** 12.0 (s, 1H, 1), 10.3 (s, 1H, 17), 8.1 – 8.0 (m, 3H, 5, 9, 16), 7.7 – 7.6 (m, 2H, 6, 8), 7.4 (d, *J* = 1.6 Hz, 2H, 12, 13), 3.0 (dd, *J* = 6.8, 8.4 Hz, 2H, 27), 2.8 (t, *J* = 7.6 Hz, 2H, 26), 2.5 – 2.4 (m, 4H, 21, 25), 1.5 (q, *J* = 5.6 Hz, 4H, 22, 24), 1.4 (q, *J* = 6.2 Hz, 2H, 23); **$^{13}$C NMR (101 MHz, DMSO-*d*$_6$) δ** 164.1 (2), 154.0 (18), 136.2 (7), 133.9 (14, 15), 133.1 (3), 129.6 (5, 9), 128.4 (6, 8), 115.3 (12, 13), 56.5 (27), 53.6 (21, 25), 26.3 (26), 25.3 (22, 24), 23.8 (23); **LR-ESI-MS**: $C_{21}H_{24}ClN_4O$ [M+H]$^+$ *m/z* found 383.50, cald 383.16; **HR-ESI-MS**: $C_{21}H_{24}ClN_4O$ [M+H]$^+$ *m/z* found 383.1611, cald 383.1639.

1-methyl-*N*-(2-((2-methylpyrrolidin-1-yl)methyl)-1H-benzo[d]imidazol-5-yl)-1H-indazole-5-carboxamide **85**

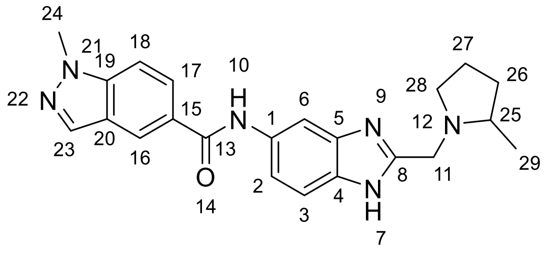

Synthesised according to general procedure **B** to give **85** (0.075 g, 0.193 mmol, 89 %) as a white solid.

**Mpt:** 179.4-181.4 °C; **$\nu_{max}$ (cm$^{-1}$)** 2962, , 1619, 1485, 1450, 1192, 836, 759, 555; **$^{1}$H NMR (400 MHz, DMSO-*d*$_6$) δ** 10.3 (s, 1H, 7), 8.5 (dd, *J* = 0.8, 1.7 Hz, 1H, 6), 8.2 (d, *J* = 0.9 Hz, 1H, 16), 8.1 (dd, *J* = 0.8, 1.7 Hz, 1H, 23), 8.0 (dd, *J* = 1.6, 8.8 Hz, 1H, 17), 7.8 – 7.7 (m, 1H, 18), 7.6 – 7.4 (m, 2H, 2, 3), 4.2 (d, *J* = 14.2 Hz, 1H, 11''), 4.1 (s, 3H, 24), 3.7 (d, *J* = 14.2 Hz, 1H, 11'), 3.1 (ddd, *J* = 4.0, 7.3, 9.6 Hz, 1H, 25), 2.8 – 2.6 (m, 1H, 28''), 2.5 (s, 1H, 28'), 2.0 – 1.9 (m, 1H, 26''), 1.8 – 1.6 (m, 2H, 26', 27'), 1.5 – 1.4 (m, 1H, 27''), 1.1 (d, *J* = 6.1 Hz, 3H, 29); **$^{13}$C NMR (101 MHz, DMSO-*d*$_6$) δ** 165.5 (13), 151.6 (8), 140.7 (23), 134.9 – 133.5 (4, 19), 127.9 (20), 127.6 (16), 125.7 (17), 123.0 (15), 121.4 (1), 119.6 (2), 115.8 (3), 109.5 (6, 18), 59.8 (11), 53.9 (25), 50.4 (28), 35.6 (24), 32.2 (26), 21.3 (27), 18.2 (29); **LR-ESI-MS**: $C_{22}H_{25}N_6O$ [M+H]$^+$ *m/z* found 389.50, cald 389.21; **HR-ESI-MS**: $C_{22}H_{25}N_6O$ [M+H]$^+$ *m/z* found 389.2062, cald 389.2090.

*N*-(2-(((cis)-3-azabicyclo[3.1.0]hexan-3-yl)methyl)-1H-benzo[d]imidazol-5-yl)-1-methyl-1H-indazole-5-carboxamide **86**

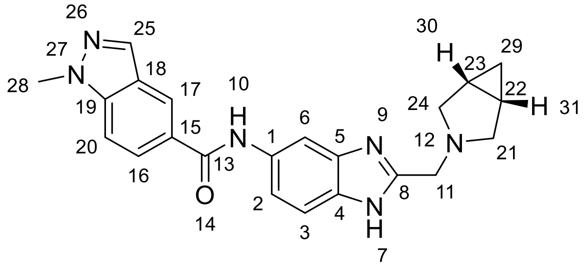

Synthesised according to general procedure **B** to give **86** (0.013 g, 0.033 mmol, 15 %) as a yellow solid.

**Mpt:** 135.6-137.6 °C; **$v_{max}$ (cm$^{-1}$)** 2794, 1640, 1529, 1450, 1315, 839, 556; **$^{1}$H NMR (400 MHz, DMSO-*d*$_6$) δ** 12.1 (s, 1H, 10), 10.3 (s, 1H, 7), 8.6 – 8.4 (m, 1H, 25), 8.2 (d, *J* = 0.9 Hz, 1H, 17), 8.1 (s, 1H, 6), 8.0 (dd, *J* = 1.6, 8.9 Hz, 1H, 16), 7.8 (dd, *J* = 1.0, 8.8 Hz, 1H, 20), 7.5 (d, *J* = 2.5 Hz, 2H, 2, 3), 4.1 (s, 3H, 28), 3.8 (s, 2H, 11'), 2.9 (d, *J* = 8.7 Hz, 2H, 21', 24'), 2.5 (p, *J* = 1.9 Hz, 2H, 21'', 24''), 1.4 (dt, *J* = 2.9, 7.3 Hz, 2H, 30, 31), 0.8 (t, *J* = 3.8 Hz, 1H, 29'), 0.4 (td, *J* = 3.9, 7.7 Hz, 1H, 29''); **$^{13}$C NMR (101 MHz, DMSO-*d*$_6$) δ** 165.4 (13), 140.6 (19), 133.9 (25), 127.6 (15), 125.6 (16), 122.9 (17), 121.3 (18), 109.5 (20), 54.3 (21, 24), 51.6 (11), 35.6 (28), 15.2 (22, 23), 6.5 (29); **LR-ESI-MS**: $C_{22}H_{23}N_6O$ $[M+H]^+$ *m/z* found 387.49, cald 387.19; **HR-ESI-MS**: $C_{22}H_{23}N_6O$ $[M+H]^+$ *m/z* found 387.1913, cald 387.1933.

*N*-(2-((2-ethylpyrrolidin-1-yl)methyl)-1H-benzo[d]imidazol-5-yl)-1-methyl-1H-indazole-5-carboxamide **87**

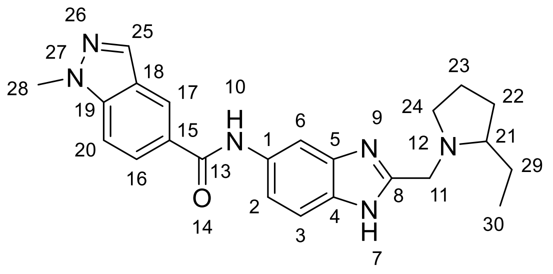

Synthesised according to general procedure **B** to give **87** (0.017 g, 0.043 mmol, 21 %) as a yellow solid.

**Mpt:** 211.5-213.5 °C; **$v_{max}$ (cm$^{-1}$)** 2958, 1641, 1529, 1450, 1414, 1191, 808, 758; **$^{1}$H NMR (400 MHz, DMSO-*d*$_6$) δ** 12.1 (s, 1H, 10), 10.2 (d, *J* = 20.9 Hz, 1H, 7), 8.5 (dd, *J* = 0.8, 1.6 Hz, 1H, 17), 8.2 (d, *J* = 0.9 Hz, 1H, 25), 8.1 (d, *J* = 11.9 Hz, 1H, 6), 8.0 (dd, *J* = 1.6, 8.8 Hz, 1H, 16), 7.8 (dt, *J* = 0.9, 8.8 Hz, 1H, 20), 7.5 (qq, *J* = 8.6, 17.2 Hz, 2H, 2, 3), 4.1 (s, 3H, 28), 4.1 (d, *J* = 14.2 Hz, 1H, 11''), 3.5 (d, *J* = 14.2 Hz, 1H, 11'), 3.0 – 2.8 (m, 1H, 21), 2.4 – 2.2 (m, 2H, 24), 1.9 (dq, *J* = 7.7, 12.2 Hz, 1H, 23''), 1.7 (dtd, *J* = 3.5, 5.9, 6.6, 14.0 Hz, 3H, 22, 23'), 1.4 (dt, *J* = 7.7, 12.2 Hz, 1H, 29''), 1.3 – 1.2 (m, 1H, 29'), 0.9 – 0.7 (m, 3H); **$^{13}$C NMR (101 MHz, DMSO-*d*$_6$) δ** 165.4 (13), 140.6 (19), 139.7 (1), 133.9 (25), 127.6 (15), 125.6 (16), 122.9 (17), 121.3 (18), 117.9 (2), 115.1 (3), 109.5 (20), 103.2 (6), 65.0 (11), 54.3 (21), 51.4 (24), 35.6 (28), 29.5 (22), 26.0 (23), 21.8 (29), 10.3 (30); **LR-ESI-MS**: $C_{23}H_{27}N_6O$ $[M+H]^+$ *m/z* found 403.51, cald 403.23; **HR-ESI-MS**: $C_{23}H_{27}N_6O$ $[M+H]^+$ *m/z* found 403.2225, cald 403.2246.

*N*-(2-((2-(methoxymethyl)pyrrolidin-1-yl)methyl)-1H-benzo[d]imidazol-5-yl)-1-methyl-1H-indazole-5-carboxamide **88**

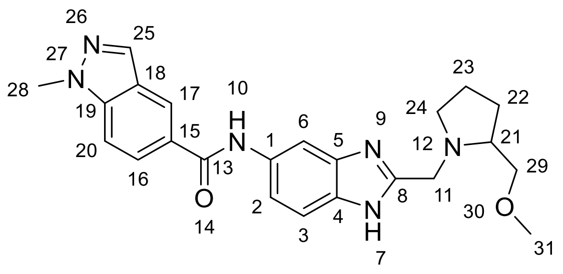

Synthesised according to general procedure **B** to give **88** (0.034 g, 0.081 mmol, 21 %) as an orange foam.

**Mpt:** 126.5-128.5 °C; **$v_{max}$ (cm$^{-1}$)** 2871, 1617, 1449, 1252, 1191, 1107, 808, 759; **$^{1}$H NMR (400 MHz, DMSO-*d*$_6$) δ** 12.1 (s, 1H, 10), 10.2 (d, *J* = 22.1 Hz, 1H, 7), 8.5 (dd, *J* = 0.8, 1.7 Hz, 1H, 17), 8.2 (d, *J* = 1.0 Hz, 1H, 25), 8.1 (d, *J* = 10.6 Hz, 1H, 6), 8.0 (dd, *J* = 1.6, 8.9 Hz, 1H, 20), 7.8 (dt, *J* = 1.0, 8.9 Hz, 1H, 16), 7.5 (td, *J* = 16.3, 23.5, 24.7 Hz, 2H, 2, 3), 4.2 (d, *J* = 14.4 Hz, 1H, 11''), 4.1 (s, 3H, 28), 3.7 (d, *J* = 14.3 Hz, 1H, 11'), 3.4 (dd, *J* = 5.3, 9.4 Hz, 1H, 21), 3.2 (s, 4H, 29'', 31), 2.9 (ddd, *J* = 3.9, 5.8, 9.4 Hz, 1H, 29'), 2.8 – 2.7 (m, 1H, 24''), 2.4 (td, *J* = 7.8, 9.0 Hz, 1H, 24'), 1.9 (dq, *J* = 8.2, 12.3 Hz, 1H, 22''), 1.7 (tt, *J* = 3.1, 7.4, 9.2 Hz, 2H, 22', 23''), 1.5 (ddd, *J* = 6.6, 12.4, 13.8 Hz, 1H, 23'); **$^{13}$C NMR (101 MHz, DMSO-*d*$_6$) δ** 165.4 (13), 152.9 (8), 140.6 (19), 133.9 (16), 127.6 (25), 125.6 (17), 122.9 (18), 121.3 (15), 118.0 (2), 115.1 (3), 109.5 (20), 103.2 (6), 75.8 (29), 62.1 (31), 58.4 (21), 54.4 (11), 52.2 (24), 38.3 (28), 35.6 (22), 28.3 (23); **LR-ESI-MS**: $C_{23}H_{27}N_6O_2$ [M+H]$^+$ *m/z* found 419.52, cald 419.22; **HR-ESI-MS**: $C_{23}H_{27}N_6O_2$ [M+H]$^+$ *m/z* found 419.2172, cald 419.2195.

(*S*)-1-methyl-*N*-(2-((3-methylpyrrolidin-1-yl)methyl)-1H-benzo[d]imidazol-5-yl)-1H-indazole-5-carboxamide **89**

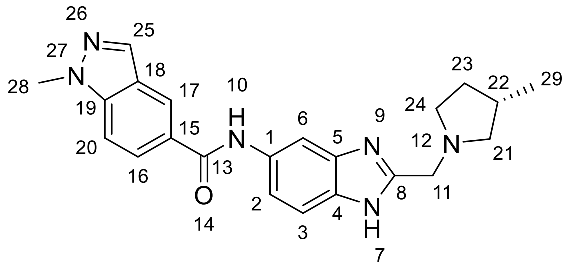

Synthesised according to general procedure **B** to give **89** (0.016 g, 0.041 mmol, 38 %) as an off white solid.

**Mpt:** 144.1-146.1 °C; **$v_{max}$ (cm$^{-1}$)** 2952, 2922, 1640, 1618, 1482, 1450, 1191, 842, 807, 758, 621; **$^{1}$H NMR (400 MHz, DMSO-*d*$_6$) δ** 12.3 (s, 1H, 10), 10.3 (s, 1H, 7), 8.5 (d, *J* = 1.6 Hz, 1H, 17), 8.2 (d, *J* = 0.9 Hz, 1H, 25), 8.1 (s, 1H, 6), 8.0 (dd, *J* = 1.6, 8.8 Hz, 1H, 20), 7.8 (d, *J* = 8.8 Hz, 1H, 16), 7.5 (s, 2H, 2, 3), 4.1 (s, 3H, 28), 3.9 – 3.7 (m, 2H, 11''), 2.8 (dd, *J* = 7.3, 8.8 Hz, 1H, 21'), 2.7 – 2.6 (m, 1H, 24'), 2.6 (td, *J* = 5.9, 8.7 Hz, 1H, 21''), 2.3 – 2.2 (m, 1H, 24''), 2.1 – 2.1 (m, 1H, 22), 2.0 – 1.9 (m, 1H, 23''), 1.3 (ddt, *J* = 6.2, 8.3, 12.3 Hz, 1H, 23'), 1.0 (d, *J* = 6.7 Hz, 3H, 29); **$^{13}$C NMR (101 MHz, DMSO-*d*$_6$) δ** 165.4 (13), 140.6 (19), 133.9 (25), 127.6 (18), 125.6 (16), 122.9 (15), 121.3 (17), 109.5 (20), 61.7 (11), 53.7 (24), 53.3 (21), 35.6 (28), 32.4 (23), 31.5 (22), 20.3 (29); **LR-ESI-MS**: $C_{22}H_{25}N_6O$ [M+H]$^+$ *m/z* found 389.54, cald 389.21; **HR-ESI-MS**: $C_{22}H_{25}N_6O$ [M+H]$^+$ *m/z* found 389.2070, cald 389.2090.

(*R*)-1-methyl-*N*-(2-((3-methylpyrrolidin-1-yl)methyl)-1H-benzo[d]imidazol-5-yl)-1H-indazole-5-carboxamide **90**

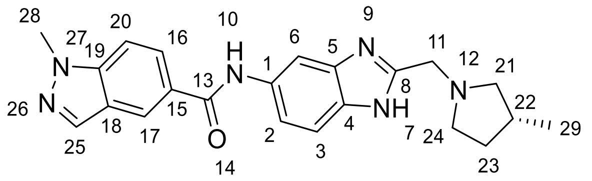

Synthesised according to general procedure **B** to give **90** (0.028 g, 0.07 mmol, 33 %) as an off white solid.

**Mpt:** 167.0-169.0 °C; **$\nu_{max}$ ($cm^{-1}$)** 2954, 1619, 1530, 1485, 1452, 834, 556; **$^1$H NMR (400 MHz, DMSO-*d*$_6$) δ** 12.2 (s, 1H, 10), 10.3 (s, 1H, 7), 8.5 (dd, *J* = 0.8, 1.7 Hz, 1H, 17), 8.2 (d, *J* = 0.9 Hz, 1H, 25), 8.1 (s, 1H, 6), 8.0 (dd, *J* = 1.6, 8.9 Hz, 1H, 16), 7.8 (dt, *J* = 1.0, 8.9 Hz, 1H, 20), 7.5 (s, 2H, 2, 3), 4.1 (s, 3H, 28), 3.9 (s, 2H, 11), 2.9 (s, 1H, 21'), 2.8 (t, *J* = 7.7 Hz, 1H, 24'), 2.7 (q, *J* = 4.0, 5.6 Hz, 1H, 21''), 2.2 (q, *J* = 6.2, 7.1 Hz, 2H, 22, 24''), 2.1 – 1.9 (m, 1H, 23'), 1.4 (ddt, *J* = 6.1, 8.1, 12.4 Hz, 1H, 23''), 1.0 (d, *J* = 6.3 Hz, 3H, 29); **$^{13}$C NMR (101 MHz, DMSO-*d*$_6$) δ** 165.4 (13), 140.6 (25), 133.9 (16), 127.6 (19), 125.6 (17), 122.9 (15), 121.3 (18), 109.5 (20), 61.5 (11), 53.8 (24), 53.0 (21), 35.6 (28), 32.2 (22), 31.5 (23), 19.9 (29); **LR-ESI-MS**: $C_{22}H_{25}N_6O$ [M+H]$^+$ *m/z* found 389.39, cald 389.21; **HR-ESI-MS**: $C_{22}H_{25}N_6O$ [M+H]$^+$ *m/z* found 389.2071, cald 389.2090.

(*R*)-1-methyl-*N*-(2-((2-methylpyrrolidin-1-yl)methyl)-1H-benzo[d]imidazol-5-yl)-1H-indazole-5-carboxamide **91**

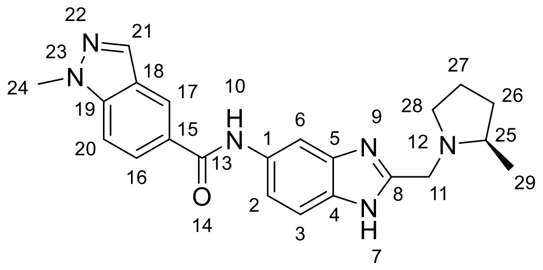

Synthesised according to general procedure **B** to give **91** (0.058 g, 0.149 mmol, 69 %) as an orange solid.

**Mpt:** 149.0-151.0 °C; **$\nu_{max}$ ($cm^{-1}$)** 2960, 1618, 1529, 1378, 1191, 840, 759, 556; **$^1$H NMR (400 MHz, DMSO-*d*$_6$) δ** 12.2 (s, 1H, 10), 10.3 (s, 1H, 7), 8.5 (dd, *J* = 0.8, 1.6 Hz, 1H, 21), 8.2 (d, *J* = 0.9 Hz, 1H, 17), 8.1 (s, 1H, 6), 8.1 – 7.9 (m, 1H, 16), 7.8 (dt, *J* = 0.9, 8.8 Hz, 1H, 20), 7.5 – 7.4 (m, 2H, 2, 3), 4.2 – 4.0 (m, 4H, 11'', 24), 3.6 (d, *J* = 14.2 Hz, 1H, 11'), 3.0 (ddd, *J* = 3.5, 7.4, 9.2 Hz, 1H, 25), 2.5 (d, *J* = 6.8 Hz, 1H, 28'), 2.3 (q, *J* = 8.8 Hz, 1H, 28''), 1.9 (dddd, *J* = 5.7, 7.3, 9.2, 12.4 Hz, 1H, 26''), 1.7 (dtd, *J* = 4.2, 8.0, 8.8, 15.0 Hz, 2H, 26', 27''), 1.4 (dddd, *J* = 6.4, 8.5, 10.0, 12.3 Hz, 1H, 27'), 1.1 (d, *J* = 6.0 Hz, 3H, 29); **$^{13}$C NMR (101 MHz, DMSO-*d*$_6$) δ** 165.4 (13), 140.6 (19), 133.9 (16), 127.6 (21), 125.6 (17), 122.9 (15), 121.3 (18), 109.5 (20), 59.3 (11), 54.0 (25), 50.8 (28), 35.6 (24), 32.4 (26), 21.4 (27), 18.7 (29); **LR-ESI-MS**: $C_{22}H_{25}N_6O$ [M+H]$^+$ *m/z* found 389.58, cald 389.21; **HR-ESI-MS**: $C_{22}H_{25}N_6O$ [M+H]$^+$ *m/z* found 389.2069, cald 389.2090.

(*S*)-1-methyl-*N*-(2-((2-methylpyrrolidin-1-yl)methyl)-1H-benzo[d]imidazol-5-yl)-1H-indazole-5-carboxamide **92**

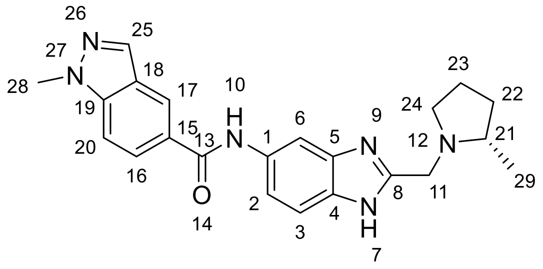

Synthesised according to general procedure **B** to give **92** (0.076 g, 0.196 mmol, 52 %) as an off white solid.

**Mpt:** 176.7-178.7 °C; **$\nu_{max}$ ($cm^{-1}$)** 2960, 1619, 837, 556; **$^{1}$H NMR (400 MHz, DMSO-$d_6$) δ** 12.2 (s, 1H, 10), 10.2 (d, *J* = 21.8 Hz, 1H, 7), 8.5 (t, *J* = 1.1 Hz, 1H, 17), 8.2 (d, *J* = 0.9 Hz, 1H, 20), 8.1 (s, 1H, 25), 8.0 (dd, *J* = 1.7, 8.8 Hz, 1H, 16), 7.8 (d, *J* = 8.9 Hz, 1H, 3), 7.6 – 7.3 (m, 2H, 2, 6), 4.1 (s, 3H, 28), 4.0 (d, *J* = 14.2 Hz, 1H, 11''), 3.5 (d, *J* = 14.1 Hz, 1H, 11'), 2.9 (ddd, *J* = 3.3, 7.5, 9.0 Hz, 1H, 21), 2.5 (s, 1H, 24''), 2.3 (q, *J* = 8.7 Hz, 1H, 24'), 1.9 (dddd, *J* = 5.6, 7.2, 9.4, 12.5 Hz, 1H, 22''), 1.7 – 1.6 (m, 2H, 22', 23''), 1.5 – 1.3 (m, 1H, 23'), 1.1 (d, *J* = 6.0 Hz, 3H, 29); **$^{13}$C NMR (101 MHz, DMSO-$d_6$) δ** 165.4 (8, 13), 140.6 (1, 5), 133.9 (4, 19), 127.6 (25), 125.6 (15, 17), 122.9 (16), 121.3 (18), 117.9 (2), 115.1 (20), 109.5 (3), 103.2 (6), 59.0 (11), 54.0 (21), 51.0 (24), 35.6 (28), 32.6 (22), 21.4 (23), 18.9 (29); **LR-ESI-MS**: $C_{22}H_{25}N_6O$ $[M+H]^+$ *m/z* found 389.35, cald 389.21; **HR-ESI-MS**: $C_{22}H_{25}N_6O$ $[M+H]^+$ *m/z* found 389.2069, cald 389.2090.

*N*-(2-((2-methylpyrrolidin-1-yl)methyl)-1H-benzo[d]imidazol-5-yl)-1H-indazole-5-carboxamide **93**

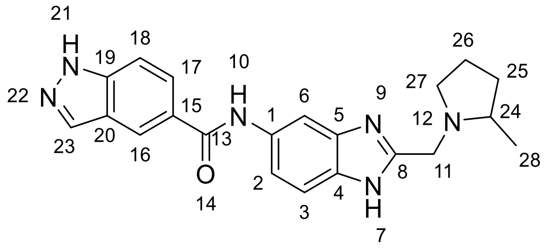

Synthesised according to general procedure **B** to give **93** (0.018 g, 0.048 mmol, 22 %) as a white solid.

**Mpt:** 197.8-199.8 °C; **$\nu_{max}$ ($cm^{-1}$)** 3129, 1623, 1530, 1485, 1376, 942, 843; **$^{1}$H NMR (400 MHz, DMSO-$d_6$) δ** 13.4 (s, 1H, 21), 12.2 (s, 1H, 10), 10.3 (s, 1H, 7), 8.6 – 8.4 (m, 1H, 23), 8.3 – 8.2 (m, 1H, 16), 8.1 (s, 1H, 6), 8.0 (ddd, *J* = 1.6, 8.8, 23.1 Hz, 1H, 17), 7.7 – 7.6 (m, 1H, 18), 7.5 – 7.4 (m, 2H, 2, 3), 4.1 (d, *J* = 14.1 Hz, 1H, 11''), 3.6 (d, *J* = 14.1 Hz, 1H, 11'), 2.9 (ddd, *J* = 3.4, 7.5, 9.1 Hz, 1H, 24), 2.5 (s, 1H, 27''), 2.3 (q, *J* = 8.8 Hz, 1H, 27'), 2.0 – 1.9 (m, 1H, 25''), 1.7 (dtd, *J* = 2.4, 5.3, 5.8, 14.8 Hz, 2H, 25', 26''), 1.4 – 1.3 (m, 1H, 26'), 1.1 (d, *J* = 6.0 Hz, 3H, 28); **$^{13}$C NMR (101 MHz, DMSO-$d_6$) δ** 165.6 (13), 127.6 (16), 126.7 (20), 125.7 (17), 123.7 (15), 122.3 (23), 121.1 (2), 109.9 (18), 59.1 (11), 54.0 (24), 50.9 (27), 32.5 (25), 21.4 (26), 18.8 (28); **LR-ESI-MS**: $C_{21}H_{23}N_6O$ $[M+H]^+$ *m/z* found 375.49, cald 375.19; **HR-ESI-MS**: $C_{21}H_{23}N_6O$ $[M+H]^+$ *m/z* found 375.1905, cald 375.1933.

(*S*)-1-cyclopropyl-*N*-(2-((2-methylpyrrolidin-1-yl)methyl)-1*H*-benzo[*d*]imidazol-5-yl)-1*H*-indazole-5-carboxamide **94**

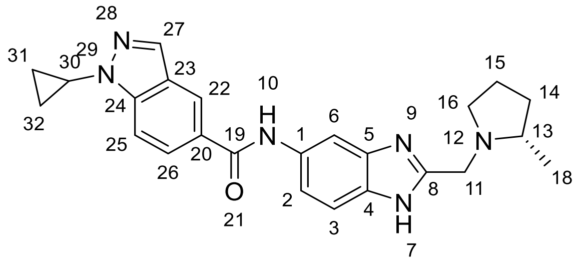

Synthesised according to general procedure **B** to give **94** (0.038 g, 0.091 mmol, 42 %) as an beige solid.

**Mpt:** 170.0-172.0 °C; **$\nu_{max}$ (cm$^{-1}$)** 3406, 2925, 1644, 1485, 1312, 1252, 838, 556; **$^1$H NMR (400 MHz, DMSO) δ** 12.19 (s, 1H, 7), 10.26 (s, 1H, 10), 8.48 (d, *J*=1.6, 1H, 22), 8.21 (s, 1H, 27), 8.13 – 7.99 (m, 2H, 6, 26), 7.79 (d, *J*=8.8, 1H, 25), 7.56 – 7.33 (m, 2H, 2, 3), 4.09 (d, *J*=14.2, 1H, 11''), 3.83 (tt, *J*=7.1, 4.0, 1H, 30), 3.59 (d, *J*=14.1, 1H, 11'), 3.06 – 2.88 (m, 1H, 13), 2.34 (d, *J*=9.1, 1H, 16'), 1.94 (dtd, *J*=12.5, 7.7, 7.1, 4.2, 1H, 16''), 1.77 – 1.61 (m, 2H, 14', 15''), 1.47 – 1.30 (m, 2H, 14'', 15'), 1.26 – 1.00 (m, 8H, 18, 31, 32); **$^{13}$C NMR (101 MHz, DMSO) δ** 165.4 (19), 141.3 (27), 134.0 (22), 128.1 (26), 125.9 (6), 123.4 (3), 121.5 (2), 109.6 (25), 54.0 (11), 50.8 (13, 16), 32.4 (30), 29.4 (14), 26.4 (15), 21.4 (18), 6.3 (31, 32); **LR-ESI-MS**: $C_{24}H_{27}N_6O$ $[M+H]^+$ *m/z* found 415.53, cald 415.23; **HR-ESI-MS**: $C_{24}H_{27}N_6O$ $[M+H]^+$ *m/z* found 415.2228, cald 415.2246.

*N*-(2-(piperidin-1-ylmethyl)-1H-benzo[d]imidazol-5-yl)-2-(trifluoromethyl)benzamide **95**

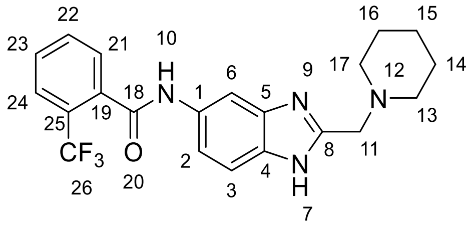

Synthesised according to general procedure **C** to give **95** (0.031 g, 0.076 mmol, 35 %) as a white solid.

**Mpt:** 139.2-141.2 °C; **$\nu_{max}$ (cm$^{-1}$)** 2934, 1652, 1532, 1270, 1034, 765; **$^{19}$F NMR (376 MHz, DMSO-*d*$_6$) δ** -57.9 , 27, 28, 29; **$^1$H NMR (400 MHz, DMSO-*d*$_6$) δ** 12.4 – 12.0 (m, 1H, 7), 10.5 (s, 1H, 10), 8.0 (d, *J* = 43.9 Hz, 1H, 6), 7.9 – 7.8 (m, 1H, 24), 7.8 – 7.8 (m, 1H, 23), 7.7 (tt, *J* = 3.1, 5.2 Hz, 2H, 21, 22), 7.4 (d, *J* = 78.9 Hz, 2H, 2, 3), 3.6 (s, 2H, 11), 2.4 (t, *J* = 5.3 Hz, 4H, 13, 17), 1.5 (p, *J* = 5.6 Hz, 4H, 14, 16), 1.4 (dt, *J* = 4.6, 11.5 Hz, 2H, 15); **$^{13}$C NMR (101 MHz, DMSO-*d*$_6$) δ** 165.3 (18), 136.6 (19), 132.6 (25), 129.9 (21), 128.6 (23), 126.3 (q, *J* = 5.0 Hz, 26), 126.0 (24), 125.7 (22), 125.2 (1), 122.5 (2), 56.6 (11), 54.1 (13, 17), 25.5 (14, 16), 23.8 (15); **LR-ESI-MS**: $C_{21}H_{22}F_3N_4O$ $[M+H]^+$ *m/z* found 403.39, cald 403.18; **HR-ESI-MS**: $C_{21}H_{22}F_3N_4O$ $[M+H]^+$ *m/z* found 403.1730, cald 403.1746.

*N*-(2-(piperidin-1-ylmethyl)-1H-benzo[d]imidazol-5-yl)benzamide **96**

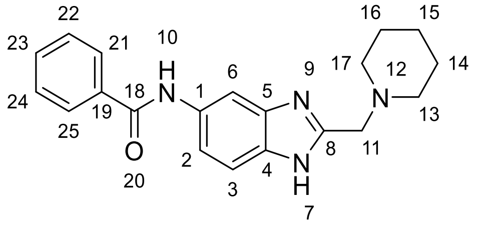

Synthesised according to general procedure **C** to give **96** (0.013 g, 0.04 mmol, 19 %) as a yellow solid.

**Mpt:** 85.6-87.6 °C; **$v_{max}$ ($cm^{-1}$)** 2933, 1600, 1527, 1277, 1108, 805, 672; **$^{1}$H NMR (400 MHz, DMSO-*$d_6$*) δ** 12.2 (s, 1H, 10), 10.2 (s, 1H, 7), 8.1 (s, 1H, 6), 8.0 (ddd, *J* = 1.4, 2.7, 6.7 Hz, 2H, 21, 25), 7.6 – 7.5 (m, 3H, 22, 23, 24), 7.5 – 7.4 (m, 2H, 2, 3), 3.6 (s, 2H, 11), 2.4 (t, *J* = 5.1 Hz, 4H, 13, 17), 1.5 (p, *J* = 5.5 Hz, 4H, 14, 16), 1.4 – 1.3 (m, 2H, 15); **$^{13}$C NMR (101 MHz, DMSO-*$d_6$*) δ** 165.3 (18), 135.3 (1), 131.4 (23), 129.2 (4), 129.1 (5), 128.8 (19), 128.4 (21, 25), 128.3 (2, 3), 127.6 (22, 24), 56.7 (11), 54.1 (13, 17), 25.4 (14, 16), 23.8 (15); **LR-ESI-MS**: $C_{20}H_{23}N_4O$ [M+H]$^+$ *m/z* found 335.39, cald 335.19; **HR-ESI-MS**: $C_{20}H_{23}N_4O$ [M+H]$^+$ *m/z* found 335.1857, cald 335.1872.

*N*-(2-(piperidin-1-ylmethyl)-1H-benzo[d]imidazol-5-yl)isonicotinamide **97**

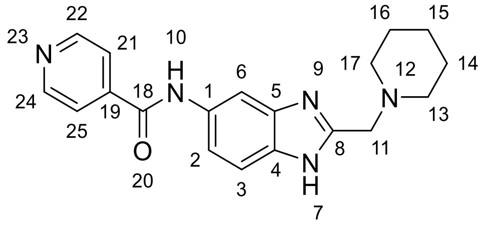

Synthesised according to general procedure **C** to give **97** (0.061 g, 0.183 mmol, 84 %) as a white solid.

**Mpt:** 84.9-86.9 °C; **$v_{max}$ ($cm^{-1}$)** 2932, 1653, 1450, 1415, 1284, 1065, 844, 809, 680, 624; **$^{1}$H NMR (400 MHz, DMSO-*$d_6$*) δ** 12.3 (s, 1H, 7), 10.5 (s, 1H, 10), 8.8 – 8.7 (m, 2H, 22, 24), 8.1 (s, 1H, 6), 8.0 – 7.8 (m, 2H, 21, 25), 7.5 (d, *J* = 2.8 Hz, 2H, 2, 3), 3.7 (s, 2H, 11), 2.5 (s, 4H, 13, 17), 1.6 (p, *J* = 5.5 Hz, 4H, 14, 16), 1.5 – 1.4 (m, 2H, 15); **$^{13}$C NMR (101 MHz, DMSO-*$d_6$*) δ** 163.7 (18), 150.2 (22, 24), 142.2 (19), 121.6 (21, 25), 56.3 (11), 54.0 (13, 17), 25.2 (14, 16), 23.6 (15); **LR-ESI-MS**: $C_{19}H_{22}N_5O$ [M+H]$^+$ *m/z* found 336.37, cald 336.18; **HR-ESI-MS**: $C_{19}H_{22}N_5O$ [M+H]$^+$ *m/z* found 336.1809, cald 336.1824.

*N*-(2-(piperidin-1-ylmethyl)-1H-benzo[d]imidazol-5-yl)picolinamide **98**

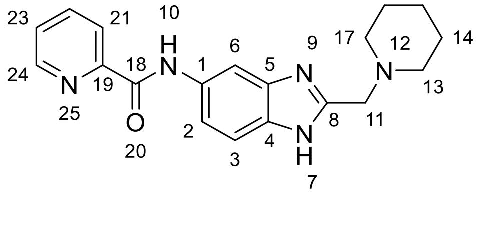

Synthesised according to general procedure **B** to give **98** (0.038 g, 0.112 mmol, 62 %) as a white solid.

**Mpt:** 170.8-172.8 °C; **$v_{max}$ (cm$^{-1}$)** 3345, 2937, 1591, 1522, 836, 807, 743, 659, 556; **$^1$H NMR (400 MHz, DMSO-*d*$_6$) δ** 12.2 (s, 1H, 10), 10.6 (s, 1H, 7), 8.7 (dt, *J* = 1.4, 4.8 Hz, 1H, 24), 8.3 – 8.1 (m, 2H, 3, 21), 8.1 (td, *J* = 1.7, 7.7 Hz, 1H, 23), 7.7 (ddd, *J* = 1.3, 4.7, 7.5 Hz, 1H, 22), 7.6 (d, *J* = 8.6 Hz, 1H, 2), 7.5 (d, *J* = 8.6 Hz, 1H, 6), 3.7 (s, 2H, 11), 1.6 (p, *J* = 5.5 Hz, 4H, 13, 17), 1.4 (dd, *J* = 5.4, 10.8 Hz, 3H, 15), 1.2 (q, *J* = 6.6, 7.2 Hz, 4H, 14, 16); **$^{13}$C NMR (101 MHz, DMSO-*d*$_6$) δ** 162.1 (18), 150.2 (19), 148.4 (22, 24), 138.1 (1), 132.8 (3), 126.8 (4, 5), 122.3 (21, 23), 115.5 (2, 6), 56.2 (11), 54.0 (13, 17), 25.2 (14, 16), 23.5 (15); **LR-ESI-MS**: $C_{19}H_{22}N_5O$ [M+H]$^+$ *m/z* found 336.38, cald 336.18; **HR-ESI-MS**: $C_{19}H_{22}N_5O$ [M+H]$^+$ *m/z* found 336.1810, cald 336.1824.

2-(3,4-dimethoxyphenyl)-*N*-(2-(piperidin-1-ylmethyl)-1H-benzo[d]imidazol-5-yl)acetamide **99**

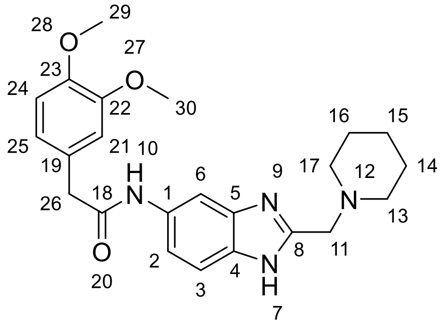


Synthesised according to general procedure **C** to give **99** (0.059 g, 0.145 mmol, 67 %) as an off white solid.

**Mpt:** 77.4-79.4 °C; **$v_{max}$ (cm$^{-1}$)** 2932, 1658, 1449, 1259, 1230, 1139, 1024, 789, 761; **$^1$H NMR (400 MHz, DMSO-*d*$_6$) δ** 12.1 (s, 1H, 10), 10.1 (s, 1H, 7), 7.4 (s, 1H, 2), 7.2 (s, 1H, 6), 7.0 – 6.6 (m, 4H, 21, 24, 25), 3.7 (s, 3H, 29), 3.7 (s, 3H, 30), 3.6 (s, 2H, 26), 3.5 (s, 2H, 11), 2.4 (t, *J* = 5.3 Hz, 4H), 1.5 (p, *J* = 5.4 Hz, 4H), 1.4 (d, *J* = 7.2 Hz, 2H); **$^{13}$C NMR (101 MHz, DMSO-*d*$_6$) δ** 173.0 (18), 169.0 (8), 148.5 (22), 147.6 (5), 128.6 (1), 127.6 (4), 121.3 (2), 121.0 (25), 113.2 (3), 113.0 (24), 111.8 (21), 111.7 (6), 56.6 (11), 55.5 (29), 55.4 (30), 54.1 (13, 17), 43.0 (26), 25.4 (14, 16), 23.8 (15); **LR-ESI-MS**: $C_{23}H_{29}N_4O_3$ [M+H]$^+$ *m/z* found 409.63, cald 409.22; **HR-ESI-MS**: $C_{23}H_{29}N_4O_3$ [M+H]$^+$ *m/z* found 409.2221, cald 409.2240.

5-methyl-*N*-(2-(piperidin-1-ylmethyl)-1H-benzo[d]imidazol-5-yl)isoxazole-3-carboxamide **100**

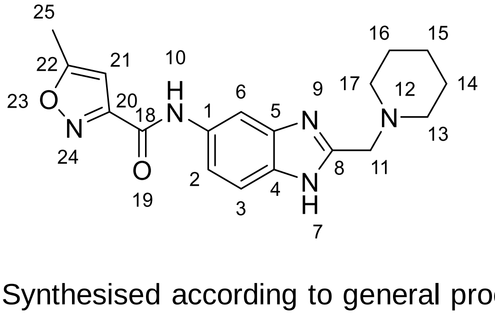


Synthesised according to general procedure **C** to give **100** (0.047 g, 0.140 mmol, 64 %) as an off white solid.

**Mpt:** 104.0-106.0 °C; **$v_{max}$ (cm$^{-1}$)** 2932, 1666, 1598, 1533, 1488, 1416, 1212, 860, 807, 610; **$^1$H NMR (400 MHz, DMSO-*d*$_6$) δ** 12.2 (s, 1H, 10), 10.6 (d, *J* = 23.0 Hz, 1H, 7), 8.0 (d, *J* =

24.8 Hz, 1H, 3), 7.6 – 7.3 (m, 2H, 2, 6), 6.7 (d, *J* = 1.1 Hz, 1H, 11, 21), 3.6 (s, 2H), 2.5 (d, *J* = 1.8 Hz, 3H, 25), 2.5 – 2.3 (m, 4H, 13, 17), 1.5 (p, *J* = 5.5 Hz, 4H, 14, 16), 1.4 – 1.3 (m, 2H, 15); **$^{13}$C NMR (101 MHz, DMSO-*d*$_6$) δ** 171.3 (22), 159.5 (20), 157.2 (18), 101.6 (2, 6), 56.6 (11), 54.1 (13, 17), 25.4 (14, 16), 23.8 (15), 11.9 (25); **LR-ESI-MS**: $C_{18}H_{22}N_5O_2$ $[M+H]^+$ *m/z* found 340.54, cald 340.18; **HR-ESI-MS**: $C_{18}H_{22}N_5O_2$ $[M+H]^+$ *m/z* found 340.1757, cald 340.1773.

4-(dimethylamino)-*N*-(2-(piperidin-1-ylmethyl)-1H-benzo[d]imidazol-5-yl)benzamide **101**

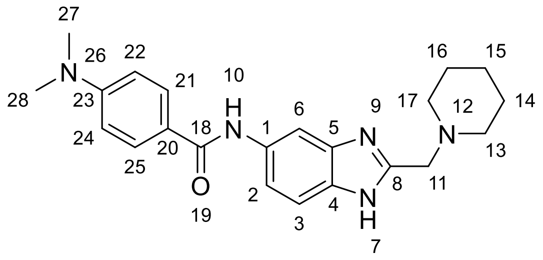


Synthesised according to general procedure **C** to give **101** (0.082 g, 0.217 mmol, 100 %) as a white solid.

**Mpt:** >240 °C; **$v_{max}$ (cm$^{-1}$)** 3290, 2932, 1639, 1605, 1441, 1193, 856; **$^1$H NMR (400 MHz, DMSO-*d*$_6$) δ** 12.2 (s, 1H, 10), 9.8 (s, 1H, 7), 8.1 (s, 1H, 3), 8.0 – 7.7 (m, 2H, 21, 25), 7.4 (s, 2H, 2, 6), 6.8 – 6.6 (m, 2H, 22, 24), 3.6 (s, 2H, 11), 3.0 (s, 6H, 27, 28), 2.5 – 2.3 (m, 4H, 13, 17), 1.5 (p, *J* = 5.5 Hz, 4H, 14, 16), 1.5 – 1.3 (m, 2H, 15); **$^{13}$C NMR (101 MHz, DMSO-*d*$_6$) δ** 172.1 (18), 165.0 (8), 152.3 (23), 130.9 (20), 129.0 (21, 25), 121.5 (2, 6), 110.8 (22, 24), 56.7 (11), 54.2 (13, 17), 25.4 (27, 28), 23.8 (14, 16), 21.1 (15); **LR-ESI-MS**: $C_{22}H_{28}N_5O$ $[M+H]^+$ *m/z* found 378.42, cald 378.23; **HR-ESI-MS**: $C_{22}H_{28}N_5O$ $[M+H]^+$ *m/z* found 378.2275, cald 378.2294.

2-chloro-5-nitro-*N*-(2-(piperidin-1-ylmethyl)-1*H*-benzo[*d*]imidazol-5-yl)benzamide **102**

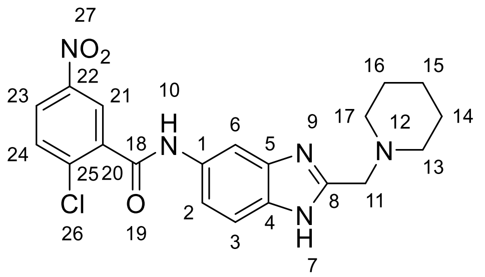


Synthesised according to general procedure **C** to give **102** (0.028 g, 0.067 mmol, 31 %) as a beige solid.

**Mpt:** >135 °C; **$v_{max}$ (cm$^{-1}$)** 2931, 1656, 1523, 1045, 738, 542; **$^1$H NMR (400 MHz, DMSO-*d*$_6$) δ** 12.3 (s, 1H, 7), 10.7 (s, 1H, 10), 8.5 (d, *J* = 2.8 Hz, 1H, 21), 8.3 (dd, *J* = 2.8, 8.8 Hz, 1H, 23), 8.1 (d, *J* = 1.9 Hz, 1H, 6), 7.9 (d, *J* = 8.9 Hz, 1H, 24), 7.5 (d, *J* = 8.6 Hz, 1H, 3), 7.3 (d, *J* = 8.7 Hz, 1H, 2), 3.7 (s, 2H, 11), 2.4 (t, *J* = 5.3 Hz, 4H, 13, 17), 1.5 (q, *J* = 5.6 Hz, 4H, 14, 16), 1.4 (td, *J* = 3.6, 6.3 Hz, 2H, 15); **$^{13}$C NMR (101 MHz, DMSO-*d*$_6$) δ** 162.5 (18), 146.2 (21), 138.1 (23), 137.2 (22), 131.3 (25), 125.6 (20), 123.9 (24), 56.5 (11), 54.1 (13, 17), 25.4 (14, 16), 23.7 (15); **LR-ESI-MS**: $C_{20}H_{21}ClN_5O_3$ $[M+H]^+$ *m/z* found 414.36, cald 414.13; **HR-ESI-MS**: $C_{20}H_{21}ClN_5O_3$ $[M+H]^+$ *m/z* found 414.1308, cald 414.1333.

*N*-(2-(piperidin-1-ylmethyl)-1H-benzo[d]imidazol-5-yl)pyrazine-2-carboxamide **103**

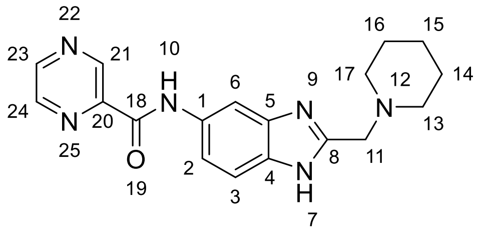


Synthesised according to general procedure **C** to give **103** (0.074 g, 0.220 mmol, 100 %) as a yellow solid.

**Mpt:** 190.9-192.9 °C; **$\nu_{max}$ (cm$^{-1}$)** 2931, 1669, 1524, 1419, 1106, 1018, 805, 650, 429; **$^{1}$H NMR (400 MHz, DMSO-*d*$_6$) δ** 12.3 (s, 1H, 7), 10.7 (s, 1H, 10), 9.3 (d, *J* = 1.5 Hz, 1H, 21), 8.9 (d, *J* = 2.5 Hz, 1H, 24), 8.8 (dd, *J* = 1.5, 2.5 Hz, 1H, 23), 8.2 (s, 1H, 6), 7.7 – 7.3 (m, 2H, 2, 3), 3.7 (s, 2H, 11), 2.4 (t, *J* = 5.4 Hz, 4H, 13, 17), 1.5 (p, *J* = 5.5 Hz, 4H, 14, 16), 1.4 (q, *J* = 6.5 Hz, 2H, 15); **$^{13}$C NMR (101 MHz, DMSO-*d*$_6$) δ** 161.3 (18), 147.5 (23), 145.4 (21), 144.0 (24), 143.2 (20), 56.6 (11), 54.1 (13, 17), 25.5 (14, 16), 23.8 (15); **LR-ESI-MS**: $C_{18}H_{21}N_6O$ $[M+H]^+$ *m/z* found 337.36, cald 337.18; **HR-ESI-MS**: $C_{18}H_{21}N_6O$ $[M+H]^+$ *m/z* found 337.1761, cald 337.1777.

2-phenoxy-*N*-(2-(piperidin-1-ylmethyl)-1H-benzo[d]imidazol-5-yl)acetamide **104**

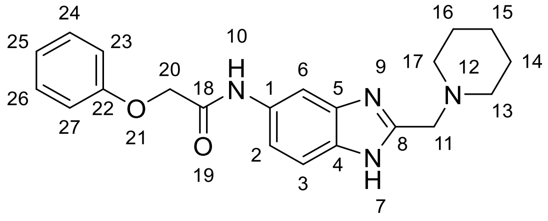


Synthesised according to general procedure **C** to give **104** (0.052 g, 0.141 mmol, 65 %) as an off white solid.

**Mpt:** 94.6-96.6 °C; **$\nu_{max}$ (cm$^{-1}$)** 2932, 1670, 1598, 1489, 807, 750, 689; **$^{1}$H NMR (400 MHz, DMSO-*d*$_6$) δ** 12.4 – 12.0 (m, 1H, 10), 10.1 (s, 1H, 7), 7.9 (d, *J* = 26.9 Hz, 1H, 3), 7.6 – 7.1 (m, 4H, 2, 6, 24, 26), 7.1 – 6.8 (m, 3H, 23, 25, 27), 4.7 (s, 2H, 20), 3.6 (s, 2H, 11), 2.4 (t, *J* = 5.2 Hz, 4H, 13, 17), 1.5 (p, *J* = 5.5 Hz, 4H, 14, 16), 1.4 (q, *J* = 4.6, 6.0 Hz, 2H, 15); **$^{13}$C NMR (101 MHz, DMSO-*d*$_6$) δ** 166.2 (18), 157.9 (8, 22), 129.5 (1, 24, 26), 121.2 (25), 114.7 (2, 3, 23, 27), 67.2 (20), 56.6 (11), 54.1 (13, 17), 25.4 (14, 16), 23.8 (15); **LR-ESI-MS**: $C_{21}H_{25}N_4O_2$ $[M+H]^+$ *m/z* found 365.58, cald 365.19; **HR-ESI-MS**: $C_{21}H_{25}N_4O_2$ $[M+H]^+$ *m/z* found 365.1960, cald 365.1978.

2-(4-chlorophenyl)-*N*-(2-(piperidin-1-ylmethyl)-1H-benzo[d]imidazol-5-yl)acetamide **105**

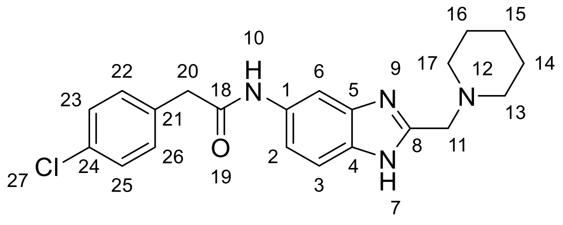

Synthesised according to general procedure **C** to give **105** (0.021 g, 0.054 mmol, 25 %) as an off white solid.

**Mpt:** 120.6-122.6 °C; **$\nu_{max}$ (cm$^{-1}$)** 2932, 1657, 1488, 1417, 1091, 804; **$^{1}$H NMR (400 MHz, DMSO-*d*$_6$) δ** 12.2 (s, 1H, 10), 10.3 – 10.0 (m, 1H, 7), 8.0 – 7.8 (m, 1H, 6), 7.5 – 7.0 (m, 6H, 2, 3, 22, 23, 25, 26), 3.6 (s, 2H, 20), 3.6 (s, 2H, 11), 2.4 (t, *J* = 5.4 Hz, 4H, 13, 17), 1.5 (p, *J* = 5.5 Hz, 4H, 14, 16), 1.4 (q, *J* = 5.8 Hz, 2H, 15); **$^{13}$C NMR (101 MHz, DMSO-*d*$_6$) δ** 168.3 (18), 152.0 (8), 139.3 (1), 135.3 (24), 134.5 (5), 133.8 (4), 131.2 (21), 131.0 (23, 25), 128.2 (22, 26), 118.2 (3), 113.8 (2), 102.0 (6), 56.6 (11), 54.1 (13, 17), 42.5 (20), 25.4 (14, 16), 23.8 (15); **LR-ESI-MS**: $C_{21}H_{24}ClN_4O$ [M+H]$^+$ *m/z* found 383.60, cald 383.16; **HR-ESI-MS**: $C_{21}H_{24}ClN_4O$ [M+H]$^+$ *m/z* found 383.1618, cald 383.1639.

*N*-(2-(piperidin-1-ylmethyl)-1H-benzo[d]imidazol-5-yl)pivalamide **106**

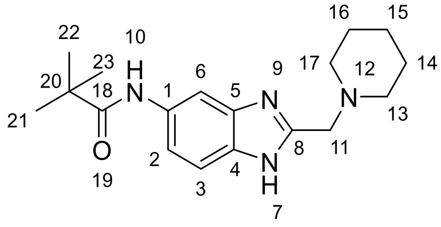


Synthesised according to general procedure **C** to give **106** (0.037 g, 0.117 mmol, 54 %) as an off white solid.

**Mpt:** 130.0-132.0 °C; **$\nu_{max}$ (cm$^{-1}$)** 2932, 1523, 1481, 1451, 1193, 806; **$^{1}$H NMR (400 MHz, DMSO-*d*$_6$) δ** 12.1 (s, 1H, 10), 9.1 (s, 1H, 7), 7.9 (d, *J* = 1.8 Hz, 1H, 6), 7.4 (d, *J* = 8.6 Hz, 1H, 3), 7.3 – 7.2 (m, 1H, 2), 3.7 (s, 2H, 11), 2.4 (s, 4H, 13, 17), 1.5 (p, *J* = 5.5 Hz, 4H, 14, 16), 1.5 – 1.3 (m, 2H, 15), 1.2 (s, 9H, 21, 22, 23); **$^{13}$C NMR (101 MHz, DMSO-*d*$_6$) δ** 176.1 (18), 56.4 (11), 54.1 (13, 17), 27.4 (14, 16), 27.0 (20), 25.2 (15), 23.6 (21, 22, 23); **LR-ESI-MS**: $C_{18}H_{27}N_4O$ [M+H]$^+$ *m/z* found 315.59, cald 315.22; **HR-ESI-MS**: $C_{18}H_{27}N_4O$ [M+H]$^+$ *m/z* found 315.2180, cald 315.2185.

3-cyano-*N*-(2-(piperidin-1-ylmethyl)-1H-benzo[d]imidazol-5-yl)benzamide **107**

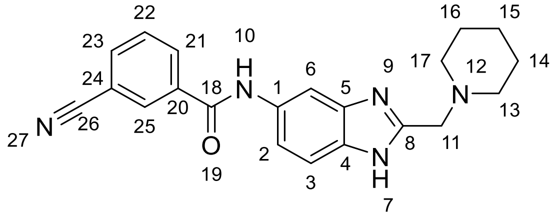


Synthesised according to general procedure **B** to give **107** (0.031 g, 0.085 mmol, 40 %) as an off white solid.

**Mpt:** 127.7-129.7 °C; **$\nu_{max}$ (cm$^{-1}$)** 2933, 1648, 1528, 1484, 1451, 1417, 805, 556; **$^{1}$H NMR (400 MHz, DMSO-*d*$_6$) δ** 12.2 (s, 1H, 10), 10.4 (s, 1H, 7), 8.4 (t, *J* = 1.8 Hz, 1H, 25), 8.3 (dt, *J* = 1.5, 8.0 Hz, 1H, 21), 8.1 – 8.0 (m, 2H, 6, 23), 7.8 (t, *J* = 7.8 Hz, 1H, 22), 7.5 (t, *J* = 7.3 Hz, 2H, 2, 3), 3.7 (s, 2H, 11), 2.5 (s, 4H, 13, 17), 1.5 (p, *J* = 5.5 Hz, 4H, 14, 16), 1.4 (q, *J* = 5.9 Hz, 2H, 15); **$^{13}$C NMR (101 MHz, DMSO-*d*$_6$) δ** 163.3 (18), 136.2 (25), 134.8 (23), 132.5 (21), 131.3 (22), 129.8 (20), 118.4 (26), 111.5 (3, 6), 56.4 (11), 54.1 (13, 17), 25.3 (14, 16), 23.6

(15); **LR-ESI-MS**: $C_{21}H_{22}N_5O$ [M+H]$^+$ *m/z* found 360.52, cald 360.18; **HR-ESI-MS**: $C_{21}H_{22}N_5O$ [M+H]$^+$ *m/z* found 360.1815, cald 360.1824.

4-cyano-*N*-(2-(piperidin-1-ylmethyl)-1H-benzo[d]imidazol-5-yl)benzamide **108**

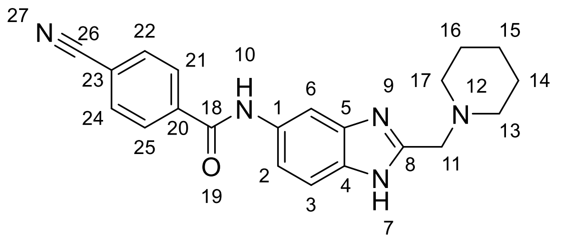

Synthesised according to general procedure **B** to give **108** (0.010 g, 0.028 mmol, 13 %) as a white solid.

**Mpt:** 133.1-135.1 °C; **$\nu_{max}$ (cm$^{-1}$)** 2931, 1647, 1529, 1450, 1297, 1108, 808, 756, 621; **$^1$H NMR (400 MHz, DMSO-*d*$_6$) δ** 12.3 (s, 1H, 7), 10.5 (s, 1H, 10), 8.2 – 8.1 (m, 2H, 21, 25), 8.1 – 8.0 (m, 1H, 6), 8.0 – 8.0 (m, 2H, 22, 24), 7.6 – 7.3 (m, 2H, 2, 3), 3.6 (s, 2H, 11), 2.4 (t, *J* = 5.3 Hz, 4H, 13, 17), 1.5 (p, *J* = 5.5 Hz, 4H, 14, 16), 1.4 (dt, *J* = 4.7, 11.3 Hz, 2H, 15); **$^{13}$C NMR (101 MHz, DMSO-*d*$_6$) δ** 163.8 (18), 139.3 (20), 132.5 (21, 25), 128.5 (22, 24), 118.4 (23), 113.6 (26), 56.7 (11), 54.2 (13, 17), 25.5 (14, 16), 23.8 (15); **LR-ESI-MS**: $C_{21}H_{22}N_5O$ [M+H]$^+$ *m/z* found 360.64, cald 360.18; **HR-ESI-MS**: $C_{21}H_{22}N_5O$ [M+H]$^+$ *m/z* found 360.1811, cald 360.1824.

*N*-(2-(piperidin-1-ylmethyl)-1H-benzo[d]imidazol-5-yl)-4-(trifluoromethyl)benzamide **109**

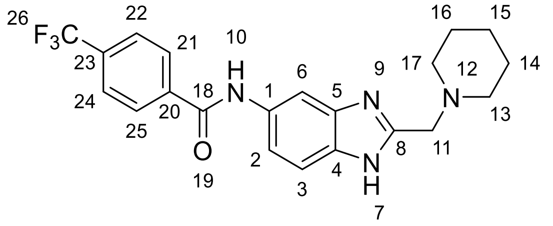

Synthesised according to general procedure **C** to give **109** (0.016 g, 0.041 mmol, 19 %) as an orange solid.

**Mpt:** 146.6-148.6 °C; **$\nu_{max}$ (cm$^{-1}$)** 2920, 2850, 1648, 1533, 1323, 1064, 855, 767, 689; **$^{19}$F NMR (376 MHz, DMSO-*d*$_6$) δ** -61.3; **$^1$H NMR (400 MHz, DMSO-*d*$_6$) δ** 12.2 (s, 1H, 7), 10.5 (s, 1H, 10), 8.2 (d, *J* = 8.1 Hz, 2H, 22, 24), 8.1 (s, 1H, 6), 7.9 (d, *J* = 8.1 Hz, 2H, 21, 25), 7.5 (s, 2H, 2, 3), 3.7 (s, 2H, 11), 2.5 – 2.4 (m, 4H, 13, 17), 1.5 (q, *J* = 5.6 Hz, 4H, 14, 16), 1.4 (q, *J* = 4.9, 5.6 Hz, 2H, 15); **$^{13}$C NMR (101 MHz, DMSO-*d*$_6$) δ** 164.1 (18), 139.1 (20), 131.3 (2), 131.0 (3), 128.6 (21, 22, 24, 25), 125.4 (q, *J* = 3.8 Hz, 26), 122.6 (6), 56.3 (11), 54.1 (13, 17), 25.2 (14, 16), 23.6 (15); **LR-ESI-MS**: $C_{21}H_{22}F_3N_4O$ [M+H]$^+$ *m/z* found 403.37, cald 403.18; **HR-ESI-MS**: $C_{21}H_{22}F_3N_4O$ [M+H]$^+$ *m/z* found 403.1733, cald 403.1746.

*N*-(2-(piperidin-1-ylmethyl)-1H-benzo[d]imidazol-5-yl)-4-(trifluoromethoxy)benzamide **110**

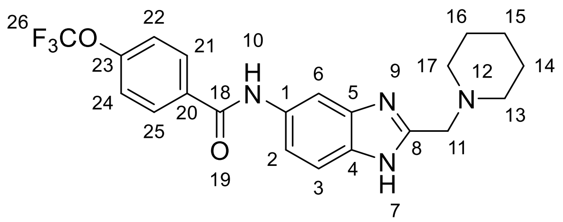

Synthesised according to general procedure **C** to give **110** (0.029 g, 0.069 mmol, 32 %) as an off white solid.

**Mpt:** 187.0-189.0 °C; **$\nu_{max}$ ($cm^{-1}$)** 2936, 1647, 1485, 1436, 1206, 1159, 806; **$^{19}$F NMR (376 MHz, DMSO-*d*$_6$) δ** -56.7; **$^1$H NMR (400 MHz, DMSO-*d*$_6$) δ** 12.2 (s, 1H, 7), 10.3 (d, *J* = 22.1 Hz, 1H, 10), 8.2 – 7.9 (m, 3H, 3, 21, 25), 7.7 – 7.2 (m, 4H, 2, 6, 22, 24), 3.6 (s, 2H, 11), 2.4 (t, *J* = 5.4 Hz, 4H, 13, 17), 1.5 (p, *J* = 5.6 Hz, 4H, 14, 16), 1.4 (q, *J* = 4.6, 6.0 Hz, 2H, 15); **$^{13}$C NMR (101 MHz, DMSO-*d*$_6$) δ** 164.1 (18), 152.3 (23), 150.3 (8), 134.4 (5), 130.0 (21, 25), 120.7 (22, 24), 118.7 (3), 118.1 (2), 115.0 (27), 103.3 (6), 56.7 (11), 54.2 (13, 17), 25.5 (14, 16), 23.8 (15); **LR-ESI-MS**: $C_{21}H_{22}F_3N_4O_2$ $[M+H]^+$ *m/z* found 419.39, cald 419.17; **HR-ESI-MS**: $C_{21}H_{22}F_3N_4O_2$ $[M+H]^+$ *m/z* found 419.1676, cald 419.1695.

2-fluoro-*N*-(2-(piperidin-1-ylmethyl)-1H-benzo[d]imidazol-5-yl)-4-(trifluoromethyl)benzamide **111**

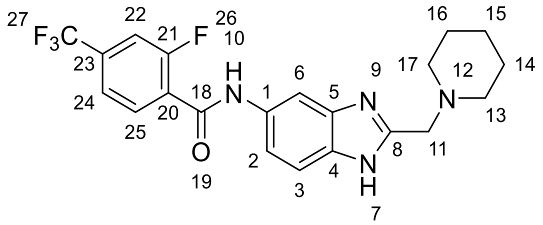

Synthesised according to general procedure **C** to give **111** (0.02 g, 0.048 mmol, 22 %) as a white solid.

**Mpt:** 117.0-119.0 °C; **$\nu_{max}$ ($cm^{-1}$)** 2934, 1656, 11421, 1327, 1125, 919, 745; **$^{19}$F NMR (376 MHz, DMSO-*d*$_6$) δ** -61.4 , 28, 29, 30, -112.5 , 26; **$^1$H NMR (400 MHz, DMSO-*d*$_6$) δ** 12.3 (s, 1H, 7), 10.6 (s, 1H, 10), 8.0 (d, *J* = 27.0 Hz, 1H, 6), 8.0 – 7.8 (m, 2H, 22, 24), 7.7 (dd, *J* = 1.7, 8.2 Hz, 1H, 25), 7.6 – 7.2 (m, 2H, 2, 3), 3.6 (s, 2H, 11), 2.4 (t, *J* = 5.3 Hz, 4H, 13, 17), 1.5 (q, *J* = 5.6 Hz, 4H, 14, 16), 1.4 (q, *J* = 6.5, 7.9 Hz, 2H, 15); **$^{13}$C NMR (101 MHz, DMSO-*d*$_6$) δ** 161.2 (18), 158.6 (d, *J* = 250.6 Hz, 21), 131.2 (d, *J* = 3.5 Hz, 25), 129.4 (d, *J* = 16.4 Hz, 20), 124.5 (d, *J* = 2.3 Hz, 24), 121.6 (q, *J* = 3.6 Hz, 27), 114.0 (d, *J* = 3.6 Hz, 22), 113.7 (d, *J* = 3.6 Hz, 23), 56.6 (11), 54.2 (13, 17), 25.5 (14, 16), 23.8 (15); **LR-ESI-MS**: $C_{21}H_{21}F_4N_4O$ $[M+H]^+$ *m/z* found 421.36, cald 421.17; **HR-ESI-MS**: $C_{21}H_{21}F_4N_4O$ $[M+H]^+$ *m/z* found 421.1630, cald 421.1651.

3-fluoro-4-methyl-*N*-(2-(piperidin-1-ylmethyl)-1H-benzo[d]imidazol-5-yl)benzamide **112**

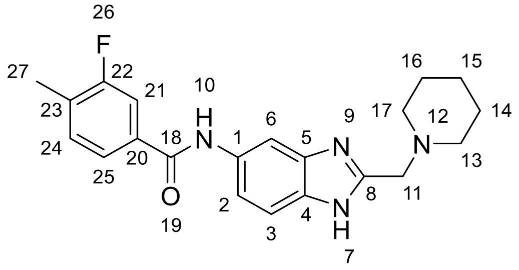


Synthesised according to general procedure **C** to give **112** (0.04 g, 0.109 mmol, 50 %) as an off white solid.

**Mpt:** 119.1-121.1 °C; **$v_{max}$ (cm$^{-1}$)** 2932, 1449, 1336, 744, 636; **$^{19}$F NMR (376 MHz, DMSO-*d*$_6$) δ** -117.1 (d, *J* = 39.4 Hz); **$^1$H NMR (400 MHz, DMSO-*d*$_6$) δ** 10.2 (s, 1H, 10), 8.0 (s, 1H, 21), 7.8 (d, *J* = 9.2 Hz, 2H, 3, 24), 7.7 – 7.5 (m, 1H, 25), 7.5 – 7.3 (m, 3H, 2, 6, 7), 3.7 (s, 2H, 11), 2.4 (t, *J* = 5.3 Hz, 4H, 13, 17), 2.3 (dd, *J* = 1.9, 9.3 Hz, 3H, 27), 1.5 (q, *J* = 5.6 Hz, 4H, 14, 16), 1.4 (q, *J* = 6.2 Hz, 2H, 15); **$^{13}$C NMR (101 MHz, DMSO-*d*$_6$) δ** 163.7 (18), 160.3 (d, *J* = 243.4 Hz, 22), 134.8 (d, *J* = 6.8 Hz, 24), 131.6 (d, *J* = 5.0 Hz, 23), 128.0 (d, *J* = 17.2 Hz, 25), 125.2 (d, *J* = 3.2 Hz, 20), 123.6 (d, *J* = 3.2 Hz, 2), 115.4 (d, *J* = 23.3 Hz, 3), 114.1 (d, *J* = 23.8 Hz, 21), 56.6 (11), 54.1 (13, 17), 25.4 (14, 16), 23.7 (15), 14.5 – 13.0 (m, 27); **LR-ESI-MS**: $C_{21}H_{24}FN_4O$ $[M+H]^+$ *m/z* found 367.39, cald 367.19; **HR-ESI-MS**: $C_{21}H_{24}FN_4O$ $[M+H]^+$ *m/z* found 367.1917, cald 367.1934.

2,4-dichloro-*N*-(2-(piperidin-1-ylmethyl)-1H-benzo[d]imidazol-5-yl)benzamide **113**

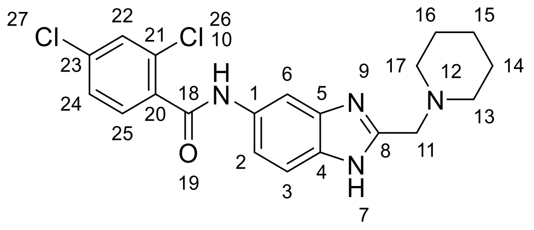


Synthesised according to general procedure **C** to give **113** (0.032 g, 0.078 mmol, 36 %) as a white solid.

**Mpt:** 139.7-141.7 °C; **$v_{max}$ (cm$^{-1}$)** 2931, 1653, 1450, 1246, 1101, 860; **$^1$H NMR (400 MHz, DMSO-*d*$_6$) δ** 12.2 (s, 1H, 10), 10.5 (d, *J* = 32.4 Hz, 1H, 7), 8.2 – 7.9 (m, 1H, 22), 7.8 (d, *J* = 2.0 Hz, 1H, 6), 7.6 (d, *J* = 8.2 Hz, 1H, 3), 7.6 (dd, *J* = 2.0, 8.2 Hz, 1H, 2), 7.5 – 7.1 (m, 2H, 24, 25), 3.6 (s, 2H, 11), 2.4 (t, *J* = 5.2 Hz, 4H, 13, 17), 1.5 (t, *J* = 5.7 Hz, 4H, 14, 16), 1.4 – 1.3 (m, 2H, 15); **$^{13}$C NMR (101 MHz, DMSO-*d*$_6$) δ** 163.7 (18), 131.3 (22), 130.4 (21, 25), 129.2 (23), 127.5 (24), 118.2 (2), 102.5 (6), 56.7 (11), 54.1 (13, 17), 25.4 (14, 16), 23.8 (15); **LR-ESI-MS**: $C_{20}H_{21}Cl_2N_4O$ $[M+H]^+$ *m/z* found 403.31, cald 403.11; **HR-ESI-MS**: $C_{20}H_{21}Cl_2N_4O$ $[M+H]^+$ *m/z* found 403.1068, cald 403.1092.

3,4-difluoro-*N*-(2-(piperidin-1-ylmethyl)-1H-benzo[d]imidazol-5-yl)benzamide **114**

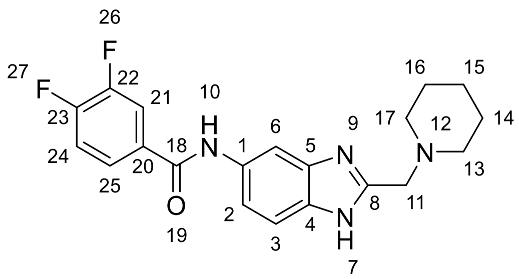

Synthesised according to general procedure **C** to give **114** (0.025 g, 0.067 mmol, 31 %) as an off white solid.

**Mpt:** 136.0-138.0 °C; **$v_{max}$ (cm$^{-1}$)** 2934, 1648, 1602, 1509, 1423, 1287, 1110, 747; **$^{19}$F NMR (376 MHz, DMSO-*d*$_6$) δ** -134.5 (dd, *J* = 22.1, 40.9 Hz, 27), -138.0 (d, *J* = 22.6 Hz, 26); **$^{1}$H NMR (400 MHz, DMSO-*d*$_6$) δ** 12.2 (s, 1H, 7), 10.3 (d, *J* = 17.8 Hz, 1H, 10), 8.1 (ddd, *J* = 2.2, 7.8, 11.6 Hz, 2H, 6, 21), 7.9 (ddt, *J* = 1.6, 3.8, 8.2 Hz, 1H, 25), 7.6 (dt, *J* = 8.4, 10.6 Hz, 1H, 24), 7.6 – 7.3 (m, 2H, 2, 3), 3.6 (s, 2H, 11), 2.4 (t, *J* = 5.3 Hz, 4H, 13, 17), 1.5 (p, *J* = 5.5 Hz, 4H, 14, 16), 1.4 (q, *J* = 4.7, 6.2 Hz, 2H, 15); **$^{13}$C NMR (101 MHz, DMSO-*d*$_6$) δ** 162.9 (18), 152.3 (8), 150.3 (dd, *J* = 12.7, 18.8 Hz, 23), 147.9 (d, *J* = 12.9 Hz, 22), 139.9 (1), 134.4 (5), 133.4 (4), 132.6 (20), 125.2 (25), 118.1 (2), 117.6 (d, *J* = 17.5 Hz, 21), 117.1 (d, *J* = 18.3 Hz, 24), 115.1 (3), 103.4 (6), 56.7 (11), 54.2 (13, 17), 25.5 (14, 16), 23.8 (15); **LR-ESI-MS**: $C_{20}H_{21}F_2N_4O$ [M+H]$^+$ *m/z* found 371.38, cald 371.17; **HR-ESI-MS**: $C_{20}H_{21}F_2N_4O$ [M+H]$^+$ *m/z* found 371.1665, cald 371.1683.

2,4-difluoro-*N*-(2-(piperidin-1-ylmethyl)-1H-benzo[d]imidazol-5-yl)benzamide **115**

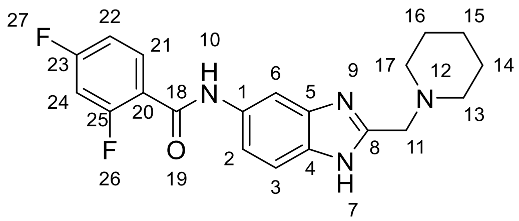

Synthesised according to general procedure **C** to give **115** (0.035 g, 0.095 mmol, 44 %) as an off white solid.

**Mpt:** 107.3-109.3 °C; **$v_{max}$ (cm$^{-1}$)** 2933, 1653, 1613, 1421, 1267, 970, 848, 807, 524; **$^{19}$F NMR (376 MHz, DMSO-*d*$_6$) δ** -106.6 (dd, *J* = 9.2, 34.9 Hz, 26), -109.9 (dd, *J* = 9.3, 25.4 Hz, 27); **$^{1}$H NMR (400 MHz, DMSO-*d*$_6$) δ** 12.2 (s, 1H, 10), 10.4 (d, *J* = 33.0 Hz, 1H, 7), 8.1 – 7.9 (m, 1H, 21), 7.8 (td, *J* = 6.6, 8.4 Hz, 1H, 24), 7.5 – 7.3 (m, 2H, 3, 22), 7.3 – 7.1 (m, 2H, 2, 6), 3.6 (s, 2H, 11), 2.4 (t, *J* = 5.3 Hz, 4H, 13, 17), 1.5 (p, *J* = 5.5 Hz, 4H, 14, 16), 1.4 – 1.3 (m, 2H, 15); **$^{13}$C NMR (101 MHz, DMSO-*d*$_6$) δ** 162.0 (18), 152.7 (25), 134.9 (5), 133.9 (21), 132.1 (dd, *J* = 4.8, 9.7 Hz), 118.7 (2), 115.9 (d, *J* = 2.6 Hz, 3), 114.8 (22), 112.3 (d, *J* = 20.0 Hz, 20), 105.1 (t, *J* = 26.1 Hz, 24), 103.1 (6), 57.1 (11), 54.6 (13, 17), 25.9 (14, 16), 24.2 (15); **LR-ESI-MS**: $C_{20}H_{21}F_2N_4O$ [M+H]$^+$ *m/z* found 371.37, cald 371.17; **HR-ESI-MS**: $C_{20}H_{21}F_2N_4O$ [M+H]$^+$ *m/z* found 371.1666, cald 371.1683.

2-methyl-*N*-(2-(piperidin-1-ylmethyl)-1H-benzo[d]imidazol-5-yl)benzamide **116**

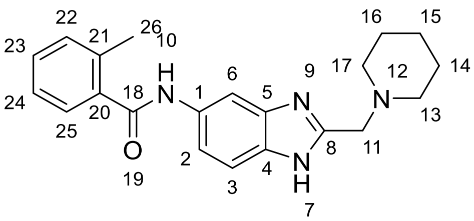


Synthesised according to general procedure **C** to give **116** (0.026 g, 0.075 mmol, 35 %) as an off white solid.

**Mpt:** 123.1-125.1 °C; **$v_{max}$ (cm$^{-1}$)** 2932, 1646, 1450, 736; **$^{1}$H NMR (400 MHz, DMSO-*d*$_6$) δ** 12.2 (s, 1H, 10), 10.2 (d, *J* = 27.3 Hz, 1H, 7), 8.1 (d, *J* = 48.7 Hz, 1H, 6), 7.8 – 7.2 (m, 6H, 2, 3, 22, 23, 24, 25), 3.6 (s, 2H, 11), 2.4 (s, 7H, 13, 17, 26), 1.5 (p, *J* = 5.5 Hz, 4H, 14, 16), 1.4 (t, *J* = 6.0 Hz, 2H, 15); **$^{13}$C NMR (101 MHz, DMSO-*d*$_6$) δ** 167.6 (18), 135.1 (1), 131.5 (5), 130.5 (21, 25), 130.1 (4), 129.4 (20), 127.2 (22, 24), 125.6 (23), 56.6 (11), 54.1 (13, 17), 25.4 (14, 16), 23.8 (15), 19.3 (26); **LR-ESI-MS**: $C_{21}H_{25}N_4O$ [M+H]$^+$ *m/z* found 349.42, cald 349.20; **HR-ESI-MS**: $C_{21}H_{25}N_4O$ [M+H]$^+$ *m/z* found 349.2009, cald 349.2028.

2-(3,4-dichlorophenyl)-*N*-(2-(piperidin-1-ylmethyl)-1H-benzo[d]imidazol-5-yl)acetamide **117**

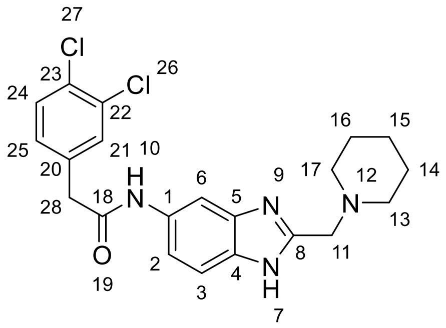


Synthesised according to general procedure **C** to give **117** (0.029 g, 0.069 mmol, 32 %) as an off white solid.

**Mpt:** 123.6-125.6 °C; **$v_{max}$ (cm$^{-1}$)** 2932, 1556, 1417, 1334, 1248, 1130, 808; **$^{1}$H NMR (400 MHz, DMSO-*d*$_6$) δ** 12.2 (s, 1H, 10), 10.1 (d, *J* = 28.6 Hz, 1H, 7), 7.9 (d, *J* = 33.0 Hz, 1H, 3), 7.7 – 7.5 (m, 2H, 24, 25), 7.5 – 7.0 (m, 3H, 2, 6, 21), 3.7 (s, 2H, 11), 3.6 (s, 2H, 28), 2.4 (s, 4H, 13, 17), 1.5 (q, *J* = 5.6 Hz, 4H, 14, 16), 1.4 (d, *J* = 7.0 Hz, 2H, 15); **$^{13}$C NMR (101 MHz, DMSO-*d*$_6$) δ** 167.8 (18), 137.3 (23), 131.3 (25), 130.7 (21), 130.4 (22), 129.7 (24), 129.2 (20), 56.6 (11), 54.1 (13, 17), 42.0 (28), 25.4 (14, 16), 23.8 (15); **LR-ESI-MS**: $C_{21}H_{23}Cl_2N_4O$ [M+H]$^+$ *m/z* found 417.36, cald 417.13; **HR-ESI-MS**: $C_{21}H_{23}Cl_2N_4O$ [M+H]$^+$ *m/z* found 417.1223, cald 417.1249.

1-methyl-*N*-(2-(piperidin-1-ylmethyl)-1H-benzo[d]imidazol-5-yl)-1H-benzo[d]imidazole-6-carboxamide **118**

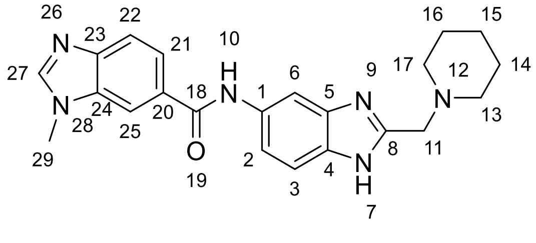

Synthesised according to general procedure **B** to give **118** (0.052 g, 0.135 mmol, 62 %) as an off white solid.

**Mpt:** 150.2-152.2 °C; **$v_{max}$ (cm$^{-1}$)** 2932, 1642, 1527, 1415, 1248, 1220, 840, 732, 434; **$^1$H NMR (400 MHz, DMSO-*d*$_6$) δ** 12.2 (s, 1H, 10), 10.3 (d, *J* = 20.0 Hz, 1H, 7), 8.3 (q, *J* = 1.1 Hz, 1H, 27), 8.2 – 8.0 (m, 2H, 21, 25), 7.9 (dd, *J* = 0.8, 8.4 Hz, 1H, 3), 7.7 (dd, *J* = 1.4, 8.4 Hz, 1H, 6), 7.5 (dd, *J* = 8.4, 31.0 Hz, 2H, 2), 4.1 (s, 3H, 29), 3.7 (s, 2H, 11), 2.4 (d, *J* = 5.5 Hz, 4H, 13, 17), 1.5 (q, *J* = 5.6 Hz, 4H, 14, 16), 1.4 (d, *J* = 7.5 Hz, 2H, 15); **$^{13}$C NMR (101 MHz, DMSO-*d*$_6$) δ** 165.5 (18), 139.1 (27), 133.0 (20), 132.5 (21), 124.9 (22), 120.7 (2), 119.7 (3), 118.0 (25), 109.6 (6), 56.7 (11), 54.2 (13, 17), 35.6 (29), 25.5 (14, 16), 23.8 (15); **LR-ESI-MS**: $C_{22}H_{25}N_6O$ $[M+H]^+$ *m/z* found 389.42, cald 389.21; **HR-ESI-MS**: $C_{22}H_{25}N_6O$ $[M+H]^+$ *m/z* found 389.2071, cald 389.2090.

3,4,5-trimethoxy-*N*-(2-(piperidin-1-ylmethyl)-1H-benzo[d]imidazol-5-yl)benzamide **119**

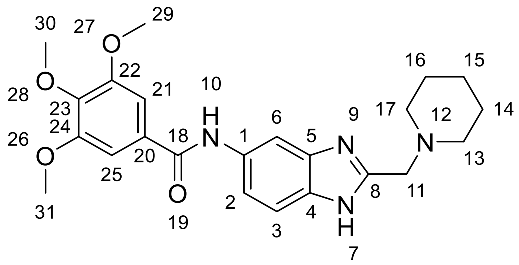

Synthesised according to general procedure **B** to give **119** (0.039 g, 0.091 mmol, 42 %) as an off white solid.

**Mpt:** 134.2-136.2 °C; **$v_{max}$ (cm$^{-1}$)** 2934, 1582, 1331, 1225, 998, 843, 727; **$^1$H NMR (400 MHz, DMSO-*d*$_6$) δ** 12.2 (s, 1H, 7), 10.1 (s, 1H, 10), 8.0 (s, 1H, 6), 7.3 (d, *J* = 27.7 Hz, 4H, 2, 3, 21, 25), 3.9 (s, 6H, 29, 31), 3.7 (d, *J* = 3.5 Hz, 3H, 30), 3.7 (s, 2H, 11), 2.4 (s, 4H, 13, 17), 1.5 (t, *J* = 5.7 Hz, 4H, 14, 16), 1.4 – 1.4 (m, 2H, 15); **$^{13}$C NMR (101 MHz, DMSO-*d*$_6$) δ** 164.6 (18), 152.6 (8), 140.1 (23), 130.3 (20), 106.5 (21, 25), 105.2 (2, 6), 60.1 (11), 56.1 (13, 17), 55.9 (30), 54.2 (29, 31), 25.4 (14, 16), 23.7 (15); **LR-ESI-MS**: $C_{23}H_{29}N_4O_4$ $[M+H]^+$ *m/z* found 425.48, cald 425.22; **HR-ESI-MS**: $C_{23}H_{29}N_4O_4$ $[M+H]^+$ *m/z* found 425.2164, cald 425.2189.

3-iodo-4-methyl-*N*-(2-(piperidin-1-ylmethyl)-1H-benzo[d]imidazol-5-yl)benzamide **120**

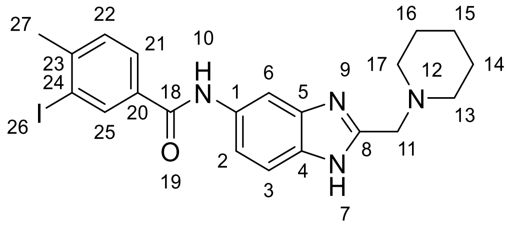

Synthesised according to general procedure **B** to give **120** (0.057 g, 0.121 mmol, 56 %) as a white solid.

**Mpt:** 156.5-158.5 °C; **$v_{max}$ (cm$^{-1}$)** 2932, 1642, 1529, 1248,746, 556; **$^1$H NMR (400 MHz, DMSO-*d*$_6$) δ** 12.2 (s, 1H, 7), 10.2 (s, 1H, 10), 8.5 – 8.3 (m, 1H, 25), 8.0 (s, 1H, 3), 8.0 – 7.8 (m, 1H, 21), 7.6 – 7.3 (m, 3H, 2, 6, 22), 3.7 (s, 2H, 11), 2.5 – 2.3 (m, 7H, 13, 17, 27), 1.5 (p, *J* = 5.5 Hz, 4H, 14, 16), 1.4 (q, *J* = 6.0 Hz, 2H, 15); **$^{13}$C NMR (101 MHz, DMSO-*d*$_6$) δ** 163.4

(18), 144.4 (25), 139.2 (5), 137.4 (21), 134.3 (23), 130.0 (4), 129.7 (20), 129.2 (22), 127.7 (2, 6), 101.1 (24), 56.6 (11), 54.1 (13, 17), 27.5 (27), 25.4 (14, 16), 23.7 (15); **LR-ESI-MS**: $C_{21}H_{24}IN_4O$ $[M+H]^+$ *m/z* found 475.30, cald 475.09; **HR-ESI-MS**: $C_{21}H_{24}IN_4O$ $[M+H]^+$ *m/z* found 475.0960, cald 475.0995.

*N*-(2-(piperidin-1-ylmethyl)-1H-benzo[d]imidazol-5-yl)-2-(pyridin-4-yl)acetamide **121**

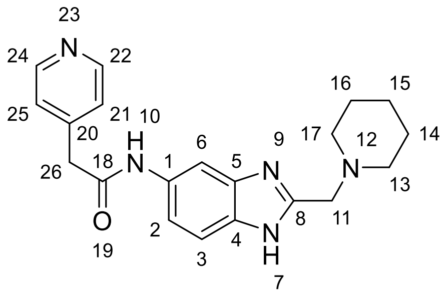


Synthesised according to general procedure **B** to give **121** (0.039 g, 0.112 mmol, 52 %) as a yellow solid.

**Mpt:** 141.5-143.5 °C; $\mathbf{v_{max}}$ **(cm**$^{-1}$**)** 2932, 1600, 1418, 1125, 765; $^1$**H NMR (400 MHz, DMSO-*d*$_6$) δ** 10.4 (s, 1H, 7), 8.6 – 8.2 (m, 3H, 3, 22, 24), 7.9 (d, *J* = 1.9 Hz, 1H, 10), 7.5 – 7.2 (m, 4H, 2, 6, 21, 25), 3.7 (s, 2H, 26), 3.7 (s, 2H, 11), 2.4 (t, *J* = 5.2 Hz, 4H, 13, 17), 1.5 (t, *J* = 5.7 Hz, 4H, 14, 16), 1.4 – 1.3 (m, 2H, 15); $^{13}$**C NMR (101 MHz, DMSO-*d*$_6$) δ** 167.4 (18), 151.9 (8), 149.5 (22, 24), 148.0 (5), 145.1 (20), 139.5 (1), 134.5 (4), 133.6 (6), 127.6 (2), 124.7 (21, 25), 119.3 (3), 56.4 (11), 54.0 (13, 17), 42.5 (26), 25.3 (14, 16), 23.6 (15); **LR-ESI-MS**: $C_{20}H_{24}N_5O$ $[M+H]^+$ *m/z* found 350.40, cald 350.19; **HR-ESI-MS**: $C_{20}H_{24}N_5O$ $[M+H]^+$ *m/z* found 350.1957, cald 350.1981.

*N*-(2-(piperidin-1-ylmethyl)-1H-benzo[d]imidazol-5-yl)-2-(trifluoromethoxy)benzamide **122**

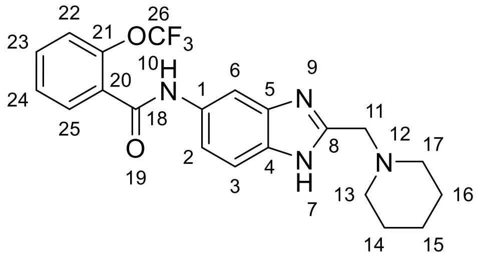


Synthesised according to general procedure **B** to give **122** (0.038 g, 0.091 mmol, 42 %) as a white solid.

**Mpt:** 107.3-109.3 °C; $\mathbf{v_{max}}$ **(cm**$^{-1}$**)** 2938, 1603, 1448, 1247, 842, 761; $^{19}$**F NMR (376 MHz, DMSO-*d*$_6$) δ** -56.5; $^1$**H NMR (400 MHz, DMSO-*d*$_6$) δ** 12.3 (s, 1H, 7), 10.4 (s, 1H, 10), 8.0 (s, 1H, 6), 7.8 (ddd, *J* = 1.8, 7.6, 51.2 Hz, 1H, 22), 7.7 – 7.6 (m, 1H, 23), 7.6 – 7.4 (m, 3H, 2, 3, 25), 7.3 (d, *J* = 8.8 Hz, 1H, 24), 3.7 (s, 2H, 11), 2.4 (t, *J* = 5.3 Hz, 4H, 13, 17), 1.5 (p, *J* = 5.5 Hz, 4H, 14, 16), 1.4 (t, *J* = 6.0 Hz, 2H, 15); $^{13}$**C NMR (101 MHz, DMSO-*d*$_6$) δ** 163.0 (18), 152.4 (21), 144.9 – 144.7 (m, 27), 131.6 (23), 131.4 (25), 129.8 (20), 127.7 (24), 127.6 (4), 122.4 (2), 121.7 (22), 121.3 (3), 118.8 (6), 56.5 (11), 54.1 (13, 17), 25.4 (14, 16), 23.7 (15); **LR-ESI-MS**: $C_{21}H_{22}F_3N_4O_2$ $[M+H]^+$ *m/z* found 419.60, cald 419.17; **HR-ESI-MS**: $C_{21}H_{22}F_3N_4O_2$ $[M+H]^+$ *m/z* found 419.1668, cald 419.1695.

4-iodo-*N*-(2-(piperidin-1-ylmethyl)-1H-benzo[d]imidazol-5-yl)benzamide **123**

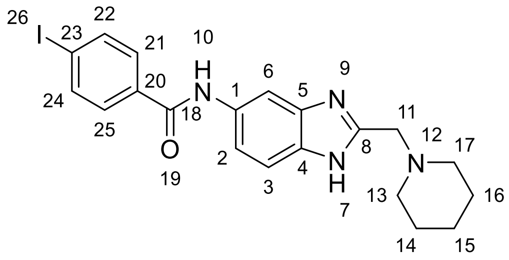

Synthesised according to general procedure **B** to give **123** (0.027 g, 0.059 mmol, 27 %) as a white solid.

**Mpt:** 198.5-200.5 °C; **$\nu_{max}$ (cm$^{-1}$)** 2930, 2796, 1643, 1585, 1526, 1298, 1107, 1005, 807; **$^{1}$H NMR (400 MHz, DMSO-*d*$_6$) δ** 12.2 (s, 1H, 10), 10.3 (d, *J* = 21.2 Hz, 1H, 7), 8.0 (d, *J* = 34.1 Hz, 1H, 6), 7.9 – 7.9 (m, 2H, 21, 25), 7.8 – 7.7 (m, 2H, 22, 24), 7.5 – 7.3 (m, 2H, 2, 3), 3.6 (s, 2H, 11), 2.5 – 2.3 (m, 4H, 13, 17), 1.5 (p, *J* = 5.6 Hz, 4H, 14, 16), 1.5 – 1.3 (m, 2H, 15); **$^{13}$C NMR (101 MHz, DMSO-*d*$_6$) δ** 164.5 (18), 152.2 (8), 137.2 (21, 25), 134.6 (5), 129.6 (22, 24), 118.0 (2), 115.0 (3), 56.7 (11), 54.2 (13, 17), 25.4 (14, 16), 23.8 (15); **LR-ESI-MS**: $C_{20}H_{22}IN_4O$ $[M+H]^+$ *m/z* found 461.43, cald 461.08; **HR-ESI-MS**: $C_{20}H_{22}IN_4O$ $[M+H]^+$ *m/z* found 461.0805, cald 461.0838.

4-chloro-*N*-(2-((1,2,4,5-tetrahydro-3*H*-benzo[*d*]azepin-3-yl)methyl)-1*H*-benzo[*d*]imidazol-5-yl)benzamide **124**

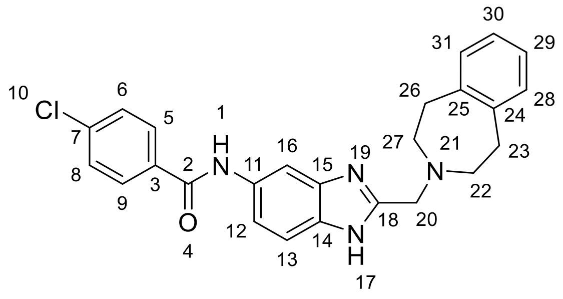

Synthesised according to general procedure **C** to give **124** (0.089 g, 0.200 mmol, 53 %) as a yellow solid.

**Mpt:** 151.7-153.7 °C; **$\nu_{max}$ (cm$^{-1}$)** 2929, 2812, 1643, 1596, 1433, 1302, 1269, 1089, 1013, 842, 807, 747; **$^{1}$H NMR (400 MHz, DMSO-*d*$_6$) δ** 12.3 (s, 1H, 1), 10.3 (s, 1H, 17), 8.1 (s, 1H, 16), 8.0 – 8.0 (m, 2H, 5, 9), 7.7 – 7.6 (m, 2H, 6, 8), 7.6 – 7.4 (m, 2H, 12, 13), 7.1 – 7.0 (m, 4H, 28, 29, 30, 31), 3.9 (s, 2H, 20), 2.9 (dd, *J* = 3.4, 6.7 Hz, 4H, 23, 26), 2.7 – 2.6 (m, 4H, 22, 27); **$^{13}$C NMR (101 MHz, DMSO-*d*$_6$) δ** 164.1 (2), 152.4 (18), 141.9 (24, 25), 136.2 (11), 133.9 (7), 129.6 (5, 9), 128.7 (6, 8), 128.4 (28, 29, 30, 31), 126.1 (3), 118.1 (12), 115.1 (13), 103.3 (16), 56.3 (20), 55.2 (22, 27), 35.7 (23, 26); **LR-ESI-MS**: $C_{25}H_{24}ClN_4O$ $[M+H]^+$ *m/z* found 431.53, cald 431.16; **HR-ESI-MS**: $C_{25}H_{24}ClN_4O$ $[M+H]^+$ *m/z* found 431.1616, cald 431.1639.

4-chloro-*N*-(2-((4-phenylpiperazin-1-yl)methyl)-1H-benzo[d]imidazol-5-yl)benzamide **125**

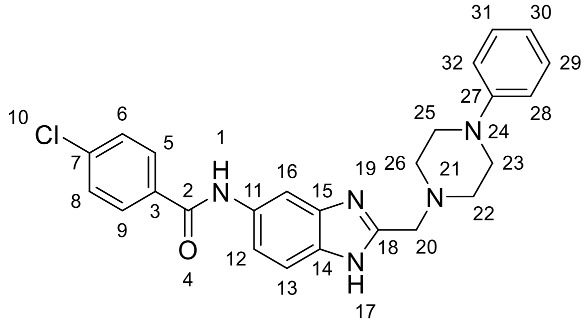

Synthesised according to general procedure **C** to give **125** (0.089 g, 0.200 mmol, 51 %) as a yellow solid.

**Mpt:** 134.3-136.3 °C; **$\nu_{max}$ (cm$^{-1}$)** 2812, 1647, 1596, 1448, 1333, 1227, 1089, 1013, 749; **$^1$H NMR (400 MHz, DMSO-*d*$_6$) δ** 12.4 (s, 1H, 1), 10.3 (s, 1H, 17), 8.1 (s, 1H, 16), 8.0 – 8.0 (m, 2H, 5, 9), 7.7 – 7.6 (m, 2H, 6, 8), 7.5 – 7.4 (m, 2H, 12, 13), 7.3 – 7.1 (m, 2H, 29, 31), 7.0 – 6.9 (m, 2H, 28, 32), 6.8 – 6.7 (m, 1H, 30), 3.8 (s, 2H, 20), 3.3 – 3.1 (m, 4H, 22, 26), 2.6 (t, *J* = 4.9 Hz, 4H, 23, 25); **$^{13}$C NMR (101 MHz, DMSO-*d*$_6$) δ** 164.2 (2), 151.0 (18), 136.2 (3, 7), 133.9 (14, 15), 129.6 (5, 9), 128.9 (29, 31), 128.4 (6, 8), 118.8 (12, 13), 115.4 (28, 30, 32), 55.8 (20), 52.8 (22, 26), 48.1 (23, 25); **LR-ESI-MS**: $C_{25}H_{25}ClN_5O$ [M+H]$^+$ *m/z* found 446.56, cald 446.18; **HR-ESI-MS**: $C_{25}H_{25}ClN_5O$ [M+H]$^+$ *m/z* found 446.1717, cald 446.1748.

4-chloro-*N*-(2-((4-(2-(pyrrolidin-1-yl)ethyl)piperidin-1-yl)methyl)-1H-benzo[d]imidazol-5-yl)benzamide **126**

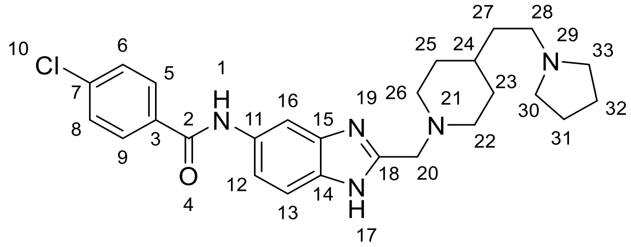

Synthesised according to general procedure **C** to give **126** (0.044 g, 0.094 mmol, 36 %) as a white solid.

**Mpt:** 145.9-147.9 °C; **$\nu_{max}$ (cm$^{-1}$)** 2917, 2795, 1646, 1596, 1419, 1273, 1089, 843, 807, 749; **$^1$H NMR (400 MHz, DMSO-*d*$_6$) δ** 12.2 (s, 1H), 10.3 (s, 1H), 8.1 – 7.9 (m, 3H), 7.7 – 7.6 (m, 2H), 7.6 – 7.3 (m, 2H), 3.7 (s, 2H, 20), 2.9 – 2.7 (m, 2H, 28), 2.4 (dt, *J* = 4.2, 7.6 Hz, 6H, 22, 23', 25', 26), 2.0 (td, *J* = 2.3, 11.4 Hz, 2H, 23'', 25''), 1.6 (dt, *J* = 5.6, 10.6 Hz, 6H, 27, 30, 33), 1.5 – 1.1 (m, 5H, 24, 31, 32); **$^{13}$C NMR (101 MHz, DMSO-*d*$_6$) δ** 164.1 (2), 136.2 (15), 133.9 (14), 129.6 (5, 9), 128.4 (6, 8), 56.3 (20), 53.7 (22, 26), 53.6 (30, 33), 53.2 (28), 35.2 (23, 25), 33.2 (24), 32.0 (31, 32), 23.0 (27); **LR-ESI-MS**: $C_{26}H_{33}ClN_5O$ [M+H]$^+$ *m/z* found 466.63, cald 466.24; **HR-ESI-MS**: $C_{26}H_{33}ClN_5O$ [M+H]$^+$ *m/z* found 466.2239, cald 466.2374.

4-chloro-*N*-(2-((4-(2-(piperidin-1-yl)ethyl)piperidin-1-yl)methyl)-1H-benzo[d]imidazol-5-yl)benzamide **127**

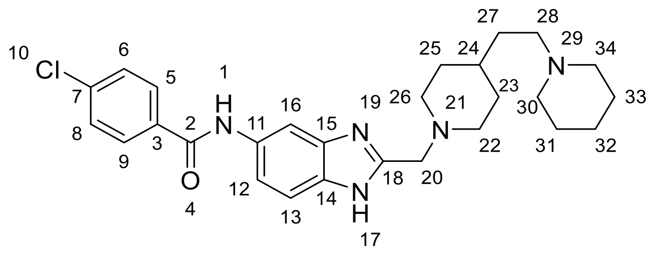


Synthesised according to general procedure **C** to give **127** (0.065 g, 0.135 mmol, 52 %) as a white solid.

**Mpt:** 115.4-117.4 °C; $\mathbf{v_{max}}$ **(cm$^{-1}$)** 2930, 2806, 1646, 1595, 1528, 1449, 1417, 1296, 1098, 1012, 808, 749, 624; **$^1$H NMR (400 MHz, DMSO-*d*$_6$) δ** 12.3 (s, 1H, 1), 10.3 (d, *J* = 19.6 Hz, 1H, 17), 8.1 (s, 1H, 16), 8.0 – 7.8 (m, 2H, 5, 9), 7.6 (d, *J* = 8.6 Hz, 2H, 6, 8), 7.5 (d, *J* = 7.7 Hz, 1H, 12), 7.4 – 7.3 (m, 1H, 13), 3.7 (s, 2H, 20), 2.5 – 2.1 (m, 17H, 22, 24, 26, 27, 28, 30, 31, 33, 34), 1.4 (p, *J* = 5.5 Hz, 4H, 23, 25), 1.3 (q, *J* = 5.9 Hz, 2H, 32); **$^{13}$C NMR (101 MHz, DMSO-*d*$_6$) δ** 164.1 (2), 151.9 (18), 136.2 (15), 133.9 (14), 129.6 (5, 9), 128.4 (6, 8), 118.1 (12), 115.1 (13), 103.3 (16), 56.2 (20), 55.9 (28), 55.6 (30, 34), 54.5 (22, 26), 53.0 (31, 33), 53.0 (23, 25), 25.6 (24, 27), 24.1 (32); **LR-ESI-MS**: $C_{27}H_{35}ClN_5O$ $[M+H]^+$ *m/z* found 481.64, cald 480.25; **HR-ESI-MS**: $C_{27}H_{35}ClN_5O$ $[M+H]^+$ *m/z* found 481.2453, cald 480.2530.

4-chloro-*N*-(2-((2-methylpyrrolidin-1-yl)methyl)-1H-benzo[d]imidazol-5-yl)benzamide **128**

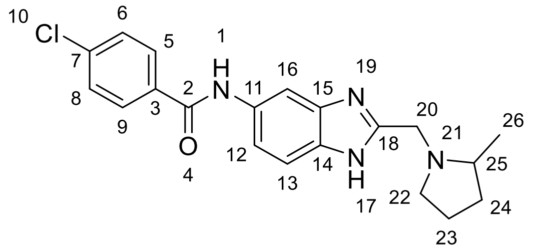


Synthesised according to general procedure **C** to give **128** (0.105 g, 0.284 mmol, 73 %) as an off white solid.

**Mpt:** 125.8-127.8 °C; $\mathbf{v_{max}}$ **(cm$^{-1}$)** 2959, 2792, 1593, 1449, 1274, 1089, 1012, 808, 749, 528; **$^1$H NMR (400 MHz, DMSO-*d*$_6$) δ** 12.3 (s, 1H, 1), 10.3 (s, 1H, 17), 8.1 – 7.9 (m, 3H, 5, 9, 16), 7.7 – 7.5 (m, 2H, 6, 8), 7.5 (s, 2H, 12, 13), 4.1 (d, *J* = 14.2 Hz, 1H, 20''), 3.6 (d, *J* = 14.2 Hz, 1H, 20'), 3.0 (ddd, *J* = 3.5, 7.5, 9.2 Hz, 1H, 25), 2.6 – 2.5 (m, 1H, 22'), 2.3 (q, *J* = 8.8 Hz, 1H, 22''), 1.9 (dddd, *J* = 5.5, 7.1, 9.3, 12.4 Hz, 1H, 23''), 1.7 (dtd, *J* = 3.0, 5.5, 6.4, 11.7 Hz, 2H, 23', 24''), 1.4 (dddd, *J* = 6.4, 8.4, 10.0, 12.2 Hz, 1H, 24'), 1.1 (d, *J* = 6.0 Hz, 3H, 26); **$^{13}$C NMR (101 MHz, DMSO-*d*$_6$) δ** 164.1 (2), 152.8 (18), 137.5 (11), 136.2 (12), 133.9 (16), 133.3 (14, 15), 131.1 (7), 129.6 (5, 9), 128.6 (3), 128.4 (6, 8), 115.6 (13), 59.2 (20), 54.0 (22), 50.8 (25), 32.4 (24), 21.4 (23), 18.7 (26); **LR-ESI-MS**: $C_{20}H_{22}ClN_4O$ $[M+H]^+$ *m/z* found 369.49, cald 369.15; **HR-ESI-MS**: $C_{20}H_{22}ClN_4O$ $[M+H]^+$ *m/z* found 369.1460, cald 369.1482.

4-chloro-*N*-(2-((4-methylpiperazin-1-yl)methyl)-1H-benzo[d]imidazol-5-yl)benzamide **129**

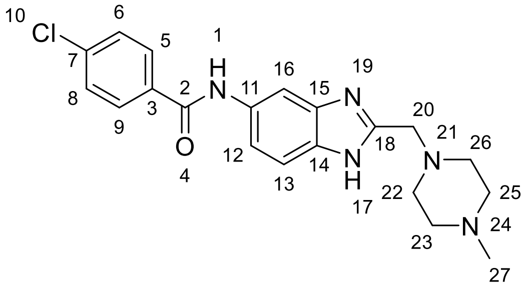

Synthesised according to general procedure **C** to give **129** (0.082 g, 0.214 mmol, 55 %) as a white solid.

**Mpt:** 158.9-160.9 °C; **v$_{max}$ (cm$^{-1}$)** 2936, 2799, 1644, 1596, 1530, 1483, 1452, 1280, 1089, 1012, 810, 749; **$^{1}$H NMR (400 MHz, DMSO-*d*$_6$) δ** 12.3 (s, 1H, 1), 10.3 (s, 1H, 17), 8.1 – 7.9 (m, 3H, 5, 9, 16), 7.6 – 7.6 (m, 2H, 6, 8), 7.5 – 7.3 (m, 2H, 12, 13), 3.7 (s, 2H, 20), 2.5 – 2.2 (m, 8H, 22, 23, 25, 26), 2.2 (s, 3H, 27); **$^{13}$C NMR (101 MHz, DMSO-*d*$_6$) δ** 164.1 (2), 151.9 (18), 139.8 (7), 136.2 (15), 133.9 (14), 129.6 (5, 9), 128.4, 118.1 (12), 115.1 (13), 110.7 (16), 103.3 (11), 55.8 (20), 54.6 (22, 26), 52.8 (23, 25), 45.8 (27); **LR-ESI-MS**: $C_{20}H_{23}ClN_5O$ $[M+H]^+$ *m/z* found 384.32, cald 384.16; **HR-ESI-MS**: $C_{20}H_{23}ClN_5O$ $[M+H]^+$ *m/z* found 384.1562, cald 384.1591.

4-chloro-*N*-(2-((dimethylamino)methyl)-1H-benzo[d]imidazol-5-yl)benzamide **130**

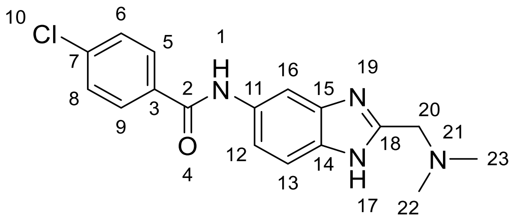

Synthesised according to general procedure **C** to give **130** (0.031 g, 0.094 mmol, 24 %) as an off white solid.

**Mpt:** 143.2-145.2 °C; **v$_{max}$ (cm$^{-1}$)** 2822, 2778, 1644, 1595, 1529, 1483, 1344, 1259, 1090, 1013, 842, 810, 741, 619; **$^{1}$H NMR (400 MHz, DMSO-*d*$_6$) δ** 12.3 (s, 1H, 1), 10.4 – 10.2 (m, 1H, 17), 8.1 – 7.9 (m, 3H, 5, 9, 16), 7.7 – 7.6 (m, 2H, 6, 8), 7.5 – 7.3 (m, 2H, 12, 13), 3.6 (s, 2H, 20), 2.2 (s, 6H, 22, 23); **$^{13}$C NMR (101 MHz, DMSO-*d*$_6$) δ** 164.1 (2), 152.5 (18), 139.8 (7), 136.2 (3), 134.4 (11), 133.9 (15), 133.6 (14), 129.6 (5, 9), 128.4 (6, 8), 118.1 (12), 115.1 (13), 103.3 (16), 57.1 (20), 45.2 (22, 23)s; **LR-ESI-MS**: $C_{17}H_{18}ClN_4O$ $[M+H]^+$ *m/z* found 329.25, cald 329.12; **HR-ESI-MS**: $C_{17}H_{18}ClN_4O$ $[M+H]^+$ *m/z* found 329.1144, cald 329.1169.

4-(benzyloxy)-*N*-(2-(piperidin-1-ylmethyl)-1H-benzo[d]imidazol-5-yl)benzamide **131**

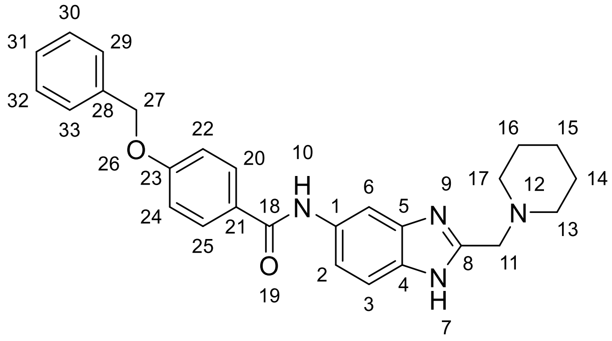

Synthesised according to general procedure **B** to give **131** (0.056 g, 0.127 mmol, 59 %) as a white solid.

**Mpt:** 197.2-199.2 °C; $\mathbf{v_{max}}$ **(cm$^{-1}$)** 3242, 2932, 1642, 1603, 1548, 1507, 1408, 1313, 1244, 1175, 997, 839, 753, 697, 557; **$^{1}$H NMR (400 MHz, DMSO-*d*$_6$) δ** 12.2 (s, 1H, 10), 10.1 (s, 1H, 7), 8.0 (s, 1H, 6), 8.0 – 7.9 (m, 2H, 20, 25), 7.6 – 7.3 (m, 7H, 2, 3, 29, 30, 31, 32, 33), 7.1 (d, *J* = 8.9 Hz, 2H, 22, 24), 5.2 (s, 2H, 27), 3.7 (s, 2H, 11), 2.5 – 2.3 (m, 4H, 13, 17), 1.6 – 1.5 (m, 4H, 14, 16), 1.4 – 1.4 (m, 2H, 15); **$^{13}$C NMR (101 MHz, DMSO-*d*$_6$) δ** 164.6 (18), 160.8 (23), 136.7 (28), 129.5 (30, 32), 128.5 (20, 25), 128.0 (21), 127.8 (22, 24), 127.5 (2, 3), 114.4 (29, 31, 33), 69.4 (27), 56.6 (11), 54.1 (13, 17), 25.4 (14, 16), 23.7 (15); **LR-ESI-MS**: $C_{27}H_{29}N_4O_2$ $[M+H]^+$ *m/z* found 441.41, cald 441.23; **HR-ESI-MS**: $C_{27}H_{29}N_4O_2$ $[M+H]^+$ *m/z* found 441.2257, cald 441.2291.

5-chloro-*N*-(2-(piperidin-1-ylmethyl)-1H-benzo[d]imidazol-5-yl)picolinamide **132**

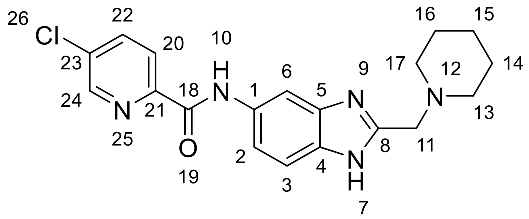

Synthesised according to general procedure **B** to give **132** (0.080 g, 0.217 mmol, 100 %) as an off white solid.

**Mpt:** 147.4-149.4 °C; $\mathbf{v_{max}}$ **(cm$^{-1}$)** 2934, 1670, 1526, 1454, 1109, 1014, 556; **$^{1}$H NMR (400 MHz, DMSO-*d*$_6$) δ** 8.2 (d, *J* = 2.0 Hz, 1H, 6), 2.7 – 2.5 (m, 4H), 10.6 (s, 1H, 7), 8.8 (dd, *J* = 1.0, 2.2 Hz, 1H, 24), 8.2 (dd, *J* = 1.6, 2.6 Hz, 2H, 20, 22), 7.6 (dd, *J* = 2.0, 8.7 Hz, 1H, 2), 7.5 (d, *J* = 8.7 Hz, 1H, 3), 3.9 (s, 2H, 11), 2.6 (t, *J* = 5.1 Hz, 4H, 13, 17), 1.6 (t, *J* = 5.7 Hz, 4H, 14, 16), 1.5 – 1.3 (m, 2H, 15); **$^{13}$C NMR (101 MHz, DMSO-*d*$_6$) δ** 161.4 (18), 150.9 (8), 148.8 (24), 147.0 (21), 137.9 (22), 134.1 (23), 132.8 (5), 128.3 (1), 123.9 (20), 115.8 (2, 3), 55.7 (11), 53.8 (13, 17), 24.8 (14, 16), 23.2 (15); **LR-ESI-MS**: $C_{19}H_{21}ClN_5O$ $[M+H]^+$ *m/z* found 370.48, cald 370.14; **HR-ESI-MS**: $C_{19}H_{21}ClN_5O$ $[M+H]^+$ *m/z* found 370.1408, cald 370.1435.

*N*-(2-(piperidin-1-ylmethyl)-1H-benzo[d]imidazol-5-yl)-4-propoxybenzamide **133**

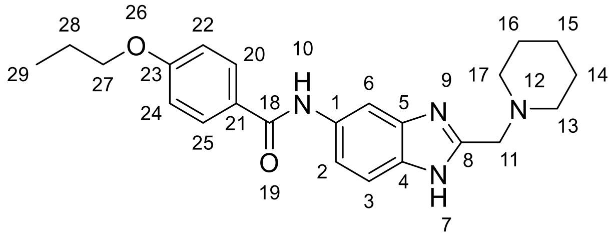

Synthesised according to general procedure **B** to give **133** (0.078 g, 0.199 mmol, 92 %) as a white solid.

**Mpt:** 127.3-129.3 °C; **$v_{max}$ (cm$^{-1}$)** 2934, 1603, 1247, 760, 555; **$^1$H NMR (400 MHz, DMSO-*d*$_6$) δ** 12.2 (s, 1H, 10), 10.1 (s, 1H, 7), 8.1 (s, 1H, 6), 8.0 (d, *J* = 8.8 Hz, 2H, 20, 25), 7.4 (d, *J* = 1.3 Hz, 2H, 2, 3), 7.0 (d, *J* = 8.9 Hz, 2H, 22, 24), 4.0 (t, *J* = 6.5 Hz, 2H, 27), 3.7 (s, 2H, 11), 2.5 – 2.4 (m, 4H, 13, 17), 1.8 – 1.7 (m, 2H, 28), 1.5 (p, *J* = 5.5 Hz, 4H, 14, 16), 1.4 (p, *J* = 3.2, 4.6 Hz, 2H, 15), 1.0 (t, *J* = 7.4 Hz, 3H, 29); **$^{13}$C NMR (101 MHz, DMSO-*d*$_6$) δ** 164.7 (18), 161.2 (23), 151.5 (8), 133.8 (1), 129.5 (20, 25), 127.1 (21), 115.7 (2), 114.0 (22, 24), 69.2 (27), 56.3 (11), 54.0 (13, 17), 25.2 (14, 16), 23.5 (15), 22.0 (28), 10.4 (29); **LR-ESI-MS**: $C_{23}H_{29}N_4O_2$ $[M+H]^+$ *m/z* found 393.58, cald 393.23; **HRS-ESI-MS**: $C_{23}H_{29}N_4O_2$ $[M+H]^+$ *m/z* found 393.2265, cald 393.2291.

*N*-(2-(piperidin-1-ylmethyl)-1H-benzo[d]imidazol-5-yl)thiophene-3-carboxamide **134**

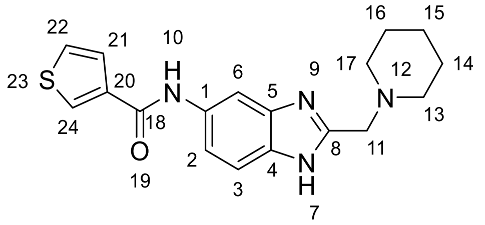

Synthesised according to general procedure **B** to give **134** (0.074 g, 0.217 mmol, 100 %) as a white solid.

**Mpt:** 142.4-144.4 °C; **$v_{max}$ (cm$^{-1}$)** 2933, 1639, 1530, 1417, 1273, 1104, 737, 555; **$^1$H NMR (400 MHz, DMSO-*d*$_6$) δ** 12.3 (s, 1H, 10), 10.1 (s, 1H, 7), 8.4 (dd, *J* = 1.4, 2.8 Hz, 1H, 24), 8.1 (t, *J* = 1.4 Hz, 1H, 6), 7.7 – 7.6 (m, 2H, 21, 22), 7.5 (d, *J* = 1.3 Hz, 2H, 2, 3), 3.7 (s, 2H, 11), 2.5 (tt, *J* = 2.0, 4.8 Hz, 4H, 13, 17), 1.6 (p, *J* = 5.5 Hz, 4H, 14, 16), 1.4 (t, *J* = 4.7 Hz, 2H, 15); **$^{13}$C NMR (101 MHz, DMSO-*d*$_6$) δ** 160.7 (18), 151.5 (8), 138.1 (22, 24), 133.5 (21), 129.4 (20), 127.3 (4), 126.8 (1), 115.7 (2, 3), 56.1 (11), 54.0 (13, 17), 25.1 (14, 16), 23.5 (15); **LR-ESI-MS**: $C_{18}H_{21}N_4OS$ $[M+H]^+$ *m/z* found 341.45, cald 341.14; **HR-ESI-MS**: $C_{18}H_{21}N_4OS$ $[M+H]^+$ *m/z* found 341.1412, cald 341.1436.

4-butyl-*N*-(2-(piperidin-1-ylmethyl)-1H-benzo[d]imidazol-5-yl)benzamide **135**

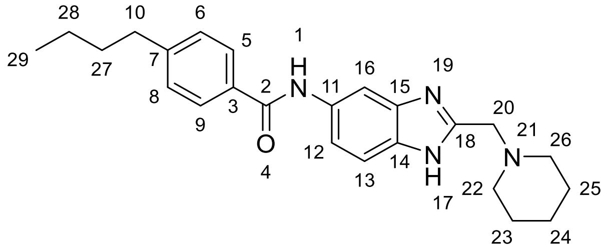

Synthesised according to general procedure **C** to give **135** (0.075 g, 0.192 mmol, 88 %) as a white solid.

**Mpt:** 180.3-182.3 °C; **$v_{max}$ (cm$^{-1}$)** 2929, 1430, 1106, 848, 808, 750, 604; **$^1$H NMR (400 MHz, DMSO-*d*$_6$) δ** 12.2 (s, 1H, 1), 10.1 (s, 1H, 17), 8.1 (s, 1H, 16), 8.0 – 7.9 (m, 2H, 5, 9), 7.4 (s, 2H, 12, 13), 7.4 – 7.3 (m, 2H, 6, 8), 3.7 (s, 2H, 20), 2.7 (t, *J* = 7.7 Hz, 2H), 2.4 (d, *J* = 5.5 Hz, 4H, 22, 26), 1.6 – 1.5 (m, 6H, 23, 25, 27), 1.4 – 1.4 (m, 2H, 24), 1.3 (dt, *J* = 7.4, 14.8 Hz, 2H, 28), 0.9 (t, *J* = 7.3 Hz, 3H, 29); **$^{13}$C NMR (101 MHz, DMSO-*d*$_6$) δ** 165.2 (2), 146.0 (7), 132.7 (14, 15), 128.2 (5, 9), 127.7 (6, 8), 56.6 (20), 54.1 (22, 26), 34.7 (10), 32.9 (27), 25.4 (23, 25), 23.8 (24), 21.7 (28), 13.8 (29); **LR-ESI-MS**: $C_{24}H_{31}N_4O$ [M+H]$^+$ *m/z* found 391.57, cald 391.25; **HR-ESI-MS**: $C_{24}H_{31}N_4O$ [M+H]$^+$ *m/z* found 391.2469, cald 391.2498.

*N*-(2-(piperidin-1-ylmethyl)-1H-benzo[d]imidazol-5-yl)-[1,1'-biphenyl]-4-carboxamide **136**

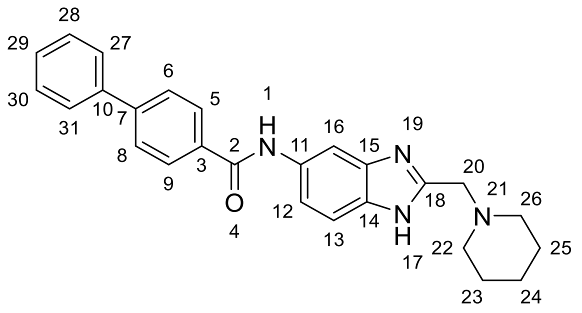


Synthesised according to general procedure **C** to give **136** (0.063 g, 0.153 mmol, 71 %) as a white solid.

**Mpt:** 203.1-205.1 °C; **$v_{max}$ (cm$^{-1}$)** 2931, 1641, 1598, 1530, 1483, 1418, 1299, 1107, 852, 694; **$^1$H NMR (400 MHz, DMSO-*d*$_6$) δ** 12.3 (s, 1H, 1), 10.3 (s, 1H, 17), 8.1 – 8.0 (m, 3H, 5, 9, 16), 7.9 – 7.8 (m, 2H, 6, 8), 7.8 – 7.7 (m, 2H, 12, 13), 7.6 – 7.4 (m, 5H, 27, 28, 29, 30, 31), 3.7 (s, 2H, 20), 2.4 (t, *J* = 5.2 Hz, 4H, 22, 26), 1.5 (p, *J* = 5.5 Hz, 4H, 23, 25), 1.4 (q, *J* = 6.3 Hz, 2H, 24); **$^{13}$C NMR (101 MHz, DMSO-*d*$_6$) δ** 164.9 (2), 142.9 (10), 139.2 (3), 134.0 (29), 129.9 (15), 129.1 (5, 9), 128.3 (28, 30), 128.1 (14), 126.9 (6, 8), 126.6 (27, 31), 56.7 (20), 54.2 (22, 26), 25.5 (23, 25), 23.8 (24); **LR-ESI-MS**: $C_{26}H_{27}N_4O$ [M+H]$^+$ *m/z* found 411.54, cald 411.22; **HR-ESI-MS**: $C_{26}H_{27}N_4O$ [M+H]$^+$ *m/z* found 411.2153, cald 411.2185.

4-butoxy-*N*-(2-(piperidin-1-ylmethyl)-1H-benzo[d]imidazol-5-yl)benzamide **137**

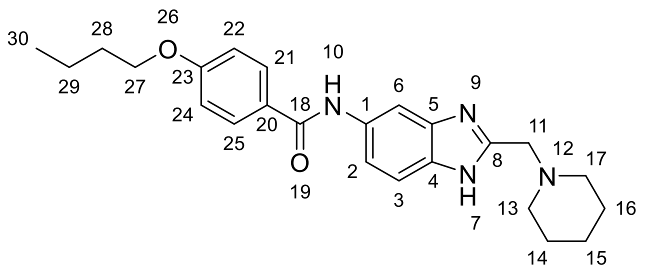


Synthesised according to general procedure **B** to give **137** (0.059 g, 0.145 mmol, 67 %) as a white solid.

**Mpt:** 153.2-155.2 °C; **$v_{max}$ (cm$^{-1}$)** 2931, 1604, 1506, 1430, 1305, 1248, 1176, 1109, 842; **$^1$H NMR (400 MHz, DMSO-*d*$_6$) δ** 12.1 (s, 1H, 10), 10.1 (s, 1H, 7), 8.1 (s, 1H, 6), 8.0 (d, *J* = 8.8

Hz, 2H, 21, 25), 7.5 – 7.4 (m, 2H, 2, 3), 7.0 (d, *J* = 8.9 Hz, 2H, 22, 24), 4.1 (t, *J* = 6.5 Hz, 2H, 27), 3.7 (s, 2H, 11), 2.5 (d, *J* = 5.7 Hz, 4H, 13, 17), 1.7 (dq, *J* = 6.5, 8.5 Hz, 2H, 28), 1.5 (q, *J* = 5.6 Hz, 4H, 14, 16), 1.5 – 1.3 (m, 4H, 15, 29), 0.9 (t, *J* = 7.4 Hz, 3H, 30); **$^{13}$C NMR (101 MHz, DMSO-*d*$_6$) δ** 164.7 (18), 161.2 (23), 129.5 (21, 25), 127.1 (20), 114.0 (22, 24), 67.4 (27), 56.4 (11), 54.1 (13, 17), 30.7 (28), 25.3 (14, 16), 23.6 (15), 18.7 (29), 13.7 (30); **LR-ESI-MS**: $C_{24}H_{31}N_4O_2$ [M+H]$^+$ *m/z* found 407.59, cald 407.25; **HR-ESI-MS**: $C_{24}H_{31}N_4O_2$ [M+H]$^+$ *m/z* found 407.2413, cald 407.2447.

*N*-(2-(((cis)-3,5-dimethylpiperidin-1-yl)methyl)-1H-benzo[d]imidazol-5-yl)-2-methylbenzo[d]oxazole-6-carboxamide **138**

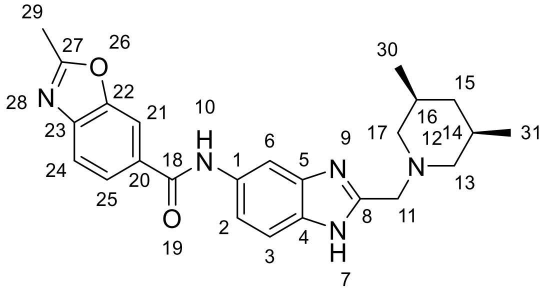


Synthesised according to general procedure **B** to give **138** (0.026 g, 0.062 mmol, 32 %) as an off white solid.

**Mpt:** 143.8-145.8 °C; **$\nu_{max}$ (cm$^{-1}$)** 2949, 2924, 1646, 1602, 1529, 1424, 1339, 1256, 738, 556; **$^1$H NMR (400 MHz, DMSO-*d*$_6$) δ** 12.2 (s, 1H, 10), 10.3 (d, *J* = 20.4 Hz, 1H, 7), 8.3 (d, *J* = 1.6 Hz, 1H, 21), 8.1 – 7.9 (m, 2H, 6, 24), 7.8 (d, *J* = 8.3 Hz, 1H, 25), 7.5 (d, *J* = 8.3 Hz, 1H, 2), 7.5 – 7.4 (m, 1H, 3), 3.7 (s, 2H, 11'), 2.8 (dt, *J* = 2.3, 10.5 Hz, 2H, 13'', 17''), 2.7 (s, 3H, 29), 1.7 (dt, *J* = 3.9, 7.0 Hz, 3H, 13', 15', 17'), 1.6 (t, *J* = 10.7 Hz, 2H, 14, 16), 0.8 (d, *J* = 6.3 Hz, 6H, 30, 31), 0.6 – 0.4 (m, 1H, 15''); **$^{13}$C NMR (101 MHz, DMSO-*d*$_6$) δ** 166.3 (27), 164.6 (18), 152.1 (8), 150.1 (22), 143.7 (23), 134.4 (5), 133.7 (4), 131.7 (20), 124.2 (24), 118.6 (25), 118.1 (2), 115.1 (3), 109.9 (21), 103.4 (6), 61.1 (13, 17), 56.2 (11), 41.6 (15), 30.6 (14, 16), 19.5 (29), 14.4 (30, 31); **LR-ESI-MS**: $C_{24}H_{28}N_5O_2$ [M+H]$^+$ *m/z* found 418.41, cald 418.22; **HR-ESI-MS**: $C_{24}H_{28}N_5O_2$ [M+H]$^+$ *m/z* found 418.2209, cald 418.2243.

4-chloro-*N*-(2-(((3R,5S)-3,5-dimethylpiperidin-1-yl)methyl)-1H-benzo[d]imidazol-5-yl)benzamide **139**

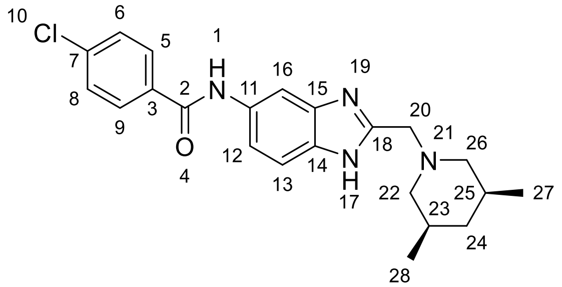


Synthesised according to general procedure **C** to give **139** (0.044 g, 0.111 mmol, 57 %) as an off white solid.

**Mpt:** 142.2-144.2 °C; **$\nu_{max}$ (cm$^{-1}$)** 2949, 2924, 1644, 1595, 1530, 1483, 1275, 1090, 1013, 843, 807, 749; **$^1$H NMR (400 MHz, DMSO-*d*$_6$) δ** 12.3 (s, 1H, 1), 10.3 (s, 1H, 17), 8.0 (s, 1H,

16), 8.0 – 7.9 (m, 2H, 5, 9), 7.7 – 7.6 (m, 2H, 6, 8), 7.5 (d, *J* = 32.2 Hz, 2H, 12, 13), 3.7 (s, 2H, 20'), 2.8 – 2.7 (m, 2H, 22'', 26''), 1.7 – 1.6 (m, 3H, 22', 24', 26'), 1.5 (t, *J* = 10.7 Hz, 2H, 23, 25), 0.8 (d, *J* = 6.3 Hz, 6H, 27, 28), 0.5 (dt, *J* = 12.1, 14.0 Hz, 1H, 24''); **$^{13}$C NMR (101 MHz, DMSO-*d*$_6$) δ** 164.1 (2), 136.2 (7), 133.9 (3), 129.6 (5, 9), 128.4 (6, 8), 61.1 (20), 56.1 (22, 26), 41.6 (24), 30.6 (23, 25), 19.5 (27, 28); **LR-ESI-MS**: $C_{22}H_{26}ClN_4O$ $[M+H]^+$ *m/z* found 397.48, cald 397.18; **HR-ESI-MS**: $C_{22}H_{26}ClN_4O$ $[M+H]^+$ *m/z* found 397.1768, cald 397.1795.

*N*-(2-(piperidin-1-ylmethyl)-1H-benzo[d]imidazol-5-yl)-4-(pyridin-4-yl)benzamide **140**

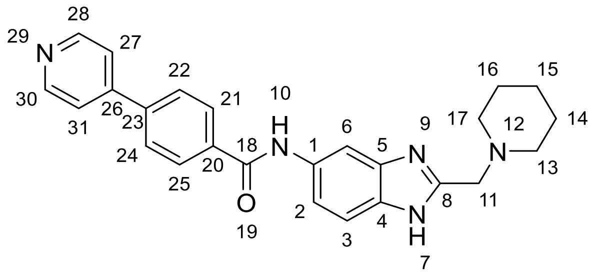


Synthesised according to general procedure **B** to give **140** (0.027 g, 0.066 mmol, 30 %) as a white solid.

**Mpt:** >230 °C; **v$_{max}$ (cm$^{-1}$)** 2931, 1645, 1597, 1534, 1483, 1414, 817, 757; **$^1$H NMR (400 MHz, DMSO-*d*$_6$) δ** 12.3 (s, 1H, 10), 10.4 (s, 1H, 7), 8.7 – 8.6 (m, 2H, 28, 30), 8.2 – 8.0 (m, 3H, 6, 21, 25), 8.0 – 7.9 (m, 2H, 22, 24), 7.9 – 7.7 (m, 2H, 27, 31), 7.6 – 7.3 (m, 2H, 2, 3), 3.7 (s, 2H, 11), 2.4 (t, *J* = 5.3 Hz, 4H, 13, 17), 1.5 (p, *J* = 5.5 Hz, 4H, 14, 16), 1.4 (q, *J* = 6.2 Hz, 2H, 15); **$^{13}$C NMR (101 MHz, DMSO-*d*$_6$) δ** 164.6 (18), 150.4 (28, 30), 146.0 (26), 139.8 (23), 135.7 (20), 128.5 (21, 25), 126.8 (22, 24), 121.4 (27, 31), 56.6 (11), 54.2 (13, 17), 25.5 (14, 16), 23.8 (15); **LR-ESI-MS**: $C_{25}H_{26}N_5O$ $[M+H]^+$ *m/z* found 412.40, cald 412.21; **HR-ESI-MS**: $C_{25}H_{26}N_5O$ $[M+H]^+$ *m/z* found 412.2106, cald 412.2137.

*N*-(2-(1-(piperidin-1-yl)ethyl)-1H-benzo[d]imidazol-5-yl)-4-(trifluoromethyl)benzamide **141**

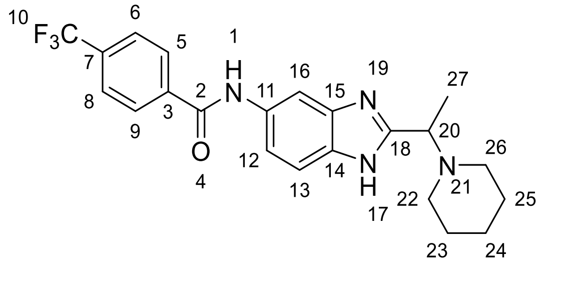


Synthesised according to general procedure **C** to give **141** (0.043 g, 0.103 mmol, 50 %) as a white solid.

**Mpt:** 148.2-150.2 °C; **v$_{max}$ (cm$^{-1}$)** 2933, 1648, 1547, 1450, 1322, 1165, 1064, 855, 688; **$^{19}$F NMR (376 MHz, DMSO-*d*$_6$) δ** -61.3; **$^1$H NMR (400 MHz, DMSO-*d*$_6$) δ** 12.1 (s, 1H, 1), 10.5 (s, 1H, 16), 8.2 (d, *J* = 8.1 Hz, 2H, 6, 8), 8.1 (s, 1H, 15), 7.9 (d, *J* = 8.1 Hz, 2H, 5, 9), 7.5 (d, *J* = 17.6 Hz, 2H, 11, 12), 3.9 (d, *J* = 7.0 Hz, 1H, 19), 2.4 (s, 4H, 21, 25), 1.5 (q, *J* = 5.7 Hz, 4H, 22, 24), 1.4 (d, *J* = 6.9 Hz, 3H, 26), 1.4 (td, *J* = 4.7, 8.0, 9.0 Hz, 2H, 23); **$^{13}$C NMR (101 MHz, DMSO-*d*$_6$) δ** 164.1 (2), 139.1 (3), 131.2 (q, *J* = 32.1 Hz, 27), 128.6 (5, 9), 125.4 (q, *J* = 3.7

Hz, 6, 8), 122.6 (7), 58.5 (19), 50.3 (21, 25), 25.7 (22, 24), 24.1 (23), 14.5 (26); **LR-ESI-MS**: $C_{22}H_{24}F_3N_4O$ [M+H]$^+$ *m/z* found 417.53, cald 417.19; **HR-ESI-MS**: $C_{22}H_{24}F_3N_4O$ [M+H]$^+$ *m/z* found 417.1875, cald 417.1902.

*N*-(2-(piperidin-1-ylmethyl)-1H-benzo[d]imidazol-5-yl)-2-naphthamide **142**

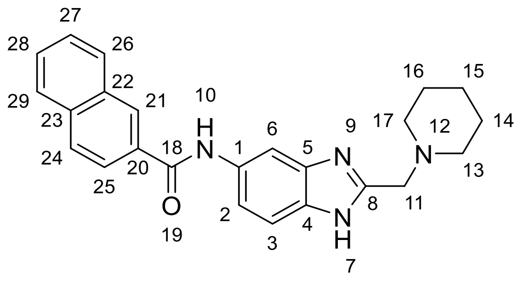


Synthesised according to general procedure **B** to give **142** (0.063 g, 0.164 mmol, 76 %) as an off white solid.

**Mpt:** 135.7-137.7 °C; **$v_{max}$ (cm$^{-1}$)** 2932, 1644, 1485, 1288, 773, 556, 474; **$^1$H NMR (400 MHz, DMSO-*d*$_6$) δ** 12.3 (s, 1H, 10), 10.4 (s, 1H, 7), 8.6 (d, *J* = 1.5 Hz, 1H, 6), 8.2 – 7.9 (m, 5H, 21, 24, 25, 26, 29), 7.7 – 7.6 (m, 2H, 27, 28), 7.6 – 7.5 (m, 2H, 2, 3), 3.7 (s, 2H, 11), 2.5 (d, *J* = 5.2 Hz, 4H, 13, 17), 1.5 (p, *J* = 5.5 Hz, 4H, 14, 16), 1.4 (q, *J* = 4.6, 5.9 Hz, 2H, 15); **$^{13}$C NMR (101 MHz, DMSO-*d*$_6$) δ** 165.8 (18), 134.7 (20), 133.0 (21), 132.6 (25), 129.4 (23), 128.4 (26), 128.3 (28), 128.2 (22), 128.1 (24), 127.3 (29), 125.0 (27), 56.9 (11), 54.5 (13, 17), 25.8 (14, 16), 24.1 (15); **LR-ESI-MS**: $C_{24}H_{25}N_4O$ [M+H]$^+$ *m/z* found 385.52, cald 385.20; **HR-ESI-MS**: $C_{24}H_{25}N_4O$ [M+H]$^+$ *m/z* found 385.1994, cald 385.2028.

3-(1-methyl-1H-indol-5-yl)-*N*-(2-(piperidin-1-ylmethyl)-1H-benzo[d]imidazol-5-yl)propanamide **143**

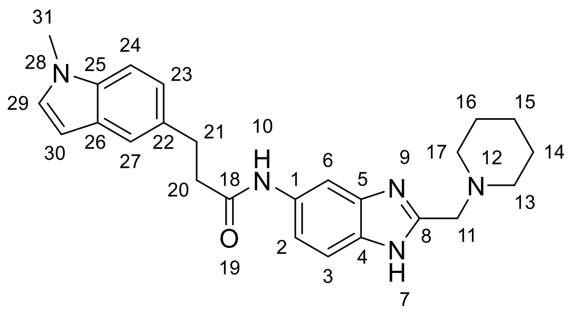


Synthesised according to general procedure **B** to give **143** (0.066 g, 0.159 mmol, 73 %) as a white solid.

**Mpt:** 136.3-138.3 °C; **$v_{max}$ (cm$^{-1}$)** 2932, 1656, 1529, 1488, 1447, 1335, 718, 556; **$^1$H NMR (400 MHz, DMSO-*d*$_6$) δ** 12.2 (s, 1H, 10), 9.9 (s, 1H, 7), 8.0 – 7.9 (m, 1H, 6), 7.4 – 7.4 (m, 2H, 24, 27), 7.3 (d, *J* = 8.4 Hz, 1H, 2), 7.3 (d, *J* = 3.0 Hz, 1H, 29), 7.2 – 7.1 (m, 1H, 3), 7.1 (dd, *J* = 1.6, 8.4 Hz, 1H, 23), 6.3 (dd, *J* = 0.9, 3.1 Hz, 1H, 30), 3.7 (s, 3H, 31), 3.7 (s, 2H, 11), 3.0 (dd, *J* = 6.7, 8.7 Hz, 2H, 21), 2.6 (dd, *J* = 6.9, 8.6 Hz, 2H, 20), 2.4 (t, *J* = 5.3 Hz, 4H, 13, 17), 1.5 (t, *J* = 5.7 Hz, 4H, 14, 16), 1.5 – 1.3 (m, 2H, 15); **$^{13}$C NMR (101 MHz, DMSO-*d*$_6$) δ** 170.3 (18), 135.1 (29), 131.6 (25), 129.6 (22), 128.2 (23), 121.9 (27), 119.4 (26), 109.5 (24),

99.9 (30), 56.5 (11), 54.1 (13, 17), 32.5 (31), 31.3 (21), 25.3 (14, 16), 23.7 (15), 18.8 (20); **LR-ESI-MS**: $C_{25}H_{30}N_5O$ $[M+H]^+$ *m/z* found 416.54, cald 416.25; **HR-ESI-MS**: $C_{25}H_{30}N_5O$ $[M+H]^+$ *m/z* found 416.2419, cald 416.2450.

*N*-(2-(piperidin-1-ylmethyl)-1H-benzo[d]imidazol-5-yl)-2,3-dihydrobenzofuran-5-carboxamide **144**

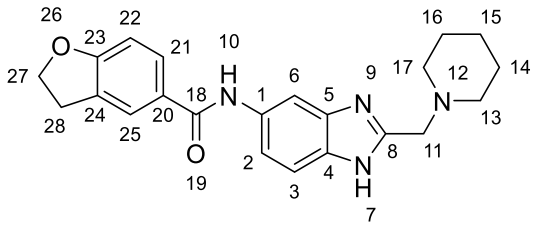


Synthesised according to general procedure **B** to give **144** (0.067 g, 0.178 mmol, 82 %) as a white solid.

**Mpt:** 143.8-145.8 °C; **$\nu_{max}$ (cm$^{-1}$)** 2932, 1640, 1608, 1528, 1484, 1241, 1106, 980, 839, 756, 556; **$^1$H NMR (400 MHz, DMSO-*d*$_6$) δ** 12.2 (s, 1H, 10), 10.0 (s, 1H, 7), 8.0 (s, 1H, 6), 7.9 (d, *J* = 1.8 Hz, 1H, 25), 7.8 (dd, *J* = 2.0, 8.3 Hz, 1H, 21), 7.4 (s, 2H, 2, 3), 6.9 (d, *J* = 8.4 Hz, 1H, 22), 4.6 (t, *J* = 8.7 Hz, 2H, 27), 3.7 (s, 2H, 11), 3.3 (t, *J* = 8.7 Hz, 2H, 28), 2.4 (d, *J* = 5.4 Hz, 4H, 13, 17), 1.5 (q, *J* = 5.6 Hz, 4H, 14, 16), 1.4 (q, *J* = 6.0 Hz, 2H, 15); **$^{13}$C NMR (101 MHz, DMSO-*d*$_6$) δ** 164.8 (18), 162.4 (23), 128.5 (25), 127.6 (21), 127.4 (20), 124.9 (24), 108.4 (22), 71.8 (27), 56.5 (11), 54.1 (13, 17), 28.6 (28), 25.4 (14, 16), 23.7 (15); **LR-ESI-MS**: $C_{22}H_{25}N_4O_2$ $[M+H]^+$ *m/z* found 377.54, cald 377.19; **HR-ESI-MS**: $C_{22}H_{25}N_4O_2$ $[M+H]^+$ *m/z* found 377.1955, cald 377.1978.

*N*-(2-(1-(piperidin-1-yl)ethyl)-1H-benzo[d]imidazol-5-yl)-4-(pyridin-4-yl)benzamide **145**

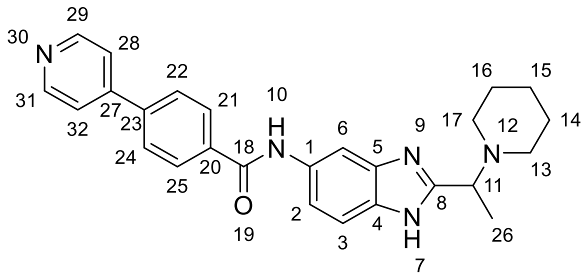


Synthesised according to general procedure **B** to give **145** (0.062 g, 0.146 mmol, 71 %) as a white solid.

**Mpt:** 192.2-194.2 °C; **$\nu_{max}$ (cm$^{-1}$)** 2933, 1650, 1599, 1544, 1485, 1302, 555; **$^1$H NMR (400 MHz, DMSO-*d*$_6$) δ** 12.1 (s, 1H, 10), 10.3 (s, 1H, 7), 8.8 – 8.6 (m, 2H, 29, 31), 8.1 (t, *J* = 6.7 Hz, 3H, 6, 21, 25), 8.0 (d, *J* = 8.5 Hz, 2H, 22, 24), 7.8 – 7.7 (m, 2H, 28, 32), 7.5 (s, 2H, 2, 3), 3.9 (d, *J* = 7.2 Hz, 1H, 11), 2.5 – 2.4 (m, 4H, 13, 17), 1.5 (t, *J* = 5.8 Hz, 4H, 14, 16), 1.4 (d, *J* = 6.9 Hz, 3H, 26), 1.4 (dd, *J* = 3.8, 8.5 Hz, 2H, 15); **$^{13}$C NMR (101 MHz, DMSO-*d*$_6$) δ** 164.7 (18), 150.4 (29, 31), 146.0 (27), 139.8 (23), 135.7 (20), 128.5 (21, 25), 126.8 (22, 24), 121.4 (28, 32), 58.6 (11), 50.3 (13, 17), 25.6 (14, 16), 24.0 (15), 14.5 (26). **LR-ESI-MS**: $C_{26}H_{28}N_5O$ $[M+H]^+$*m/z* found 426.61, cald 426.23; **HR-ESI-MS**: $C_{26}H_{28}N_5O$ $[M+H]^+$ *m/z* found 426.2260, cald 426.2294.

4-cyano-*N*-(2-(((3R,5S)-3,5-dimethylpiperidin-1-yl)methyl)-1H-benzo[d]imidazol-5-yl)benzamide **146**

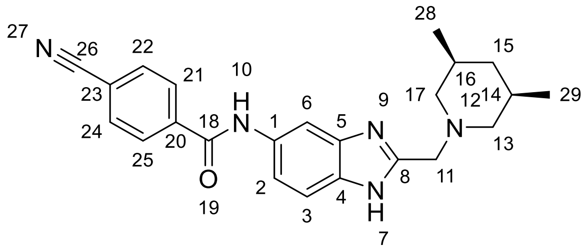

Synthesised according to general procedure **B** to give **146** (0.052 g, 0.134 mmol, 69 %) as an off white solid.

**Mpt:** 148.3-150.3 °C; **$\nu_{max}$ ($cm^{-1}$)** 2949, 2923, 2229, 1647, 1600, 1529, 1485, 1452, 1416, 1278, 806, 756, 568; **$^{1}$H NMR (400 MHz, DMSO-$d_6$) δ** 12.3 (s, 1H, 10), 10.5 (s, 1H, 7), 8.2 – 8.1 (m, 2H, 21, 25), 8.1 (s, 1H, 6), 8.0 – 7.9 (m, 2H, 22, 24), 7.5 (d, *J* = 20.5 Hz, 2H, 2, 3), 3.7 (s, 2H, 11'), 2.9 – 2.7 (m, 2H, 13'', 17''), 1.7 (tt, *J* = 4.4, 5.8, 8.5 Hz, 3H, 13', 15', 17'), 1.5 (t, *J* = 10.7 Hz, 2H, 14, 16), 0.8 (d, *J* = 6.2 Hz, 6H, 28, 29), 0.5 (dt, *J* = 12.1, 13.9 Hz, 1H, 15''); **$^{13}$C NMR (101 MHz, DMSO-$d_6$) δ** 163.8 (18), 139.3 (20), 132.5 (21, 25), 128.5 (22, 24), 118.4 (23), 113.7 (26), 61.1 (13, 17), 56.1 (11), 41.6 (15), 30.6 (14, 16), 19.5 (28, 29); **LR-ESI-MS**: $C_{23}H_{26}N_5O$ $[M+H]^+$ *m/z* found 388.57, cald 388.21; **HR-ESI-MS**: $C_{23}H_{26}N_5O$ $[M+H]^+$ *m/z* found 388.2113, cald 388.2137.

4-(benzyloxy)-*N*-(2-(((cis)-3,5-dimethylpiperidin-1-yl)methyl)-1H-benzo[d]imidazol-5-yl)benzamide **147**

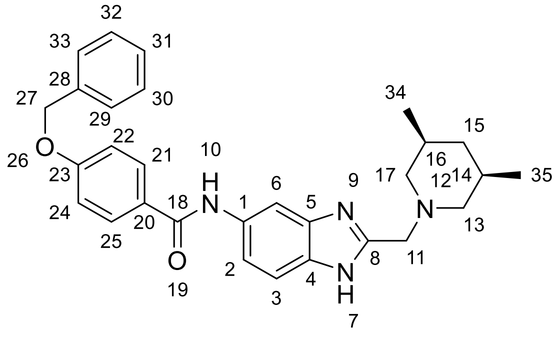

Synthesised according to general procedure **B** to give **147** (0.035 g, 0.075 mmol, 39 %) as an off white solid.

**Mpt:** >225 °C; **$\nu_{max}$ ($cm^{-1}$)** 2922, 1641, 1603, 1452, 1174, 1000, 734, 695, 613; **$^{1}$H NMR (400 MHz, DMSO-$d_6$) δ** 12.2 (s, 1H, 10), 10.1 (s, 1H, 7), 8.1 (s, 1H, 6), 8.0 (d, *J* = 8.8 Hz, 2H, 21, 25), 7.5 – 7.3 (m, 7H, 2, 3, 29, 30, 31, 32, 33), 7.2 – 7.0 (m, 2H, 22, 24), 5.2 (s, 2H, 27''), 3.7 (s, 2H, 11'), 2.9 – 2.7 (m, 2H, 13'', 17''), 1.8 – 1.6 (m, 3H, 13', 15'', 17'), 1.5 (t, *J* = 10.7 Hz, 2H, 14, 16), 0.8 (d, *J* = 6.3 Hz, 6H, 34, 35), 0.6 – 0.4 (m, 1H, 15'); **$^{13}$C NMR (101 MHz, DMSO-$d_6$) δ** 164.6 (18), 160.8 (23), 136.7 (28), 129.5 (21, 25), 128.5 (30, 32), 128.0 (31), 127.8 (29, 33), 127.5 (20), 114.4 (22, 24), 69.4 (27), 61.1 (13, 17), 56.1 (11), 41.6 (15), 30.6 (14, 16), 19.5 (34, 35); **LR-ESI-MS**: $C_{29}H_{33}N_4O_2$ $[M+H]^+$ *m/z* found 469.41, cald 469.26; **HR-ESI-MS**: $C_{29}H_{33}N_4O_2$ $[M+H]^+$ *m/z* found 469.2570, cald 469.2604.

3-(*N*-(4-chlorobenzyl)sulfamoyl)-4-methoxy-*N*-(2-(piperidin-1-ylmethyl)-1H-benzo[d]imidazol-5-yl)benzamide **148**

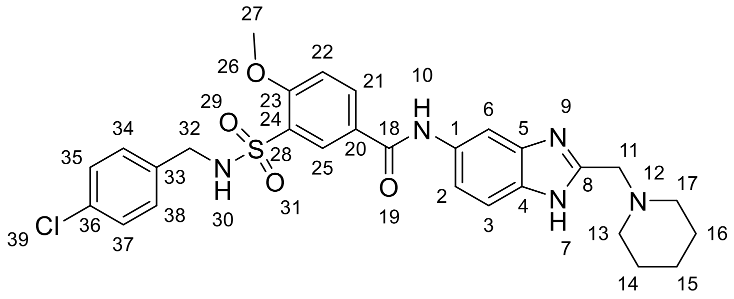

Synthesised according to general procedure **B** to give **148** (0.066 g, 0.116 mmol, 54 %) as a clear oil.

**v$_{max}$ (cm$^{-1}$)** 2933, 1647, 1600, 1488, 1322, 1267, 1150, 1066, 842, 556; **$^{1}$H NMR (400 MHz, DMSO-*d*$_6$) δ** 12.2 (s, 1H), 10.3 (s, 1H), 8.3 (d, *J* = 2.3 Hz, 1H, 25), 8.2 (dd, *J* = 2.3, 8.7 Hz, 1H, 21), 8.1 – 8.0 (m, 2H, 6), 7.5 – 7.4 (m, 2H, 35, 37), 7.3 – 7.1 (m, 5H, 2, 3, 22, 34, 38), 4.1 (d, *J* = 6.3 Hz, 2H, 32), 3.9 (s, 3H, 27), 3.7 (s, 2H, 11), 2.5 – 2.4 (m, 4H, 13, 17), 1.5 (p, *J* = 5.5 Hz, 4H, 14, 16), 1.4 – 1.4 (m, 2H, 15); **$^{13}$C NMR (101 MHz, DMSO-*d*$_6$) δ** 163.6 (18), 158.3 (23), 136.9 (36), 133.7 (25), 131.6 (21), 129.4 (35, 37), 129.2 (20), 128.1 (33), 127.9 (34, 38), 126.6 (24), 112.3 (22), 56.5 (11), 56.3 (27), 54.1 (13, 17), 45.4 (32), 25.6 (14, 16), 23.8 (15); **LR-ESI-MS**: $C_{28}H_{31}ClN_5O_4S$ [M+H]$^+$ *m/z* found 568.58, cald 568.18; **HR-ESI-MS**: $C_{28}H_{31}ClN_5O_4S$ [M+H]$^+$ *m/z* found 569.3997, cald 568.1785.

*N*-(2-((2-methylpyrrolidin-1-yl)methyl)-1H-benzo[d]imidazol-5-yl)-4-(pyridin-4-yl)benzamide **149**

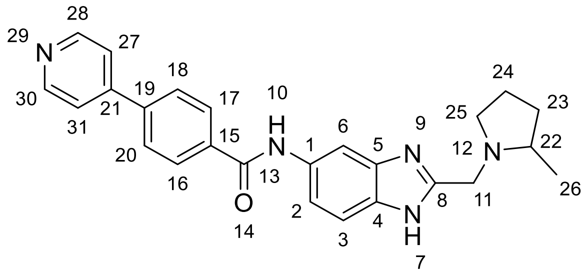

Synthesised according to general procedure **B** to give **149** (0.017 g, 0.042 mmol, 19 %) as a white solid.

**Mpt:** 222.0-224.0 °C; **v$_{max}$ (cm$^{-1}$)** 2962, 2787, 1641, 1549, 1408, 1307, 809, 758, 557; **$^{1}$H NMR (400 MHz, DMSO-*d*$_6$) δ** 12.2 (s, 1H, 10), 10.3 (s, 1H, 7), 8.7 – 8.6 (m, 2H, 28, 30), 8.2 – 8.1 (m, 2H, 16, 17), 8.1 (s, 1H, 6), 8.0 – 7.9 (m, 2H, 18, 20), 7.9 – 7.8 (m, 2H, 27, 31), 7.5 (s, 2H, 2, 3), 4.1 (d, *J* = 14.2 Hz, 1H, 11''), 3.6 (d, *J* = 14.2 Hz, 1H, 11'), 3.0 (d, *J* = 9.5 Hz, 1H, 22), 2.7 – 2.5 (m, 1H, 25'), 2.3 (d, *J* = 9.0 Hz, 1H, 25''), 2.0 – 1.9 (m, 1H, 23''), 1.8 – 1.6 (m, 2H, 23', 24''), 1.5 – 1.3 (m, 1H, 24'), 1.2 – 1.0 (m, 3H, 26); **$^{13}$C NMR (101 MHz, DMSO-*d*$_6$) δ** 164.7 (13), 150.4 (28, 30), 146.0 (21), 139.8 (19), 135.7 (15), 128.5 (16, 17), 126.8 (18, 20), 121.4 (27, 31), 59.3 (11), 54.0 (22), 50.8 (25), 32.4 (23), 21.4 (24), 18.7 (26); **LR-ESI-MS**: $C_{25}H_{26}N_5O$ [M+H]$^+$ *m/z* found 412.56, cald 412.21; **HR-ESI-MS**: $C_{25}H_{26}N_5O$ [M+H]$^+$ *m/z* found 412.2102, cald 412.2137.

4-((6-methylpyrazin-2-yl)oxy)-*N*-(2-((2-methylpyrrolidin-1-yl)methyl)-1H-benzo[d]imidazol-5-yl)benzamide **150**

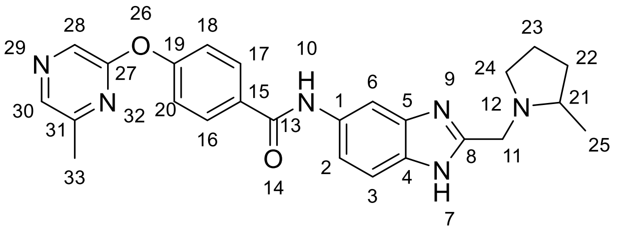

Synthesised according to general procedure **B** to give **150** (0.053 g, 0.120 mmol, 55 %) as an off white solid .

**Mpt:** 101.3-103.3 °C; **$\nu_{max}$ (cm$^{-1}$)** 2962, 1646, 1532, 1400, 1290, 1249, 1167, 837, 555; **$^1$H NMR (400 MHz, DMSO-*d*$_6$) δ** 10.3 (s, 1H, 7), 8.4 – 8.3 (m, 2H, 28, 30), 8.1 – 8.1 (m, 1H, 6), 8.1 – 8.0 (m, 2H, 16, 17), 7.5 (d, *J* = 1.9 Hz, 2H, 2, 3), 7.4 – 7.3 (m, 2H, 18, 20), 4.2 (d, *J* = 14.3 Hz, 1H, 11''), 3.7 (d, *J* = 14.2 Hz, 1H, 11'), 3.1 (q, *J* = 4.0, 7.0 Hz, 1H, 21), 2.8 – 2.7 (m, 1H, 24''), 2.4 (s, 3H, 33), 2.1 – 1.9 (m, 1H, 24'), 1.8 – 1.6 (m, 2H, 22'', 23''), 1.5 – 1.4 (m, 1H, 22'), 1.3 – 1.1 (m, 4H, 23', 25); **$^{13}$C NMR (101 MHz, DMSO-*d*$_6$) δ** 164.6 (13), 158.3 (27), 155.7 (31), 151.1 (8), 138.9 (28, 30), 132.4 (4), 131.8 (5), 131.3 (15), 129.6 (16, 17), 120.6 (18, 20), 60.0 (21), 54.0 (11), 50.4 (24), 32.1 (22), 21.3 (23), 20.6 (33), 18.1 (25); **LR-ESI-MS**: $C_{25}H_{27}N_6O_2$ [M+H]$^+$ *m/z* found 443.52, cald 443.22; **HR-ESI-MS**: $C_{25}H_{27}N_6O_2$ [M+H]$^+$ *m/z* found 443.2161, cald 443.2195.

*N*-(2-(piperidin-1-ylmethyl)-1H-benzo[d]imidazol-5-yl)-3-(trifluoromethyl)benzamide **151**

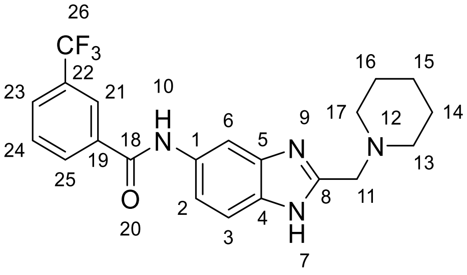

Synthesised according to general procedure **C** to give **151** (0.034 g, 0.084 mmol, 39 %) as a white solid.

**Mpt:** 92.7-94.7 °C; **$\nu_{max}$ (cm$^{-1}$)** 2936, 1648, 1329, 1258, 808, 650; **$^{19}$F NMR (376 MHz, DMSO-*d*$_6$) δ** -61.0; **$^1$H NMR (400 MHz, DMSO-*d*$_6$) δ** 12.3 (s, 1H, 10), 10.5 (s, 1H, 7), 8.4 – 8.1 (m, 2H, 21, 25), 8.1 – 7.9 (m, 2H, 3, 23), 7.8 (t, *J* = 7.8 Hz, 1H, 24), 7.5 (d, *J* = 22.9 Hz, 2H, 2, 6), 3.7 (s, 2H, 11), 2.4 (t, *J* = 5.3 Hz, 4H, 13, 17), 1.5 (p, *J* = 5.5 Hz, 4H, 14, 16), 1.5 – 1.3 (m, 2H, 15); **$^{13}$C NMR (101 MHz, DMSO-*d*$_6$) δ** 163.7 (18), 136.1 (22), 131.8 (25), 129.7 (23, 24), 124.2 (3), 56.6 (11), 54.2 (13, 17), 25.4 (14, 16), 23.8 (15); **LR-ESI-MS**: $C_{21}H_{22}F_3N_4O$ [M+H]$^+$ *m/z* found 403.30, cald 403.18; **HR-ESI-MS**: $C_{21}H_{22}F_3N_4O$ [M+H]$^+$ *m/z* found 403.1733, cald 403.1746.

*N*$_1$-isopropyl-*N*$_4$-(2-(piperidin-1-ylmethyl)-1H-benzo[d]imidazol-5-yl)terephthalamide **152**

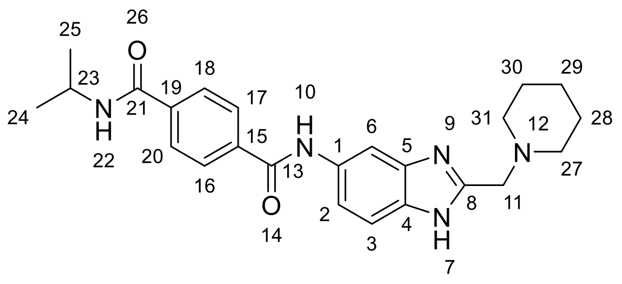

Synthesised according to general procedure **B** to give **152** (0.055 g, 0.131 mmol, 60 %) as an off white solid.

**Mpt:** 186.6-188.6 °C; **$v_{max}$ ($cm^{-1}$)** 2933, 1633, 1531, 1279, 1172, 840, 555; **$^{1}$H NMR (400 MHz, DMSO-*$d_6$*) δ** 12.2 (s, 1H, 10), 10.3 (s, 1H, 7), 8.4 (d, *J* = 7.8 Hz, 1H, 22), 8.1 – 8.0 (m, 3H, 6, 16, 17), 8.0 – 7.9 (m, 2H, 18, 20), 7.5 (s, 2H, 2, 3), 4.1 (dt, *J* = 6.5, 7.8 Hz, 1H, 23), 3.7 (s, 2H, 11), 2.4 (d, *J* = 6.0 Hz, 4H, 27, 31), 1.5 (p, *J* = 5.4 Hz, 4H, 28, 30), 1.5 – 1.3 (m, 2H, 29), 1.2 (d, *J* = 6.6 Hz, 6H, 24, 25); **$^{13}$C NMR (101 MHz, DMSO-*$d_6$*) δ** 164.7 (21), 164.6 (13), 137.2 (15, 19), 127.5 (16, 17), 127.3 (18, 20), 56.5 (11), 54.1 (27, 31), 41.2 (23), 25.3 (28, 30), 23.7 (29), 22.3 (24, 25); **LR-ESI-MS**: $C_{24}H_{30}N_5O_2$ $[M+H]^+$ *m/z* found 420.57, cald 420.24; **HR-ESI-MS**: $C_{24}H_{30}N_5O_2$ $[M+H]^+$ *m/z* found 420.2367, cald 420.2400.

*N*-(2-(piperidin-1-ylmethyl)-1H-benzo[d]imidazol-5-yl)-1H-indole-6-carboxamide **153**

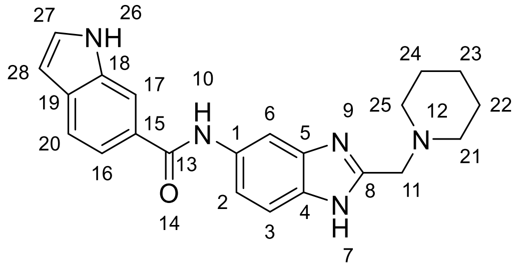

Synthesised according to general procedure **B** to give **153** (0.065 g, 0.174 mmol, 80 %) as an off white solid.

**Mpt:** >215.0 °C; **$v_{max}$ ($cm^{-1}$)** 3399, 3150, 2934, 1623, 1526, 1451, 1107, 831, 555; **$^{1}$H NMR (400 MHz, DMSO-*$d_6$*) δ** 11.5 (t, *J* = 2.3 Hz, 1H, 26), 10.2 (s, 1H, 7), 8.1 (dd, *J* = 1.6, 8.5 Hz, 2H, 6, 27), 7.7 – 7.6 (m, 2H, 16, 17), 7.6 – 7.4 (m, 3H, 2, 3, 20), 6.5 (ddd, *J* = 0.9, 1.9, 3.0 Hz, 1H, 28), 3.7 (s, 2H, 11), 2.5 (dt, *J* = 3.7, 9.2 Hz, 4H, 21, 25), 1.5 (t, *J* = 5.7 Hz, 4H, 22, 24), 1.5 – 1.3 (m, 2H, 23); **$^{13}$C NMR (101 MHz, DMSO-*$d_6$*) δ** 167.7 (13), 153.0 (8), 136.7 (1), 135.6 (5), 131.5 (27), 129.7 (4), 129.3 (15), 120.9 (16), 119.9 (17), 117.1 (2, 3, 6), 113.1 (20), 102.8 (28), 57.7 (11), 55.5 (21, 25), 26.6 (22, 24), 25.0 (23); **LR-ESI-MS**: $C_{22}H_{24}N_5O$ $[M+H]^+$ *m/z* found 374.35, cald 374.19; **HR-ESI-MS**: $C_{22}H_{24}N_5O$ $[M+H]^+$ *m/z* found 374.1958, cald 374.1981.

4-cyclopropyl-*N*-(2-(piperidin-1-ylmethyl)-1H-benzo[d]imidazol-5-yl)benzamide **154**

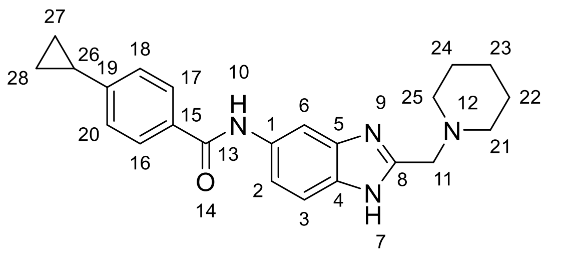

Synthesised according to general procedure **B** to give **154** (0.056 g, 0.150 mmol, 69 %) as an off white solid.

**Mpt:** 147.8-149.8 °C; **$v_{max}$ ($cm^{-1}$)** 2934, 1609, 1451, 837, 555; **$^1$H NMR (400 MHz, DMSO-*d*$_6$) δ** 12.3 (s, 1H, 10), 10.1 (s, 1H, 7), 8.1 (t, *J* = 1.4 Hz, 1H, 6), 7.9 (d, *J* = 8.4 Hz, 2H, 16, 17), 7.5 (d, *J* = 1.3 Hz, 2H, 2, 3), 7.2 (d, *J* = 8.4 Hz, 2H, 18, 20), 3.8 (s, 2H, 11), 2.5 (p, *J* = 1.9 Hz, 4H, 21, 25), 2.0 (tt, *J* = 5.0, 8.3 Hz, 1H, 26), 1.5 (q, *J* = 5.6 Hz, 4H, 22, 24), 1.4 (d, *J* = 6.1 Hz, 2H, 23), 1.1 – 1.0 (m, 2H, 27', 28'), 0.8 – 0.7 (m, 2H, 27'', 28''); **$^{13}$C NMR (101 MHz, DMSO-*d*$_6$) δ** 165.1 (13), 151.4 (8), 147.8 (19), 133.7 (6), 132.1 (4, 5), 127.7 (16, 17), 125.1 (18, 20), 115.7 (2, 3), 56.1 (11), 54.0 (21, 25), 25.1 (22, 24), 23.5 (23), 15.2 (26), 10.2 (27, 28); **LR-ESI-MS**: $C_{23}H_{27}N_4O$ $[M+H]^+$ *m/z* found 375.38, cald 375.22; **HR-ESI-MS**: $C_{23}H_{27}N_4O$ $[M+H]^+$ *m/z* found 375.2158, cald 375.2185.

4-((1H-pyrrol-1-yl)methyl)-*N*-(2-(piperidin-1-ylmethyl)-1H-benzo[d]imidazol-5-yl)benzamide **155**

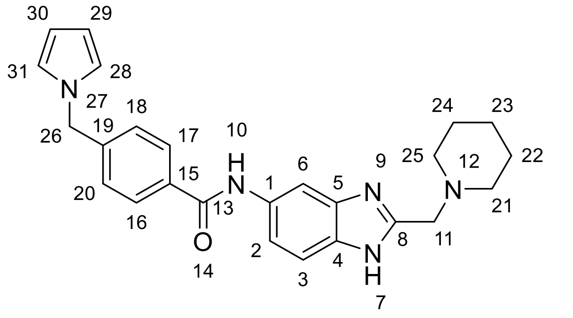

Synthesised according to general procedure **B** to give **155** (0.027 g, 0.064 mmol, 30 %) as a clear oil.

**$v_{max}$ ($cm^{-1}$)** 2933, 1644, 1529, 1417, 1276, 842, 719, 556; **$^1$H NMR (400 MHz, DMSO-*d*$_6$) δ** 12.2 (s, 1H, 10), 10.2 (s, 1H, 7), 8.0 (s, 1H, 6), 8.0 – 7.9 (m, 2H, 18, 20), 7.4 (s, 2H, 2, 3), 7.3 – 7.2 (m, 2H, 16, 17), 6.8 (t, *J* = 2.1 Hz, 2H, 28, 31), 6.0 (t, *J* = 2.1 Hz, 2H, 29, 30), 5.2 (s, 2H, 26), 3.7 (s, 2H, 11), 2.5 – 2.3 (m, 4H, 21, 25), 1.5 (p, *J* = 5.5 Hz, 4H, 22, 24), 1.4 (t, *J* = 6.0 Hz, 2H, 23); **$^{13}$C NMR (101 MHz, DMSO-*d*$_6$) δ** 165.0 (13), 142.5 (19), 134.3 (15), 127.9 (16, 17), 127.0 (18, 20), 121.1 (28, 31), 108.2 (29, 30), 56.5 (11), 54.1 (21, 25), 51.8 (26), 25.4 (22, 24), 23.7 (23); **LR-ESI-MS**: $C_{25}H_{28}N_5O$ $[M+H]^+$ *m/z* found 414.56, cald 414.23; **HR-ESI-MS**: $C_{25}H_{28}N_5O$ $[M+H]^+$ *m/z* found 414.2263, cald 414.2294.

4-((dimethylamino)methyl)-*N*-(2-(piperidin-1-ylmethyl)-1H-benzo[d]imidazol-5-yl)benzamide **156**

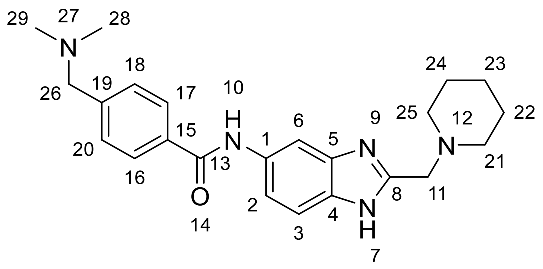


Synthesised according to general procedure **B** to give **156** (0.019 g, 0.049 mmol, 22 %) as a white solid.

**Mpt:** 175.0-177.0 °C; **$\nu_{max}$ (cm$^{-1}$)** 2933, 2777, 1643, 1418, 806, 749; **$^1$H NMR (400 MHz, DMSO-$d_6$) δ** 12.2 (s, 1H, 10), 10.3 – 10.1 (m, 1H, 7), 8.2 – 8.0 (m, 1H, 6), 8.0 – 7.9 (m, 2H, 16, 17), 7.6 – 7.3 (m, 4H, 2, 3, 18, 20), 3.6 (s, 2H, 11), 3.5 (s, 2H, 26), 2.4 (t, *J* = 5.3 Hz, 4H, 21, 25), 2.2 (s, 6H, 28, 29), 1.5 (p, *J* = 5.4 Hz, 4H, 22, 24), 1.4 – 1.3 (m, 2H, 23); **$^{13}$C NMR (101 MHz, DMSO-$d_6$) δ** 165.1 (13), 152.1 (19), 142.6 (5), 134.4 (1), 133.9 (4), 128.5 (16, 17), 127.6 (18, 20), 118.0 (2), 115.0 (3), 103.2 (6), 63.0 (26), 56.7 (11), 54.2 (21, 25), 45.0 (28, 29), 25.5 (22, 24), 23.8 (23); **LR-ESI-MS**: $C_{23}H_{30}N_5O$ [M+H]$^+$ *m/z* found 392.57, cald 392.25; **HR-ESI-MS**: $C_{23}H_{30}N_5O$ [M+H]$^+$ *m/z* found 392.2415, cald 392.2450.

*N*-(2-(piperidin-1-ylmethyl)-1H-benzo[d]imidazol-5-yl)-4-(pyridin-3-yloxy)benzamide **157**

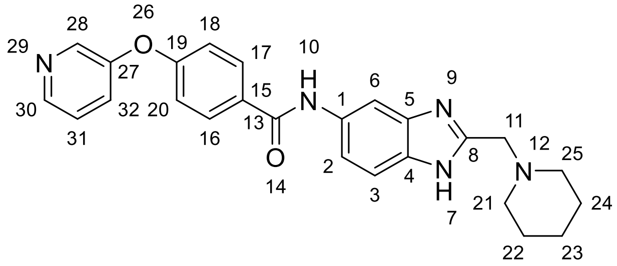


Synthesised according to general procedure **B** to give **157** (0.019 g, 0.089 mmol, 41 %) as an off white solid.

**Mpt:** 143.5-145.5 °C; **$\nu_{max}$ (cm$^{-1}$)** 2933, 1646, 1604, 1500, 1423, 1235, 1168, 839, 555; **$^1$H NMR (400 MHz, DMSO-$d_6$) δ** 12.2 (s, 1H, 10), 10.2 (s, 1H, 7), 8.5 – 8.4 (m, 2H, 28, 30), 8.1 – 8.0 (m, 3H, 6, 16, 17), 7.6 (ddd, *J* = 1.4, 2.9, 8.4 Hz, 1H, 31), 7.5 – 7.4 (m, 3H, 2, 3, 32), 7.2 (d, *J* = 8.8 Hz, 2H, 18, 20), 3.7 (s, 2H, 11), 2.5 – 2.4 (m, 4H, 21, 25), 1.5 (t, *J* = 5.7 Hz, 4H, 22, 24), 1.4 (d, *J* = 5.6 Hz, 2H, 23); **$^{13}$C NMR (101 MHz, DMSO-$d_6$) δ** 164.4 (13), 158.9 (27), 152.4 (19), 145.4 (30), 141.6 (28), 130.5 (15), 130.0 (16, 17), 126.8 (31), 124.9 (32), 117.7 (18, 20), 56.4 (11), 54.1 (21, 25), 25.2 (22, 24), 23.6 (23); **LR-ESI-MS**: $C_{25}H_{26}N_5O_2$ [M+H]$^+$ *m/z* found 428.51, cald 428.21; **HR-ESI-MS**: $C_{25}H_{26}N_5O_2$ [M+H]$^+$ *m/z* found 428.2056, cald 428.2087.

4-isobutyl-*N*-(2-(piperidin-1-ylmethyl)-1H-benzo[d]imidazol-5-yl)benzamide **158**

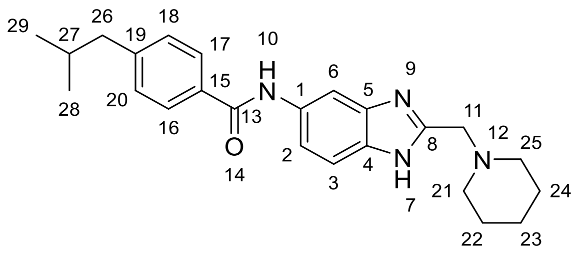

Synthesised according to general procedure **B** to give **158** (0.035 g, 0.090 mmol, 41 %) as a tan oil.

**$v_{max}$ ($cm^{-1}$)** 2931, 1644, 1529, 1452, 835, 750, 555; **$^1$H NMR (400 MHz, DMSO-*d*$_6$) δ** 12.2 (s, 1H, 10), 10.2 (s, 1H, 7), 8.2 – 8.0 (m, 1H, 6), 7.9 (d, *J* = 8.3 Hz, 2H, 16, 17), 7.5 (d, *J* = 1.4 Hz, 2H, 2, 3), 7.4 – 7.3 (m, 2H, 18, 20), 3.8 (s, 2H, 11), 2.6 – 2.5 (m, 6H, 21, 25, 26), 1.9 (dt, *J* = 6.8, 13.5 Hz, 1H, 27), 1.6 (p, *J* = 5.5 Hz, 4H, 22, 24), 1.4 (d, *J* = 6.5 Hz, 2H, 23), 0.9 (d, *J* = 6.6 Hz, 6H, 28, 29); **$^{13}$C NMR (101 MHz, DMSO-*d*$_6$) δ** 165.3 (13), 144.9 (19), 133.8 (4, 5), 132.7 (15), 128.9 (16, 17), 127.5 (18, 20), 115.8 (2, 3), 55.8 (11), 53.9 (21, 25), 44.4 (26), 29.6 (27), 24.9 (22, 24), 23.3 (23), 22.1 (28, 29); **LR-ESI-MS**: $C_{24}H_{31}N_4O$ $[M+H]^+$ *m/z* found 391.60, cald 391.25; **HR-ESI-MS**: $C_{24}H_{31}N_4O$ $[M+H]^+$ *m/z* found 391.2473, cald 391.2498.

4-(acrylamidomethyl)-*N*-(2-(piperidin-1-ylmethyl)-1H-benzo[d]imidazol-5-yl)benzamide **159**

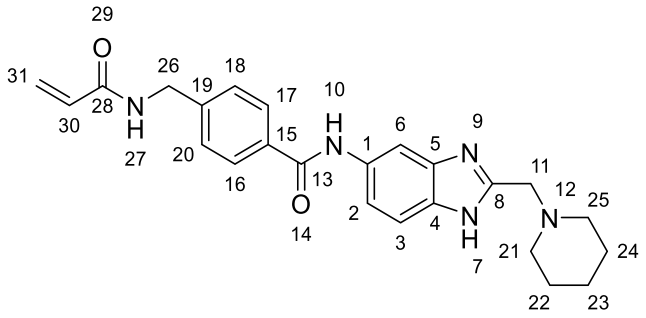

Synthesised according to general procedure **B** to give **159** (0.044 g, 0.108 mmol, 50 %) as an off white solid.

**Mpt:** 151.7-153.7 °C; **$v_{max}$ ($cm^{-1}$)** 2935, 1652, 1530, 1246, 838, 556; **$^1$H NMR (400 MHz, DMSO-*d*$_6$) δ** 12.3 (s, 1H, 10), 10.2 (s, 1H, 7), 8.7 (t, *J* = 6.1 Hz, 1H, 27), 8.1 (s, 1H, 6), 8.0 – 7.8 (m, 2H, 16, 17), 7.6 – 7.2 (m, 4H, 2, 3, 18, 20), 6.3 (dd, *J* = 10.2, 17.1 Hz, 1H, 31''), 6.1 (dd, *J* = 2.3, 17.1 Hz, 1H, 31'), 5.6 (dd, *J* = 2.2, 10.1 Hz, 1H, 30), 4.4 (d, *J* = 6.1 Hz, 2H, 26), 3.7 (s, 2H, 11), 2.4 (t, *J* = 5.5 Hz, 4H, 21, 25), 1.5 (t, *J* = 5.7 Hz, 4H, 22, 24), 1.4 (d, *J* = 6.0 Hz, 2H, 23); **$^{13}$C NMR (101 MHz, DMSO-*d*$_6$) δ** 165.0 (28), 164.8 (13), 142.9 (19), 133.8 (31), 131.6 (15), 127.8 (16, 17), 127.2 (18, 20), 125.6 (30), 56.5 (11), 54.1 (21, 25), 25.3 (22, 24), 23.7 (23); **LR-ESI-MS**: $C_{24}H_{28}N_5O_2$ $[M+H]^+$ *m/z* found 418.34, cald 418.22; **HR-ESI-MS**: $C_{24}H_{28}N_5O_2$ $[M+H]^+$ *m/z* found 418.2213, cald 418.2243.

4-((2-oxopyrrolidin-1-yl)methyl)-*N*-(2-(piperidin-1-ylmethyl)-1H-benzo[d]imidazol-5-yl)benzamide **160**

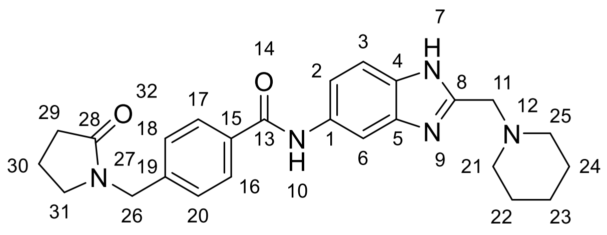


Synthesised according to general procedure **B** to give **160** (0.042 g, 0.097 mmol, 45 %) as a yellow solid.

**Mpt:** 155.5-157.5 °C; **$v_{max}$ (cm$^{-1}$)** 2934, 1651, 1531, 1266, 836, 556; **$^1$H NMR (400 MHz, DMSO-*d*$_6$) δ** 12.3 (s, 1H, 10), 10.2 (s, 1H, 7), 8.1 (s, 1H, 6), 7.9 (d, *J* = 8.3 Hz, 2H, 16, 17), 7.5 (s, 2H, 2, 3), 7.4 (d, *J* = 8.3 Hz, 2H, 18, 20), 4.5 (s, 2H, 26), 3.7 (s, 2H, 11), 3.3 (t, *J* = 7.0 Hz, 2H, 31), 2.5 (t, *J* = 4.4 Hz, 4H, 21, 25), 2.3 (t, *J* = 8.1 Hz, 2H, 29), 1.9 (dq, *J* = 7.3, 10.9 Hz, 2H, 30), 1.5 (p, *J* = 5.5 Hz, 4H, 22, 24), 1.4 (q, *J* = 5.3, 5.7 Hz, 2H, 23); **$^{13}$C NMR (101 MHz, DMSO-*d*$_6$) δ** 174.2 (28), 165.0 (13), 140.6 (19), 134.2 (15), 129.6 (1), 128.0 (16, 17), 127.6 (4), 127.5 (18, 20), 56.3 (11), 54.0 (21, 25), 46.2 (26), 45.2 (31), 30.2 (29), 25.2 (22, 24), 23.5 (23), 17.4 (30); **LR-ESI-MS**: $C_{25}H_{30}N_5O_2$ [M+H]$^+$ *m/z* found 432.66, cald 432.24; **HR-ESI-MS**: $C_{25}H_{30}N_5O_2$ [M+H]$^+$ *m/z* found 432.2373, cald 432.2400.

*N*-(2-(piperidin-1-ylmethyl)-1H-benzo[d]imidazol-5-yl)-4-(pyridin-3-ylmethoxy)benzamide **161**

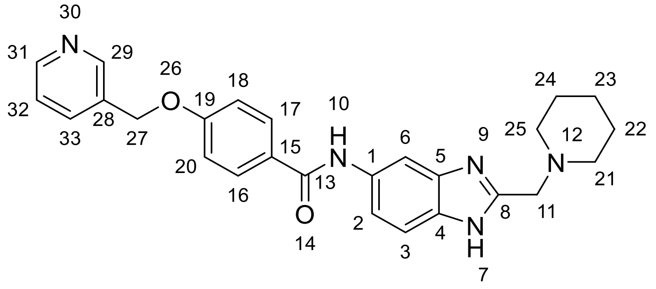


Synthesised according to general procedure **B** to give **161** (0.031 g, 0.07 mmol, 32 %) as an off white solid.

**Mpt:** 163.2-165.2 °C; **$v_{max}$ (cm$^{-1}$)** 2934, 1640, 1604, 1506, 1427, 1244, 1179, 837, 556; **$^1$H NMR (400 MHz, DMSO-*d*$_6$) δ** 12.2 (s, 1H, 10), 10.1 (s, 1H, 7), 8.7 (d, *J* = 2.2 Hz, 1H, 29), 8.6 – 8.5 (m, 1H, 31), 8.1 (s, 1H, 6), 8.0 – 7.9 (m, 2H, 16, 17), 7.9 (dt, *J* = 2.0, 7.8 Hz, 1H, 33), 7.5 – 7.3 (m, 3H, 2, 3, 32), 7.2 – 7.1 (m, 2H, 18, 20), 5.3 (s, 2H, 27), 3.7 (s, 2H, 11), 2.5 (s, 4H, 21, 25), 1.5 (q, *J* = 5.6 Hz, 4H, 22, 24), 1.4 (q, *J* = 5.8 Hz, 2H, 23); **$^{13}$C NMR (101 MHz, DMSO-*d*$_6$) δ** 164.6 (13), 160.5 (19), 149.3 (31), 149.2 (29), 135.8 (15), 132.3 (33), 129.6 (16, 17), 127.7 (28), 123.7 (32), 114.4 (18, 20), 67.1 (27), 56.4 (11), 54.1 (21, 25), 25.3 (22, 24), 23.6 (23); **LR-ESI-MS**: $C_{26}H_{28}N_5O_2$ [M+H]$^+$ *m/z* found 442.54, cald 442.22; **HR-ESI-MS**: $C_{26}H_{28}N_5O_2$ [M+H]$^+$ *m/z* found 442.2211, cald 442.2243.

4-((1H-imidazol-1-yl)methyl)-*N*-(2-(piperidin-1-ylmethyl)-1H-benzo[d]imidazol-5-yl)benzamide **162**

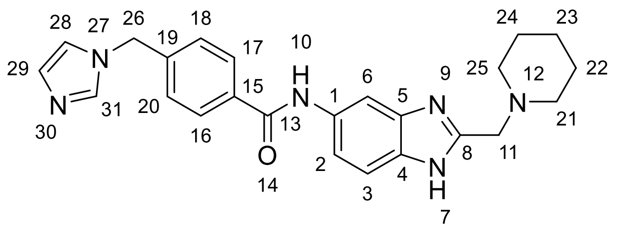

Synthesised according to general procedure **B** to give **162** (0.014 g, 0.034 mmol, 16 %) as an off white solid.

**Mpt:** 127.7-129.7 °C; $\mathbf{v_{max}}$ **(cm$^{-1}$)** 2932, 1646, 1534, 1484, 807, 722; **$^{1}$H NMR (400 MHz, DMSO-*d*$_6$) δ** 12.3 (s, 1H, 10), 10.2 (s, 1H, 7), 8.0 (s, 1H, 6), 8.0 (d, *J* = 8.3 Hz, 2H, 16, 17), 7.8 (d, *J* = 1.2 Hz, 1H, 31), 7.4 (s, 2H, 2, 3), 7.4 – 7.3 (m, 2H, 18, 20), 7.2 (d, *J* = 1.3 Hz, 1H, 28), 6.9 (d, *J* = 1.1 Hz, 1H, 29), 5.3 (s, 2H, 26), 3.6 (s, 2H, 11), 2.5 – 2.3 (m, 4H, 21, 25), 1.5 (p, *J* = 5.5 Hz, 4H, 22, 24), 1.4 (q, *J* = 5.9 Hz, 2H, 23); **$^{13}$C NMR (101 MHz, DMSO-*d*$_6$) δ** 164.9 (13), 141.1 (31), 137.6 (29), 134.6 (15), 128.8 (19), 128.0 (16, 17), 127.3 (18, 20), 119.6 (28), 56.6 (11), 54.1 (21, 25), 49.1 (26), 25.4 (22, 24), 23.8 (23); **LR-ESI-MS**: $C_{24}H_{27}N_6O$ [M+H]$^+$ *m/z* found 415.53, cald 415.23; **HR-ESI-MS**: $C_{24}H_{26}N_6NaO$ [M+Na]$^+$ *m/z* found 437.2026, cald 437.2066.

4-(2-(dimethylamino)ethyl)-*N*-(2-(piperidin-1-ylmethyl)-1H-benzo[d]imidazol-5-yl)benzamide **163**

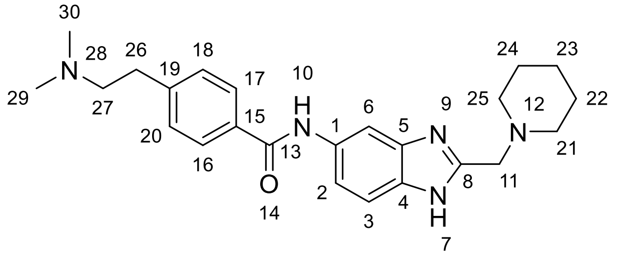

Synthesised according to general procedure **B** to give **163** (0.051 g, 0.126 mmol, 58 %) as a tan solid.

**Mpt:** 217.9-219.9 °C; $\mathbf{v_{max}}$ **(cm$^{-1}$)** 3292, 2940, 2798, 1636, 1451, 813; **$^{1}$H NMR (400 MHz, DMSO-*d*$_6$) δ** 12.2 (s, 1H, 10), 10.1 (s, 1H, 7), 8.2 – 7.9 (m, 1H, 6), 7.9 (d, *J* = 8.3 Hz, 2H, 16, 17), 7.6 – 7.2 (m, 4H, 2, 3, 18, 20), 3.6 (s, 2H, 11), 2.8 (t, *J* = 7.5 Hz, 2H, 27), 2.5 – 2.5 (m, 2H, 26), 2.4 (t, *J* = 5.1 Hz, 4H, 21, 25), 2.2 (s, 6H, 29, 30), 1.5 (p, *J* = 5.5 Hz, 4H, 22, 24), 1.4 – 1.3 (m, 2H, 23); **$^{13}$C NMR (101 MHz, DMSO-*d*$_6$) δ** 165.2 (13), 144.3 (19), 132.9 (15), 128.6 (16, 17), 127.6 (18, 20), 60.5 (27), 56.7 (11), 54.2 (21, 25), 45.1 (29, 30), 33.1 (26), 25.5 (22, 24), 23.8 (23); **LR-ESI-MS**: $C_{24}H_{32}N_5O$ [M+H]$^+$ *m/z* found 406.40, cald 406.26; **HR-ESI-MS**: $C_{24}H_{32}N_5O$ [M+H]$^+$ *m/z* found 406.2581, cald 406.2607.

*N*$_1$, *N*$_1$-dimethyl-*N*$_4$-(2-(piperidin-1-ylmethyl)-1H-benzo[d]imidazol-5-yl)terephthalamide **164**

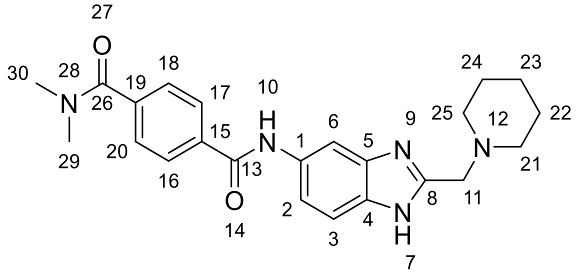

Synthesised according to general procedure **B** to give **164** (0.030 g, 0.074 mmol, 34 %) as a white solid.

**Mpt:** 143.0-145.0 °C; **$v_{max}$ (cm$^{-1}$)** 2930, 1611, 1264, 859, 724; **$^{1}$H NMR (400 MHz, DMSO-*d*$_6$) δ** 12.2 (s, 1H, 10), 10.3 (s, 1H, 7), 8.1 (s, 1H, 6), 8.0 – 7.9 (m, 2H, 16, 17), 7.5 (d, *J* = 8.3 Hz, 2H, 18, 20), 7.5 (s, 2H, 2, 3), 3.7 (s, 2H, 11), 3.0 (s, 3H, 29), 2.9 (s, 3H, 30), 2.4 (t, *J* = 5.4 Hz, 4H, 21, 25), 1.5 (q, *J* = 5.6 Hz, 4H, 22, 24), 1.4 – 1.3 (m, 2H, 23); **$^{13}$C NMR (101 MHz, DMSO-*d*$_6$) δ** 169.5 (26), 164.7 (13), 139.2 (15), 135.8 (19), 127.7 (16, 17), 126.9 (18, 20), 56.6 (11), 54.1 (21, 25), 34.7 (29, 30), 25.4 (22, 24), 23.7 (23); **LR-ESI-MS**: $C_{23}H_{28}N_5O_2$ $[M+H]^+$ *m/z* found 406.59, cald 406.22; **HR-ESI-MS**: $C_{23}H_{28}N_5O_2$ $[M+H]^+$ *m/z* found 406.2223, cald 406.2243.

4-((2-methoxyethoxy)methyl)-*N*-(2-(piperidin-1-ylmethyl)-1H-benzo[d]imidazol-5-yl)benzamide **165**

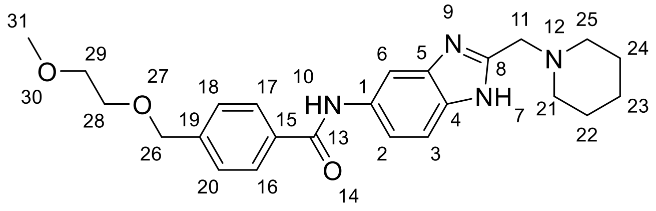

Synthesised according to general procedure **B** to give **165** (0.056 g, 0.133 mmol, 61 %) as an orange solid.

**Mpt:** 101.4-103.4 °C; **$v_{max}$ (cm$^{-1}$)** 2932, 1646, 1529, 1449, 1081, 836, 555; **$^{1}$H NMR (400 MHz, DMSO-*d*$_6$) δ** 12.2 (s, 1H, 10), 10.2 (s, 1H, 7), 8.1 (d, *J* = 1.3 Hz, 1H, 6), 8.0 (d, *J* = 8.3 Hz, 2H, 16, 17), 7.5 – 7.4 (m, 4H, 2, 3, 18, 20), 4.6 (s, 2H, 26), 3.8 (s, 2H, 11), 3.6 – 3.5 (m, 2H, 28), 3.5 – 3.4 (m, 2H, 29), 3.3 (s, 3H, 31), 2.6 (d, *J* = 6.3 Hz, 4H, 21, 25), 1.6 (t, *J* = 5.7 Hz, 4H, 22, 24), 1.4 (d, *J* = 5.9 Hz, 2H, 23); **$^{13}$C NMR (101 MHz, DMSO-*d*$_6$) δ** 165.1 (13), 150.6 (8), 142.1 (19), 134.2 (15), 133.8 (5), 128.4 (4), 127.7 (16, 17), 127.1 (18, 20), 120.2 (3), 115.8 (2), 71.5 (26), 71.3 (28), 69.2 (29), 58.2 (31), 55.7 (11), 53.8 (21, 25), 24.8 (22, 24), 23.2 (23); **LR-ESI-MS**: $C_{24}H_{31}N_4O_3$ $[M+H]^+$ *m/z* found 423.58, cald 423.24; **HR-ESI-MS**: $C_{24}H_{31}N_4O_3$ $[M+H]^+$ *m/z* found 423.2370, cald 423.2396.

4-(2-hydroxyethoxy)-*N*-(2-(piperidin-1-ylmethyl)-1H-benzo[d]imidazol-5-yl)benzamide **166**

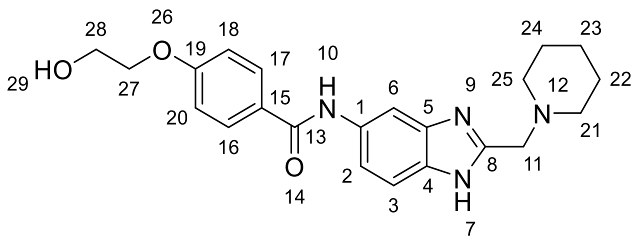

Synthesised according to general procedure **B** to give **166** (0.015 g, 0.039 mmol, 18 %) as an off white solid.

**Mpt:** 147.3-149.3 °C; **$v_{max}$ (cm$^{-1}$)** 2930, 1603, 1246, 1178, 807, 622; **$^{1}$H NMR (400 MHz, DMSO-*d*$_6$) δ** 12.2 (s, 1H, 10), 10.1 (s, 1H, 7), 8.0 (s, 1H, 6), 8.0 – 7.9 (m, 2H, 16, 17), 7.4 (s, 2H, 2, 3), 7.2 – 7.0 (m, 2H, 18, 20), 5.0 (t, *J* = 5.5 Hz, 1H, 29), 4.1 (t, *J* = 4.9 Hz, 2H, 28), 3.7 (d, *J* = 4.8 Hz, 2H, 27), 3.7 (s, 2H, 11), 2.5 – 2.3 (m, 4H, 21, 25), 1.5 (p, *J* = 5.5 Hz, 4H, 22, 24), 1.4 (q, *J* = 6.0 Hz, 2H, 23); **$^{13}$C NMR (101 MHz, DMSO-*d*$_6$) δ** 164.7 (13), 161.2 (19), 129.5 (16, 17), 127.2 (15), 114.0 (18, 20), 69.8 (28), 59.5 (27), 56.6 (11), 54.1 (21, 25), 25.4 (22, 24), 23.7 (23); **LR-ESI-MS**: $C_{22}H_{27}N_4O_3$ $[M+H]^+$ *m/z* found 395.53, cald 395.21; **HR-ESI-MS**: $C_{22}H_{27}N_4O_3$ $[M+H]^+$ *m/z* found 395.2059, cald 395.2083.

4-((methylsulfonyl)methyl)-*N*-(2-(piperidin-1-ylmethyl)-1H-benzo[d]imidazol-5-yl)benzamide **167**

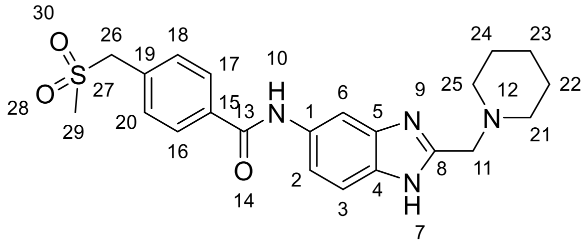

Synthesised according to general procedure **B** to give **167** (0.033 g, 0.077 mmol, 36 %) as a white solid.

**Mpt:** 163.4-165.4 °C; **$v_{max}$ (cm$^{-1}$)** 2931, 1647, 1530, 1292, 839, 556; **$^{1}$H NMR (400 MHz, DMSO-*d*$_6$) δ** 12.2 (s, 1H, 10), 10.3 (s, 1H, 7), 8.1 (s, 1H, 6), 8.0 – 7.9 (m, 2H, 16, 17), 7.6 (d, *J* = 8.4 Hz, 2H, 18, 20), 7.4 (s, 2H, 2, 3), 4.6 (s, 2H, 26), 3.7 (s, 2H, 11), 2.9 (s, 3H, 29), 2.4 (t, *J* = 5.4 Hz, 4H, 21, 25), 1.5 (t, *J* = 5.7 Hz, 4H, 22, 24), 1.4 – 1.3 (m, 2H, 23); **$^{13}$C NMR (101 MHz, DMSO-*d*$_6$) δ** 165.0 (13), 135.3 (19), 132.4 (15), 130.9 (16, 17), 127.8 (18, 20), 59.1 (26), 56.5 (11), 54.1 (21, 25), 30.7 (29), 25.4 (22, 24), 23.7 (23); **LR-ESI-MS**: $C_{22}H_{27}N_4O_3S$ $[M+H]^+$ *m/z* found 427.49, cald 427.18; **HR-ESI-MS**: $C_{22}H_{27}N_4O_3S$ $[M+H]^+$ *m/z* found 427.1777, cald 427.1804.

(*R*)-*N*-(2-((2-methylpyrrolidin-1-yl)methyl)-1H-benzo[d]imidazol-5-yl)-4-(pyridin-4-yl)benzamide **168**

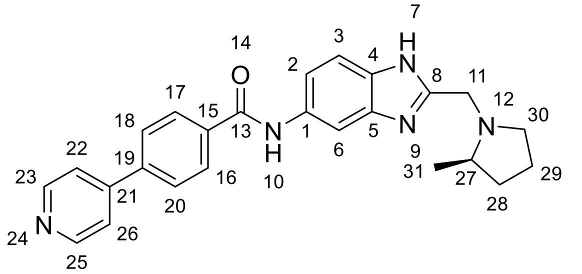

Synthesised according to general procedure **B** to give **168** (0.051 g, 0.124 mmol, 57 %) as a salmon pink solid.

**Mpt:** 162.4-164.4 °C; **$\nu_{max}$ (cm$^{-1}$)** 2959, 2796, 1645, 1597, 1532, 1483, 1415, 843, 817, 756; **$^1$H NMR (400 MHz, DMSO-*d*$_6$) δ** 12.2 (s, 1H, 10), 10.3 (s, 1H, 7), 8.8 – 8.6 (m, 2H, 23, 25), 8.1 (d, *J* = 8.5 Hz, 2H, 16, 17), 8.1 (s, 1H, 6), 8.0 – 7.9 (m, 2H, 18, 20), 7.8 – 7.7 (m, 2H, 22, 26), 7.5 (s, 2H, 2, 3), 4.1 (d, *J* = 14.1 Hz, 1H, 11''), 3.6 (d, *J* = 14.3 Hz, 1H, 11'), 3.0 – 2.9 (m, 1H, 27), 2.5 (s, 1H, 30'), 2.3 (d, *J* = 8.9 Hz, 1H, 30''), 2.0 – 1.9 (m, 1H, 28''), 1.7 (ddt, *J* = 6.4, 8.9, 12.6 Hz, 2H, 28', 29''), 1.4 (dddd, *J* = 6.3, 8.4, 9.9, 12.2 Hz, 1H, 29'), 1.1 (d, *J* = 6.0 Hz, 3H, 31); **$^{13}$C NMR (101 MHz, DMSO-*d*$_6$) δ** 164.6 (13), 150.4 (23, 25), 146.0 (21), 139.8 (19), 135.7 (15), 128.5 (16, 17), 126.8 (18, 20), 121.4 (22, 26), 59.2 (11), 54.0 (27), 50.8 (30), 32.5 (28), 21.4 (29), 18.7 (31); **LR-ESI-MS**: $C_{25}H_{26}N_5O$ [M+H]$^+$ *m/z* found 412.61, cald 412.21; **HR-ESI-MS**: $C_{25}H_{26}N_5O$ [M+H]$^+$ *m/z* found 412.2110, cald 412.2137.

*N*-(2-(((trans)-3,4-dimethylpyrrolidin-1-yl)methyl)-1H-benzo[d]imidazol-5-yl)-1-methyl-1H-indazole-5-carboxamide **169**

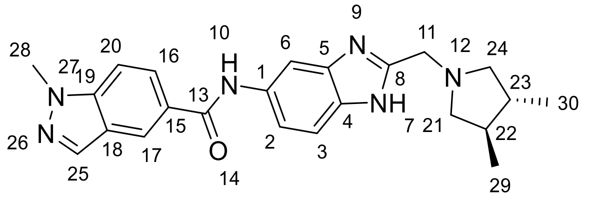

Synthesised according to general procedure **B** to give **169** (0.039 g, 0.097 mmol, 47 %) as an off white solid.

**Mpt:** 136.0-138.0 °C; **$\nu_{max}$ (cm$^{-1}$)** 2954, 1618, 1450, 1191, 837, 556; **$^1$H NMR (400 MHz, DMSO-*d*$_6$) δ** 12.2 (s, 1H, 10), 10.3 (s, 1H, 7), 8.5 (dd, *J* = 0.8, 1.7 Hz, 1H, 17), 8.2 (d, *J* = 0.9 Hz, 1H, 25), 8.1 (s, 1H, 6), 8.0 (dd, *J* = 1.6, 8.9 Hz, 1H, 16), 7.8 (dt, *J* = 0.9, 8.9 Hz, 1H, 20), 7.5 (d, *J* = 2.2 Hz, 2H, 2, 3), 4.1 (s, 3H, 28), 4.1 – 3.9 (m, 2H, 11''), 3.0 (t, *J* = 8.1 Hz, 2H, 21'', 24'), 2.5 (t, *J* = 8.8 Hz, 2H, 21', 24''), 1.8 – 1.6 (m, 2H, 22, 23), 1.0 (d, *J* = 6.2 Hz, 6H, 29, 30); **$^{13}$C NMR (101 MHz, DMSO-*d*$_6$) δ** 165.4 (13), 140.6 (19), 133.9 (25), 127.6 (17), 125.6 (16), 122.9 (15), 121.3 (18), 109.5 (20), 61.4 (11), 53.1 (21), 40.0 (23), 35.6 (28), 17.5 (29, 30); **LR-ESI-MS**: $C_{23}H_{27}N_6O$ [M+H]$^+$ *m/z* found 403.41, cald 403.23; **HR-ESI-MS**: $C_{23}H_{27}N_6O$ [M+H]$^+$ *m/z* found 403.2223, cald 403.2246.

*N*-(2-(((trans)-3,4-dimethylpyrrolidin-1-yl)methyl)-1H-benzo[d]imidazol-5-yl)-4-(pyridin-4-yl)benzamide **170**

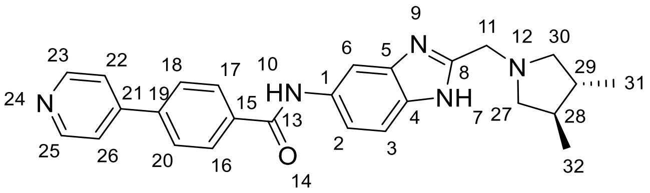


Synthesised according to general procedure **B** to give **170** (0.029 g, 0.07 mmol, 34 %) as a white solid.

**Mpt:** 211.5-213.5 °C; **$v_{max}$ (cm$^{-1}$)** 3302, 2947, 1638, 1594, 1573, 1283, 813, 752, 650; **$^1$H NMR (400 MHz, DMSO-*d*$_6$) δ** 12.3 (d, *J* = 11.1 Hz, 1H, 10), 10.5 – 10.2 (m, 1H, 7), 8.7 (q, *J* = 5.7 Hz, 2H, 23, 25), 8.3 – 8.1 (m, 3H, 16, 17), 8.1 – 7.9 (m, 2H, 22, 26), 7.8 (q, *J* = 5.7 Hz, 2H, 18, 20), 7.7 – 7.3 (m, 2H, 2, 3), 3.9 – 3.7 (m, 2H, 11''), 2.9 – 2.7 (m, 2H, 27'', 30''), 2.3 (p, *J* = 8.0 Hz, 2H, 27', 30'), 1.7 (tt, *J* = 6.1, 10.9 Hz, 2H, 28, 29), 1.0 (q, *J* = 6.0 Hz, 6H, 31, 32); **$^{13}$C NMR (101 MHz, DMSO-*d*$_6$) δ** 164.6 (13), 150.4 (23, 25), 146.0 (21), 139.8 (19), 135.7 (15), 134.4 (5), 133.7 (4), 128.5 (16, 17), 126.8 (18, 20), 121.4 (22, 26), 118.1 (2), 115.1 (3), 103.3 (6), 61.7 (27, 30), 53.6 (11), 40.4 (28, 29), 18.3 (31, 32); **LR-ESI-MS**: $C_{26}H_{28}N_5O$ [M+H]$^+$ *m/z* found 426.44, cald 426.23; **HR-ESI-MS**: $C_{26}H_{28}N_5O$ [M+H]$^+$ *m/z* found 426.2267, cald 426.2294.

*N*-(2-(piperidin-1-ylmethyl)-1H-benzo[d]imidazol-5-yl)-4-(pyrrolidin-1-ylmethyl)benzamide **171**

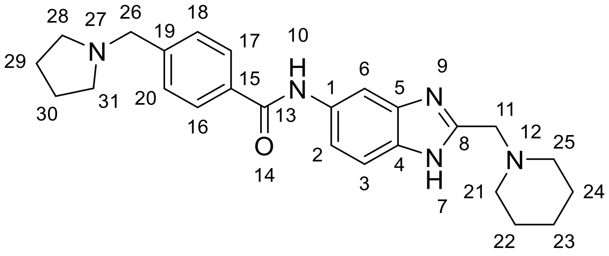


Synthesised according to general procedure **B** to give **171** (0.078 g, 0.187 mmol, 86 %) as a yellow solid.

**Mpt:** 137.7-139.7 °C; **$v_{max}$ (cm$^{-1}$)** 2930, 2790, 1642, 1529, 1277, 1107, 852, 805; **$^1$H NMR (400 MHz, DMSO-*d*$_6$) δ** 12.2 (s, 1H, 10), 10.2 (d, *J* = 22.2 Hz, 1H, 7), 8.0 (d, *J* = 35.6 Hz, 1H, 6), 7.9 (d, *J* = 8.2 Hz, 2H, 16, 17), 7.4 (dd, *J* = 8.6, 17.5 Hz, 4H, 2, 3, 18, 20), 3.6 (s, 4H, 11, 26), 2.5 – 2.3 (m, 8H, 21, 25, 28, 31), 1.8 – 1.7 (m, 4H, 29, 30), 1.5 (p, *J* = 5.6 Hz, 4H, 22, 24), 1.4 (q, *J* = 4.7, 6.1 Hz, 2H, 23); **$^{13}$C NMR (101 MHz, DMSO-*d*$_6$) δ** 165.2 (13), 152.1 (8), 143.1 (19), 139.6 (5), 134.4 (4), 133.7 (15), 128.3 (16, 17), 127.6 (18, 20), 118.0 (3), 115.0 (2), 103.2 (6), 59.3 (26), 56.7 (11), 54.2 (21, 25), 53.5 (28, 31), 25.5 (22, 24), 23.8 (23), 23.2 (29, 30); **LR-ESI-MS**: $C_{25}H_{32}N_5O$ [M+H]$^+$ *m/z* found 418.59, cald 418.26; **HR-ESI-MS**: $C_{25}H_{32}N_5O$ [M+H]$^+$ *m/z* found 418.2580, cald 418.2607.

(*R*)-4-(1H-imidazol-1-yl)-*N*-(2-((3-methylpyrrolidin-1-yl)methyl)-1H-benzo[d]imidazol-5-yl)benzamide **172**

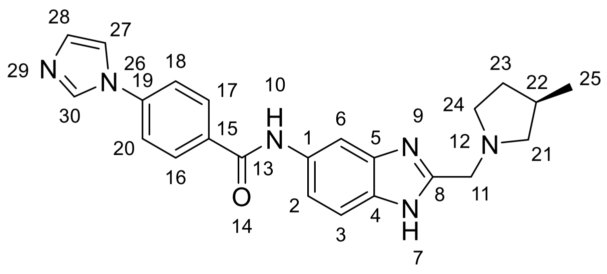

Synthesised according to general procedure **B** to give **172** (0.037 g, 0.09 mmol, 100 %) as a yellow solid.

**Mpt:** 285.2-287.2 °C; **$v_{max}$ (cm$^{-1}$)** 2955, 1646, 1606, 1517, 1483, 1425, 1300, 1244, 1058, 815; **$^1$H NMR (400 MHz, DMSO-*d*$_6$) δ** 12.3 (s, 1H, 10), 10.3 (s, 1H, 7), 8.4 (t, *J* = 1.2 Hz, 1H, 30), 8.1 (d, *J* = 8.8 Hz, 2H, 16, 17), 8.1 (d, *J* = 14.1 Hz, 1H, 6), 7.9 (t, *J* = 1.4 Hz, 1H, 28), 7.9 – 7.8 (m, 2H, 18, 20), 7.5 (s, 2H, 2, 3), 7.2 (t, *J* = 1.2 Hz, 1H, 27), 3.8 (d, *J* = 1.6 Hz, 2H, 11'), 2.8 (dd, *J* = 7.2, 8.8 Hz, 1H, 21'), 2.7 – 2.7 (m, 1H, 21''), 2.6 (td, *J* = 5.9, 8.7 Hz, 1H, 24'), 2.2 (qd, *J* = 2.2, 6.7 Hz, 1H, 24''), 2.2 – 2.1 (m, 1H, 23''), 2.1 – 1.9 (m, 1H, 22), 1.3 (ddd, *J* = 2.3, 6.2, 12.4 Hz, 1H, 23'), 1.0 (d, *J* = 6.7 Hz, 3H, 25); **$^{13}$C NMR (101 MHz, DMSO-*d*$_6$) δ** 164.2 (13), 139.0 (30), 135.7 (19), 133.1 (15), 131.0 (1), 130.3 (28), 129.4 (16, 17), 119.7 (2, 3), 119.5 (18, 20), 117.8 (27), 61.6 (11), 53.7 (21), 53.2 (24), 32.4 (22), 31.5 (23), 20.2 (25); **LR-ESI-MS**: $C_{23}H_{25}N_6O$ [M+H]$^+$ *m/z* found 401.54, cald 401.21; **HR-ESI-MS**: $C_{23}H_{25}N_6O$ [M+H]$^+$ *m/z* found 401.2063, cald 401.2090.

*N*-(2-(((trans)-3,4-dimethylpyrrolidin-1-yl)methyl)-1H-benzo[d]imidazol-5-yl)-4-(1H-imidazol-1-yl)benzamide **173**

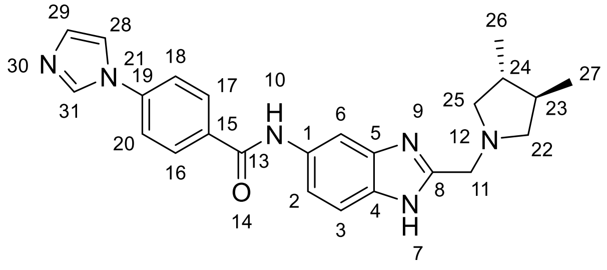

Synthesised according to general procedure **B** to give **173** (0.026 g, 0.063 mmol, 73 %) as an off white solid.

**Mpt:** 165.0-167.0 °C; **$v_{max}$ (cm$^{-1}$)** 2953, 1608, 1514, 1302, 1249, 839, 555; **$^1$H NMR (400 MHz, DMSO-*d*$_6$) δ** 12.3 (s, 1H, 10), 10.3 (s, 1H, 7), 8.4 (d, *J* = 1.2 Hz, 1H, 31), 8.1 (d, *J* = 8.7 Hz, 2H, 16, 17), 8.1 (s, 1H, 6), 7.9 (t, *J* = 1.4 Hz, 1H, 28), 7.9 (d, *J* = 8.7 Hz, 2H, 18, 20), 7.5 (s, 2H, 2, 3), 7.2 (t, *J* = 1.1 Hz, 1H, 29), 3.9 – 3.8 (m, 2H, 11), 3.0 – 2.8 (m, 2H, 22'', 25'), 2.3 (dd, *J* = 6.7, 9.1 Hz, 2H, 22', 25''), 1.8 – 1.6 (m, 2H, 23, 24), 1.0 (d, *J* = 6.2 Hz, 6H, 26, 27); **$^{13}$C NMR (101 MHz, DMSO-*d*$_6$) δ** 164.2 (13), 139.0 (31), 135.7 (19), 133.1 (29), 130.3 (15), 129.4 (16, 17), 119.5 (18, 20), 117.9 (28), 61.6 (22, 25), 53.4 (11), 40.3 (23, 24), 18.1 (26, 27); **LR-ESI-MS**: $C_{24}H_{27}N_6O$ [M+H]$^+$ *m/z* found 415.58, cald 415.23; **HR-ESI-MS**: $C_{24}H_{26}N_6NaO$ [M+H]$^+$ *m/z* found 437.2025, cald 437.2066.

*N*-(2-(((1*S*,4*S*)-2-oxa-5-azabicyclo[2.2.1]heptan-5-yl)methyl)-1H-benzo[d]imidazol-5-yl)-1-methyl-1H-indazole-5-carboxamide **174**

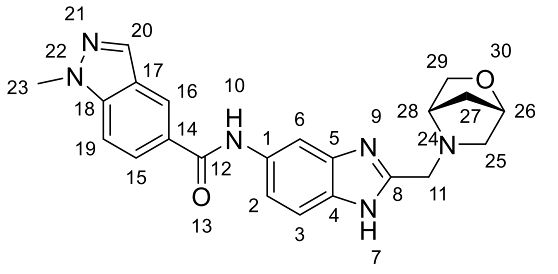

Synthesised according to general procedure **B** to give **174** (0.034 g, 0.084 mmol, 41 %) as an off white solid.

**Mpt:** 146.7-148.7 °C; **$\nu_{max}$ (cm$^{-1}$)** 2954, 1644, 1193, 950; **$^{1}$H NMR (400 MHz, DMSO-*d*$_6$) δ** 12.2 (s, 1H, 10), 10.2 (s, 1H, 7), 8.5 (dd, *J* = 0.8, 1.7 Hz, 1H, 16), 8.2 (d, *J* = 0.9 Hz, 1H, 20), 8.1 (d, *J* = 9.4 Hz, 1H, 6), 8.0 (dd, *J* = 1.6, 8.9 Hz, 1H, 15), 7.8 (dt, *J* = 0.9, 8.9 Hz, 1H, 19), 7.5 (s, 2H, 2, 3), 4.4 (t, *J* = 2.0 Hz, 1H, 29'), 4.1 (s, 3H, 23), 4.0 – 3.8 (m, 3H, 11'', 26, 29''), 3.6 – 3.5 (m, 2H, 11', 28), 2.8 (dd, *J* = 1.7, 9.9 Hz, 1H, 25''), 2.6 (d, *J* = 10.0 Hz, 1H, 25'), 1.8 (dd, *J* = 2.1, 9.8 Hz, 1H, 27''), 1.7 – 1.6 (m, 1H, 27'); **$^{13}$C NMR (101 MHz, DMSO-*d*$_6$) δ** 165.4 (12), 140.6 (18), 133.9 (15), 127.6 (20), 125.6 (16), 122.9 (14), 121.3 (17), 109.5 (19), 76.2 (26), 69.4 (29), 61.2 (28), 60.6 (25), 51.7 (11), 35.6 (23), 34.4 (27); **LR-ESI-MS**: $C_{22}H_{23}N_6O_2$ $[M+H]^+$ *m/z* found 403.47, cald 403.19; **HR-ESI-MS**: $C_{22}H_{23}N_6O_2$ $[M+H]^+$ *m/z* found 403.1860, cald 403.1882.

*N*-(2-((2,2-dimethylpyrrolidin-1-yl)methyl)-1H-benzo[d]imidazol-5-yl)-4-(1H-imidazol-1-yl)benzamide **175**

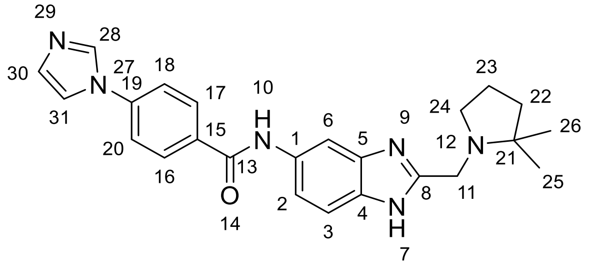

Synthesised according to general procedure **B** to give **175** (0.0054 g, 0.013 mmol, 6 %) as a yellow film.

**$\nu_{max}$ (cm$^{-1}$)** 2958, 1608, 1514, 1483, 1301, 1249, 1054, 962, 848, 809, 655; **$^{1}$H NMR (400 MHz, DMSO-*d*$_6$) δ** 12.0 (s, 1H, 10), 10.3 (s, 1H, 7), 8.4 (t, *J* = 1.2 Hz, 1H, 28), 8.2 – 8.0 (m, 2H, 6, 16, 17), 7.9 (t, *J* = 1.4 Hz, 1H, 30), 7.9 – 7.8 (m, 2H, 18, 20), 7.4 (d, *J* = 17.6 Hz, 2H, 2, 3), 7.2 (d, *J* = 1.2 Hz, 1H, 31), 3.7 (s, 2H, 11), 2.7 (t, *J* = 6.8 Hz, 3H, 24), 1.8 – 1.6 (m, 4H, 22, 23), 1.1 (s, 6H, 25, 26); **$^{13}$C NMR (101 MHz, DMSO-*d*$_6$) δ** 139.0 (28), 135.7 (30), 133.1 (15), 130.3 (19), 129.4 (16, 17), 119.5 (18, 20), 117.8 (31), 59.8 (21, 24), 51.3 (11), 46.8 (22), 22.7 (25, 26), 20.2 (23); **LR-ESI-MS**: $C_{24}H_{27}N_6O$ $[M+H]^+$ *m/z* found 415.51, cald 415.23; **HR-ESI-MS**: $C_{24}H_{26}N_6NaO$ $[M+H]^+$ *m/z* found 437.2027, cald 437.2066.

*N*-(2-((2-isopropylpyrrolidin-1-yl)methyl)-1H-benzo[d]imidazol-5-yl)-1-methyl-1H-indazole-5-carboxamide **176**

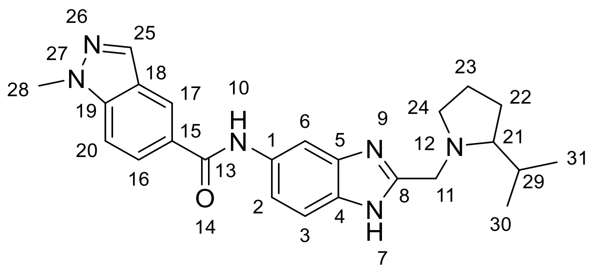

Synthesised according to general procedure **B** to give **176** (0.019 g, 0.044 mmol, 15 %) as an off white solid.

**Mpt:** 256.8-258.8 °C; **$v_{max}$ (cm$^{-1}$)** 2954, 2794, 1640, 1544, 1411, 1314, 1190, 1109, 810, 759, 616; **$^{1}$H NMR (400 MHz, DMSO-*d*$_6$) δ** 12.1 (s, 1H, 10), 10.3 (s, 1H, 7), 8.5 (dd, *J* = 0.8, 1.6 Hz, 1H, 17), 8.2 (d, *J* = 0.9 Hz, 1H, 25), 8.1 (s, 1H, 6), 8.0 (dd, *J* = 1.7, 8.8 Hz, 1H, 16), 7.8 (dt, *J* = 0.9, 8.9 Hz, 1H, 20), 7.5 (d, *J* = 19.1 Hz, 2H, 2, 3), 4.1 (s, 3H, 28), 4.0 (d, *J* = 14.3 Hz, 1H, 11''), 3.5 (d, *J* = 14.1 Hz, 1H, 11'), 2.9 (ddd, *J* = 2.1, 6.2, 8.2 Hz, 1H, 21), 2.5 (s, 1H, 29), 2.4 (dd, *J* = 2.8, 6.9 Hz, 1H, 24'), 2.3 (dtd, *J* = 2.6, 6.5, 9.2 Hz, 1H, 24''), 1.9 (td, *J* = 4.4, 6.8 Hz, 1H, 22''), 1.7 – 1.4 (m, 3H, 22', 23), 0.9 (dd, *J* = 2.5, 6.8 Hz, 6H, 30, 31); **$^{13}$C NMR (101 MHz, DMSO-*d*$_6$) δ** 165.9 (13), 141.1 (19), 134.3 (16), 128.1 (25), 126.1 (18), 123.4 (15), 121.8 (17), 109.9 (20), 68.7 (21), 55.0 (11), 52.2 (24), 36.0 (28), 28.8 (23), 24.7 (22), 23.0 (29), 20.7 (30), 16.0 (31); **LR-ESI-MS**: $C_{24}H_{29}N_6O$ $[M+H]^+$ *m/z* found 417.54, cald 417.24; **HR-ESI-MS**: $C_{24}H_{29}N_6O$ $[M+H]^+$ *m/z* found 417.2378, cald 417.2403.

2-(piperidin-1-ylmethyl)-1H-benzo[d]imidazole **177**

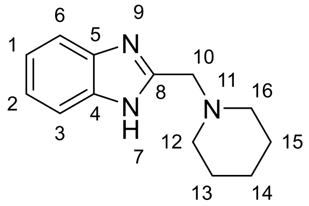

A solution of 2-(chloromethyl)-1H-benzo[d]imidazole (0.2 g, 1.2 mmol, 1 eq) in anhydrous MeCN (6 mL, 0.2 M) was stirred at room temperature as a brown suspension before piperidine (102 mg, 1.2 mmol, 1 eq) was added dropwise. The suspension was then heated conventionally at 100°C overnight. Upon reaction completion the solution was cooled to room temperature before being concentrated to a brown oil. The oil was loaded onto a KP-Sil SNAP 25 g column and eluted with CH/EA (100:0 to 0:100) to give the product as an off-white solid **177** (0.149 g, 0.69 mmol, 58%).

**Mpt:** 204.1-206.1 °C; **$v_{max}$ (cm$^{-1}$)** 2935, 2802, 1455, 1418, 1335, 1270, 1108, 744, 487; **$^{1}$H NMR (400 MHz, DMSO-*d*$_6$) δ** 12.23 (s, 1H, 7), 7.48 (d, *J* = 6.6 Hz, 2H, 2, 6), 7.26 – 6.92 (m, 2H, 1, 3), 3.66 (s, 2H, 10), 2.40 (t, *J* = 5.4 Hz, 4H, 12, 16), 1.52 (p, *J* = 5.6 Hz, 4H, 13, 15), 1.39 (td, *J* = 3.4, 6.8 Hz, 2H, 14); **$^{13}$C NMR (101 MHz, DMSO-*d*$_6$) δ** 152.0 (3, 6), 121.3 (1, 2), 56.6 (10), 54.1 (12, 16), 25.4 (13, 15), 23.7 (14); **LR-ESI-MS**: $C_{13}H_{18}N_3$ $[M+H]^+$ *m/z* found 216.21, cald 216.15; **HR-ESI-MS**: $C_{13}H_{17}N_3Na$ $[M+Na]^+$ *m/z* found 238.1338, cald 238.13202.

2-((2-methylpiperidin-1-yl)methyl)-1H-benzo[d]imidazole **178**

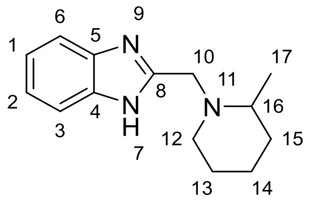


A solution of 2-(chloromethyl)-1H-benzo[d]imidazole (0.2 g, 1.2 mmol, 1 eq) in anhydrous MeCN (6 mL, 0.2 M) was stirred at room temperature as a brown suspension before 2-methylpiperidine (119 mg, 1.2 mmol, 1 eq) was added dropwise. The suspension was then heated conventionally at 100°C overnight. Upon reaction completion the solution was cooled to room temperature before being concentrated to a brown oil. The oil was loaded onto a KP-Sil SNAP 25 g column and eluted with CH/EA (100:0 to 0:100) to give the product as an off-white solid **178** (0.081 g, 0.355 mmol, 30%).

**Mpt:** 204.6-206.6 °C; **$v_{max}$ ($cm^{-1}$)** 2923, 2851, 1453, 1354, 1375, 1271, 1224, 742; **$^1$H NMR (400 MHz, DMSO-*$d_6$*) δ** 12.1 (s, 1H, 7), 7.5 (s, 2H, 1, 2), 7.2 – 7.1 (m, 2H, 3, 6), 4.0 (d, *J* = 14.6 Hz, 1H, 10''), 3.6 (d, *J* = 14.5 Hz, 1H, 10'), 2.7 (dt, *J* = 4.0, 11.7 Hz, 1H, 16), 2.4 – 2.3 (m, 1H, 12''), 2.2 – 2.1 (m, 1H, 12'), 1.7 – 1.6 (m, 2H, 15'), 1.6 – 1.4 (m, 2H, 13''), 1.2 (tt, *J* = 5.7, 12.3 Hz, 2H, 14'), 1.1 (d, *J* = 6.1 Hz, 3H, 17); **$^{13}$C NMR (101 MHz, DMSO-*$d_6$*) δ** 152.6 (3, 6), 122.6 – 118.5 (m, 1, 2), 55.7 , 10, 52.6 , 16, 51.8 , 12, 34.1 , 15, 25.6 , 13, 23.5 , 14, 19.0 , 17. **LR-ESI-MS:** $C_{14}H_{20}N_3$ $[M+H]^+$ *m/z* found 230.24, cald 230.17; **HR-ESI-MS**: $C_{14}H_{20}N_3$ $[M+H]^+$ *m/z* found 230.1670, cald 230.1657.

2-((3-methylpiperidin-1-yl)methyl)-1H-benzo[d]imidazole **179**

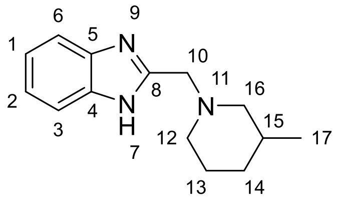


A solution of 2-(chloromethyl)-1H-benzo[d]imidazole (0.2 g, 1.2 mmol, 1 eq) in anhydrous MeCN (6 mL, 0.2 M) was stirred at room temperature as a brown suspension before 3-methylpiperidine (119 mg, 1.2 mmol, 1 eq) was added dropwise. The suspension was then heated conventionally at 100°C overnight. Upon reaction completion the solution was cooled to room temperature before being concentrated to a brown oil. The oil was loaded onto a KP-Sil SNAP 25 g column and eluted with CH/EA (100:0 to 0:100) to give the product as a yellow solid **179** (0.046 g, 0.199 mmol, 17%).

**Mpt:** 204.7-206.7 °C; **$v_{max}$ ($cm^{-1}$)** 2924, 1453, 1421, 1339, 1271, 1043, 1020, 999; **$^1$H NMR (400 MHz, DMSO-*$d_6$*) δ** 12.2 (s, 1H, 7), 7.5 (d, *J* = 24.9 Hz, 2H, 3, 6), 7.1 (dd, *J* = 2.9, 6.3 Hz, 2H, 1, 2), 3.8 – 3.5 (m, 2H, 10), 2.8 (td, *J* = 4.6, 10.3, 10.9 Hz, 2H, 12'', 16''), 2.0 (td, *J* = 3.0, 11.3 Hz, 1H, 16'), 1.8 – 1.4 (m, 5H, 12', 13, 14'', 15), 1.0 – 0.7 (m, 4H, 14', 17); **$^{13}$C NMR (101 MHz, DMSO-*$d_6$*) δ** 152.0 (3, 6), 120.8 (1), 118.4 (2), 61.5 (16), 56.3 (10), 53.6 (12), 32.4 (14), 30.6 (15), 25.0 (13), 19.5 (17); **LR-ESI-MS:** $C_{14}H_{20}N_3$ $[M+H]^+$ *m/z* found 230.23, cald 230.17; **HR-ESI-MS**: $C_{14}H_{20}N_3$ $[M+H]^+$ *m/z* found 230.1669, cald 230.1657.

2-((4-methylpiperidin-1-yl)methyl)-1H-benzo[d]imidazole **180**

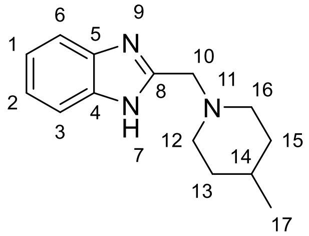


A solution of 2-(chloromethyl)-1H-benzo[d]imidazole (0.2 g, 1.2 mmol, 1 eq) in anhydrous MeCN (6 mL, 0.2 M) was stirred at room temperature as a brown suspension before 4-methylpiperidine (119 mg, 1.2 mmol, 1 eq) was added dropwise. The suspension was then heated conventionally at 100°C overnight. Upon reaction completion the solution was cooled to room temperature before being concentrated to a brown oil. The oil was loaded onto a KP-Sil SNAP 25 g column and eluted with CH/EA (100:0 to 0:100) to give the product as a white solid **180** (0.025 g, 0.11 mmol, 9%).

**Mpt:** 192.9-194.9 °C; **$v_{max}$ (cm$^{-1}$)** 2921, 2799, 1329, 1316, 1252, 739; **$^1$H NMR (400 MHz, DMSO-*d*$_6$) δ** 12.2 (s, 1H, 7), 7.5 (d, *J* = 36.3 Hz, 2H, 3, 6), 7.1 (q, *J* = 4.4 Hz, 2H, 1, 2), 3.7 (s, 2H, 10), 2.8 (dt, *J* = 3.2, 11.9 Hz, 2H, 12'', 16''), 2.0 (td, *J* = 2.5, 11.6 Hz, 2H, 12', 16'), 1.7 – 1.5 (m, 2H, 13'', 15''), 1.3 (ddd, *J* = 3.7, 6.8, 11.0 Hz, 1H, 14), 1.3 – 1.1 (m, 2H, 13', 15'), 0.9 (d, *J* = 6.4 Hz, 3H, 17); **$^{13}$C NMR (101 MHz, DMSO-*d*$_6$) δ** 152.1 (5, 8), 121.1 – 120.5 (m, 1, 2), 118.3 (6), 111.1 (3), 56.2 (10), 53.5 (12, 16), 33.8 (13, 15), 30.0 (14), 21.8 (17); **LR-ESI-MS:** $C_{14}H_{20}N_3$ [M+H]$^+$ *m/z* found 230.24, cald 230.17; **HR-ESI-MS**: $C_{14}H_{20}N_3$ [M+H]$^+$ *m/z* found 230.1671, cald 230.1657.

*N*-(1H-benzo[d]imidazol-5-yl)benzamide **181**

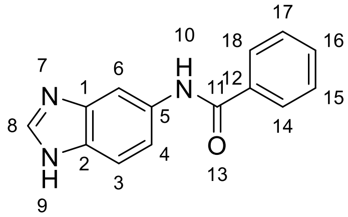


Initially a solution of 1H-benzo[d]imidazol-5-amine (0.2 g, 1.5 mmol, 1 eq), *N*,*N*-dimethylpyridin-4-amine (18 mg, 0.15 mmol, 0.1 eq) and DIPEA (314 µL, 1.8 mmol, 1.2 eq) in DCM (7.5 mL, 0.2 M) was cooled to 0°C before benzoyl chloride (209 µL, 1.8 mmol, 1.2 eq) was added dropwise. The solution was allowed to stir at ambient temperature before being concentrated onto silica gel and eluted through a KP-Sil SNAP 25 g column DCM/DCM (20% MeOH) (1:0 to 0:1) to give the product **181** as a white solid (0.013 g, 0.06 mmol, 4%).

**Mpt:** 288.5-290.5 °C; **$v_{max}$ (cm$^{-1}$)** 2981, 2824, 1637, 1599, 1578, 1476, 1293, 1243, 807, 693, 409; **$^1$H NMR (400 MHz, DMSO-*d*$_6$) δ** 12.4 (s, 1H, 9), 10.2 (s, 1H, 10), 8.3 – 8.1 (m, 1H, 8), 8.1 – 7.9 (m, 3H, 3, 4, 6), 7.7 – 7.3 (m, 5H, 14, 15, 16, 17, 18); **$^{13}$C NMR (101 MHz, DMSO-*d*$_6$) δ** 165.4 (11), 135.2 (3), 131.3 (4, 6), 128.3 (14, 18), 127.6 (15, 17); **LR-ESI-MS**: $C_{14}H_{12}N_3O$ [M+H]$^+$ *m/z* found 238.17, cald 238.09; **HR-ESI-MS**: $C_{14}H_{12}N_3O$ [M+H]$^+$ *m/z* found 238.0984, cald 238.0980.

*N*-(2-(((trans)-2,5-dimethylpyrrolidin-1-yl)methyl)-1*H*-benzo[*d*]imidazol-5-yl)-1-methyl-1*H*-indazole-5-carboxamide **182**

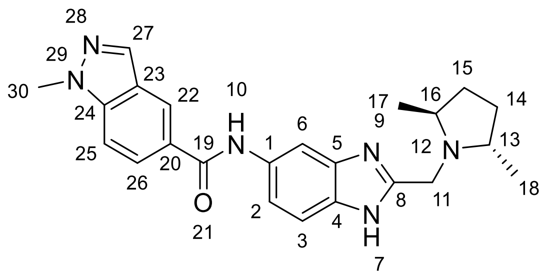

Synthesised according to general procedure **B** to give **182** (0.037 g, 0.093 mmol, 60 %) as an white solid.

**Mpt:** 149.3-151.3 °C; $\mathbf{v_{max}}$ **(cm$^{-1}$)** 2958, 1599, 1484, 1415, 1310, 1192, 840, 556; **$^{1}$H NMR (400 MHz, DMSO) δ** 12.09 (s, 1H, 8), 10.25 (s, 1H, 7), 8.48 (d, *J*=1.6, 1H, 26), 8.25 (d, *J*=0.9, 1H, 29), 8.10 (s, 1H, 6), 8.02 (dd, *J*=8.8, 1.6, 1H, 22), 7.76 (d, *J*=8.8, 1H, 23), 7.47 (s, 2H, 2, 3), 4.10 (s, 3H, 30), 3.92 (s, 2H, 11''), 2.92 – 2.57 (m, 2H, 13, 16), 1.82 (s, 2H, 14'', 15''), 1.48 – 1.25 (m, 2H, 14', 15'), 1.14 – 0.88 (m, 6H, 17, 18); **$^{13}$C NMR (101 MHz, DMSO) δ** 165.4 (19), 140.6 (29), 133.9 (22), 127.6 (6), 125.6 (26), 122.9 (23), 121.3 (2), 109.5 (3), 64.9 (11), 59.7 (13, 16), 47.9 (30), 35.6 (17, 18), 19.8 (15), 15.2 (14); **LR-ESI-MS**: $C_{23}H_{27}N_6O$ $[M+H]^+$ *m/z* found 403.49, cald 403.23; **HR-ESI-MS**: $C_{23}H_{27}N_6O$ $[M+H]^+$ *m/z* found 403.2246, cald 403.2246.

3-fluoro-*N*-(2-(piperidin-1-ylmethyl)-1H-benzo[d]imidazol-5-yl)benzamide **183**

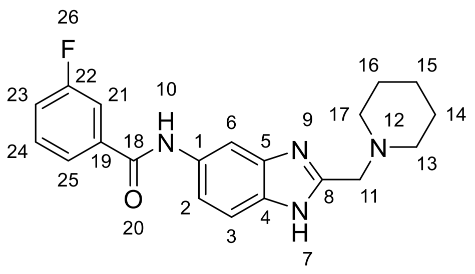

Synthesised according to general procedure **C** to give **183** (0.057 g, 0.163 mmol, 75 %) as an off white solid.

**Mpt:** 108.8-110.8 °C; $\mathbf{v_{max}}$ **(cm$^{-1}$)** 3067, 2934, 1647, 1585, 1536, 1435, 1381, 1218, 768, 744, 665; **$^{19}$F NMR (376 MHz, DMSO-*d*$_6$) δ** -112.7 (td, *J* = 5.9, 9.3 Hz); **$^{1}$H NMR (400 MHz, DMSO-*d*$_6$) δ** 12.4 (s, 0H), 10.3 (s, 1H, 10), 8.0 (s, 1H, 7), 7.9 – 7.7 (m, 2H, 21, 23), 7.7 – 7.4 (m, 5H, 2, 3, 6, 24, 25), 3.7 (s, 2H, 11), 2.4 (t, *J* = 5.4 Hz, 4H, 13, 17), 1.5 (p, *J* = 5.5 Hz, 4H, 14, 16), 1.4 (q, *J* = 6.0 Hz, 2H, 15); **$^{13}$C NMR (101 MHz, DMSO-*d*$_6$) δ** 163.8 (18), 162.0 (d, J = 244.0 Hz, 22), 152.4 (8), 137.5 (d, J = 6.5 Hz, 19), 130.5 (d, J = 8.2 Hz, 21), 130.4 (d, J = 8.1 Hz, 24), 125.3 (d, J = 2.8 Hz, 23), 123.8 (d, J = 2.8 Hz, 25), 118.4 (5), 118.2 (4, 115.7 (3), 115.5 (1), 114.6 (6), 114.3 (2), 56.6 (11), 54.1 (13, 17), 25.4 (14, 16), 23.7 (15); **LR-ESI-MS**: $C_{20}H_{22}FN_4O$ $[M+H]^+$ *m/z* found 353.36, cald 353.18; **HR-ESI-MS**: $C_{20}H_{22}FN_4O$ $[M+H]^+$ *m/z* found 353.1765, cald 353.1778.

2-(((trans)-2,5-dimethylpyrrolidin-1-yl)methyl)-5-nitro-1*H*-benzo[*d*]imidazole **184**

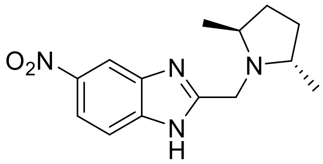


Initially a suspension of 2-(chloromethyl)-5-nitro-1H-benzo[d]imidazole **3** (1.3 g, 6.14 mmol, 1 eq) and $Na_2CO_3$ (1.563 g, 14.75 mmol, 2.4 eq) in anhydrous MeCN (8 mL, 0.8 M) had 2,5-dimethylpyrrolidine, HCl (1 g, 7.37 mmol, 1.2 eq) added at room temperature. The reaction was allowed to stir at room temperature overnight. Upon reaction completion the suspension was filtered through a sintered frit and washed with acetone. The filtrate was concentrated to a residue which was immediately submitted to the next step without further purification as **184**.

2-(((trans)-2,5-dimethylpyrrolidin-1-yl)methyl)-1*H*-benzo[*d*]imidazol-5-amine **185**

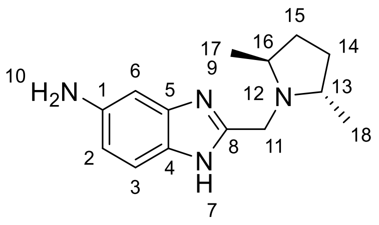

Synthesised according to general procedure **A** to give **185** (0.089 g, 0.36 mmol, 32 %) as a white solid.

**Mpt:** 171.31-173.3 °C; **$\nu_{max}$ ($cm^{-1}$)** 3356, 2955, 1631, 1422, 1221, 1013, 800, 434; **$^1$H NMR (400 MHz, $CDCl_3$) δ** 7.46 – 7.22 (m, 1H, 6), 6.76 (s, 1H, 3), 6.59 (dd, *J* = 8.5, 2.2 Hz, 1H, 2), 3.87 (s, 2H, 11), 2.78 – 2.49 (m, 2H, 13, 16), 1.94 – 1.70 (m, 2H, 14', 15'), 1.48 – 1.23 (m, 2H, 14'', 15''), 0.97 (dd, *J* = 6.2, 1.9 Hz, 6H, 17, 18). **$^{13}$C NMR (101 MHz, $CDCl_3$) δ** 153.7 (8), 142.1 (6), 112.0 (2, 3), 62.10 (11), 50.6 (13, 16), 31.3 (14, 15), 20.6 (17, 18); **LR-ESI-MS**: $C_{14}H_{21}N_4$ $[M+H]^+$ *m/z* found 245.47, cald 245.17; **HR-ESI-MS**: $C_{14}H_{20}N_4Na$ $[M+Na]^+$ *m/z* found 267.1574, cald 267.1586.

methyl 1-cyclopropyl-1*H*-indazole-5-carboxylate **186**

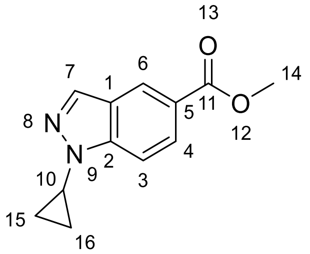

Initially methyl 1H-indazole-5-carboxylate (0.828 g, 4.70 mmol, 1 eq), cyclopropylboronic acid (0.807 g, 9.40 mmol, 2 eq), $Cu(OAc)_2$ (0.854 g, 4.70 mmol, 1 eq) and 2,2'-bipyridine (0.734 g, 4.70 mmol, 1 eq) were dissolved in 1, 2-Dichloroethane (42 mL, 0.11 M) and heated to 70°C for 16 h under a blanket of air. Upon reaction completion the mixture was filtered through a bed of celite and the filtrate was concentrated to a residue which was purified via Biotage LPLC eluting with CH:EA (1:0 to 1:1) to give the product **186** as a yellow solid (0.641 g, 2.96 mmol, 63%).

**Mpt:** 76.0-78.0 °C; **$v_{max}$ ($cm^{-1}$)** 1714, 1617, 1438, 1311, 1251, 1086, 761, 452; **$^1$H NMR (400 MHz, DMSO-*$d^6$*) δ** 8.44 (dd, *J*=1.6, 0.8, 1H, 7), 8.18 (d, *J*=0.9, 1H, 6), 7.95 (dd, *J*=8.8, 1.6, 1H, 4), 7.74 (dt, *J*=8.9, 0.9, 1H, 3), 3.86 (s, 3H, 14), 3.79 (tt, *J*=6.7, 4.0, 1H, 10), 1.20 – 0.95 (m, 4H, 15, 16); **$^{13}$C NMR (101 MHz, DMSO) δ** 166.5 (11), 141.9 (7), 134.5 (4), 126.4 (6), 124.2 (5), 123.6 (2), 122.4 (1), 109.9 (3), 52.0 (14), 29.4 (10), 6.2 (15, 16); **LR-ESI-MS**: $C_{12}H_{13}N_2O_2$ $[M+H]^+$ *m/z* found 217.20, cald 217.10; **HR-ESI-MS**: $C_{12}H_{13}N_2O_2$ $[M+H]^+$ *m/z* found 217.0967, cald 217.0977.

1-cyclopropyl-1*H*-indazole-5-carboxylic acid **187**

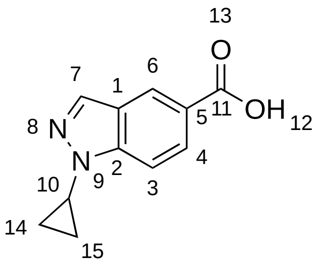


Initially compound **186** (0.573 g, 2.65 mmol, 1 eq) was dissolved in MeOH (4 mL, 0.6 M) and had crushed NaOH pellets (0.53 g, 13.25 mmol, 5 eq) added before being stirred at 65°C for 2 h. Upon reaction completion the mixture was concentrated to dryness and then dissolved in water. The aqueous mixture was carefully acidified with 2 *N* HCl (until pH 4-5) until the product began to precipitate, which was then filtered off, washed with water and dried in a vacuum oven to give the product **187** as a pale yellow solid (0.366 g, 1.81 mmol, 68%).

**Mpt:** 174.6-176.6 °C; **$v_{max}$ ($cm^{-1}$)** 2783, 1701, 1618, 1243, 1175, 765, 687, 603, 481; **$^1$H NMR (400 MHz, DMSO-*$d^6$*) δ** 12.84 (s, 1H, 12), 8.57 – 8.39 (m, 1H, 7), 8.18 (s, 1H, 6), 7.98 (dd, *J*=8.8, 1.6, 1H, 4), 7.73 (d, *J*=8.8, 1H, 3), 3.92 – 3.69 (m, 1H, 10), 1.13 (tt, *J*=6.3, 2.4, 4H, 14, 15); **$^{13}$C NMR (101 MHz, DMSO) δ** 167.6 (11), 141.9 (7), 134.4 (4), 126.8 (6), 124.1 (1), 123.6 (2), 123.6 (5), 109.7 (3), 29.4 (10), 6.2 (14, 15); **LR-ESI-MS**: $C_{11}H_{11}N_2O_2$ $[M+H]^+$ *m/z* found 203.37, cald 203.08; **HR-ESI-MS**: $C_{11}H_{11}N_2O_2$ $[M+H]^+$ *m/z* found 203.0808, cald 203.0821.

## II.II AlphaScreen

### Selectivity

| YEATS2 $IC_{50}$ (µM) | YEATS4 $IC_{50}$ (µM) | BRD4 (1) $IC_{50}$ (µM) | CECR2 (2) $IC_{50}$ (µM) | FALZ $IC_{50}$ (µM) | CBP $IC_{50}$ (µM) | TAF1 $IC_{50}$ (µM) |
|---|---|---|---|---|---|---|
| >10 | >10 | >10 | >10 | >10 | >10 | >10 |

**Supplementary Table 1.** AlphaScreen selectivity screen of compound **92** over other acyllysine reading domains.

## II.III Isothermal Titration Calorimetry (ITC)

### Methods

Experiments were carried out on a Nano-ITC Standard Volume Instrument (TA Instruments). All experiments were carried out at 25 °C in 20 mM HEPES pH 7.5, 150 mM NaCl, 0.5 mM TCEP and 5% glycerol. Protein solutions were buffer exchanged by gel filtration. The titrations were conducted using an initial injection of 2 µL followed by 32 injections of 8 µL. Background dilution heat was subtracted from each experiment. Thermodynamic parameters were calculated using $\Delta G = \Delta H - T\,\Delta S = -RT\ln K_B$ , where $K_D = 1/K_B$. $\Delta G$, $\Delta H$ and $\Delta S$ are changes in free energy, enthalpy and entropy respectively. Independent single site binding models were employed in data analysis.

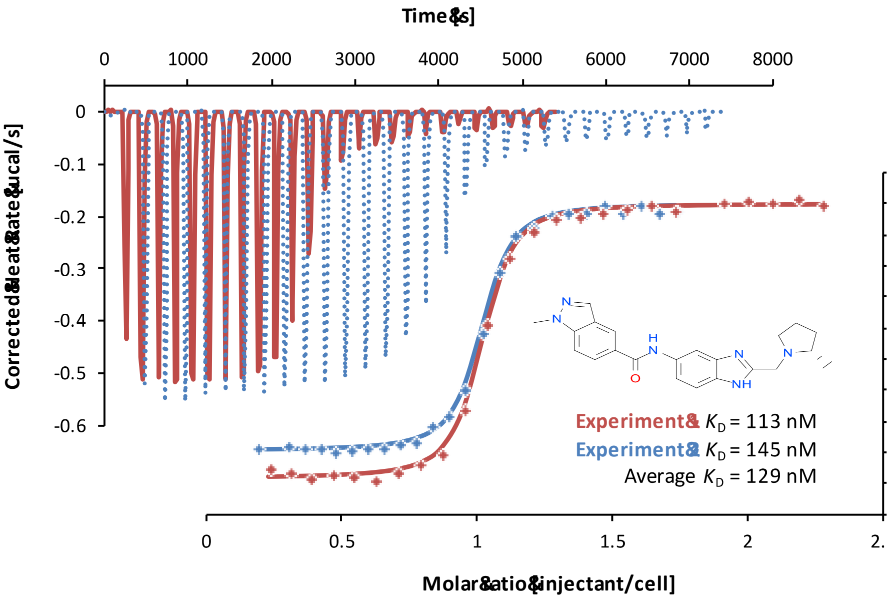


**Supplementary Figure 1.** ITC traces of compound **92** and MLLT1 YD

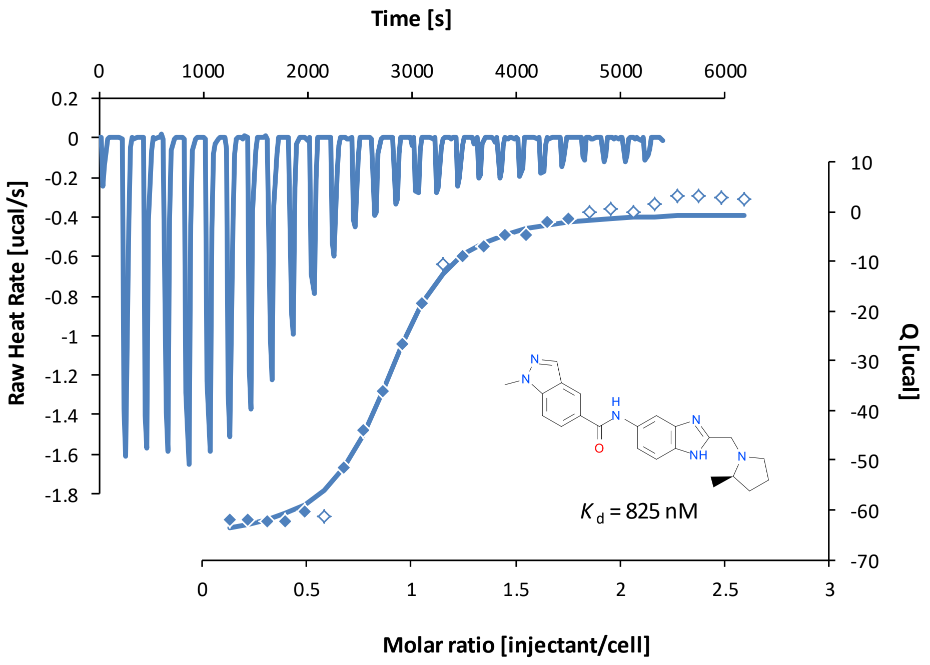


**Supplementary Figure 2.** ITC trace of compound **91** and MLLT3 YD

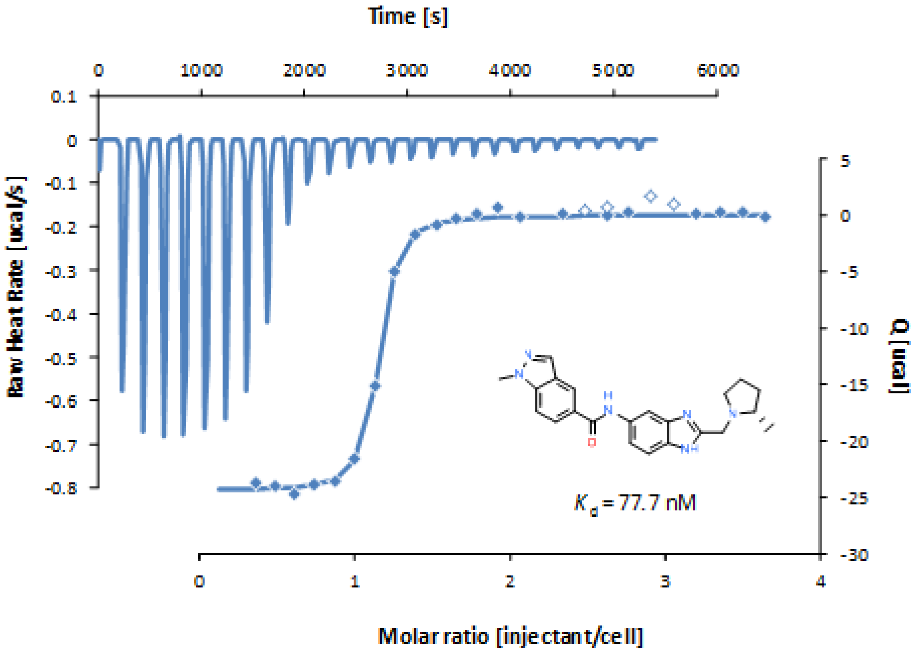


**Supplementary Figure 3.** ITC trace of compound **92** and MLLT3 YD

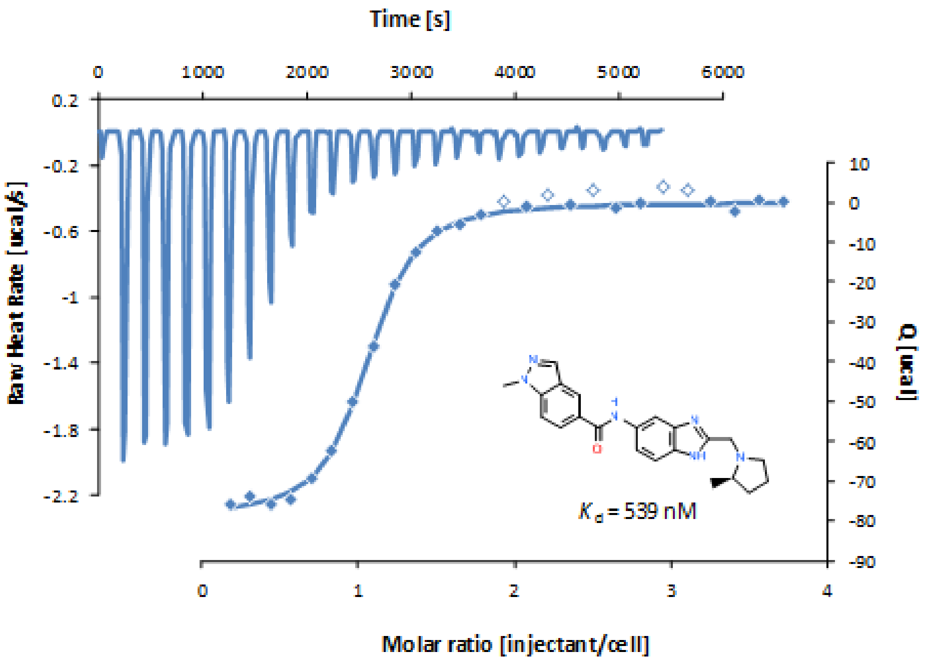


**Supplementary Figure 4.** ITC trace of compound **91** and MLLT3 YD

## II.IV Protein Expression and Purification

Human bromodomains were cloned, expressed and purified as previously described.[1] The cloning, expression and purification of human YEATS domains are described as follows;

Sequences for the wild type YEATS domains of MLLT1, MLLT3, GAS41 and YEATS2 were cloned into expression vectors (Table S1). *E. coli* Rosetta cells were grown in TB medium at 37°C. Overexpression was induced after five hours by addition of IPTG to a final concentration of 0.4 mM and cells were incubated at 17°C overnight. Cells were harvested by centrifugation and the cell pellet was resuspended in cold binding buffer (50 mM Tris at pH 7.5, 500 mM NaCl, 20 mM imidazole, 2 mM DTT) on a magnetic stirring plate at 4°C. The suspension was lysed using an Avestin EmulsiFlex-C5 homogeniser (1 pass without pressure, four passes with pressure between 1000-1500 bar). The lysate was clarified via centrifugation at 16,000 rpm at 4°C for 1 hour (JLA-16 rotor in Beckmann Coulter Avanti J-26S XP centrifuge). The supernatant was loaded onto a standard NiNTA column (HisTrap FF, 5 ml, GE Healthcare Lifesciences) on an ÄKTAxpress system (GE). After washing with binding buffer, the target protein was eluted at 300 mM imidazole. The eluted peak fractions were pooled and concentrated to 5 ml (Amicon concentrators, 10 kDa molecular weight cut off (MWCO)) and then separated via size exclusion chromatography (buffered with 20 mM Tris at pH 7.5, 500 mM NaCl, 2 mM DTT) using a GE Superdex 75 column on an ÄKTAxpress system. Protein concentration was quantified via extinction at 280 nM (NanoDrop ND-1000 Spectrophotometer) and protein identity was verified via SDS-PAGE and LC/MS. Pooled fractions were then again concentrated to at least 2 mg/ml and stored at -80°C.

## II.V NanoLuciferase Bioluminescent Resonance Energy Transfer (NanoBRET) Assay[2]

### Methods

HEK293 cells (8 x $10^5$) were plated in each well of a 6-well plate after 6 h cells were co-transfected with Histone H3.3-HaloTag (NM_002107) and a NanoLuciferase fusion of MLLT1 or MLLT3 (WT sequences from Promega HaloTag® human ORF in pFN21A) at a 1:500 and 1:10 (NanoLuc® to HaloTag®) ratio respectively with FuGENE HD transfection regent. Sixteen hours post-transfection, cells were collected and exchanged into media containing phenol red-free DMEM and 4% FBS in the absence (control sample) or the presence (experimental sample) of 100 nM NanoBRET 618 fluorescent ligand (Promega). Cell density was adjusted to 2 x $10^5$ cells/mL and then seeded 100 µL/well in a white 96-well plate (Corning Costar #3917). Compounds were then added directly to media (in the presence of SAHA 2.5 µM) at final concentrations 0.01-30 µM or an equivalent amount of DMSO as a vehicle control, and the plates were incubated for 24 h at 37 $^oC$ in the presence of 5% $CO_2$.

NanoBRET Nano-Glo substrate (Promega) was added to both control and experimental samples at a final concentration of 10 µM. Readings were performed within 5 minutes using a ClarioSTAR (BMG labtech) equipped with LP 480 and 610 nm filters. A corrected BRET ratio was calculated and is defined as the ratio of the emission at 610 nm/640 nm for experimental samples minus the emission at 610 nm/640 nm for control samples (not incubated with NanoBRET fluorescent ligand). BRET ratios are expressed as milliBRET units (mBU), where 1 mBU corresponds to the corrected BRET ratio multiplied by 1000.

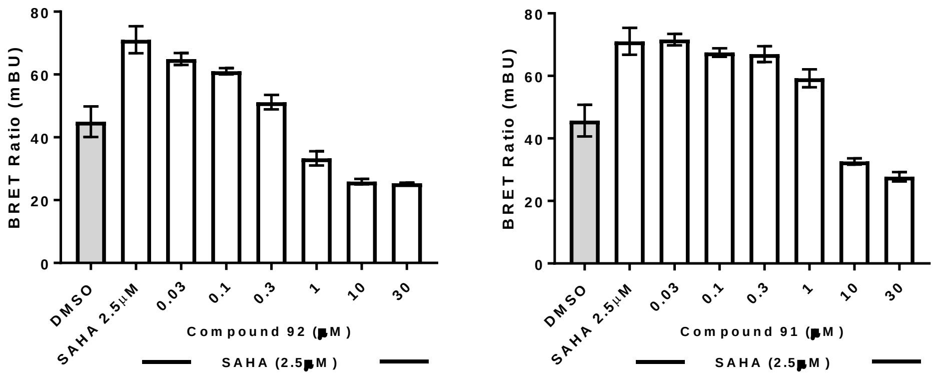


**Supplementary Figure 5.** Shows a representative NanoBRET™ assay demonstrating the dose response of compound **92** and **91** (24 h treatment) in full length MLLT3 N-terminal NanoLuc, Histone 3.3 C-terminal HaloTag in HEK293 cells. Treatment with SAHA (2.5 µM) alone increases MLLT3-Histone 3.3 interaction (mBU) and the addition of compound **92** can reduce this interaction in a dose dependent manner. Mean±SD of BRET ratio (mBU), n=4 technical replicates.

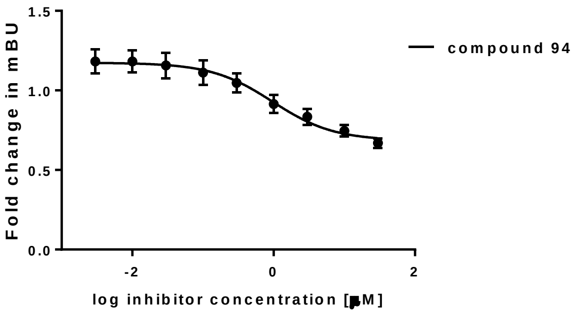


**Supplementary Figure 6.** NanoBRET dose response for compound **94** after 24h treatment using N-terminal-nanoLuc-MLLT3 and C-terminal HaloTag-H3.3 in HEK293 cells, in the presence of 2.5 μM SAHA. Graph represent n=3 biological replicates, with n=5 technical replicates. Mean±SEM (mBU – BRET units). Average $IC_{50}$ 0.967 ± 0.22 μM

## II.VI Fluorescence recovery after photobleaching (FRAP) Assay[3–6]

### Methods

### Cloning for FRAP

The full-length sequence for wild-type human MLLT1, Mutant MLLT1 (78Y to A) and wild-type MLLT3 were amplified from a DNA plasmid template using Phusion High Fidelity Polymerase (New England Biolabs) and primers including appropriate attB sequences. PCR products flanked by attB sequences were cloned with Gateway BP Clonase II enzyme mix (Life Technologies) into pDONR221 to create entry clones. Entry clone plasmids containing WT MLLT1, MLLT3 or mutant MLLT1 were recombined into Vivid Colours pcDNA6.2/C-EmGFP-DEST vectors (Life Technologies) using Gateway LR Clonase II enzyme mix (Life Technologies) to create chimeric GFP expression clones.

### FRAP Assay

FRAP experiments were performed using a protocol modified from a previous study [3]. Briefly, U2OS cells were seeded ($10^5$ cells) in a glassed bottomed 35 mm culture dish (FluoroDish, WPI) and cultured at 37ºC in a humidified 5% $CO_2$ atmosphere in McCoy's *5A* (Modified) Medium *(*Thermofisher) containing 10% FBS and 1% GlutaMAX (Gibco). 24 hours post seeding, cells were transfected (at 1:4, DNA:FuGENE HD transfection regent, Promega) with wild-type MLLT1, MLLT3 or mutant MLLT1 pcDNA6.2/C-EmGFP-DEST (2.5µM SAHA was added to the transfected cells, except for the wild-type (WT) condition). 1µM inhibitor was added 1 hour before imaging, which was carried out 24 hours after SAHA addition. Imaging was performed using a Zeiss LSM 710 laser-scanning confocal microscope (40x /1.3 N.A., Oil-immersion objective lens) attached to a Zeiss Axio Observer Z1 motorized inverted microscope. All experiments were performed under 37ºC and 5% $CO_2$. Argon ion laser (488 nm, 40mW, set at 50% power, with 0.3% of power for FRAP acquisition at pinhole diameter 1.39 Airy units) was used to excite GFP and perform FRAP. The Photomultiplier tube detector was set to detect fluorescence between 500 and 570 nm and cells within the gain range of 650 – 850 were chosen for bleaching. Cells expressing very high or very low GFP were excluded from selection for bleaching. Accordingly, cell with approximately the same nucleus size was chosen. A circular region of photobleach area was fixed at 31 $\mu m^2$ for all experiments. A time lapse series was taken to record GFP intensity pre (5 scans) and post (175 scans) bleaching. Images were collect using the Carl Zeiss Zen Black software at 170 ms per 512 x 512 frame (35.42 µm x 35.42 µm). Intensity values were collected from 3 regions: 1) bleach area, 2) whole nucleus and 3) a region outside the cell to determine the background signal. Data were exported and analysed using easyFRAP, a stand-alone, single-screen open source MatLab GUI [4] implemented with the double normalization method to normalize recovery curves [5] and with double term curve fitting. Fitted curves with $R^2$ values <0.95 were removed from further analysis. Results are from 3 independent experiments, with each set containing at least 10 cells per condition. One-way ANOVA with Tukey–Kramer correction for multiple comparisons was used to detect significant differences ($P < 0.05$) between conditions.

## II.VII Thermal Shift Assay[6]

### Methods

Thermal melting experiments were carried out using an Mx3005p Real Time PCR machine (Stratagene). Proteins were buffered in 10 mM HEPES pH 7.5, 500 mM NaCl and assayed in a 96-well plate at a final concentration of 2 μM in 20 μL volume. Compounds were added at a final concentration of 50 μM. SYPRO Orange (Molecular Probes) was added as a fluorescence probe at a dilution of 1:1000. Excitation and emission filters for the SYPRO-Orange dye were set to 465 nm and 590 nm, respectively. The temperature was raised with a step of 3 °C per minute from 25 °C to 96 °C and fluorescence readings were taken at each interval. The unfolding curves were fitted using the Boltzmann equation to determine the middle point of the transition.

| | Compound **1** * | SGC-iMLLT (**92**) * | **Bromosporine †** |
|---|---|---|---|
| ASH1L | 0.32 | -0.305 | 0.51 |
| ATAD2A | 0.54 | 0.56 | 0.67 |
| BAZ1A | 1.085 | -0.26 | -0.77 |
| BAZ1B | 1.03 | -0.19 | -0.23 |
| BAZ2A | 0.81 | 0.25 | 1.125 |
| BAZ2B | 0.57 | 0.155 | 1.385 |
| BRD1 | 0.2 | 0.215 | 2.055 |
| BRD2(1) | 1.345 | -0.28 | 4.46 |
| BRD2(2) | 0.55 | 0.19 | 5.8 |
| BRD3(1) | 1.82 | -0.42 | 5.275 |
| BRD3(2) | 0.675 | -0.1 | 6.56 |
| BRD4(1) | 1.095 | -0.035 | 6.61 |
| BRD4(2) | 0.715 | 0.1 | 6.575 |
| BRD7 | 0.57 | -0.08 | 8.48 |
| BRD9 | -0.915 | -1.445 | 7.145 |
| BRDT(1) | 1.83 | 0.21 | 4.68 |
| BRDT(2) | -0.015 | -0.555 | 6.035 |
| BRPF1A | -1.045 | -1.305 | 0.145 |
| BRPF1B | 0.275 | -0.435 | 4.23 |
| BRPF3 | 1.015 | -0.245 | 1 |
| BRD8 | -1.225 | -0.715 | 0.74 |
| CECR2 | 1.28 | 1.205 | 11.42 |
| CREBBP | 0.675 | -0.075 | 3.31 |
| EP300 | 0.285 | 0.145 | 3.66 |
| FALZ | 0.235 | -0.315 | 1.5 |
| GCN5 | 0.145 | -0.225 | 0.925 |
| KIAA1240 | 0.355 | 0.15 | 0.59 |
| LOC93349 | 0.035 | -1.05 | 2.385 |
| MLL | -0.18 | -1.31 | 0.75 |
| PB1(1) | -1.095 | -1.745 | 0.98 |
| PB1(2) | 0.105 | -0.17 | 0.105 |
| PB1(3) | 1.5 | 1.09 | 0.24 |
| PB1(4) | 0.22 | -0.215 | 1.035 |
| PB1(5) | -0.245 | -0.56 | 0.98 |
| PB1(6) | 0.17 | -0.365 | 0.82 |
| PCAF | 0.895 | 0.05 | 1.025 |
| PHIP(2) | -0.595 | -1.48 | -2.61 |
| SMARCA2A | 1.445 | 0.97 | 0.35 |
| SMARCA4 | 0.135 | 0.035 | 0.12 |
| SP140 | -0.15 | -0.915 | -1.045 |
| TAF1(1) | 0.005 | -0.15 | 0.805 |

| | | | |
|---|---|---|---|
| TAF1(2) | -0.22 | -0.21 | 5.03 |
| TAF1L(1) | 0.39 | -0.215 | 0.575 |
| TAF1L(2) | -0.065 | -0.48 | 4.83 |
| TRIM33B | 0.02 | -0.28 | 0.03 |
| TIF1a | -1.13 | -1.04 | 0.16 |
| TRIM28 | 0.02 | -0.225 | 0.265 |
| WDR9(2) | -0.18 | -0.775 | -1.225 |

**Supplementary Table 2.** Thermal stabilisation of compound **1**, **92** and Bromosporine with recombinant human bromodomain proteins. *50 µM compound concentration. †10 µM compound concentration.

## II.VIII Cellular Thermal Shift Assay[6]

### Methods

HEK293 and MV4;11 cells were cultured at 37 °C in a humidified 5% CO2 atmosphere in DMEM Medium containing 10% FBS, respectively. Cells were grown until approximately 80% confluency and treated with indicated concentrations of compound **92** or **91** (0.5% DMSO final concentration) for 30 minutes. Cells were then collected, washed with PBS, transferred in PCR tubes, and pelleted by centrifugation (300 *g*, 3 min, RT.) The PBS was removed, and the cell pellets heated at the indicated temperature for 3 minutes in a PCR machine (UNO96, VMR) and then placed on ice. Lysis buffer (50 mM Tris pH 7.5, 0.8% v/v NP-40, 5% v/v glycerol, 1.5 mM $MgCl_2$, 100 mM NaCl, 25 mM NaF, 1mM $Na_3VO_4$, 1mM PMSF, 1mM DTT, 10 µg/mL TLCK, 1 µg/mL Leupeptin, 1 µg/mL Aprotinin, 1 µg/mL soy bean trypsin, ≥250 units/mL Benzonase) was added and the cells lysed by 3 freeze-thaw cycles in liquid nitrogen. Aggregated proteins were removed by centrifugation (17000 *g*, 20 min, 4 °C) and the protein concentration of the retained soluble fraction determined. 4× sample loading buffer (Bio-Rad) containing 20% v/v DTT was added and samples were heated for 6 min at 90 °C prior to separation by SDS-PAGE. Proteins were transferred to nitrocellulose blotting membrane (Amersham, GE healthcare) and membranes were blocked with blocking buffer (2.5% (m/v) BLOT-QuickBlocker (Merck) in PBST (Phosphate-buffered saline with 0.05% (v/v) Tween 20) before probing with antibodies. Blots were imaged on an Odyssey CLx imager (LI-COR), with quantitative analysis performed using ImageQuant software. Antibodies: anti-MLLT1 (Cell Signalling Technology, 14893, 1:500 dilution); anti-GAPDH (Santa Cruz Technology, sc-365062, 1:1000 dilution); anti-rabbit alexa fluor 680 (LI-COR, A-21109, 1:10,000 dilution); anti-mouse alexa fluor 750 (LI-COR, A-21037, 1:10,000 dilution).

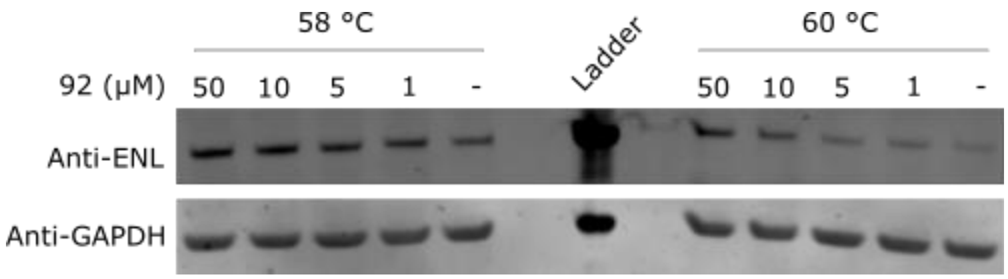


**Supplementary Figure 7.** Western blot showing dose dependent heat shock stabilisation of endogenous MLLT1 in HEK293 cells by compound **92**.

### II.IX *in vitro* Metabolism Studies

Metabolic stability studies were carried out exposing nominated compounds to samples of primary human hepatocytes. Compounds were analysed by LC-MS for loss of parent compound at 0, 10, 30, 60 and 120-minute time points and analysis of metabolites formed. The rate of metabolic degradation of parent compound was used to calculate, $t_{1/2}$ (summarised in Supplementary Figure 8).

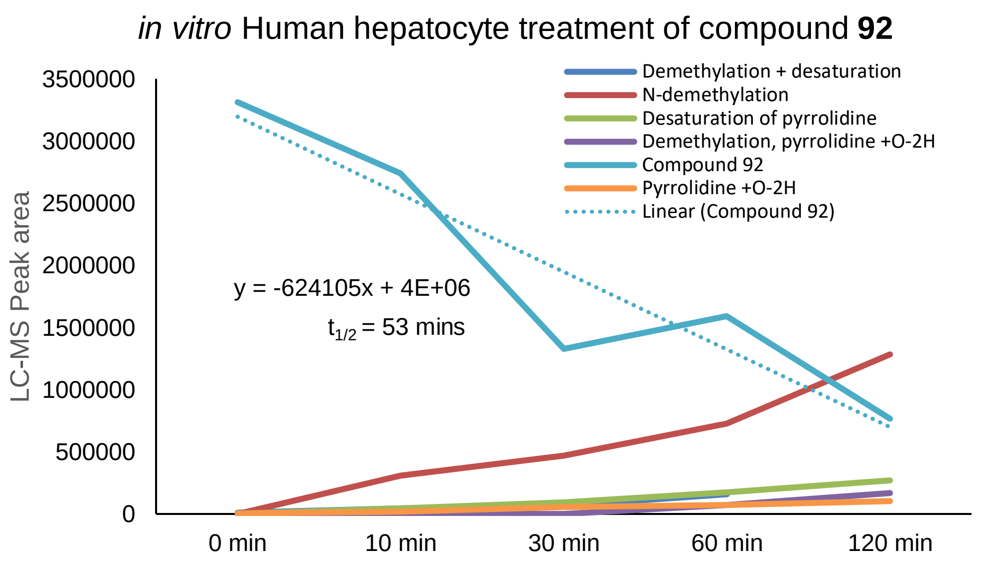


**Supplementary Figure 8.** In vitro metabolic stability of compound **92**.

## II.X Cell Proliferation Studies

| Developmental Therapeutics Program One Dose Mean Graph | **NSC:** D-803869 / 1 | **Conc:** 1.00E-5 Molar | **Test Date:** Feb 26, 2018 |
|---|---|---|---|
| | **Experiment ID:** 1802OS44 | | **Report Date:** Apr 02, 2018 |

| Panel/Cell Line | Growth Percent |
|---|---|
| Leukemia | |
| CCRF-CEM | 82.97 |
| HL-60(TB) | 86.69 |
| K-562 | 84.81 |
| MOLT-4 | 95.66 |
| RPMI-8226 | 89.78 |
| SR | 82.78 |
| Non-Small Cell Lung Cancer | |
| A549/ATCC | 98.11 |
| EKVX | 91.92 |
| HOP-62 | 101.93 |
| HOP-92 | 96.14 |
| NCI-H226 | 94.67 |
| NCI-H23 | 100.85 |
| NCI-H322M | 106.56 |
| NCI-H460 | 106.69 |
| NCI-H522 | 95.79 |
| Colon Cancer | |
| COLO 205 | 107.35 |
| HCC-2998 | 109.07 |
| HCT-116 | 93.85 |
| HCT-15 | 96.51 |
| HT29 | 81.68 |
| KM12 | 104.65 |
| SW-620 | 106.37 |
| CNS Cancer | |
| SF-268 | 102.31 |
| SF-295 | 96.96 |
| SF-539 | 102.10 |
| SNB-19 | 99.15 |
| SNB-75 | 101.45 |
| U251 | 102.93 |
| Melanoma | |
| LOX IMVI | 102.19 |
| MALME-3M | 90.80 |
| M14 | 101.61 |
| MDA-MB-435 | 99.52 |
| SK-MEL-2 | 111.66 |
| SK-MEL-28 | 108.78 |
| SK-MEL-5 | 87.64 |
| UACC-257 | 90.94 |
| UACC-62 | 93.69 |
| Ovarian Cancer | |
| IGROV1 | 112.48 |
| OVCAR-3 | 116.54 |
| OVCAR-4 | 101.53 |
| OVCAR-5 | 110.67 |
| OVCAR-8 | 109.96 |
| NCI/ADR-RES | 107.38 |
| SK-OV-3 | 89.92 |
| Renal Cancer | |
| 786-0 | 93.12 |
| A498 | 84.05 |
| ACHN | 106.55 |
| CAKI-1 | 103.34 |
| RXF 393 | 111.49 |
| SN12C | 98.85 |
| TK-10 | 110.19 |
| UO-31 | 96.37 |
| Prostate Cancer | |
| PC-3 | 97.54 |
| DU-145 | 108.23 |
| Breast Cancer | |
| MCF7 | 76.77 |
| MDA-MB-231/ATCC | 106.83 |
| HS 578T | 98.82 |
| BT-549 | 104.22 |
| T-47D | 90.41 |
| MDA-MB-468 | 110.70 |
| Mean | 99.21 |
| Delta | 22.44 |
| Range | 39.77 |

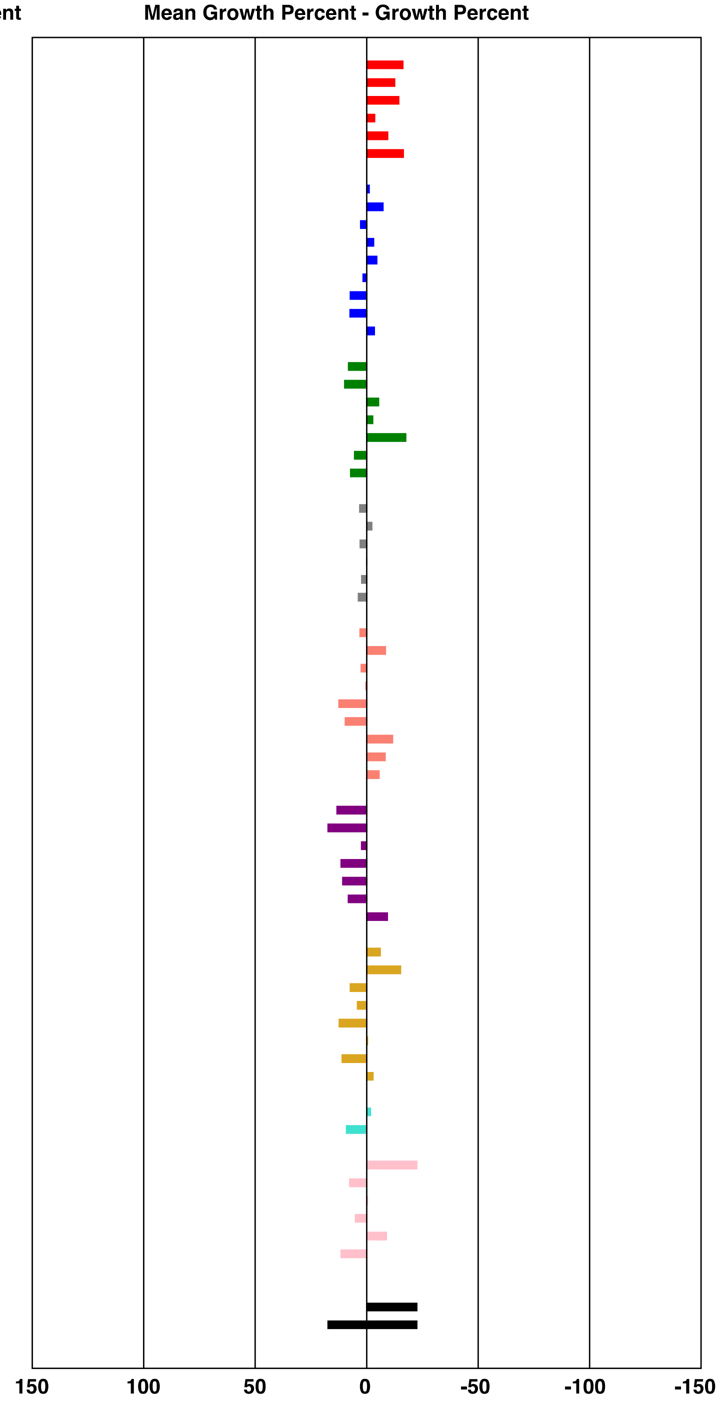


**Supplementary Figure 9.** NCI-60 panel data showing effects of compound **92** on cancer cell proliferation.

## II.XI X-Ray crystallography

### Methods

### Purification, Crystallisation and Structure determination

Recombinant MLLT1 YEATS domain (1-148) was sub-cloned into pNIC-CH which was expressed in *E. coli* Rosetta cultured in terrific broth and induced with 0.5 mM IPTG overnight at 18 °C. The protein incorporating non-cleavable C-terminal $His_6$ tag was purified using $Ni^{2+}$-immobilized metal affinity chromatography (IMAC) and size-exclusion chromatography. Pure protein at 0.45 mM was stored in buffer 25 mM Tris pH 7.5, 300 mM NaCl, 0.2 mM TCEP. Crystallisation experiments were performed at 18 °C using sitting drop vapor diffusion method and either conditions containing i) 25% PEG3350, 0.2 M ammonium sulfate, 0.1 M bis-tris pH 5.5. or ii) 25% medium molecular weight PEG smears.[7] Viable crystals were soaked with the compounds at the concentration of 15 mM overnight and subsequently were cryo-protected using mother liquor supplemented with 25% ethylene glycol. Diffraction data were collected at SLS beamline X06SA using wavelength of 1 Å, and were processed and scaled using XDS[8] and aimless[9], respectively. Initial structure solutions were obtained using molecular replacement method with Phaser[10] and the published coordinates of MLLT1 (PDB ID 5J9S). Manual model rebuilding was performed in COOT[11], alternated with structure refinement in REFMAC[12]. Geometric correctness of the final models was verified by molprobity[13]. Data collection and refinement statistics are summarised in Supplementary Table 10.

| Complex | MLLT1-**92** | MLLT1-**94** |
|---|---|---|
| **PDB accession codes** | **6HT1** | **6HT0** |
| ***Data Collection*** | | |
| Resolution[a] (Å) | 48.84-2.10 (2.17-2.10) | 45.77-1.80 (1.84-1.80) |
| Space group | $P4_32_12$ | $P4_32_12$ |
| Cell dimensions | *a* , *b* = 48.8, 48.8, *c* = 133.2 Å | *a* , *b* = 48.8, *c* = 132.9 Å |
| | $\alpha, \beta, \gamma$ = 90.0° | $\alpha, \beta, \gamma$ = 90.0° |
| No. unique reflections[a] | 10,098 (956) | 15,693 (900) |
| Completeness[a] (%) | 100.0 (100.0 | 100.0 (100.0) |
| I/σI[a] | 13.2 (3.4) | 17.6 (2.6) |
| $R_{merge}$[a] (%) | 0.069 (0.463) | 0.059 (0.774) |
| Redundancy[a] | 7.8 (7.6) | 10.0 (10.3) |
| ***Refinement*** | | |
| No. atoms in refinement (P/L/O)[b] | 1200/ 29/ 89 | 1,221/ 86/ 31 |
| B factor (P/L/O)[b] (Å$^2$) | 53/ 59/ 61 | 35/ 41/ 44 |
| $R_{fact}$ (%) | 20.1 | 20.1 |
| $R_{free}$ (%) | 25.2 | 24.9 |
| rms deviation bond[c] (Å) | 0.015 | 0.016 |
| rms deviation angle[c] (°) | 1.5 | 1.6 |
| ***Molprobity Ramachandran*** | | |
| Favour (%) | 96.45 | 97.86 |
| Outlier (%) | 0 | 0 |

**Supplementary Table 3**. Data collection and refinement statistics of MLLT1-ligand complexes. [a]Values in brackets show the statistics for the highest resolution shells. [b] P/L/O indicates proteins, ligand of interest, and other atoms including waters and solvent molecules, respectively.

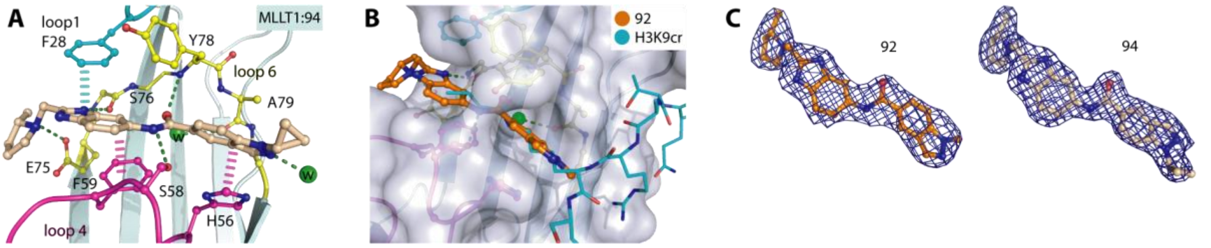


**Supplementary Figure 10**. A) Detailed interactions of compound **94** (beige sticks) with MLLT1 YD (light green sheets), loop 1 (cyan), loop 4 (magenta) and loop 6 (yellow) are also depicted. B) Space occupancy model of compound **92** (orange sticks) in complex with MLLT1 YD (PDB ID 6HT1) overlaid with a co-crystal structure of MLLT3 YD with H3Kcr9 (blue sticks) (PDB ID 5HJB). C) $F_oF_c$ omitted map of compound **92** and **94** contoured at 3 σ from co-crystal structures with MLLT1 YD (PDB ID 6HT1 & 6HT0 respectively).

## II.XII Analysis of gene expression by quantitative polymerase chain reaction (qPCR)

### Methods

MV4;11 cells were cultured at 37ºC in a humidified 5% $CO_2$ atmosphere in RPMI medium containing 10% FBS and 1% L-glutamine. Cells were seeded at a density of 2 x $10^6$ cells/well in 6 well plates pre-coated in polyhema, to enable suspension culture. Cells were incubated with 1 µM of compound **92**, **91**, or 50 nM of **JQ1** for 72 hours. After collecting cells and washing with PBS, RNA was extracted from cells using RNeasy Mini Kit (Qiagen), following the manufacturer's protocol. Total RNA was quantified by Nanodrop and 500 ng of RNA was converted to cDNA using GoScript Reverse Transcription System (Promega). The final cDNA products were quantified by QPCR using FAM dye-labelled Taqman primers and probe sets (see table below). Data was analysed using the comparative cycle threshold ($C_T$) method where $C_T$ values were normalised by subtracting that from an endogenous housekeeping gene (VIC dye-labelled *ACTB*). The gene expression levels from compound-treated cells are presented as relative to those of DMSO treated control cells. Statistical analyses were carried out using two-way ANOVA (GraphPad Prism 7).

| Gene name | Assay ID |
|---|---|
| *MYC* (c-myc) | Hs00905030_m1 |
| *MPO* | Hs00924296_m1 |
| *DDN* | Hs00391784_m1 |
| *CTSG* | Hs00175195_m1 |
| *CD86* | Hs01567026_m1 |
| *ACTB* (Beta Actin) Endogenous Control | 4326315E |

**Supplementary Table 4.** Taqman Gene Expression Assays (Life Technologies). Taqman MGB probes have 5' FAM reporter dye (or VIC) and 3' non-fluorescent quencher.

## III Supplementary References

[1] P. Filippakopoulos, S. Picaud, M. Mangos, T. Keates, J.-P. Lambert, D. Barsyte-Lovejoy, I. Felletar, R. Volkmer, S. Muller, T. Pawson, et al., *Cell* **2012**, *149*, 214–231.

[2] T. Machleidt, C. C. Woodroofe, M. K. Schwinn, J. Méndez, M. B. Robers, K. Zimmerman, P. Otto, D. L. Daniels, T. a Kirkland, K. V Wood, *ACS Chem. Biol.* **2015**, *10*, 1797–1804.

[3] M. Philpott, C. M. Rogers, C. Yapp, C. Wells, J. P. Lambert, C. Strain-Damerell, N. A. Burgess-Brown, A. C. Gingras, S. Knapp, S. Muller, *Epigenetics Chromatin* **2014**, *7*, 14.

[4] M. A. Rapsomaniki, P. Kotsantis, I. E. Symeonidou, N. N. Giakoumakis, S. Taraviras, Z. Lygerou, *Bioinformatics* **2012**, DOI 10.1093/bioinformatics/bts241.

[5] R. D. Phair, S. A. Gorski, T. Misteli, *Methods Enzymol.* **2004**, DOI 10.1016/S0076-6879(03)75025-3.

[6] R. Jafari, H. Almqvist, H. Axelsson, M. Ignatushchenko, T. Lundbäck, P. Nordlund, D. M. Molina, *Nat. Protoc.* **2014**, *9*, 2100–2122.

[7] A. Chaikuad, S. Knapp, F. Von Delft, *Acta Crystallogr. Sect. D Biol. Crystallogr.* **2015**, *71*, 1627–1639.

[8] W. Kabsch, *Acta Crystallogr. Sect. D Biol. Crystallogr.* **2010**, *66*, 125–132.

[9] P. R. Evans, G. N. Murshudov, *Acta Crystallogr. D. Biol. Crystallogr.* **2013**, *69*, 1204–14.

[10] A. J. McCoy, *Methods Mol. Biol.* **2017**, *1607*, 421–453.

[11] P. Emsley, *Acta Crystallogr. Sect. D, Struct. Biol.* **2017**, *73*, 203–210.

[12] A. A. Vagin, R. A. Steiner, A. A. Lebedev, L. Potterton, S. McNicholas, F. Long, G. N. Murshudov, *Acta Crystallogr. D. Biol. Crystallogr.* **2004**, *60*, 2184–95.

[13] C. J. Williams, J. J. Headd, N. W. Moriarty, M. G. Prisant, L. L. Videau, L. N. Deis, V. Verma, D. A. Keedy, B. J. Hintze, V. B. Chen, et al., *Protein Sci.* **2018**, *27*, 293–315.
